# Safety of the Malaria Vaccine Candidate, RTS,S/AS01_E_ in 5 to 17 Month Old Kenyan and Tanzanian Children

**DOI:** 10.1371/journal.pone.0014090

**Published:** 2010-11-29

**Authors:** John Lusingu, Ally Olotu, Amanda Leach, Marc Lievens, Johan Vekemans, Aurélie Olivier, Sarah Benns, Raimos Olomi, Salum Msham, Trudie Lang, Jayne Gould, Karin Hallez, Yolanda Guerra, Patricia Njuguna, Ken O. Awuondo, Anangisye Malabeja, Omar Abdul, Samwel Gesase, Denise Dekker, Lincoln Malle, Sadiki Ismael, Neema Mturi, Chris J. Drakeley, Barbara Savarese, Tonya Villafana, W. Ripley Ballou, Joe Cohen, Eleanor M. Riley, Martha M. Lemnge, Kevin Marsh, Philip Bejon, Lorenz von Seidlein

**Affiliations:** 1 National Institute for Medical Research, Tanga, Tanzania; 2 Centre for Geographic Medicine Research (Coast), KEMRI, Kilifi, Kenya; 3 GlaxoSmithKline Biologicals, Rixensart, Belgium; 4 Joint Malaria Programme, Korogwe, Tanzania; 5 London School of Hygiene and Tropical Medicine, London, United Kingdom; 6 PATH Malaria Vaccine Initiative, Bethesda, Maryland, United States of America; 7 MedImmune, LLC, Gaithersburg, Maryland, United States of America; 8 Nuffield Department of Medicine, Oxford, Maryland, United Kingdom; Queensland Institute of Medical Research, Australia

## Abstract

The malaria vaccine candidate, RTS,S/AS01_E_, showed promising protective efficacy in a trial of Kenyan and Tanzanian children aged 5 to 17 months. Here we report on the vaccine's safety and tolerability. The experimental design was a Phase 2b, two-centre, double-blind (observer- and participant-blind), randomised (1∶1 ratio) controlled trial. Three doses of study or control (rabies) vaccines were administered intramuscularly at 1 month intervals. Solicited adverse events (AEs) were collected for 7 days after each vaccination. There was surveillance and reporting for unsolicited adverse events for 30 days after each vaccination. Serious adverse events (SAEs) were recorded throughout the study period which lasted for 14 months after dose 1 in Korogwe, Tanzania and an average of 18 months post-dose 1 in Kilifi, Kenya. Blood samples for safety monitoring of haematological, renal and hepatic functions were taken at baseline, 3, 10 and 14 months after dose 1. A total of 894 children received RTS,S/AS01_E_ or rabies vaccine between March and August 2007. Overall, children vaccinated with RTS,S/AS01_E_ had fewer SAEs (51/447) than children in the control group (88/447). One SAE episode in a RTS,S/AS01_E_ recipient and nine episodes among eight rabies vaccine recipients met the criteria for severe malaria. Unsolicited AEs were reported in 78% of subjects in the RTS,S/AS01_E_ group and 74% of subjects in the rabies vaccine group. In both vaccine groups, gastroenteritis and pneumonia were the most frequently reported unsolicited AE. Fever was the most frequently observed solicited AE and was recorded after 11% of RTS,S/AS01_E_ doses compared to 31% of doses of rabies vaccine. The candidate vaccine RTS,S/AS01_E_ showed an acceptable safety profile in children living in a malaria-endemic area in East Africa. More data on the safety of RTS,S/AS01_E_ will become available from the Phase 3 programme.

**Trial Registration:**

ClinicalTrials.gov NCT00380393

## Introduction

The most advanced malaria vaccine candidate currently under evaluation is RTS,S/AS [1]. The RTS,S antigen construct has been evaluated in combination with two different adjuvant systems: AS01 and AS02. The ability of the vaccine to protect against malaria has been demonstrated in studies involving North American adults [2], African adults [3], [4] and de-escalating age groups in sub-Saharan Africa [5], [6], [7], [8]. Previous studies have evaluated the safety of RTS,S/AS in a range of populations from varying locations and age groups [3], [5], [7], [8], [9], [10], [11], [12], [13], [14], [15], [16], [17], [18]. By the end of 2009, 1296 individuals had received 3720 doses of RTS,S/AS01 and 2826 individuals had received 7694 doses of RTS,S/AS02 in Phase 1 and 2 studies. To date, all reports have suggested that RTS,S/AS is well tolerated and has a good safety profile. Since preliminary data suggested better immunogenicity with the AS01 adjuvant than AS02 [12], [15], the efficacy of the candidate RTS,S/AS01_E_ against clinical malaria was evaluated in a cohort of Kenyan and Tanzanian children 5 to 17 months of age. RTS,S/AS01_E_ was found to have a protective efficacy of 53% (95% confidence interval [CI], 28% to 69%; p<0.001) over an average follow-up period of 8 months [19]. Here we report on the safety and tolerability of RTS,S/AS01_E_ in African children 5 to 17 months of age.

## Methods

The study was registered at ClinicalTrials.gov (ClinicalTrials.gov number: NCT00380393). Approval was obtained from the Kenyan Medical Research Institute National Ethics Committee, the Tanzanian Medical Research Coordinating Committee, the Central Oxford Research Ethics Committee, the London School of Hygiene and Tropical Medicine Ethics Committee, and the Western Institutional Review Board in Seattle. The study was overseen by an Independent data monitoring committee and local safety monitors, and conducted in accordance with the Helsinki Declaration of 1964 (revised 1996) and Good Clinical Practice guidelines. The protocol for this trial and supporting CONSORT checklist are available as supporting information; see Checklist S1 and Protocol S1.

### Study sites

The study was conducted at two study sites: Korogwe district, Tanzania and Kilifi district, Kenya. Both sites are within 100km of the coast of the Indian Ocean and within 300 km of each other. The study sites have been previously described [19]. Briefly, in Kilifi, Kenya, children were recruited in two administrative locations (Pingilikani and Junju), within the Chonyi area in the southern part of Kilifi District. In Tanzania, children were recruited from the catchment areas of three dispensaries (Ngombezi, Mbagai and Makuyuni) in Korogwe district, Tanga Region. Both sites are malaria endemic, with all year round transmission and two high transmission seasons. There are successful ITN distribution programmes in both countries and artemether/lumefantrine was the first line anti-malarial treatment. There were no insecticide spraying campaigns in the area at the time of the study. Both areas are rural, and most of the population are subsistence farmers.

The participants, who were residents of the study area, were healthy male and female children aged 5 to 17 months at the time of the first vaccination. All children had been previously vaccinated with HBs as part of the Expanded Programme on Immunisation (EPI).

### Sensitisation and informed consent

To obtain informed consent a multistage sensitisation process was undertaken. In both sites the planned trial was first discussed with the village elders. After the elders granted permission public meetings were held during which the trial was explained to all interested villagers. Following the meetings the parents of eligible children were visited by study staff familiar with the village to answer specific questions regarding the participation in the trial. There were subtle differences in conduct of the sensitisation between the two sites which have been described by Lang and coworkers [20]. Following the sensitisation consenting parents were asked to sign a written informed consent using approved Swahili or Giriama consent forms. The thumb print of an illiterate parents on the consent form, countersigned by an independent, literate witness was accepted.

### Study Design

**Figure 1:**
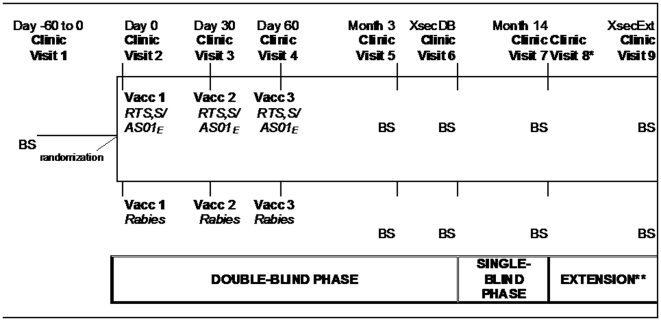
Study design overview. **BS;** Blood Sample, **Vacc;** Vaccination, **Extension;** Extension of the single-blind Phase. **XsecDB**: cross sectional at the end of double-blind Phase (; mean follow-up: 10 months, range: min 7.1; max 12.8 months post-dose 1; SD 1.3). **XsecExt**: cross sectional at the end of the extension Phase; mean follow-up: 18 months (range: min 13.8; max 20.1 months post-dose 1; SD 1.5). *Clinic Visit 8 took place on the same day as clinic Visit 7 and included consent process for participation in the extension Phase (Kilifi only). **Applicable to Kilifi only.

The experimental design was a Phase 2b, randomised (1∶1 ratio) controlled trial. The blinding of study staff and participants differed during the first and the second phase of the trial and is illustrated in Figure 1. The mean duration study children participated in the first phase was 10 months (range: min 7.1; max 12.8 months post-dose 1; SD 1.3). During the first phase of the trial study staff and parents/guardians of participating children were blinded after which the investigators were unblinded but the parents/guardians of participating children remained blinded (Figure 1). In Korogwe, participating children were followed until study conclusion 14 months after dose 1. In Kilifi, children participated in an extended follow-up until the end of the transmission period in October 2008, for all subjects regardless of their enrolment date. This design resulted in different post-vaccination times for individual subjects. The average follow-up period for these subjects was 18 months post-dose 1 in Kenya (range: min 13.8; max 20.1 months post-dose 1; SD 1.5).

### Vaccines

All subjects were randomised to receive either RTS,S/AS01_E_ (GlaxoSmithKline [GSK] Biologicals, Belgium) or a rabies vaccine. RTS,S is composed of two polypeptides which are co-expressed in *Saccharomyces cerevisiae*. One polypeptide RTS includes fractions of the *Plasmodium falciparum* circumsporozoite protein, specifically the CSP repeat region (R), T-cell epitopes (T) fused to “S”, the hepatitis B virus surface antigen (HbsAg) [21]. The other polypeptide, the second S, includes the unfused HbsAg sequence. The two polypeptides assemble spontaneously to form virus like particles. The antigen RTS,S is administered in the proprietary Adjuvant System AS01 comprising liposomes, MPL (3-D-deacylated Monophosphoryl Lipid A) and QS21 (a triterpene glycoside purified from the bark of *Quillaja saponaria*) [21]. The suffix “E” indicates the paediatric dose formulation of AS01, which is the only dose used throughout this trial. The control was a human diploid-cell rabies vaccine (Sanofi-Pasteur). AS01E is presented in a 3 mL monodose vial. One dose contains 25 ug of MPL and 25 ug of Stimulon® QS21 (a triterpene glycoside purified from the bark of Quillaja saponaria) with liposomes. The potency of one dose (1.0 mL) of Sanofi-Pasteur's Human Diploid Cell Rabies Vaccine is at least 2.5 IU of rabies antigen. Sanofi-Pasteur's Human Diploid Cell Rabies Vaccine is a creamy white to orange, freeze-dried vaccine for reconstitution with the diluent prior to use; the reconstituted vaccine is a clear to slightly opaque, colourless suspension.

### Enrolment and vaccinations

Children between 5 months and 17 months of age at the time of first vaccination, who were judged at the time of assessment to have no serious acute or chronic illness as determined by clinical or physical examination, medical history records or laboratory screening tests, were eligible to participate in the trial, provided that the parents or guardians gave their consent. Re-consent was obtained from parents or guardians of subjects for participation in the extension follow-up in Kilifi. Vaccines were administered intramuscularly into the left deltoid at 0, 1, and 2 months. The screening and vaccinations were conducted in clinics which were built or rehabilitated for the purpose of this trial as existing clinic space was in high demand. The RTS,S/AS01_E_ and rabies vaccines were packaged in identical boxes labelled with treatment numbers from a randomization list generated at GSK, and then shipped to the trial sites. Block randomization was used (Block size = 6), with stratification according to study site. Subjects were assigned treatment numbers on the basis of order of attendance at the clinic. Blinding was maintained using a range of measures including opening labelled boxes out of sight of the investigators who evaluated the study end points, study subjects and their parents or guardians, masking syringes and vaccine preparation being undertaken by personnel who took no other part in study-related procedures.

### Definitions

An adverse event (AE) was defined as any untoward medical occurrence in a study participant whether or not considered related to the vaccine. A serious adverse event (SAE) was defined as any untoward medical occurrence that resulted either in death, was life-threatening, required hospitalization or prolongation of existing hospitalization, resulted in disability or was considered severe by the investigators. SAEs were classified according to the preferred term in the Medical Dictionary for Regulatory Activities (MedDRA) [22]. The intensity of all AEs was graded from 1 to 3, with 3 being the most severe (Table 1). For seizures occurring within 7 days of vaccination, data collection and presentation was undertaken according to the Brighton Collaboration guidelines.^22^ Adverse events were treated according to standard medical practice and followed up until resolution. For all general solicited AEs and all unsolicited serious and non-serious AEs the investigators' assessment of causal relationship to vaccination was recorded: ‘yes’, there was a reasonable possibility that the vaccination contributed to the event, or ‘no’, the vaccination was not suspected to have contributed to the AE; there are other more likely causes. Abnormal assessments from interview, examination or laboratory results that were judged by the investigator to be clinically significant were recorded as AEs or SAEs. Severe malaria was defined prospectively according to standard case definitions (Table 2), and confirmed by medical review of patient records.

**Table 1:** Intensity scales for adverse events following vaccination.

Adverse Event	Intensity grade	Parameter
Pain at injection site	0	Absent
	1	Minor reaction to touch
	2	Cries/protests on touch
	3	Cries when limb is moved/spontaneously painful
Swelling at injection site	0	None
	1	<5 mm
	2	5 to 20 mm
	3	>20 mm
Fever*	0	<37.5°C
	1	37.5–38.0°C
	2	>38–39.0°C
	3	>39.0°C
Irritability or Fussiness	0	Behaviour as usual
	1	Crying more than usual/no effect on normal activity
	2	Crying more than usual/interferes with normal activity
	3	Crying that cannot be comforted/prevents normal activity
Drowsiness	0	Behaviour as usual
	1	Drowsiness easily tolerated
	2	Drowsiness that interferes with normal activity
	3	Drowsiness that prevents normal activity
Loss of appetite	0	Appetite as usual
	1	Eating less than usual/no effect on normal activity
	2	Eating less than usual/interferes with normal activity
	3	Not eating at all

*Fever is defined as axillary temperature ≥37.5°C.

**Table 2:** Severe malaria definitions for reporting of SAEs.

**Severe malaria anaemia**	Asexual P. falciparum parasitemia >0 definitive reading
	Hematocrit <15%^1^
	No other more probable cause of illness
**Cerebral malaria**	Asexual P. falciparum parasitemia >0 definitive reading
	Coma score ≤2^2^
	No other identifiable cause of loss of consciousness
**Severe malaria (other)**	Asexual P. falciparum parasitemia >0 definitive reading
	No other more probable cause of illness
	Does not meet criteria for severe malaria anaemia or cerebral malaria
	One of the following:
	Multiple seizures^3^
	Prostration^4^
	Hypoglycemia^5^
	Acidosis
	Circulatory collapse

1 The lowest value recorded by either centrifuge or Coulter counter at any point during the admission was used to determine a case.

2 The coma-score was assessed after correction of hypoglycemia and 60 minutes after control of fits. If fitting could not be controlled within 30 minutes the child was diagnosed with cerebral malaria.

3 Two or more generalized convulsions within a 24-hour period prior to admission.

4 Inability to sit unaided.

5 <2.2 mmol/dL.

### Solicited AEs

Solicited AEs were recorded for 7 days after each vaccination [vaccination Day 0 (at the clinic) and for the 6 subsequent days]. Trained field workers visited each child at home, daily on days 1 through 6 following each vaccination and completed a structured interview regarding local and general AEs. Local solicited AEs included pain at the injection site (defined as a participant's reaction to touch or movement of the injected site or arm), swelling at the injection site (defined as enlargement or bulging). General solicited adverse events included fever (defined as an axillary temperature ≥37.5°C), irritability or fussiness (defined as abnormal crying), drowsiness (defined as sleepiness or tiredness), loss of appetite (defined as eating less than usual). If a field worker found any Grade 3 symptom, the participant was examined by a study physician.

### Unsolicited AEs

Non-serious unsolicited AEs were monitored for 30 days after each vaccination. Events were mainly captured when a child presented unwell to a health facility in the study area where a surveillance system was implemented. The child was reviewed by clinically qualified personnel and treated as required. In addition symptoms identified by field workers at scheduled visits (solicited AE data collection or at a 2-weekly home visit [starting 14 days post-dose 3 and continuing until study end]) were reported to medical research staff, who evaluated the symptom and arranged clinical reviews as appropriate.

### SAEs

SAEs were documented from the administration of the first vaccine dose until the study conclusion at 14 months post-dose 1 in Korogwe and an average of 18 months post-dose 1 in Kilifi. A study physician reviewed medical details of all SAEs before unblinding at the end of both the double-blind phase and the study prior to data analysis (Figure 1). In the case of a death, supplementary information was collected using a verbal autopsy.

### Laboratory tests

Blood samples for safety monitoring of haematological (haemoglobin, white cell count, platelet count), renal (creatinine) and hepatic function (alanine transferase) were collected four times during the study period: at baseline; 3 months post-dose 1, at the end of the double-blind phase (mean 10.1 months, SD 1.3) and 14 months post-dose 1. The severity of abnormal laboratory results was graded (Table 3). When a temperature ≥37.5°C was recorded, a blood film was made and a rapid test (Optimal®) for malaria was conducted. Rapid test results were used to make treatment decisions, but the blood film results (read in duplicate) were used to define the protocol-defined study endpoint, presence or absence of malaria. In Korogwe, biochemical parameters were measured using a dry biochemistry photometer VITROS DT Control II (Orto Clinical Diagnostics, Johnson & Johnson Company, NY, USA). Haematological tests were undertaken using a Sysmex KX-21N cell counter (Sysmex Corporation Kobe, Japan). In Kilifi, biochemical parameters were measured using a Selectra E analyzer (Vital Scientific, The Netherlands) and haematological tests were undertaken using a Coulter Counter AcT 5Diff CP (Beckmann Coulter, Florida, USA). In addition, in Kilifi a microbiology laboratory was available to isolate and identify bacterial pathogens. The study protocol did not require testing for the presence of HIV infection for inclusion. No child was diagnosed with HIV infection during the conduct of the trial.

**Table 3:** Grading scale for laboratory results following vaccination.

	Acceptable limit/normal range	Toxicity grade			
		1	2	3	4
**Haemoglobin**	≥8.0 g/dL	<8.0 g/dL	<6.0 g/dL	<5.0 g/dL	<5.0 g/dL & clinical signs
**Total white cell count** †	≥4.0×10^3^/µL to <17×10^3^/µL	2.5 to 4.0×10^3^/µL	1.5 to 2.4×10^3^/µL	1.0 to 1.4×10^3^/µL	<1.0×10^3^/µL
**Platelets** †	≥75×10^3^/µL	50 to 74×10^3^/µL	25 to 49×10^3^/µL	<25×10^3^/µL	<25×10^3^/µL & clinical signs
**ALT** *	≤60 IU/L	1.1 to 2.5×ULN**	2.6 to 5.0×ULN	5.1 to 10.0×ULN	>10.0×ULN
**Creatinine** *	≤60 µmol/L	1.1 to 1.5×ULN	1.6 to 3.0×ULN	3.1 to 6.0×ULN	>6.0×ULN or requires dialysis

† Grading scale adapted from Division of AIDS table for grading severity of adult and paediatric adverse events December 2004.

*Grading scale adapted from WHO Toxicity Grading Scale for Determining Severity of Adverse Events, February 2003.

**ULN: Upper Limit of Normal.

### Analysis

The safety analysis was performed on the total vaccinated cohort. AEs and SAEs, as well as laboratory results at baseline and post-vaccination, were tabulated according to vaccine group attribution with exact 95% Confidence Intervals (CI). Biochemical and haematological parameters outside the normal range were described; the frequency distribution of results by toxicity grades (Table 3) was tabulated by group. SAS version 8 (SAS, Cary, NC, USA) was used for the data analysis.

## Results

**Figure 2:**
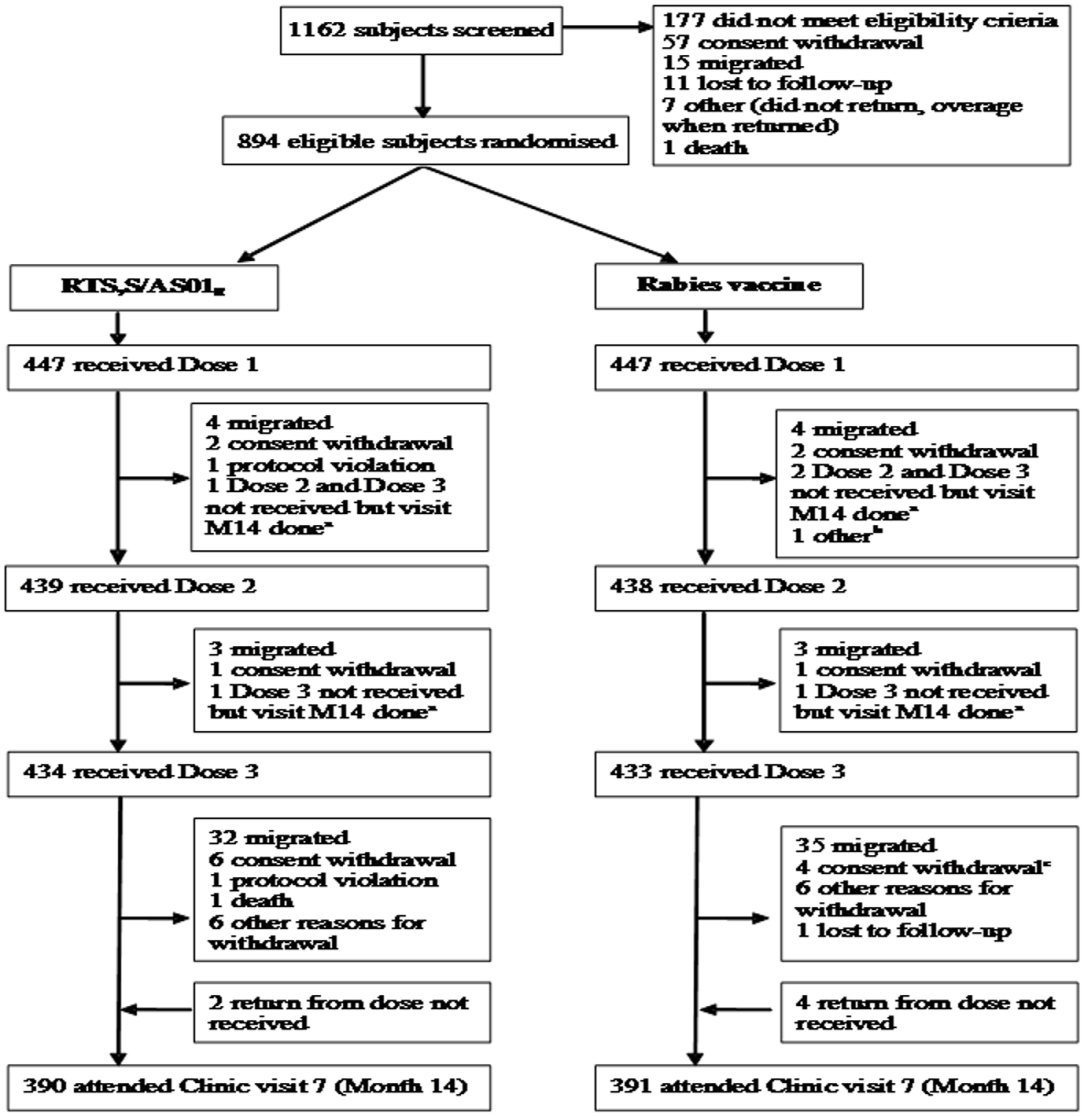
The assembly and disposition of study participants. ^a^ Subjects were temporally out of study area but returned for Month 14 (M14) visit. ^b^ Investigator decided not to continue vaccination as EPI vaccination documentation was not available, this subject returned for Month 14 (M14) visit. ^c^ One subject died after consent withdrawal.

Vaccinations took place over a 6-month period from March until August 2007. The disposition of study participants is shown in Figure 2. A total of 894 subjects received the first dose of either RTS,S/AS01_E_ (N = 447) or the rabies vaccine (N = 447). The mean age of children was 11.4 months (SD 3.6) in the RTS,S/AS01_E_ group and 11.4 months (SD 3.4) in the rabies group. 229 subjects who received RTS,S/AS01_E_ (51%) and 222 children who received the rabies vaccine (50%) were girls. Fourteen months after receiving the first dose, 57 children in the RTS,S/AS01_E_ group (13%) and 56 children in the rabies vaccine group (13%) were lost to follow-up. Of the 447 children enrolled in Kilifi, 349 (78%) participated in the extension follow-up and 327 (73%) completed the last study visit.

We compared the occurrence of solicited local and general AEs within 7 days following each dose and overall (Table 4). Fever was the most frequently observed solicited AE and occurred more frequently after the rabies vaccine (409 of 1318 vaccinations, 31%) than after RTS,S/AS01_E_ (149 of 1320; 11%) (Table 4). All febrile convulsions occurred within the 7 day follow-up period. The second most common solicited AE was pain. Low grade pain (1–2 intensity grade) was recorded after 13% of the RTS,S/AS01_E_ vaccinations (172 of 1320) and 12% of the rabies vaccinations (157 of 1318). No pain with grade 3 intensity was reported. Irritability was recorded following 59 (5%) of 1320 RTS,S/AS01_E_ administrations and following 21 (2%) of 1318 rabies vaccine administrations. Swelling at the vaccination site, drowsiness, and loss of appetite were infrequently observed and similarly distributed among RTS,S/AS01_E_ and rabies vaccine recipients. Unsolicited AEs were equally balanced between both study groups (Table S1). Unsolicited AEs were reported in 78% of subjects in the RTS,S/AS01_E_ group and 74% of subjects in the rabies vaccine group. In both vaccine groups, gastroenteritis and pneumonia were the most frequently reported unsolicited AE^19^.

**Table 4:** Frequency (%) of solicited local and general adverse events within 7 days following each dose and overall.

			RTSS/AS01_E_					Rabies				
Symptom/Sign	Dose	Intensity Grade	N	n	%	95% CI		N	n	%	95% CI	
Pain	Dose 1	Any	**447**	51	11.4	8.6	14.7	**447**	39	8.7	6.3	11.7
	Dose 2	Any	**439**	45	10.3	7.6	13.5	**438**	38	8.7	6.2	11.7
	Dose 3	Any	**434**	76	17.5	14.1	21.4	**433**	80	18.5	14.9	22.5
	Overall	Any	**1320**	172	13.0	11.3	15.0	**1318**	157	11.9	10.2	13.8
Swelling	Dose 1	Any	**447**	20	4.5	2.8	6.8	**447**	12	2.7	1.4	4.6
	Dose 2	Any	**439**	6	1.4	0.5	3.0	**438**	3	0.7	0.1	2.0
		Grade 3	**439**	1	0.2	0.0	1.3	**438**	0	0.0	0.0	0.8
	Dose 3	Any	**434**	8	1.8	0.8	3.6	**433**	1	0.2	0.0	1.3
		Grade 3	**434**	2	0.5	0.1	1.7	**433**	0	0.0	0.0	0.8
	Overall	Any	**1320**	34	2.6	1.8	3.6	**1318**	16	1.2	0.7	2.0
		Grade 3	**1320**	3	0.2	0.0	0.7	**1318**	0	0.0	0.0	0.3
Drowsiness	Dose 1	Any	**447**	44	9.8	7.2	13.0	**447**	28	6.3	4.2	8.9
	Dose 2	Any	**439**	22	5.0	3.2	7.5	**438**	25	5.7	3.7	8.3
		Grade 3	**439**	0	0.0	0.0	0.8	**438**	1	0.2	0.0	1.3
	Dose 3	Any	**434**	15	3.5	1.9	5.6	**433**	11	2.5	1.3	4.5
	Overall	Any	**1320**	81	6.1	4.9	7.6	**1318**	64	4.9	3.8	6.2
		Grade 3	**1320**	0	0.0	0.0	0.3	**1318**	1	0.1	0.0	0.4
Fever	Dose 1	Any	**447**	55	12.3	9.4	15.7	**447**	138	30.9	26.6	35.4
		Grade 3	**447**	1	0.2	0.0	1.2	**447**	2	0.4	0.1	1.6
	Dose 2	Any	**439**	56	12.8	9.8	16.2	**438**	159	36.3	31.8	41.0
		Grade 3	**439**	3	0.7	0.1	2.0	**438**	8	1.8	0.8	3.6
	Dose 3	Any	**434**	38	8.8	6.3	11.8	**433**	112	25.9	21.8	30.3
		Grade 3	**434**	1	0.2	0.0	1.3	**433**	2	0.5	0.1	1.7
	Overall	Any	**1320**	149	11.3	9.6	13.1	**1318**	409	31.0	28.5	33.6
		Grade 3	**1320**	5	0.4	0.1	0.9	**1318**	12	0.9	0.5	1.6
Irritability	Dose 1	Any	**447**	26	5.8	3.8	8.4	**447**	12	2.7	1.4	4.6
	Dose 2	Any	**439**	16	3.6	2.1	5.9	**438**	7	1.6	0.6	3.3
	Dose 3	Any	**434**	17	3.9	2.3	6.2	**433**	2	0.5	0.1	1.7
	Overall	Any	**1320**	59	4.5	3.4	5.7	**1318**	21	1.6	1.0	2.4
Loss of appetite	Dose 1	Any	**447**	34	7.6	5.3	10.5	**447**	28	6.3	4.2	8.9
		Grade 3	**447**	0	0.0	0.0	0.8	**447**	1	0.2	0.0	1.2
	Dose 2	Any	**439**	24	5.5	3.5	8.0	**438**	13	3.0	1.6	5.0
		Grade 3	**439**	0	0.0	0.0	0.8	**438**	1	0.2	0.0	1.3
	Dose 3	Any	**434**	6	1.4	0.5	3.0	**433**	2	0.5	0.1	1.7
	Overall	Any	**1320**	64	4.8	3.8	6.1	**1318**	43	3.3	2.4	4.4
		Grade 3	**1320**	0	0.0	0.0	0.3	**1318**	2	0.2	0.0	0.5

For each dose:

N = number of subjects with at least one administered dose.

n (%) = number of doses followed by the symptom/sign.

For Overall/dose:

N = number of administered doses.

n(%) = number(percentage) of doses followed by at least one type of symptom.

95%CI = Exact 95% confidence interval.

The absence of a grade in the table suggests that none of the participants was included in this grade.

Table 5 presents details of SAE following administration of RTS,S/AS01_E_ and the rabies vaccine. Overall, SAEs occurred more frequently in children in the control group than in the RTS,S/AS01_E_ group: 88/447 children who received the rabies vaccine (20%; 95% CI 16% to 24%) compared to 51/447 children who received RTS,S/AS01_E_ (11%; 95% CI 9% to 15%) had at least one SAE. One SAE episode in a RTS,S/AS01_E_ recipient and nine episodes among eight rabies vaccine recipients met the criteria for severe malaria described (Table 2). Three children in the control group had cerebral malaria and one had severe anaemia. All children treated for severe malaria recovered without sequelae. The most frequently detected SAEs in both groups were pneumonia (n = 42), febrile convulsions (n = 34), *P. falciparum* infection (n = 33), gastroenteritis (n = 32), and upper respiratory tract infections (n = 12). One SAE, a febrile convulsion in a subject from the RTS,S/AS01_E_ group, was considered by the investigator to be causally related to the study vaccine. The child was closely followed and had no sequelae. Two deaths, both following convulsions, were reported. In the RTS,S/AS01_E_ group, a 17-month-old boy died at home 9 months after the third dose of RTS,S/AS01_E_. A 2-year-old boy died 13 months after the third dose of rabies vaccine. The verbal autopsies conducted after the deaths failed to ascertain the aetiologies for either death. Both fatal SAEs were considered by investigators to be unrelated to study vaccination.

**Table 5:** Serious Adverse Events (The number of severe malaria cases have been previously been described [19]. One child in the RTS,S/AS01_E_ and 9 children in the rabies vaccine arm were diagnosed with severe malaria.)

	RTS,S/AS01_E_N = 447				RabiesN = 447			
	n	%	95% CI		n	%	95% CI	
At least one SAE	51	11.4	8.6	14.7	88	19.7	16.1	23.7
At least one SAE excluding malaria related SAEs	50	11.2	8.4	14.5	85	19.0	15.5	23.0
**SAEs occurring in more than one subject**								
Pneumonia	16	3.6	2.1	5.7	26	5.8	3.8	8.4
Febrile convulsion	14	3.1	1.7	5.2	20	4.5	2.8	6.8
Gastroenteritis	10	2.2	1.1	4.1	22	4.9	3.1	7.4
Plasmodium falciparum infection	8	1.8	0.8	3.5	25	5.6	3.7	8.1
Upper respiratory tract infection	8	1.8	0.8	3.5	4	0.9	0.2	2.3
Anaemia	5	1.1	0.4	2.6	11	2.5	1.2	4.4
Urinary tract infection	3	0.7	0.1	1.9	0	0.0	0.0	0.8
Convulsion	3	0.7	0.1	1.9	2	0.4	0.1	1.6
Dysentery	2	0.4	0.1	1.6	1	0.2	0.0	1.2
Asthma	2	0.4	0.1	1.6	1	0.2	0.0	1.2
Bronchiolitis	1	0.2	0.0	1.2	3	0.7	0.1	1.9
Cholera	1	0.2	0.0	1.2	2	0.4	0.1	1.6
Malnutrition	1	0.2	0.0	1.2	2	0.4	0.1	1.6
Rectal prolapse	0	0.0	0.0	0.8	2	0.4	0.1	1.6
Cerebral malaria	0	0.0	0.0	0.8	3	0.7	0.1	1.9
Petroleum distillate poisoning	0	0.0	0.0	0.8	2	0.4	0.1	1.6
Thermal burn	0	0.0	0.0	0.8	2	0.4	0.1	1.6
Marasmus	0	0.0	0.0	0.8	2	0.4	0.1	1.6
Bronchial hyperreactivity	0	0.0	0.0	0.8	2	0.4	0.1	1.6

N = number of subjects with at least one administered dose.

n/% = number/percentage of subjects reporting at least once the symptom.

95% CI = exact 95% confidence interval.

The most frequently observed haematological abnormality was anaemia which was observed in <2% RTS,S/AS01_E_ and <4% rabies vaccine recipients during the 3 post-vaccination blood draws and in similar proportions in both groups overall (Table 6). Abnormal white cell counts and platelet counts were detected in 1% or fewer study participants and in a similar proportion in both vaccine groups. At 14 months post-dose 1, an elevated ALT was recorded in samples from 14 children in the RTS,S/AS01_E_ group and from 16 children in the control group. An elevated creatinine was detected in <1% of study participants and in similar proportions in each vaccine group.

**Table 6:** Frequency (%) of abnormal laboratory results following vaccination.

	Toxicity grade	RTS,S/AS01_E_		Rabies vaccine	
		N = 447		N = 447	
		n	(%)	n	(%)
**Haemoglobin**					
month 3	1	**7**	(1.7)	**10**	(2.4)
month 9	1	**6**	(1.5)	**8**	(2.0)
month 14	1	**5**	(1.3)	**14**	(3.6)
	2	**1**	(0.3)	**0**	(0.0)
**White blood cells**					
month 3	1	**0**	(0.0)	**1**	(0.2)
month 14	1	**0**	(0.0)	**1**	(0.3)
**Platelets**					
month 3	1	**1**	(0.2)	**0**	(0.0)
	2	**2**	(0.5)	**4**	(0.9)
	3	**0**	(0.0)	**1**	(0.2)
month 9	1	**4**	(1.0)	**1**	(0.3)
	2	**1**	(0.3)	**0**	(0.0)
month 14	1	**1**	(0.3)	**1**	(0.3)
	2	**2**	(0.5)	**4**	(1.0)
	3	**1**	(0.3)	**3**	(0.8)
**Alanine transferase**					
month 3	1	**1**	(0.2)	**2**	(0.5)
	2	**0**	(0.0)	**1**	(0.2)
month 9	1	**5**	(1.3)	**8**	(2.0)
	2	**0**	(0.0)	**1**	(0.2)
month 14	1	**11**	(2.8)	**13**	(3.4)
	2	**2**	(0.5)	**2**	(0.5)
	3	**1**	(0.3)	**0**	(0.0)
	4	**0**	(0.0)	**1**	(0.3)
**Creatinine**					
month 9	1	**1**	(0.3)	**0**	(0.0)
	2	**0**	(0.0)	**1**	(0.2)
month 14	1	**0**	(0.0)	**2**	(0.5)

## Discussion

In this study involving children aged 5 to 17 months, living in a malaria-endemic area, a three-dose regimen of RTS,S/AS01_E_ was well tolerated. Fever within 7 days of vaccination occurred less frequently following RTS,S/AS01_E_ (after 11% of doses) than after rabies vaccination (31%). Whilst this occurrence is comparable with some studies^9,14^, it is lower than other trials, in which an incidence up to 48% has been observed^5,23^. Fever could be a factor for vaccine acceptability and adherence to the three dose vaccine schedule. Parents or guardians may be reluctant to vaccinate their child if fever occurred after vaccinations. In our study febrile convulsions were recorded in 3.1% of the RTS,S/AS01_E_ and 4.5% of the control subjects. A single episode of febrile convulsions following a dose of RTS,S/AS01_E_ was considered to be causally related to the study vaccine. A similar case has been previously reported^23^. Post-vaccination febrile seizures are well described, hence the occurrence of febrile seizures will have to be closely monitored in future RTS,S/AS01_E_ trials. Other signs of vaccine reactogenicity including pain, swelling, drowsiness, irritability and loss of appetite tended to be mild and infrequent.

Interestingly, children vaccinated with RTS,S/AS01_E_ had significantly fewer SAEs than children in the control group. In particular, children who had received RTS,S/AS01_E_ recorded less pneumonia or gastroenteritis during the 14-months post-vaccination surveillance period. One potential explanation for this finding is that children who were protected against malaria may have been indirectly protected against other infectious diseases due to overall better health and a less severely and frequently challenged immune system. The implication of this explanation would be a considerable improvement in the health of children living in malaria endemic areas once a protective malaria vaccine becomes universally available^24^.

An important limitation of our trial was that as only 447 children received the RTS,S/AS01_E_ vaccine, the study was not powered to detect a rare event. Considering the very large target population for a malaria vaccine, more data on the safety of RTS,S/AS01_E_ will be needed before licensure and roll-out. A large, multicentre Phase 3 trial of RTS,S/AS01_E_ in which up to 16,000 children will participate is currently under way and will greatly extend the safety database.

## Supporting Information

Table S1 Unsolicited events tables.
(0.20 MB DOC)

Checklist S1 CONSORT Checklist.
(0.22 MB DOC)

Protocol S1 Trial Protocol.
(1.40 MB PDF)

## References

[1] Vekemans J, Ballou WR (2008). Plasmodium falciparum malaria vaccines in development.. Expert Rev Vaccines.

[2] Kester KE, McKinney DA, Tornieporth N, Ockenhouse CF, Heppner DG (2001). Efficacy of recombinant circumsporozoite protein vaccine regimens against experimental Plasmodium falciparum malaria.. J Infect Dis.

[3] Bojang K, Milligan P, Pinder M, Doherty T, Leach A (2009). Five-year safety and immunogenicity of GlaxoSmithKline's candidate malaria vaccine RTS,S/AS02 following administration to semi-immune adult men living in a malaria-endemic region of The Gambia.. Hum Vaccin.

[4] Bojang KA, Milligan PJ, Pinder M, Vigneron L, Alloueche A (2001). Efficacy of RTS,S/AS02 malaria vaccine against Plasmodium falciparum infection in semi-immune adult men in The Gambia: a randomised trial.. Lancet.

[5] Abdulla S, Oberholzer R, Juma O, Kubhoja S, Machera F (2008). Safety and immunogenicity of RTS,S/AS02D malaria vaccine in infants.. N Engl J Med.

[6] Alonso PL, Sacarlal J, Aponte JJ, Leach A, Macete E (2004). Efficacy of the RTS,S/AS02A vaccine against Plasmodium falciparum infection and disease in young African children: randomised controlled trial.. Lancet.

[7] Macete E, Aponte JJ, Guinovart C, Sacarlal J, Ofori-Anyinam O (2007). Safety and immunogenicity of the RTS,S/AS02A candidate malaria vaccine in children aged 1–4 in Mozambique.. Trop Med Int Health.

[8] Macete EV, Sacarlal J, Aponte JJ, Leach A, Navia MM (2007). Evaluation of two formulations of adjuvanted RTS, S malaria vaccine in children aged 3 to 5 years living in a malaria-endemic region of Mozambique: a Phase I/IIb randomized double-blind bridging trial.. Trials.

[9] Aponte JJ, Aide P, Renom M, Mandomando I, Bassat Q (2007). Safety of the RTS,S/AS02D candidate malaria vaccine in infants living in a highly endemic area of Mozambique: a double blind randomised controlled phase I/IIb trial.. Lancet.

[10] Bojang KA, Olodude F, Pinder M, Ofori-Anyinam O, Vigneron L (2005). Safety and immunogenicty of RTS,S/AS02A candidate malaria vaccine in Gambian children.. Vaccine.

[11] Kester KE, Cummings JF, Ockenhouse CF, Nielsen R, Hall BT (2008). Phase 2a trial of 0, 1, and 3 month and 0, 7, and 28 day immunization schedules of malaria vaccine RTS,S/AS02 in malaria-naive adults at the Walter Reed Army Institute of Research.. Vaccine.

[12] Kester KE, Cummings JF, Ofori-Anyinam O, Ockenhouse CF, Krzych U (2009). Randomized, double-blind, phase 2a trial of falciparum malaria vaccines RTS,S/AS01B and RTS,S/AS02A in malaria-naive adults: safety, efficacy, and immunologic associates of protection.. J Infect Dis.

[13] Kester KE, McKinney DA, Tornieporth N, Ockenhouse CF, Heppner DG (2007). A phase I/IIa safety, immunogenicity, and efficacy bridging randomized study of a two-dose regimen of liquid and lyophilized formulations of the candidate malaria vaccine RTS,S/AS02A in malaria-naive adults.. Vaccine.

[14] Lell B, Agnandji S, von Glasenapp I, Haertle S, Oyakhiromen S (2009). A randomized trial assessing the safety and immunogenicity of AS01 and AS02 adjuvanted RTS,S malaria vaccine candidates in children in Gabon.. PLoS One.

[15] Polhemus ME, Remich SA, Ogutu BR, Waitumbi JN, Otieno L (2009). Evaluation of RTS,S/AS02A and RTS,S/AS01B in adults in a high malaria transmission area.. PLoS One.

[16] Sacarlal J, Aide P, Aponte JJ, Renom M, Leach A (2009). Long-term safety and efficacy of the RTS,S/AS02A malaria vaccine in Mozambican children.. J Infect Dis.

[17] Sacarlal J, Aponte JJ, Aide P, Mandomando I, Bassat Q (2008). Safety of the RTS,S/AS02A malaria vaccine in Mozambican children during a Phase IIb trial.. Vaccine.

[18] Stoute JA, Heppner DG, Mason CJ, Siangla J, Opollo MO (2006). Phase 1 safety and immunogenicity trial of malaria vaccine RTS,S/AS02A in adults in a hyperendemic region of western Kenya.. Am J Trop Med Hyg.

[19] Bejon P, Lusingu J, Olotu A, Leach A, Lievens M (2008). Efficacy of RTS,S/AS01E vaccine against malaria in children 5 to 17 months of age.. N Engl J Med.

[20] Lang TA, Gould J, von Seidlein L, Lusingu JP, Mshamu S (2010). Approaching the Community about Screening Children for a Multi-Centre Malaria Vaccine Trial.. Journal of International Health.

[21] Cohen J, Nussenzweig V, Nussenzweig R, Vekemans J, Leach A (2009). From the circumsporozoite protein to the RTS,S/AS candidate vaccine.. Hum Vaccin.

[22] MedDRA (2007).

