# Supplementary material for: Safety of the Malaria Vaccine Candidate, RTS,S/AS01E in 5 to 17 Month Old Kenyan and Tanzanian Children
Source: PLoS One. 2010 Nov 29;5(11):e14090. doi: 10.1371/journal.pone.0014090 (PMC2993932; doi:10.1371/journal.pone.0014090)
Supplement: Table S1 — Unsolicited events tables. (0.20 MB DOC) [file pone.0014090.s001.doc]

Percentage of subjects reporting the occurrence of grade 3 unsolicited symptoms classified by MedDRA Primary System Organ Class and Preferred Term within the 30-day (Days 0-29) post-vaccination period (Total Vaccinated Cohort)

|  | | **RTS,S/AS01E  N = 447** | | | | **Rabies  N = 447** | | | |  |
| --- | --- | --- | --- | --- | --- | --- | --- | --- | --- | --- |
| **Primary System Organ Class (CODE)** | **Preferred Term (CODE)** | **n** | **%** | **95% CI** | | **n** | **%** | **95% CI** | |  |
| At least one symptom |  | 36 | 8.1 | 5.7 | 11.0 | 51 | 11.4 | 8.6 | 14.7 |  |
| Blood and lymphatic system disorders (10005329) | Anaemia (10002034) | 0 | 0.0 | 0.0 | 0.8 | 1 | 0.2 | 0.0 | 1.2 |  |
| Eye disorders (10015919) | Conjunctivitis (10010741) | 2 | 0.4 | 0.1 | 1.6 | 3 | 0.7 | 0.1 | 1.9 |  |
| Gastrointestinal disorders (10017947) | Diarrhoea (10012735) | 1 | 0.2 | 0.0 | 1.2 | 1 | 0.2 | 0.0 | 1.2 |  |
|  | Enteritis (10014866) | 1 | 0.2 | 0.0 | 1.2 | 0 | 0.0 | 0.0 | 0.8 |  |
| Infections and infestations (10021881) | Abscess (10000269) | 1 | 0.2 | 0.0 | 1.2 | 2 | 0.4 | 0.1 | 1.6 |  |
|  | Abscess limb (10050473) | 1 | 0.2 | 0.0 | 1.2 | 0 | 0.0 | 0.0 | 0.8 |  |
|  | Acarodermatitis (10063409) | 0 | 0.0 | 0.0 | 0.8 | 1 | 0.2 | 0.0 | 1.2 |  |
|  | Bacterial infection (10060945) | 1 | 0.2 | 0.0 | 1.2 | 0 | 0.0 | 0.0 | 0.8 |  |
|  | Bronchiolitis (10006448) | 3 | 0.7 | 0.1 | 1.9 | 5 | 1.1 | 0.4 | 2.6 |  |
|  | Cerebral malaria (10063094) | 0 | 0.0 | 0.0 | 0.8 | 1 | 0.2 | 0.0 | 1.2 |  |
|  | Dysentery (10051402) | 2 | 0.4 | 0.1 | 1.6 | 0 | 0.0 | 0.0 | 0.8 |  |
|  | Ear infection (10014011) | 0 | 0.0 | 0.0 | 0.8 | 1 | 0.2 | 0.0 | 1.2 |  |
|  | Gastroenteritis (10017888) | 6 | 1.3 | 0.5 | 2.9 | 11 | 2.5 | 1.2 | 4.4 |  |
|  | Impetigo (10021531) | 5 | 1.1 | 0.4 | 2.6 | 0 | 0.0 | 0.0 | 0.8 |  |
|  | Lower respiratory tract infection (10024968) | 5 | 1.1 | 0.4 | 2.6 | 8 | 1.8 | 0.8 | 3.5 |  |
|  | Otitis media (10033078) | 0 | 0.0 | 0.0 | 0.8 | 1 | 0.2 | 0.0 | 1.2 |  |
|  | Plasmodium falciparum infection (10035500) | 0 | 0.0 | 0.0 | 0.8 | 2 | 0.4 | 0.1 | 1.6 |  |
|  | Pneumonia (10035664) | 6 | 1.3 | 0.5 | 2.9 | 10 | 2.2 | 1.1 | 4.1 |  |
|  | Septic rash (10062657) | 0 | 0.0 | 0.0 | 0.8 | 1 | 0.2 | 0.0 | 1.2 |  |
|  | Skin infection (10040872) | 0 | 0.0 | 0.0 | 0.8 | 2 | 0.4 | 0.1 | 1.6 |  |
|  | Streptococcal sepsis (10048960) | 0 | 0.0 | 0.0 | 0.8 | 1 | 0.2 | 0.0 | 1.2 |  |
|  | Subcutaneous abscess (10042343) | 2 | 0.4 | 0.1 | 1.6 | 1 | 0.2 | 0.0 | 1.2 |  |
|  | Upper respiratory tract infection (10046306) | 4 | 0.9 | 0.2 | 2.3 | 2 | 0.4 | 0.1 | 1.6 |  |
| Injury, poisoning and procedural complications (10022117) | Femur fracture (10016454) | 0 | 0.0 | 0.0 | 0.8 | 1 | 0.2 | 0.0 | 1.2 |  |
|  | Petroleum distillate poisoning (10034761) | 0 | 0.0 | 0.0 | 0.8 | 1 | 0.2 | 0.0 | 1.2 |  |
| Nervous system disorders (10029205) | Convulsion (10010904) | 0 | 0.0 | 0.0 | 0.8 | 1 | 0.2 | 0.0 | 1.2 |  |
|  | Febrile convulsion (10016284) | 2 | 0.4 | 0.1 | 1.6 | 5 | 1.1 | 0.4 | 2.6 |  |
| Reproductive system and breast disorders (10038604) | Balanitis (10004073) | 1 | 0.2 | 0.0 | 1.2 | 0 | 0.0 | 0.0 | 0.8 |  |
| At least one symptom = at least one symptom experienced (regardless of the MedDRA Preferred Term)  N = number of subjects with at least one administered dose  n/% = number/percentage of subjects reporting at least once the symptom  95% CI= exact 95% confidence interval | | | | | | | | | | |
|  | | | | | | | | | | |

Percentage of doses with unsolicited symptoms classified by MEDDRA Primary System Organ Class and Preferred Term within the 30-day (Days 0-29) post-vaccination period (Total Vaccinated Cohort)

|  | | **RTS,S/AS01E  N = 1320** | | | | **Rabies  N = 1318** | | | | |
| --- | --- | --- | --- | --- | --- | --- | --- | --- | --- | --- |
| **Primary System Organ Class (CODE)** | **Preferred Term (CODE)** | **n** | **%** | **95% CI** | | **n** | **%** | **95% CI** | | |
| At least one symptom |  | 539 | 40.8 | 38.2 | 43.5 | 502 | 38.1 | 35.5 | 40.8 | |
| Blood and lymphatic system disorders (10005329) | Anaemia (10002034) | 5 | 0.4 | 0.1 | 0.9 | 4 | 0.3 | 0.1 | 0.8 | |
|  | Lymphadenopathy (10025197) | 1 | 0.1 | 0.0 | 0.4 | 0 | 0.0 | 0.0 | 0.3 | |
| Eye disorders (10015919) | Conjunctivitis (10010741) | 15 | 1.1 | 0.6 | 1.9 | 18 | 1.4 | 0.8 | 2.1 | |
| Gastrointestinal disorders (10017947) | Diarrhoea (10012735) | 21 | 1.6 | 1.0 | 2.4 | 21 | 1.6 | 1.0 | 2.4 | |
|  | Enteritis (10014866) | 1 | 0.1 | 0.0 | 0.4 | 0 | 0.0 | 0.0 | 0.3 | |
|  | Glossitis (10018386) | 1 | 0.1 | 0.0 | 0.4 | 0 | 0.0 | 0.0 | 0.3 | |
|  | Mouth ulceration (10028034) | 1 | 0.1 | 0.0 | 0.4 | 1 | 0.1 | 0.0 | 0.4 | |
|  | Stomatitis (10042128) | 0 | 0.0 | 0.0 | 0.3 | 3 | 0.2 | 0.0 | 0.7 | |
|  | Vomiting (10047700) | 2 | 0.2 | 0.0 | 0.5 | 3 | 0.2 | 0.0 | 0.7 | |
| General disorders and administration site conditions (10018065) | Swelling (10042674) | 0 | 0.0 | 0.0 | 0.3 | 1 | 0.1 | 0.0 | 0.4 | |
| Infections and infestations (10021881) | Abscess (10000269) | 10 | 0.8 | 0.4 | 1.4 | 9 | 0.7 | 0.3 | 1.3 | |
|  | Abscess limb (10050473) | 1 | 0.1 | 0.0 | 0.4 | 2 | 0.2 | 0.0 | 0.5 | |
|  | Abscess of eyelid (10000297) | 0 | 0.0 | 0.0 | 0.3 | 1 | 0.1 | 0.0 | 0.4 | |
|  | Acarodermatitis (10063409) | 8 | 0.6 | 0.3 | 1.2 | 5 | 0.4 | 0.1 | 0.9 | |
|  | Amoebiasis (10001980) | 0 | 0.0 | 0.0 | 0.3 | 1 | 0.1 | 0.0 | 0.4 | |
|  | Amoebic dysentery (10001986) | 3 | 0.2 | 0.0 | 0.7 | 5 | 0.4 | 0.1 | 0.9 | |
|  | Bacterial infection (10060945) | 1 | 0.1 | 0.0 | 0.4 | 4 | 0.3 | 0.1 | 0.8 | |
|  | Body tinea (10005913) | 2 | 0.2 | 0.0 | 0.5 | 2 | 0.2 | 0.0 | 0.5 | |
|  | Bronchiolitis (10006448) | 13 | 1.0 | 0.5 | 1.7 | 15 | 1.1 | 0.6 | 1.9 | |
|  | Bronchitis (10006451) | 1 | 0.1 | 0.0 | 0.4 | 2 | 0.2 | 0.0 | 0.5 | |
|  | Bronchopneumonia (10006469) | 6 | 0.5 | 0.2 | 1.0 | 1 | 0.1 | 0.0 | 0.4 | |
|  | Bullous impetigo (10006563) | 1 | 0.1 | 0.0 | 0.4 | 0 | 0.0 | 0.0 | 0.3 | |
|  | Candidiasis (10007152) | 0 | 0.0 | 0.0 | 0.3 | 1 | 0.1 | 0.0 | 0.4 | |
|  | Cellulitis (10007882) | 2 | 0.2 | 0.0 | 0.5 | 3 | 0.2 | 0.0 | 0.7 | |
|  | Cerebral malaria (10063094) | 0 | 0.0 | 0.0 | 0.3 | 1 | 0.1 | 0.0 | 0.4 | |
|  | Conjunctivitis bacterial (10061784) | 2 | 0.2 | 0.0 | 0.5 | 0 | 0.0 | 0.0 | 0.3 | |
|  | Conjunctivitis viral (10010755) | 0 | 0.0 | 0.0 | 0.3 | 1 | 0.1 | 0.0 | 0.4 | |
|  | Dermatitis infected (10012470) | 3 | 0.2 | 0.0 | 0.7 | 3 | 0.2 | 0.0 | 0.7 | |
|  | Dysentery (10051402) | 9 | 0.7 | 0.3 | 1.3 | 11 | 0.8 | 0.4 | 1.5 | |
|  | Ear infection (10014011) | 3 | 0.2 | 0.0 | 0.7 | 3 | 0.2 | 0.0 | 0.7 | |
|  | Febrile infection (10051998) | 3 | 0.2 | 0.0 | 0.7 | 5 | 0.4 | 0.1 | 0.9 | |
|  | Fungal infection (10017533) | 1 | 0.1 | 0.0 | 0.4 | 0 | 0.0 | 0.0 | 0.3 | |
|  | Fungal skin infection (10017543) | 0 | 0.0 | 0.0 | 0.3 | 1 | 0.1 | 0.0 | 0.4 | |
|  | Furuncle (10017553) | 1 | 0.1 | 0.0 | 0.4 | 0 | 0.0 | 0.0 | 0.3 | |
|  | Gastroenteritis (10017888) | 117 | 8.9 | 7.4 | 10.5 | 92 | 7.0 | 5.7 | 8.5 | |
|  | Helminthic infection (10061201) | 6 | 0.5 | 0.2 | 1.0 | 9 | 0.7 | 0.3 | 1.3 | |
|  | Hookworm infection (10020376) | 1 | 0.1 | 0.0 | 0.4 | 2 | 0.2 | 0.0 | 0.5 | |
|  | Impetigo (10021531) | 28 | 2.1 | 1.4 | 3.1 | 21 | 1.6 | 1.0 | 2.4 | |
|  | Infection parasitic (10021857) | 1 | 0.1 | 0.0 | 0.4 | 1 | 0.1 | 0.0 | 0.4 | |
|  | Influenza (10022000) | 1 | 0.1 | 0.0 | 0.4 | 1 | 0.1 | 0.0 | 0.4 | |
|  | Lower respiratory tract infection (10024968) | 27 | 2.0 | 1.4 | 3.0 | 28 | 2.1 | 1.4 | 3.1 | |
|  | Malaria (10025487) | 4 | 0.3 | 0.1 | 0.8 | 7 | 0.5 | 0.2 | 1.1 | |
|  | Nasopharyngitis (10028810) | 1 | 0.1 | 0.0 | 0.4 | 2 | 0.2 | 0.0 | 0.5 | |
|  | Oral candidiasis (10030963) | 5 | 0.4 | 0.1 | 0.9 | 5 | 0.4 | 0.1 | 0.9 | |
|  | Oral herpes (10067152) | 0 | 0.0 | 0.0 | 0.3 | 1 | 0.1 | 0.0 | 0.4 | |
|  | Otitis externa (10033072) | 2 | 0.2 | 0.0 | 0.5 | 2 | 0.2 | 0.0 | 0.5 | |
|  | Otitis media (10033078) | 13 | 1.0 | 0.5 | 1.7 | 9 | 0.7 | 0.3 | 1.3 | |
|  | Otitis media acute (10033079) | 3 | 0.2 | 0.0 | 0.7 | 3 | 0.2 | 0.0 | 0.7 | |
|  | Otitis media chronic (10033081) | 0 | 0.0 | 0.0 | 0.3 | 1 | 0.1 | 0.0 | 0.4 | |
|  | Parotitis (10034038) | 0 | 0.0 | 0.0 | 0.3 | 1 | 0.1 | 0.0 | 0.4 | |
|  | Plasmodium falciparum infection (10035500) | 0 | 0.0 | 0.0 | 0.3 | 3 | 0.2 | 0.0 | 0.7 | |
|  | Pneumonia (10035664) | 189 | 14.3 | 12.5 | 16.3 | 178 | 13.5 | 11.7 | 15.5 | |
|  | Respiratory tract infection (10062352) | 22 | 1.7 | 1.0 | 2.5 | 14 | 1.1 | 0.6 | 1.8 | |
|  | Rhinitis (10039083) | 27 | 2.0 | 1.4 | 3.0 | 21 | 1.6 | 1.0 | 2.4 | |
|  | Septic rash (10062657) | 0 | 0.0 | 0.0 | 0.3 | 1 | 0.1 | 0.0 | 0.4 | |
|  | Shigella infection (10054178) | 1 | 0.1 | 0.0 | 0.4 | 0 | 0.0 | 0.0 | 0.3 | |
|  | Skin bacterial infection (10052891) | 11 | 0.8 | 0.4 | 1.5 | 9 | 0.7 | 0.3 | 1.3 | |
|  | Skin infection (10040872) | 13 | 1.0 | 0.5 | 1.7 | 12 | 0.9 | 0.5 | 1.6 | |
|  | Streptococcal sepsis (10048960) | 0 | 0.0 | 0.0 | 0.3 | 1 | 0.1 | 0.0 | 0.4 | |
|  | Subcutaneous abscess (10042343) | 7 | 0.5 | 0.2 | 1.1 | 4 | 0.3 | 0.1 | 0.8 | |
|  | Tinea capitis (10043866) | 0 | 0.0 | 0.0 | 0.3 | 1 | 0.1 | 0.0 | 0.4 | |
|  | Tinea infection (10060889) | 1 | 0.1 | 0.0 | 0.4 | 3 | 0.2 | 0.0 | 0.7 | |
|  | Tinea pedis (10043873) | 1 | 0.1 | 0.0 | 0.4 | 1 | 0.1 | 0.0 | 0.4 | |
|  | Tooth abscess (10044016) | 1 | 0.1 | 0.0 | 0.4 | 0 | 0.0 | 0.0 | 0.3 | |
|  | Upper respiratory tract infection (10046306) | 93 | 7.0 | 5.7 | 8.6 | 59 | 4.5 | 3.4 | 5.7 | |
|  | Urinary tract infection (10046571) | 13 | 1.0 | 0.5 | 1.7 | 9 | 0.7 | 0.3 | 1.3 | |
|  | Varicella (10046980) | 2 | 0.2 | 0.0 | 0.5 | 2 | 0.2 | 0.0 | 0.5 | |
|  | Wound infection (10048038) | 0 | 0.0 | 0.0 | 0.3 | 1 | 0.1 | 0.0 | 0.4 | |
|  | Wound sepsis (10058041) | 2 | 0.2 | 0.0 | 0.5 | 1 | 0.1 | 0.0 | 0.4 | |
| Injury, poisoning and procedural complications (10022117) | Arthropod bite (10003399) | 1 | 0.1 | 0.0 | 0.4 | 0 | 0.0 | 0.0 | 0.3 | |
|  | Clavicle fracture (10009245) | 1 | 0.1 | 0.0 | 0.4 | 0 | 0.0 | 0.0 | 0.3 | |
|  | Femur fracture (10016454) | 0 | 0.0 | 0.0 | 0.3 | 1 | 0.1 | 0.0 | 0.4 | |
|  | Hand fracture (10019114) | 1 | 0.1 | 0.0 | 0.4 | 0 | 0.0 | 0.0 | 0.3 | |
|  | Injury (10022116) | 0 | 0.0 | 0.0 | 0.3 | 1 | 0.1 | 0.0 | 0.4 | |
|  | Joint sprain (10023229) | 2 | 0.2 | 0.0 | 0.5 | 1 | 0.1 | 0.0 | 0.4 | |
|  | Limb injury (10061225) | 1 | 0.1 | 0.0 | 0.4 | 0 | 0.0 | 0.0 | 0.3 | |
|  | Petroleum distillate poisoning (10034761) | 1 | 0.1 | 0.0 | 0.4 | 1 | 0.1 | 0.0 | 0.4 | |
|  | Post procedural haematoma (10063188) | 0 | 0.0 | 0.0 | 0.3 | 1 | 0.1 | 0.0 | 0.4 | |
|  | Soft tissue injury (10041291) | 0 | 0.0 | 0.0 | 0.3 | 1 | 0.1 | 0.0 | 0.4 | |
|  | Thermal burn (10053615) | 3 | 0.2 | 0.0 | 0.7 | 6 | 0.5 | 0.2 | 1.0 | |
|  | Wound (10052428) | 1 | 0.1 | 0.0 | 0.4 | 2 | 0.2 | 0.0 | 0.5 | |
| Metabolism and nutrition disorders (10027433) | Anorexia (10002646) | 0 | 0.0 | 0.0 | 0.3 | 1 | 0.1 | 0.0 | 0.4 | |
|  | Kwashiorkor (10023504) | 1 | 0.1 | 0.0 | 0.4 | 0 | 0.0 | 0.0 | 0.3 | |
|  | Pica (10035001) | 2 | 0.2 | 0.0 | 0.5 | 0 | 0.0 | 0.0 | 0.3 | |
| Musculoskeletal and connective tissue disorders (10028395) | Muscular weakness (10028372) | 0 | 0.0 | 0.0 | 0.3 | 1 | 0.1 | 0.0 | 0.4 | |
| Nervous system disorders (10029205) | Convulsion (10010904) | 0 | 0.0 | 0.0 | 0.3 | 1 | 0.1 | 0.0 | 0.4 | |
|  | Epilepsy (10015037) | 0 | 0.0 | 0.0 | 0.3 | 1 | 0.1 | 0.0 | 0.4 | |
|  | Febrile convulsion (10016284) | 4 | 0.3 | 0.1 | 0.8 | 5 | 0.4 | 0.1 | 0.9 | |
| Reproductive system and breast disorders (10038604) | Balanitis (10004073) | 1 | 0.1 | 0.0 | 0.4 | 0 | 0.0 | 0.0 | 0.3 | |
|  | Genital lesion (10061978) | 1 | 0.1 | 0.0 | 0.4 | 0 | 0.0 | 0.0 | 0.3 | |
| Respiratory, thoracic and mediastinal disorders (10038738) | Asthma (10003553) | 3 | 0.2 | 0.0 | 0.7 | 3 | 0.2 | 0.0 | 0.7 | |
|  | Bronchial hyperreactivity (10066091) | 0 | 0.0 | 0.0 | 0.3 | 3 | 0.2 | 0.0 | 0.7 | |
|  | Cough (10011224) | 0 | 0.0 | 0.0 | 0.3 | 1 | 0.1 | 0.0 | 0.4 | |
|  | Restrictive pulmonary disease (10048667) | 0 | 0.0 | 0.0 | 0.3 | 1 | 0.1 | 0.0 | 0.4 | |
| Skin and subcutaneous tissue disorders (10040785) | Blister (10005191) | 1 | 0.1 | 0.0 | 0.4 | 0 | 0.0 | 0.0 | 0.3 | |
|  | Dermatitis (10012431) | 4 | 0.3 | 0.1 | 0.8 | 1 | 0.1 | 0.0 | 0.4 | |
|  | Dermatitis allergic (10012434) | 0 | 0.0 | 0.0 | 0.3 | 1 | 0.1 | 0.0 | 0.4 | |
|  | Dermatitis atopic (10012438) | 1 | 0.1 | 0.0 | 0.4 | 0 | 0.0 | 0.0 | 0.3 | |
|  | Eczema (10014184) | 1 | 0.1 | 0.0 | 0.4 | 0 | 0.0 | 0.0 | 0.3 | |
|  | Linear iga disease (10024515) | 1 | 0.1 | 0.0 | 0.4 | 0 | 0.0 | 0.0 | 0.3 | |
|  | Pain of skin (10033474) | 0 | 0.0 | 0.0 | 0.3 | 1 | 0.1 | 0.0 | 0.4 | |
|  | Pruritus (10037087) | 1 | 0.1 | 0.0 | 0.4 | 0 | 0.0 | 0.0 | 0.3 | |
|  | Rash (10037844) | 0 | 0.0 | 0.0 | 0.3 | 1 | 0.1 | 0.0 | 0.4 | |
|  | Skin ulcer (10040943) | 1 | 0.1 | 0.0 | 0.4 | 1 | 0.1 | 0.0 | 0.4 | |
| At least one symptom = at least one symptom experienced (regardless of the MedDRA Preferred Term)  N = number of administered doses  n/% = number/percentage of doses with the symptom  95% CI= exact 95% confidence interval | | | | | | | | | |  |
|  | | | | | | | | | |  |
