# Supplementary material for: Safety of the Malaria Vaccine Candidate, RTS,S/AS01E in 5 to 17 Month Old Kenyan and Tanzanian Children
Source: PLoS One. 2010 Nov 29;5(11):e14090. doi: 10.1371/journal.pone.0014090 (PMC2993932; doi:10.1371/journal.pone.0014090)
Supplement: Protocol S1 — Trial Protocol. (1.40 MB PDF) [file pone.0014090.s003.pdf]

Sponsor:
GlaxoSmithKline Biologicals
Rue de l'Institut 89, 1330 Rixensart,
Belgium.

**Study vaccines**	GlaxoSmithKline Biologicals' candidate *Plasmodium falciparum* malaria vaccine RTS,S/AS01E (0.5 mL dose) and Sanofi-Pasteur's Rabies Vaccine produced from Human Diploid Cells. Amended 20 December 2006
**eTrack study number and abbreviated title**	106464, Malaria 049
**Investigational New Drug (IND) number**	BB-IND 12937
**Date of approval**	Final, 15 June 2006
**Amendment 1**	Final, 23 October 2006
**Amendment 2**	Final 20 November 2006
**Amendment 3**	Final 20 December 2006
Amendment 4	***Final 17 April 2008***
**Title**	A Phase IIb randomized, double-blind, controlled study of the efficacy against episodes of clinical malaria due to *Plasmodium falciparum* infection of GlaxoSmithKline Biologicals' candidate vaccine RTS,S/AS01E, administered IM according to a 0, 1, 2-month vaccination schedule in children aged 5 months to 17 months living in Tanzania and Kenya.

GlaxoSmithKline Biologicals

Coordinating author Conor Cahill, Scientific Writer

Contributing authors

- W Ripley Ballou, Vice President – World Wide Clinical Development
- Joe Cohen, Vice President – R&D, Vaccines For Emerging Diseases
- Sabine Corachan, Central Study Coordinator
- Marie-Ange Demoitié, Clinical Immuno RO Manager – R&D, Vaccines for Emerging Diseases
- Marie Claude Dubois, Project Manager – Disease Area Programme Vaccines for Emerging Diseases
- Amanda Leach, Clinical Development Manager
- Marc Lievens, Statistician
- Philip Moris, Scientist, Human CMI
- Opokua Ofori-Anyinam, Clinical Development Manager
- Isabelle Ramboer, Clinical Study Coordinator
- Jean François Stallaert, Clinical Study Coordinator
- Joelle Thonnard, Safety Physician, Biological Clinical Safety & Pharmacovigilance
- Marie Chantal Uwamwezi, Scientist – Regulatory Development Unit III
- Wendy Valinski, US Regulatory Affairs
- Johan Vekemans, Clinical Development Manager
- Ziad Zeinoun, Safety Coordinator

Copyright 2008 the GlaxoSmithKline group of companies. All rights reserved. Unauthorized copying or use of this information is prohibited.

**eTrack study number**	106464
**eTrack abbreviated title**	Malaria 049
**IND number**	BB-IND 12937
**Date of approval**	Final, 15 June 2006
**Amendment 1**	23 October 2006
**Amendment 2**	Final 20 November 2006
**Amendment 3**	Final 20 December 2006
Amendment 4	***Final 17 April 2008***
**Title**	A Phase IIb randomized, double-blind, controlled study of the efficacy against episodes of clinical malaria due to *Plasmodium falciparum* infection of GlaxoSmithKline Biologicals' candidate vaccine RTS,S/AS01E, administered IM according to a 0, 1, 2-month vaccination schedule in children aged 5 months to 17 months living in Tanzania and Kenya.

Joint Malaria Programme (JMP) Korogwe, Tanzania

- Chris Drakeley, Local Trial Planning Committee
- Roly Gosling, Local Trial Planning Committee
- Brian Greenwood, Trial Coordinating Committee
- Martha Lemnge, Local Trial Planning Committee
- John Lusingu, Co-principal Investigator
- Paul Milligan, Trial Coordinating Committee
- Raimos Olomi, Local Trial Planning Committee
- Hugh Reyburn, Trial Coordinating Committee
- Eleanor Riley, Trial Coordinating Committee
- Thor Theander, Trial Coordinating Committee
- Lorenz von Seidlein, Co-principal Investigator

Kenya Medical Research Institute (KEMRI) Wellcome Collaborative Research Programme, Kilifi, Kenya

- Philip Bejon
- Trudie Lang
- Brett Lowe
- Kevin Marsh
- Ally Olotu
- Norbert Peshu

Malaria Vaccine Initiative (MVI) at Program for Appropriate Technology in Health (PATH) Contributing Authors

- Terrell Carter, Technical Coordinator
- Carolyn Petersen, Director, Clinical & Regulatory Affairs
- Barbara Savarese, Senior Program Officer
- Marla Sillman, Technical Coordinator
- Tonya Villafana, Senior Program Officer

Amended 20 November 2006

GlaxoSmithKline Biologicals will act as sponsor for this trial

63f195821c4e0e87c982cb189f3de2c4

**eTrack study number**	106464
**eTrack abbreviated title**	Malaria 049
**IND number**	BB-IND 12937
**Date of approval**	Final, 15 June 2006
**Amendment 1**	23 October 2006
**Amendment 2**	Final 20 November 2006
**Amendment 3**	Final 20 December 2006
Amendment 4	***Final 17 April 2008***
**Title**	A Phase IIb randomized, double-blind, controlled study of the efficacy against episodes of clinical malaria due to *Plasmodium falciparum* infection of GlaxoSmithKline Biologicals' candidate vaccine RTS,S/AS01E, administered IM according to a 0, 1, 2-month vaccination schedule in children aged 5 months to 17 months living in Tanzania and Kenya.

Sponsor signatory approval

~~**Sponsor signatory:**~~	~~W Ripley Ballou~~<br>~~Vice President – World Wide Clinical Development~~
~~**Signature:**~~	____________________________
~~**Date:**~~	____________________________

63f195821c4e0e87c982cb189f3de2c4

Sponsor Information

eTrack study number 106464

eTrack abbreviated title Malaria 049

IND number BB-IND 12937

1. Co-principal Investigators (JMP)

John Lusingu

Joint Malaria Programme,
PO Box 2228, Korogwe,
Tanzania.

Tel: +255.272.64.79.57
Mobile: +255.578.767.95.15
email: jpalusingu@yahoo.co.uk

Lorenz von Seidlein

Joint Malaria Programme,
PO Box 2228, Korogwe,
Tanzania

Tel: +255.272.64.20.44
Fax: +255.272.64.36.88
Mobile: +255.745.10.96.70
email: lseidlein@ivi.int

2. Co-principal Investigators (KEMRI)

Philip Bejon

Kilifi KEMRI-Wellcome Trust, Collaborative Programme,
PO Box 230, Kilifi,
Kenya.

Tel: +44.191.284.11.86
Mobile: +44.770.434.40.07
email: pbejon@kilifi.mimcom.net

Kevin Marsh

Kilifi KEMRI-Wellcome Trust, Collaborative Programme,
PO Box 230, Kilifi,
Kenya.

Tel: +254.733.79.72.18
Mobile: +254.152.50.43
email: kmarsh@kilifi.mimcom.net

Ally Olotu

Kilifi KEMRI-Wellcome Trust, Collaborative Programme,
PO Box 230, Kilifi,
Kenya.

Tel: +254.415.225.35
aolotu@kilifi.mimcom.net

3. Medical Monitor

Amanda Leach
Clinical Development Manager,
GlaxoSmithKline Biologicals,
Rue de l'Institut 89, 1330 Rixensart,
Belgium.

Tel: +32.2.656.77.88
Mobile: +32.476.77.79.79
Fax: +32.2.656.80.44
email: amanda.leach@gskbio.com

eTrack study number 106464

eTrack abbreviated title Malaria 049

IND number BB-IND 12937

4. Central Study Coordinator (JMP)

Jean François Stallaert
Central Study Coordinator,
GlaxoSmithKline Biologicals,
Rue de l'Institut 89, 1330 Rixensart,
Belgium.
Tel: +32.2.656.***94.28***
Fax: +32.2.656.80.44
email: ***jean-francois.j.stallaert@gskbio.com***

Amended 17 April 2008

5. Central Study Coordinator (KEMRI)

Jean François Stallaert
Central Study Coordinator,
GlaxoSmithKline Biologicals,
Rue de l'Institut 89, 1330 Rixensart,
Belgium.
Tel: +32.2.656.94.28
Fax: +32.2.656.80.44
email: jean-francois.j.stallaert@gskbio.com

63f195821c4e0e87c982cb189f3de2c4

eTrack study number 106464

eTrack abbreviated title Malaria 049

IND number BB-IND 12937

7. Study Contacts for Reporting of a Serious Adverse Event occurring at JMP

All of the contacts listed below must be informed of each SAE occurring at JMP.

Study Contact at GSK Biologicals for Reporting Serious Adverse Events
Manager Clinical Safety Vaccines

GSK Biologicals Clinical Safety Physician,
GlaxoSmithKline Biologicals,
Rue de l'Institut 89, 1330 Rixensart,
Belgium.

Tel: +32.2.656.87.98
Fax: +32.2.656.80.09
Mobile phone for 7/7 day availability: +32.477.40.47.13 or +32.472.90.66.00 or +32.474.53.48.68
email: rix.ct-safety-vac@gskbio.com

Amended 23 October 2006

Central Study Coordinator (JMP)

Jean François Stallaert
Central Study Coordinator,
GlaxoSmithKline Biologicals,
Rue de l'Institut 89, 1330 Rixensart,
Belgium.
Tel: +32.2.656.***94.28***
Fax: +32.2.656.80.44
email: ***jean-francois.j.stallaert@gskbio.com***

Amended 17 April 2008

Clinical Research Associate (JMP)

Ezekiel Oenga,
Clinical Research Associate,
GlaxoSmithKline, PO Box 78392-00507,
Nairobi, Kenya.

Tel: +254 20 6933 322
Fax: +254 20 558 329
email: ezekiel.r.oenga@gsk.com

Amended 23 October 2006

Local Safety Monitor (JMP)

Dr Mberesero,

Safi Medics,
Tanga,
Tanzania.

Tel: +255.264.46.46
email: +255.748.40.83.33

Malaria Vaccine Initiative

Carolyn Petersen,
Malaria Vaccine Initiative, PATH,
7500 Old Georgetown Road, 12th Floor,
Bethesda, MD 20814,
USA.

Tel: +1.240.395.27.16
or Tel: +1.240.395.27.00
Fax: +1.240.395.25.91
email: sae@malariavaccine.org

alternate email:
cpetersen@malariavaccine.org

eTrack study number 106464

eTrack abbreviated title Malaria 049

IND number BB-IND 12937

8. Study Contacts for Reporting of a Serious Adverse Event occurring at KEMRI

All of the contacts listed below must be informed of each SAE occurring at KEMRI.

Study Contact at GSK Biologicals for Reporting Serious Adverse Events
Manager Clinical Safety Vaccines

GSK Biologicals Clinical Safety Physician,
GlaxoSmithKline Biologicals,
Rue de l'Institut 89, 1330 Rixensart,
Belgium.

Tel: +32.2.656.87.98
Fax: +32.2.656.80.09
Mobile phone for 7/7 day availability: +32.477.40.47.13 *or* +32.472.90.66.00 *or* +32.474.53.48.68
email: rix.ct-safety-vac@gskbio.com

Central Study Coordinator (KEMRI)

Jean François Stallaert,
Central Study Coordinator,
GlaxoSmithKline Biologicals,
Rue de l'Institut 89, 1330 Rixensart,
Belgium.

Tel: +32.2.656.94.28
Fax: +32.2.656.80.44
email: jean-francois.j.stallaert@gskbio.com

Clinical Research Associate (KEMRI)

Ezekiel Oenga,
Clinical Research Associate,
GlaxoSmithKline, PO Box 78392-00507,
Nairobi, Kenya.
Tel: +254 20 6933 322
Fax: +254 20 558 329
email: ezekiel.r.oenga@gsk.com

Amended 23 October 2006

Local Safety Monitor (KEMRI)

James Alexander Berkley,
KEMRI/Wellcome Trust,
PO Box 230,
Kilifi 80108,
Kenya.

Tel +254.41.52.25.35
Fax +254.4152.23.90
Mobile +254.720.86.70.11

email:jberkley@kilifi.kemri-wellcome.org

Malaria Vaccine Initiative

Carolyn Petersen,
Malaria Vaccine Initiative, PATH,
7500 Old Georgetown Road, 12th Floor,
Bethesda, MD 20814, USA.

Tel: +1.240.395.27.16
or Tel: +1.240.395.27.00
Fax: +1.240.395.25.91
email: sae@malariavaccine.org

alternate email:
cpetersen@malariavaccine.org

eTrack study number 106464

eTrack abbreviated title Malaria 049

IND number BB-IND 12937

8. Study Contact for Emergency Code Break

GSK Biologicals Clinical Safety Physician,
GlaxoSmithKline Biologicals,
Rue de l'Institut 89, 1330 Rixensart,
Belgium.

Tel: +32.2.656.87.98
Fax: +32.2.656.80.09
Mobile phone for 7/7 day availability: +32.477.40.47.13 *or* +32.472.90.66.00 *or* +32.474.53.48.68

9. Study Centers

1: Joint Malaria Programme (JMP), Korogwe, Tanzania.

2: Kenya Medical Research Institute (KEMRI) Wellcome Trust, Collaborative Programme, Kilifi, Kenya.

63f195821c4e0e87c982cb189f3de2c4

Investigator Agreements

**eTrack study number**	106464
**eTrack abbreviated title**	Malaria 049
**IND number**	BB-IND 12937
**Title**	A Phase IIb randomized, double-blind, controlled study of the efficacy against episodes of clinical malaria due to *Plasmodium falciparum* infection of GlaxoSmithKline Biologicals' candidate vaccine RTS,S/AS01E, administered IM according to a 0, 1, 2-month vaccination schedule in children aged 5 months to 17 months living in Tanzania and Kenya.

I agree:

- To assume responsibility for the proper conduct of the study at this site.
- To conduct the study in compliance with this protocol, any mutually agreed future protocol amendments, and with any other study conduct procedures provided by GlaxoSmithKline Biologicals (GSK Biologicals).
- Not to implement any changes to the protocol without agreement from the sponsor and prior review and written approval from the Institutional Review Board (IRB) or Independent Ethics Committee (IEC), except where necessary to eliminate an immediate hazard to the subjects, or for administrative aspects of the study (where permitted by all applicable regulatory requirements).
- That I am thoroughly familiar with the appropriate use of the vaccines, as described in this protocol, and any other information provided by the sponsor, including, but not limited to, the following: the current Investigator's Brochure (IB) or equivalent document, IB supplement (if applicable), prescribing information (in the case of a marketed vaccine).
- That I am aware of, and will comply with, "Good Clinical Practices" (GCP) and all applicable regulatory requirements.
- To ensure that all persons assisting me with the study are adequately informed about the GSK Biologicals investigational product(s) and other study-related duties and functions as described in the protocol.
- That I have been informed that certain regulatory authorities require the sponsor to obtain and supply, as necessary, details about the investigator's ownership interest in the sponsor or the investigational product, and more generally about his/her financial ties with the sponsor. GSK Biologicals will use and disclose the information solely for the purpose of complying with regulatory requirements.

Hence I:

- Agree to supply GSK Biologicals with any necessary information regarding ownership interest and financial ties (including those of my spouse and dependent children).
- Agree to promptly update this information if any relevant changes occur during the course of the study and for 1 year following completion of the study.
- Agree that GSK Biologicals may disclose any information it has about such ownership interests and financial ties to regulatory authorities.
- Agree to provide GSK Biologicals with an updated Curriculum Vitae and other FDA required documents.

~~John Lusingu~~ ~~Co-principal Investigator, Joint Malaria Programme (JMP)~~
~~Korogwe, Tanzania~~

______________________________ ______________________
~~Investigator signature~~ ~~Date~~

**eTrack study number**	106464
**eTrack abbreviated title**	Malaria 049
**IND number**	BB-IND 12937
**Title**	A Phase IIb randomized, double-blind, controlled study of the efficacy against episodes of clinical malaria due to *Plasmodium falciparum* infection of GlaxoSmithKline Biologicals' candidate vaccine RTS,S/AS01E, administered IM according to a 0, 1, 2-month vaccination schedule in children aged 5 months to 17 months living in Tanzania and Kenya.

I agree:

- To assume responsibility for the proper conduct of the study at this site.
- To conduct the study in compliance with this protocol, any mutually agreed future protocol amendments, and with any other study conduct procedures provided by GlaxoSmithKline Biologicals (GSK Biologicals).
- Not to implement any changes to the protocol without agreement from the sponsor and prior review and written approval from the Institutional Review Board (IRB) or Independent Ethics Committee (IEC), except where necessary to eliminate an immediate hazard to the subjects, or for administrative aspects of the study (where permitted by all applicable regulatory requirements).
- That I am thoroughly familiar with the appropriate use of the vaccines, as described in this protocol, and any other information provided by the sponsor, including, but not limited to, the following: the current Investigator's Brochure (IB) or equivalent document, IB supplement (if applicable), prescribing information (in the case of a marketed vaccine).
- That I am aware of, and will comply with, "Good Clinical Practices" (GCP) and all applicable regulatory requirements.
- To ensure that all persons assisting me with the study are adequately informed about the GSK Biologicals investigational product(s) and other study-related duties and functions as described in the protocol.
- That I have been informed that certain regulatory authorities require the sponsor to obtain and supply, as necessary, details about the investigator's ownership interest in the sponsor or the investigational product, and more generally about his/her financial ties with the sponsor. GSK Biologicals will use and disclose the information solely for the purpose of complying with regulatory requirements.

63f195821c4e0e87c982cb189f3de2c4

Hence I:

- Agree to supply GSK Biologicals with any necessary information regarding ownership interest and financial ties (including those of my spouse and dependent children).
- Agree to promptly update this information if any relevant changes occur during the course of the study and for 1 year following completion of the study.
- Agree that GSK Biologicals may disclose any information it has about such ownership interests and financial ties to regulatory authorities.
- Agree to provide GSK Biologicals with an updated Curriculum Vitae and other FDA required documents.

~~Lorenz von Seidlein~~ ~~Co-principal Investigator, Joint Malaria Programme (JMP) Korogwe, Tanzania~~

_______________________________________ _______________________

~~Investigator signature~~ ~~Date~~

**eTrack study number**	106464
**eTrack abbreviated title**	Malaria 049
**IND number**	BB-IND 12937
**Title**	A Phase IIb randomized, double-blind, controlled study of the efficacy against episodes of clinical malaria due to *Plasmodium falciparum* infection of GlaxoSmithKline Biologicals' candidate vaccine RTS,S/AS01E, administered IM according to a 0, 1, 2-month vaccination schedule in children aged 5 months to 17 months living in Tanzania and Kenya.

I agree:

- To assume responsibility for the proper conduct of the study at this site.
- To conduct the study in compliance with this protocol, any mutually agreed future protocol amendments, and with any other study conduct procedures provided by GlaxoSmithKline Biologicals (GSK Biologicals).
- Not to implement any changes to the protocol without agreement from the sponsor and prior review and written approval from the Institutional Review Board (IRB) or Independent Ethics Committee (IEC), except where necessary to eliminate an immediate hazard to the subjects, or for administrative aspects of the study (where permitted by all applicable regulatory requirements).
- That I am thoroughly familiar with the appropriate use of the vaccines, as described in this protocol, and any other information provided by the sponsor, including, but not limited to, the following: the current Investigator's Brochure (IB) or equivalent document, IB supplement (if applicable), prescribing information (in the case of a marketed vaccine).
- That I am aware of, and will comply with, "Good Clinical Practices" (GCP) and all applicable regulatory requirements.
- To ensure that all persons assisting me with the study are adequately informed about the GSK Biologicals investigational product(s) and other study-related duties and functions as described in the protocol.
- That I have been informed that certain regulatory authorities require the sponsor to obtain and supply, as necessary, details about the investigator's ownership interest in the sponsor or the investigational product, and more generally about his/her financial ties with the sponsor. GSK Biologicals will use and disclose the information solely for the purpose of complying with regulatory requirements.

63f195821c4e0e87c982cb189f3de2c4

Hence I:

- Agree to supply GSK Biologicals with any necessary information regarding ownership interest and financial ties (including those of my spouse and dependent children).
- Agree to promptly update this information if any relevant changes occur during the course of the study and for 1 year following completion of the study.
- Agree that GSK Biologicals may disclose any information it has about such ownership interests and financial ties to regulatory authorities.
- Agree to provide GSK Biologicals with an updated Curriculum Vitae and other FDA required documents.

~~Philip Bejon~~	~~Co-principal Investigator, Kenya Medical Research Institute (KEMRI) Wellcome Collaborative Research Programme, Kilifi, Kenya~~

~~Investigator signature~~	~~Date~~

**eTrack study number**	106464
**eTrack abbreviated title**	Malaria 049
**IND number**	BB-IND 12937
**Title**	A Phase IIb randomized, double-blind, controlled study of the efficacy against episodes of clinical malaria due to *Plasmodium falciparum* infection of GlaxoSmithKline Biologicals' candidate vaccine RTS,S/AS01E, administered IM according to a 0, 1, 2-month vaccination schedule in children aged 5 months to 17 months living in Tanzania and Kenya.

I agree:

- To assume responsibility for the proper conduct of the study at this site.
- To conduct the study in compliance with this protocol, any mutually agreed future protocol amendments, and with any other study conduct procedures provided by GlaxoSmithKline Biologicals (GSK Biologicals).
- Not to implement any changes to the protocol without agreement from the sponsor and prior review and written approval from the Institutional Review Board (IRB) or Independent Ethics Committee (IEC), except where necessary to eliminate an immediate hazard to the subjects, or for administrative aspects of the study (where permitted by all applicable regulatory requirements).
- That I am thoroughly familiar with the appropriate use of the vaccines, as described in this protocol, and any other information provided by the sponsor, including, but not limited to, the following: the current Investigator's Brochure (IB) or equivalent document, IB supplement (if applicable), prescribing information (in the case of a marketed vaccine).
- That I am aware of, and will comply with, "Good Clinical Practices" (GCP) and all applicable regulatory requirements.
- To ensure that all persons assisting me with the study are adequately informed about the GSK Biologicals investigational product(s) and other study-related duties and functions as described in the protocol.
- That I have been informed that certain regulatory authorities require the sponsor to obtain and supply, as necessary, details about the investigator's ownership interest in the sponsor or the investigational product, and more generally about his/her financial ties with the sponsor. GSK Biologicals will use and disclose the information solely for the purpose of complying with regulatory requirements.

Hence I:

- Agree to supply GSK Biologicals with any necessary information regarding ownership interest and financial ties (including those of my spouse and dependent children).
- Agree to promptly update this information if any relevant changes occur during the course of the study and for 1 year following completion of the study.
- Agree that GSK Biologicals may disclose any information it has about such ownership interests and financial ties to regulatory authorities.
- Agree to provide GSK Biologicals with an updated Curriculum Vitae and other FDA required documents.

~~Kevin Marsh~~	~~Co-principal Investigator, Kenya Medical Research Institute (KEMRI) Wellcome Collaborative Research Programme, Kilifi, Kenya~~

~~Investigator signature~~	~~Date~~

63f195821c4e0e87c982cb189f3de2c4

**eTrack study number**	106464
**eTrack abbreviated title**	Malaria 049
**IND number**	BB-IND 12937
**Title**	A Phase IIb randomized, double-blind, controlled study of the efficacy against episodes of clinical malaria due to *Plasmodium falciparum* infection of GlaxoSmithKline Biologicals' candidate vaccine RTS,S/AS01E, administered IM according to a 0, 1, 2-month vaccination schedule in children aged 5 months to 17 months living in Tanzania and Kenya.

I agree:

- To assume responsibility for the proper conduct of the study at this site.
- To conduct the study in compliance with this protocol, any mutually agreed future protocol amendments, and with any other study conduct procedures provided by GlaxoSmithKline Biologicals (GSK Biologicals).
- Not to implement any changes to the protocol without agreement from the sponsor and prior review and written approval from the Institutional Review Board (IRB) or Independent Ethics Committee (IEC), except where necessary to eliminate an immediate hazard to the subjects, or for administrative aspects of the study (where permitted by all applicable regulatory requirements).
- That I am thoroughly familiar with the appropriate use of the vaccines, as described in this protocol, and any other information provided by the sponsor, including, but not limited to, the following: the current Investigator's Brochure (IB) or equivalent document, IB supplement (if applicable), prescribing information (in the case of a marketed vaccine).
- That I am aware of, and will comply with, "Good Clinical Practices" (GCP) and all applicable regulatory requirements.
- To ensure that all persons assisting me with the study are adequately informed about the GSK Biologicals investigational product(s) and other study-related duties and functions as described in the protocol.
- That I have been informed that certain regulatory authorities require the sponsor to obtain and supply, as necessary, details about the investigator's ownership interest in the sponsor or the investigational product, and more generally about his/her financial ties with the sponsor. GSK Biologicals will use and disclose the information solely for the purpose of complying with regulatory requirements.

63f195821c4e0e87c982cb189f3de2c4

Hence I:

- Agree to supply GSK Biologicals with any necessary information regarding ownership interest and financial ties (including those of my spouse and dependent children).
- Agree to promptly update this information if any relevant changes occur during the course of the study and for 1 year following completion of the study.
- Agree that GSK Biologicals may disclose any information it has about such ownership interests and financial ties to regulatory authorities.
- Agree to provide GSK Biologicals with an updated Curriculum Vitae and other FDA required documents.

~~Ally Olotu~~	~~Co-principal Investigator, Kenya Medical Research Institute (KEMRI) Wellcome Collaborative Research Programme, Kilifi, Kenya~~

~~Investigator signature~~	~~Date~~

Synopsis

Title A Phase IIb randomized, double-blind, controlled study of the efficacy against episodes of clinical malaria due to *Plasmodium falciparum* infection of GlaxoSmithKline Biologicals' candidate vaccine RTS,S/AS01E, administered IM according to a 0, 1, 2-month vaccination schedule in children aged 5 months to 17 months living in Tanzania and Kenya.

Indication/Study population Primary immunization of healthy male and female children aged 5 months to 17 months at enrolment, if eligible according to inclusion and exclusion criteria.

Rationale The RTS,S/AS01E candidate malaria vaccine is being developed for the routine immunization of infants and children living in malaria-endemic areas as part of the Expanded Program of Immunization (EPI). The RTS,S/AS01E vaccine candidate consists of sequences of the circumsporozoite (CS) protein and hepatitis B surface antigen (HBsAg) with the proprietary adjuvant AS01E (proprietary liposomes, MPL® and Stimulon ® QS21 immunostimulants).

The first RTS,S-based malaria vaccine to be evaluated in children was RTS,S/AS02A (0.25 mL dose). The AS02A adjuvant consists of a proprietary oil-in-water emulsion and two immunostimulants, MPL® and Stimulon® QS21. The RTS,S/AS02A vaccine has demonstrated 30% efficacy against clinical malaria and 58% efficacy against severe malaria disease in children aged 1 to 4 years (Malaria-026). In addition, in that study the vaccine demonstrated a favorable safety profile and induction of protective anti-HBs antibody levels.

The RTS,S/AS01 vaccines have been developed in parallel with the RTS,S/AS02 vaccines with the aim of improving the immune response and increasing vaccine efficacy. A recent challenge study with RTS,S/AS01B in malaria-naïve adults (Malaria-027) has shown encouraging results, indicating a similar safety profile to that of RTS,S/AS02A, higher humoral immunogenicity, a favorable Th1 cell mediated immune profile and a trend towards higher vaccine efficacy. In light of those promising results, a study comparing the safety of RTS,S/AS02A with RTS,S/AS01B is underway in Kenyan adults (Malaria-044; BB-IND11220). It will also examine immunogenicity and efficacy against *P. falciparum* infection versus control.

RTS,S/AS01E (0.5 mL dose) is composed of the same active constituents in the same quantities as in a 0.25 mL dose of RTS,S/AS01B. It therefore mirrors the relationship between RTS,S/AS02A used in studies in adults and RTS,S/AS02D developed for immunization of children and infants. RTS,S/AS01E was first administered to children 18 months to 4 years of age participating in the Malaria-046 trial in Gabon. A table detailing the various vaccine formulations that have been investigated in humans (or are planned for human trials) is given below.

	**Freeze-dried fraction**	**Liquid fraction**				
**Formulation**	**RTS,S (µg)**		**MPL (µg)**	**QS21 (µg)**	**Dose Volume**	
RTS,S/AS02A (0.5 mL dose)	50	Oil-in-water emulsion	50	50	0.5 mL	Efficacy against infection, Gambian adults (Malaria-005)
RTS,S/AS02A (0.25 mL dose)	25	Oil-in-water emulsion	25	25	0.25 mL	Efficacy against clinical disease, Mozambican children (Malaria-026)
RTS,S/AS02D	25	Oil-in-water emulsion	25	25	0.5 mL	Pediatric formulation (Malaria-034, -038, -040)
RTS,S/AS01B	50	Liposomes	50	50	0.5 mL	Efficacy in challenge model, adults (Malaria-027), endemic countries (Malaria-044)
RTS,S/AS01E	25	Liposomes	25	25	0.5 mL	Proposed pediatric formulation (Malaria-046)

The vaccine will be further tested in younger children in a safety and immunogenicity trial in 5 to 17 month old children in Ghana (Malaria-047). The DSMB will review unblinded safety data from that trial to authorize progression to the proposed trial.

In this study, the efficacy of a three dose regimen of RTS,S/AS01E against clinical malaria disease due to infection with *P. falciparum* will be determined. Safety, reactogenicity and immunogenicity will be explored as secondary and tertiary objectives.

This product development plan is conducted under a partnership agreement with the Malaria Vaccine Initiative at PATH (MVI) and is guided by a joint MVI/GSK Steering Committee.

This study is overseen by a formally constituted DSMB operating under a charter.

63f195821c4e0e87c982cb189f3de2c4

Objectives **Primary**

NOTE: *In the event that malaria rates are lower in the year of the study the cross sectional survey, unblinding and analysis scheduled at 4½ months post Dose 3 will be delayed until approximately 245 children have experienced an episode.*

- For RTS,S/AS01E when administered as 3 doses intramuscularly on a 0, 1, 2-month schedule to children aged 5 to 17 months living in malaria-endemic areas:
  - to assess efficacy against clinical malaria disease meeting the primary case definition (detected on active and passive case detection) with *P. falciparum* over a period of 4½ months (starting two weeks post Dose 3).

Secondary

NOTE: *In the event that malaria rates are lower in the year of the study the cross sectional survey, unblinding and analysis scheduled at 4½ months post Dose 3 will be delayed until approximately 245 children have experienced an episode.*

- For RTS,S/AS01E when administered as three doses intramuscularly on a 0, 1, 2-month schedule to children aged 5 to 17 months living in malaria-endemic areas:
  - to assess efficacy against clinical malaria disease meeting the secondary case definition (detected on active and passive case detection) with *P. falciparum* over a period of 4½ months (starting two weeks post Dose 3).
  - to assess safety and reactogenicity until 4½ months post Dose 3.
  - to assess the antibody responses to the hepatitis B surface (HBs) antigen up to 1 month post Dose 3.
  - to assess the antibody responses to the circumsporozoite (CS) antigen up to 4½ months post Dose 3.
  - to assess the T-cell immune response to CS antigen up to 1 month post Dose 3 in an Intracellular Cytokine Assay.
  - to assess the effect on hemoglobin, parasite prevalence and parasite density.

Tertiary

- For RTS,S/AS01E when administered as three doses intramuscularly on a 0, 1, 2-month schedule to children aged 5 to 17 months living in malaria-endemic areas:

63f195821c4e0e87c982cb189f3de2c4

- to assess efficacy against clinical malaria disease (detected on active and passive case detection) with *P. falciparum* over a period of 12 months post Dose 3.
- to assess safety between 4½ months post Dose 3 and 12 months post Dose 3.
- ***To assess safety until October 2008 in Kilifi and September 2008 in Korogwe.***

Amended 17 April 2008

- ***To assess efficacy against clinical malaria (detected on active and passive case detection) with* P. falciparum *from Dose 3 until October 2008 in Kilifi and September 2008 in Korogwe.***

Amended 17 April 2008

- to assess antibody responses to the circumsporozoite (CS) antigen at 12 months post Dose 3.
- to assess antibody responses to the hepatitis B surface (HBs) antigen at 12 months post Dose 3.
- ***to assess antibody responses to the circumpsorozoite (CS) antigen at a cross-sectional survey occurring in October 2008 in Kilifi and September 2008 in Korogwe.***

Amended 17 April 2008

- to assess the T-cell immune response to CS antigen up to 12 months post Dose 3 in an Intracellular Cytokine Assay.

Exploratory

- For RTS,S/AS01E when administered as three doses intramuscularly on a 0, 1, 2-month schedule to children aged 5 to 17 months living in malaria-endemic areas:
  - to evaluate the cellular immune responses to CS antigen up to 12 months post Dose 3 using cytokine elispot assay.
  - to evaluate the regulatory T-cell immune response to CS and blood stage malaria antigens at 4½ months post Dose 3.
  - to evaluate the humoral immune response to malaria blood stage antigens up to 12 months post Dose 3.

Study design

- Experimental design: Phase IIb, two-center, double blind (observer blind, participant blind), randomized (1:1 ratio), controlled trial with two groups.
- Healthy male and female children aged 5 to 17 months will be screened; those determined to be eligible, based on the inclusion and exclusion criteria, will be enrolled in the study.

- Hepatitis B immunization status will be recorded on the CRF at study entry.
- All EPI vaccines given throughout the trial will be recorded in the CRF.
- Route of administration: all vaccines will be administered by the intramuscular route to the left deltoid.
- Each child will be observed for at least 60 minutes after vaccination to evaluate and treat any acute adverse events (AEs).
- There will be a 7-day follow-up period for solicited AEs post-vaccination: Day 0 evaluation will be carried out by the study physician at the study center. Subsequently, trained field workers will visit the children to solicit adverse events (AEs) on days 1 to 6 after each vaccination.
- There will be a 30-day (day of vaccination and 29 subsequent days) follow-up after each vaccine dose for reporting unsolicited symptoms.
- Serious adverse events (SAEs) will be recorded throughout the study period. Prior to vaccination, any SAEs due directly to study procedures will be captured. All SAEs will be captured beginning with the administration of the first dose and ending 12 months after Dose 3 of study vaccines.
- All children will be followed by Active Case Detection (ACD) and Passive Case Detection (PCD) for a period extending to one year post Dose 3***, and then, under a separate consent, until October 2008 in Kilifi and September 2008 in Korogwe***.

  Amended 17 April 2008
- Two cross sectional surveys for efficacy will be performed at 4½ and 12 months post Dose 3 to determine parasite prevalence, parasite density and hemoglobin***, and under a separate consent, a third cross-sectional survey for efficacy will be performed in October 2008 in Kilifi and September 2008 in Korogwe to determine parasite prevalence, parasite density and hemoglobin.***

  Amended 17 April 2008
- In the event that malaria rates are lower in the year of the study the cross sectional survey, unblinding and analysis scheduled at 4½ months post Dose 3 will be delayed until approximately 245 children have experienced an episode.
- Anti-CS antibody titers will be determined at baseline, 1, 4½ and 12 months post Dose 3 ***and under a separate consent, at a cross-sectional survey occurring in October 2008 in Kilifi and September 2008 in Korogwe*** in all study participants.

  Amended 17 April 2008

- Anti-HBs antibody titers will be determined at baseline, 1 and 12 months post Dose 3 in all study participants.
- CS-specific IFN-γ ELISPOT responses will be evaluated at baseline, 1 and 12 months post Dose 3 in study participants at KEMRI, Kenya.
- Regulatory T-cell immune response will be determined at 4½ months post Dose 3 in all study participants.
- Anti-malaria blood stage humoral immunity will be determined at baseline, 1 and 12 months post Dose 3 in all study participants.
- Timing of final analysis is projected for 4½ months post Dose 3. The study will continue in a single-blind manner and be reported as an annex at the end of the study.
- The final analysis is projected to occur after the completion of 4 months of surveillance, at which time it is assumed that 36% of children in the control group will have been affected by a malaria episode. Assuming a true vaccine efficacy of 30%, 400 evaluable subjects per group will have 90% power to detect a difference between the groups using a two sided Fischer exact test with alpha level of 5%.
- The vaccine research teams will ensure that insecticide treated bed net use is optimized in the study population.
- Data collection: conventional Case Report Form (CRF).
- Treatment Groups and vaccination schedule:

Vaccine	Regimen	Number of infants to be enrolled	Estimated number evaluable
RTS,S/AS01E	0, 1, 2-month	445*	400
Rabies vaccine	0, 1, 2-month	445*	400

*approximate figure

Number of subjects

890 subjects to be enrolled. It is expected that approximately 800 subjects will be evaluable at study end.

Primary malaria case definition

- *P falciparum* infection > 2 500 parasites per µL in a child with a documented fever ≥ 37.5°C (axillary) detected by ACD or PCD.

Secondary malaria case definition

- *P falciparum* infection > 0 parasites per µL in a child with a documented fever ≥ 37.5°C (axillary) detected by ACD or PCD.

Primary endpoint

NOTE: *In the event that malaria rates are lower in the year of the study the cross sectional survey, unblinding and analysis scheduled at 4½ months post Dose 3 will be delayed until approximately 245 children have experienced an episode.*

- Time to first case of malaria meeting the primary case definition over a period starting 14 days after Dose 3 and extending for 4 months.

Secondary endpoints

Efficacy

- Time to first case of malaria meeting the secondary case definition over a period starting 14 days after Dose 3 and extending for 4 months.
- Multiple events of malaria meeting the primary case definition over a period starting 14 days after Dose 3 and extending for 4 months.
- Multiple events of malaria meeting the secondary case definition over a period starting 14 days after Dose 3 and extending for 4 months.
- Parasite prevalence and density at 4½ months post Dose 3.
- Hemoglobin at 4½ months post Dose 3.

Safety

- Occurrence of solicited symptoms and local reactions over a 7-day follow-up period (day of vaccination and 6 subsequent days) after each vaccination.
- Occurrence of unsolicited symptoms over a 30-day follow-up period (day of vaccination and 29 subsequent days) after each vaccination.
- Occurrence of serious adverse events from the time of first vaccination (Month 0) until 4½ months post Dose 3 (Month 6½).
- Occurrence of parameters of hematological monitoring outside acceptable ranges.

Immunogenicity

- Anti-CS antibody titers prior to vaccination, 1 month post Dose 3 and 4½ months post Dose 3.
- Anti-HBs antibody titers prior to vaccination and 1 month post Dose 3.
- Frequency of CD4+ and CD8+ CS-specific T-cells expressing at least two of the following: IL-2, CD40L, TNF-α and IFN-γ prior to vaccination and 1 month post Dose 3.

Tertiary endpoints

Efficacy

- Time to first case of malaria meeting the primary case definition over a period starting 14 days after Dose 3 and extending for 12 months ***and a period starting 14 days after Dose 3 and extending until October 2008 in Kilifi and September 2008 in Korogwe***.
  Amended 17 April 2008

- Time to first case of malaria meeting the secondary case definition over a period starting 14 days after Dose 3 and extending for 12 months ***and a period starting 14 days after Dose 3 and extending until October 2008 in Kilifi and September 2008 in Korogwe***.
Amended 17 April 2008
- Multiple events of malaria meeting the primary case definition over a period starting 14 days after Dose 3 and extending for 12 months ***and a period starting 14 days after Dose 3 and extending until October 2008 in Kilifi and September 2008 in Korogwe***.
Amended 17 April 2008
- Multiple events of malaria meeting the secondary case definition over a period starting 14 days after Dose 3 and extending for 12 months ***and a period starting 14 days after Dose 3 and extending until October 2008 in Kilifi and September 2008 in Korogwe***.
Amended 17 April 2008
- Parasite prevalence and density at 12 months post Dose 3 ***and in October 2008 in Kilifi and in September 2008 in Korogwe***.
Amended 17 April 2008
- Hemoglobin at 12 months post Dose 3 ***and in October 2008 in Kilifi and in September 2008 in Korogwe***.
Amended 17 April 2008

Safety

- Occurrence of SAEs throughout the entire study period.

Immunogenicity

- Anti-CS antibody titers at 12 months post Dose 3 (Month 14) ***and in October 2008 in Kilifi and in September 2008 in Korogwe***.
Amended 17 April 2008
- Anti-HBs antibody titers at 12 months post Dose 3.
- Frequency of CD4+ and CD8+ CS-specific T-cells expressing at least two of the following: IL-2, CD40L, TNF-α and IFN-γ prior to vaccination and 12 months post Dose 3.

Exploratory endpoints

Immunogenicity

- Numbers of IFN-γ producing cells upon CS stimulation in short and long term culture Elispot assays at baseline,one month and 12 months post Dose 3.
- Frequency of malaria antigen-specific effector cells and regulatory T-cells and levels of expression of effector and regulatory cytokines at 4½ months post Dose 3.

- Anti-malaria blood stage antigens antibody levels at baseline, one month, 4½ months and 12 months post Dose 3.

63f195821c4e0e87c982cb189f3de2c4

TABLE OF CONTENTS

PAGE

SYNOPSIS 19

LIST OF TABLES 32

LIST OF FIGURES 33

LIST OF APPENDICES 34

LIST OF ABBREVIATIONS 35

GLOSSARY OF TERMS 38

1. INTRODUCTION 42
1.1. Malaria 42
1.2. Hepatitis 42
1.3. RTS,S candidate vaccine 43
1.3.1. AS01 adjuvant system 43
1.3.2. AS02 adjuvant system 44
1.4. The RTS,S/AS02 candidate malaria vaccine; key clinical efficacy, safety and immunogenicity data 44
1.5. The RTS,S/AS01B candidate malaria vaccine; preliminary clinical safety, efficacy and immunogenicity data 46
1.6. Rationale for the study design 47
1.6.1. Rationale for the use of Rabies vaccine as a control 48
1.6.2. Rationale for immunogenicity investigations 49
1.6.3. Rationale for parasite genotyping 50
1.6.4. Rationale for study extension 50

2. OBJECTIVES 51
2.1. Primary objective 51
2.2. Secondary objectives 51
2.3. Tertiary objectives 51
2.4. Exploratory objectives 52

3. STUDY DESIGN OVERVIEW 53

4. STUDY COHORT 54
4.1. JMP Tanzania 55
4.1.1. JMP: Study Area Geography, Population 55
4.1.2. JMP: Malaria Epidemiology 56
4.1.3. JMP: Provision of Health Care 56
4.1.4. JMP: HIV and HIV services 56
4.2. KEMRI, Kenya 57
4.2.1. KEMRI: Study Area Geography, Population 57
4.2.2. KEMRI: Malaria Epidemiology 57
4.2.3. KEMRI: Provision of Health Care 57
4.2.4. KEMRI: HIV and HIV services 58
4.3. Inclusion criteria 58

4.4. Exclusion criteria for enrolment 58
4.5. Elimination criteria during the study 60
4.6. Contraindications to subsequent vaccination 60
4.6.1. Indications for deferral of vaccination 60
4.6.2. Absolute contraindications to further vaccination 61

5. CONDUCT OF STUDY 61
5.1. Ethics and regulatory considerations 61
5.1.1. Institutional Review Board/Independent Ethics Committee (IRB/IEC) 61
5.1.2. Informed consent 63
5.1.3. Storage of study documentation at investigator's sites 65
5.1.4. Safety Monitoring Plan 65
5.1.4.1. Data Safety Monitoring Board (DSMB) 65
5.1.4.1.1. Data Reviewed by the DSMB 66
5.1.4.2. Local Safety Monitor (LSM) 66
5.1.4.2.1. Data Reviewed by the LSM 66
5.1.4.3. Process if the trial is suspended 66
5.1.5. Rabies vaccination 67
5.1.5.1. Exposure to rabies 67
5.2. Recruitment/Screening 67
5.2.1. Community information 67
5.2.2. Screening of volunteers 67
5.3. Vaccination process 68
5.4. Home follow-up visits for assessment of reactogenicity (7-day follow-up period) 69
5.5. Monitoring of hematological and biochemical laboratory parameters 70
5.6. Surveillance for SAEs (all subjects) 70
5.6.1. At health facilities 70
5.7. Provision of health care 70
5.7.1. Health care provision to study subjects at JMP 70
5.7.2. Health care provision to study subjects at KEMRI 71
5.7.3. Measles vaccination 72
5.8. Management and treatment of malaria in all subjects 72
5.8.1. Insecticide impregnated bednets 72
5.9. Active case detection (ACD) of Malaria 72
5.10. Passive case detection (PCD) of malaria 73
5.11. Cross sectional surveys 73
5.12. Subject identification 74
5.13. Outline of study procedures 75
5.14. Detailed description of study stages/visits 78
5.15. Sample handling and analysis 87
5.15.1. Treatment and storage of biological samples 87
5.15.2. Laboratory assays 87
5.15.3. Serology and cell-mediated immunity plan 88
5.16. Collection of covariates 90

6. INVESTIGATIONAL PRODUCTS AND ADMINISTRATION 90
6.1. Study vaccines 90
6.1.1. RTS,S/AS01E vaccine 90
6.1.1.1. RTS,S antigen presentation: 90
6.1.1.2. AS01E adjuvant: 91

6.1.2. Sanofi-Pasteur's Human Diploid Cell Rabies Vaccine 91
6.2. Dosage and administration 91
6.2.1. RTS,S/AS01E 91
6.2.2. Sanofi-Pasteur's Human Diploid Cell Rabies Vaccine 92
6.3. Storage 92
6.4. Treatment allocation and randomization 92
6.4.1. Randomization of supplies 92
6.4.2. Randomization of subjects 92
6.5. Method of blinding and breaking the study blind 93
6.6. Replacement of unusable vaccine doses 93
6.7. Packaging 94
6.8. Vaccine accountability 94
6.9. Concomitant medication/treatment 94

7. HEALTH ECONOMICS 95

8. ADVERSE EVENTS AND SERIOUS ADVERSE EVENTS 95
8.1. Definition of an adverse event 95
8.2. Definition of a serious adverse event 96
8.3. Clinical laboratory parameters and other abnormal assessments qualifying as adverse events and serious adverse events 97
8.4. Time period, frequency, and method of detecting adverse events and serious adverse events 97
8.4.1. Solicited adverse events 99
8.5. Evaluating adverse events and serious adverse events 99
8.5.1. Assessment of intensity 99
8.5.2. Assessment of causality 101
8.6. Follow-up of adverse events and serious adverse events and assessment of outcome 103
8.7. Prompt reporting of serious adverse events to GSK Biologicals 104
8.7.1. Time frames for submitting serious adverse event reports to GSK Biologicals 104
8.7.2. Completion and transmission of serious adverse event reports to GSK Biologicals 104
8.8. Regulatory reporting requirements for serious adverse events 107
8.9. Post-study adverse events and serious adverse events 107
8.10. Pregnancy 107
8.11. Treatment of adverse events 107

9. SUBJECT COMPLETION AND WITHDRAWAL 108
9.1. Subject completion 108
9.2. Subject withdrawal 108
9.2.1. Subject withdrawal from the study 108
9.2.2. Subject withdrawal from investigational product 109

10. DATA EVALUATION: CRITERIA FOR EVALUATION OF OBJECTIVES 109
10.1. Primary endpoint 109
10.2. Secondary endpoints 109
10.3. Tertiary endpoints 110
10.4. Exploratory endpoint 111
10.5. Case Definitions for Malaria 111
10.6. Estimated sample size 112

10.6.1. Power Calculations 112
10.7. Study cohorts to be evaluated 112
10.7.1. Total Vaccinated cohort 112
10.7.2. According-to-Protocol (ATP) cohort for analysis of safety 112
10.7.3. According-to-Protocol (ATP) cohort for analysis of immunogenicity 113
10.7.4. According-to-Protocol (ATP) cohort for analysis of efficacy 113
10.8. Derived and transformed data 113
10.9. Statistical methods for Vaccine Efficacy endpoints 113
10.9.1. Time to event analyses 113
10.9.2. Total number of events analyses 114
10.9.3. Cross-sectional analyses 114
10.9.4. Calculation of time at risk 115
10.9.5. Covariates 115
10.10. Final analyses 116
10.10.1. Analysis of efficacy 116
10.10.2. Analysis of demographics/baseline characteristics 116
10.10.3. Analysis of immunogenicity 116
10.10.3.1. Anti-CS antibodies 117
10.10.3.2. Anti-HBs antibodies 117
10.10.3.3. Frequency of T-cells specific to CS 117
10.10.3.4. Exploratory immunogenicity endpoints 117
10.10.4. Analysis of safety 118
10.10.4.1. Secondary safety endpoints 118
10.11. Annex analysis at study conclusion 119
10.11.1. Analysis of efficacy 119
10.11.2. Analysis of safety 119
10.11.3. Analysis of immunogenicity 119
10.11.3.1. Anti-CS antibodies 119
10.11.3.2. Anti-HBs antibodies 119
10.11.3.3. Frequency of CD4+/CD8+ CS-specific T-cells 120
10.12. Planned interim analysis 120

11. ADMINISTRATIVE MATTERS 120

12. REFERENCES 120

63f195821c4e0e87c982cb189f3de2c4

LIST OF TABLES

PAGE

Table 1 Formulations of RTS,S 43

Table 2 Approximate number of doses of RTS,S/AS02A administered to date with number of recipients........ 44

Table 3 List of study procedures 75

Table 4 Intervals between study visits........ 78

Table 5 Laboratory immunological assays to be performed 87

Table 6 Summary of blood sampling time points/immunological assays and volumes to be collected........ 89

Table 7 Summary of time periods between which different classes of concomitant medication/treatment/vaccination must be recorded........ 95

Table 8 Intensity scales for solicited symptoms in infants/toddlers and children less than 6 years of age........ 100

Table 9 Acceptable/normal ranges for blood testing........ 101

Amended 17 April 2008

LIST OF FIGURES

PAGE

Figure 1 Map of Kenya and Tanzania indicating the principal towns in the study areas 55

Amended 17 April 2008

LIST OF APPENDICES

PAGE

Appendix A World Medical Association Declaration of Helsinki 126

Appendix B Administrative Matters 130

Appendix C Overview of the Recruitment Plan 136

Appendix D Handling of Biological Samples Collected by the Investigator 137

Appendix E Shipment of Biological Samples 141

Appendix F Laboratory Assays 143

Appendix G Vaccine supplies, packaging and accountability 145

Appendix H Composition of Data Safety Monitoring Board 148

Appendix I Amendments and Administrative Changes to the Protocol 149

List of Abbreviations

ACD	Active Case Detection
AE	Adverse event
ALT	Alanine aminotransferase
anti-CS	Antibody to the *P. falciparum* circumsporozoite (CS) repeat domain
anti-HBs	Antibody to the hepatitis B surface antigen
ATP	According to protocol
CI	Confidence interval
CMI	Cell-mediated immunity
CMP	Centre for Medical Parasitology, Denmark.
CRF	Case report form
CS	Circumsporozoite protein of *P. falciparum*
DTPw/Hib	Diphtheria, Tetanus, Pertussis (whole cell) and *Hemophilus influenzae* type B conjugate vaccine
DSMB	Data safety monitoring board
EIA	Enzyme immunosorbent assay
ELISA	Enzyme linked immunosorbent assay
EPI	Expanded program on immunization
FDA	Food and Drug Administration, United States
GCP	Good clinical practice
GMT	Geometric mean titer
GSK	GlaxoSmithKline
HAART	Highly Active Retroviral Therapy
HBsAg	Hepatitis B surface antigen
HBV	Hepatitis B virus

63f195821c4e0e87c982cb189f3de2c4

Hep B	Hepatitis B
HIV	Human immunodeficiency virus
IB	Investigator's brochure
ICF	Informed consent form
ICH	International Committee on Harmonization
IEC	Independent ethics committee
IFN-γ	Interferon gamma
IM	Intramuscular
IND	Investigational new drug
IRB	Institutional review board
IU	International unit
ITN	Insecticide-treated bednet
JMP	Joint Malaria Programme
KCMC	Kilimanjaro Christian Medical Centre, Tanzania
KEMRI	Kenya Medical Research Institute
kg	Kilogram
LEP	Low egg passage
LLN	Lower limit of normal
LSHTM	London School of Hygiene and Tropical Medicine, UK
LSM	Local safety monitor
MedDRA	Medical Dictionary for Regulatory Activities
mg	Milligram
mL	Milliliter
MPL®	3-deacylated monophosphoryl lipid A
MVI	Malaria Vaccine Initiative

63f195821c4e0e87c982cb189f3de2c4

NIMR	National Institute of Medical Research Tanzania
P. falciparum	*Plasmodium falciparum*
PATH	Program for Appropriate Technology in Health
PCD	Passive Case Detection
PFS	Pre-filled syringe
PI	Principal Investigator
PMCT	Prevention of Mother-to-Child Transmission
PPP	Pre-patent period
QS 21	'*Quillaja saponaria* 21': a triterpene glycoside purified from the bark of the soap bark tree, *Quillaja saponaria*
RAP	Report and Analysis Plan
RTS	Hybrid protein comprising HBs (hepatitis B surface antigen) and CSP portions
RTS,S	Particulate antigen, containing both RTS and HBs proteins
SAE	Serious adverse event
SOP	Standard operating procedure
SP	sulfadoxine-pyrimethamine
ULN	Upper limit of normal
VCT	Voluntary Counseling and Testing
VE	Vaccine efficacy
WRAIR	Walter Reed Army Institute of Research

Glossary of Terms

Adverse event:	Any untoward medical occurrence in a patient or clinical investigation subject, temporally associated with the use of a medicinal product, whether or not considered related to the medicinal product.<br><br>An AE can therefore be any unfavorable and unintended sign (including an abnormal laboratory finding), symptom, or disease (new or exacerbated) temporally associated with the use of a medicinal product. For marketed medicinal products, this also includes failure to produce expected benefits (i.e. lack of efficacy), abuse or misuse.
Severe Adverse Event	A serious adverse event (SAE) is any untoward medical occurrence that:<br><br>a. results in death;<br>b. is life-threatening;<br>c. requires hospitalization or prolongation of existing hospitalization;<br>d. results in disability/incapacity;<br>e. medical or scientific judgment should be exercised in deciding whether reporting is appropriate in other situations, such as important medical events that may not be immediately life-threatening or result in death or hospitalization but may jeopardize the subject or may require medical or surgical intervention to prevent one of the other outcomes listed in the above definition. These should also be considered serious. Examples of such events are invasive or malignant cancers, intensive treatment in an emergency room or at home for allergic bronchospasm, blood dyscrasias or convulsions that do not result in hospitalization.<br><br>A full definition of the events that constitute SAEs can be found in Section 8.2).

Blinding:	A procedure in which one or more parties to the trial are kept unaware of the treatment assignment in order to reduce the risk of biased study outcomes. In a single-blind trial, the investigator and/or his staff are aware of the treatment assignment but the subject is not. In an observer-blind study, the subject and the study personnel involved in the clinical evaluation of the subjects are blinded while other study personnel may be aware of the treatment allocation. When the investigator and sponsor staff who are involved in the treatment or clinical evaluation of the subjects and review/analysis of data are also unaware of the treatment assignments, the study is double blind.
Central Study Coordinator:	An individual assigned by and centrally located at GSK Biologicals at Rixensart who is responsible for assuring proper conduct of a clinical study.
Data Safety Monitoring Board (DSMB):	The DSMB is an independent committee appointed to oversee ethical and safety aspects of the conduct of the study. See Section 5.1.4.1 for a full overview of the role and structure of the DSMB.
Eligible:	Qualified for enrollment into the study based upon strict adherence to inclusion/exclusion criteria.
eTrack	GSK's clinical trials tracking tool.
Evaluable:	Meeting all eligibility criteria, complying with the procedures defined in the protocol, and, therefore, included in the according-to-protocol (ATP) analysis (see Sections 4.3, 4.4, and 10.7.2 for details on criteria for evaluability).
Investigational product:	A pharmaceutical form of an active ingredient or placebo being tested or used as a reference in a clinical trial, including a product with a marketing authorization when used in a way different from the approved form, or when used for an unapproved indication, or when used to gain further information about an approved use. The investigational products for this study are RTS,S/AS01E and ***Sanofi-Pasteur's Human Diploid Cell Rabies Vaccine***.

Amended 20 December 2006

Local Safety Monitor (LSM):	The overall role of the Local Safety Monitor, an experienced physician based in-country, will be to support the study investigators and to act as a link between the investigators and the Data Safety Monitoring Board (DSMB) (see Section 5.1.4.2 for further details on the LSM).
Medical Monitor:	An individual medically qualified to assume the responsibilities of the sponsor (GSK Biologicals) especially in regards to the ethics, clinical safety of a study and the assessment of adverse events.
Pre-patent Period (PPP)	The time in days between experimental sporozoite challenge and first detection of parasitemia by peripheral blood thick smear.
Protocol amendment:	ICH defines a protocol amendment as: "A written description of a change(s) to or formal clarification of a protocol". GSK Biologicals further details this to include a change to an approved protocol that affects the safety of subjects, scope of the investigation, study design, or scientific integrity of the study.
Protocol administrative change:	A protocol administrative change addresses changes to only logistical or administrative aspects of the study.<br><br>N.B. Any change that falls under the definition of a protocol amendment (e.g. a change that affects the safety of subjects, scope of the investigation, study design, or scientific integrity of the study) MUST be prepared as an amendment to the protocol.
Randomization:	Process of random attribution of treatment to subjects in order to reduce bias of selection.
Solicited adverse event:	Adverse events (AEs) to be recorded as endpoints in the clinical study. The presence/occurrence/intensity of these events is actively solicited from the subject or an observer during a specified post-vaccination follow-up period.
Study Monitor:	An individual assigned by the sponsor who is responsible for assuring proper conduct of a clinical study.
Subject:	Term used throughout the protocol to denote an individual that has been contacted in order to participate in the clinical study, either as a recipient of the investigational product(s) or as a control.
Treatment:	Term used throughout the clinical study to denote a set of investigational products or marketed products intended to be administered to a subject, identified by a unique number, according to the study randomization or treatment allocation.

Treatment number:	A unique number identifying a treatment to a subject, according to the study randomization or treatment allocation.
Unsolicited adverse event:	Any adverse event (AE) reported in addition to those solicited during the clinical study. Also any "solicited" symptom with onset outside the specified period of follow-up for solicited symptoms will be reported as an unsolicited adverse event.

1. INTRODUCTION

1.1. Malaria

Four species of the *Plasmodium* protozoan parasite are the etiologic agents of malaria in humans (*P. falciparum*, *P. vivax*, *P. ovale* and *P. malariae*). Of these four parasites, *P. falciparum* is the major cause of severe morbidity and mortality.

There can be no doubt of the importance of *P. falciparum* malaria as a major cause of human suffering and economic drain across sub-Saharan Africa [Breman 2001a; Gallup 2001]. In this region, it causes the deaths of between 0.5 and 2.0 million children every year and is a common reason for admission to hospital, leading each year to about 300 million clinical episodes in children under five years [Breman 2001a].

The incidence of malaria in much of Africa is increasing for a variety of reasons: changes in agricultural practices, armed conflicts, migration of refugees, increasing drug resistance to conventional anti-malarial drugs, and insecticide resistance of the anopheline mosquito vectors. It is estimated that without effective control the number of cases of clinical malaria will more than double over the next 20 years. The burden of malaria at the country level correlates closely with the rate of economic development even after adjustment for confounding factors, indicating that malaria is an important constraint on economic progress [Breman 2001b].

Clinical manifestations of *P. falciparum* disease appear as a result of the parasite infection of the red blood cell (RBC). Initial symptoms may include fever, chills, headache, joint and muscle pain, sweating, and vomiting. Acute complications may result from hemolysis leading to anemia and the propensity of infected RBCs to become adhesive and to be sequestered in capillaries thus causing local inflammatory reactions and damage to vital organs, leading to cerebral, hepatic, renal or pulmonary malaria. In *P. falciparum* malaria, an untreated acute attack can progress very rapidly and death may occur within a short timeframe.

Efforts to develop vaccines that target each stage of the parasite life cycle, to identify protective antigens and to understand the nature of the protective immune responses have been ongoing for the past three decades. The approach of GlaxoSmithKline (GSK) Biologicals has been to focus on vaccines that target the free sporozoite and intra-hepatic stages of the parasites (i.e. the pre-erythrocytic stages).

1.2. Hepatitis

Hepatitis B is an infection of the liver due to hepatitis B virus (HBV); it is an important public health problem across the developing world. World-wide approximately 350 million people carry HBV and about 1 million chronic carriers die annually [Vryheid 2001]. The likelihood of the infection becoming chronic is dependent upon the age at infection: 90% if infected in infancy, 30% to 50% if infected between the ages of 1 to 4 years, and low in adulthood. For those that become chronically infected during childhood the risk of death from HBV-related liver cancer or cirrhosis in adult life is approximately 25% [World Health Organization 2003].

1.3. RTS,S candidate vaccine

The candidate vaccine consists of sequences of the circumsporozoite (CS) protein and the hepatitis B surface antigen (HBsAg) adjuvanted with AS02 (proprietary oil-in-water emulsion formulated with MPL® and Stimulon® QS21 immunostimulants) or AS01 (liposome formulation with MPL and QS21 immunostimulants).

The HBsAg contained in the RTS,S candidate malaria vaccine is encoded by the hepatitis B virus S protein gene that is identical to the gene used to express HBsAg in GSK Biologicals' Engerix-B® vaccine against hepatitis B. As a result, vaccines containing RTS,S in combination with AS02 and AS01 adjuvants also provide protection against hepatitis B.

In parallel to the continued development of RTS,S/AS02 in children in endemic countries, GSK Biologicals and the Walter Reed Army Institute of Research have continued to pursue strategies to improve the vaccine efficacy (VE) and duration of efficacy. One such strategy is the formulation of the RTS,S antigen with the AS01 adjuvant system. Both the AS02 and AS01 adjuvant systems have a number of similar key components (Table 1).

Table 1 Formulations of RTS,S

	Freeze-dried fraction	Liquid fraction				
Formulation	RTS,S (µg)		MPL (µg)	QS21 (µg)	Dose Volume	
RTS,S/AS02A (0.5 mL dose)	50	Oil-in-water emulsion	50	50	0.5 mL	Efficacy demonstrated in adults in The Gambia (Malaria-005[a])
RTS,S/AS02A (0.25 mL dose)	25	Oil-in-water emulsion	25	25	0.25 mL	Efficacy against clinical disease in children in Mozambique (Malaria-026[b])
RTS,S/AS02D	25	Oil-in-water emulsion	25	25	0.5 mL	Pediatric formulation (Malaria-034[c], -038[d], -040[e])
RTS,S/AS01B	50	Liposomes	50	50	0.5 mL	Efficacy in challenge model, adults (Malaria-027[f]), endemic countries (Malaria-044[g])
RTS,S/AS01E	25	Liposomes	25	25	0.5 mL	Proposed pediatric formulation (Malaria-046[h])

a GSK data on file, Malaria-005, Clinical Study Report, 2006; Bojang 2001; Alloueche 2003; Pinder 2004; Reece 2004
b GSK data on file, Malaria-026, Clinical Study Report, 2004; Alonso 2004; Alonso 2005
c GSK data on file, Malaria-034, Clinical Study Report, 2005
d GSK data on file, Malaria-038, Clinical Study Protocol, 2005
e GSK data on file, Malaria-040, Clinical Study Protocol, 2005
f GSK data on file, Malaria-027, Clinical Study Protocol, 2003
g GSK data on file, Malaria-044, Clinical Study Protocol, 2005
h GSK data on file, Malaria-046, Clinical Study Protocol, 2006

1.3.1. AS01 adjuvant system

The GSK proprietary adjuvant system 1 (AS01) is composed of the immunostimulants Stimulon® QS21 (a triterpene glycoside purified from the bark of *Quillaja saponaria*) and MPL® with liposomes. RTS,S/AS01E is composed of the same active constituents in the same quantities as in a 0.25 mL dose of RTS,S/AS01B, but is formulated to supply a 0.5 mL dose volume.

1.3.2. AS02 adjuvant system

The GSK proprietary adjuvant system 2 (AS02) is composed of the immunostimulants Stimulon® QS21 (a triterpene glycoside purified from the bark of *Quillaja saponaria*) and MPL® with proprietary oil-in-water emulsion. RTS,S/AS02D is composed of the same active constituents in the same quantities as in a 0.25 mL dose of RTS,S/AS02A, but is formulated to supply a 0.5 mL dose volume.

1.4. The RTS,S/AS02 candidate malaria vaccine; key clinical efficacy, safety and immunogenicity data

In the clinical trial setting to date, the RTS,S/AS02A candidate vaccine has been administered to both malaria-naïve adult subjects (subjects who live in countries where there is no naturally occurring malaria transmission) and naturally exposed adults and children (subjects who live in countries where malaria transmission occurs naturally). The numbers of doses of RTS,S-containing vaccines and the number of recipients is tabulated in Table 2. A comprehensive summary of the results of reported trials to date can be found in the Malaria Vaccine Investigator Brochure 2005 [GSK data on file].

Table 2 Approximate number of doses of RTS,S/AS02A administered to date with number of recipients

Subject population	Recipients	Doses administered
Malaria-naïve adults [a]	247	596
Naturally-exposed adults [b]	266	842
Naturally-exposed children [b]	1292	3746

a subjects who took part in studies conducted in the USA and Belgium, where there is no naturally occurring transmission of malaria
b subjects who took part in studies conducted in malaria-endemic countries in Africa.

Early clinical development of the RTS,S malaria candidate vaccine was initiated in studies in malaria-naïve adults in collaboration with the WRAIR in which confirmation of the efficacy, safety and immunogenicity of the RTS,S/AS02A vaccine formulation was demonstrated [Stoute 1997; Kester 2001]. Two doses of RTS,S/AS02A (0.5 mL) provided protection to 37.8% healthy non-immune volunteers against homologous sporozoite challenge (pooled results for WRMAL-004 [Report, GSK data on file] & -005 [Report, GSK data on file]); 3 doses demonstrated protection of 43.2% of subjects (pooled results for WRMAL-004 & Malaria-012 [Report, GSK data on file]). In subjects not protected, the prepatent period (PPP) was significantly prolonged in the RTS,S/AS02A group compared to control (WRMAL-004, WRMAL-005 & Malaria-012). Protective efficacy was low following re-challenge six months after Dose 3 of RTS,S/AS02A, but a statistically significant difference between PPP for vaccinees compared to infectivity control was observed (WRMAL-004, WRMAL-005 & Malaria-012). A strong humoral immune response to the RTS,S/AS02A vaccine in terms of anti-CS and anti-HBs antibodies was demonstrated in all the adult studies in malaria-naïve individuals. Evaluations of the CMI response showed consistently that administration of RTS,S/AS02A induced strong cellular Th1 T-cell responses, specific to the vaccine antigen [Lalvani, 1999; Epstein, 2004; Sun, 2003].

The RTS,S/AS02A vaccine progressed to evaluation in subjects under conditions of natural transmission. In adult males from The Gambia, VE against infection adjusted for covariates was 71% (95% CI: 46 to 85; p< 0.001) during the first 2 months and 34% (95% CI: 8.0 to 53, p=0.014) for the entire 15 week surveillance period (Malaria-005 [Report, GSK data on file], [Bojang 2001]). VE, adjusted for covariates, following a booster dose given during a second year malaria season was 47% (95% CI: 3 to 71; p=0.039). Protection was not limited to the NF54 parasite genotype from which the vaccine was derived [Alloueche, 2003]. Safety surveillance over approximately 5 years showed no safety signal (Malaria-016, -017 & -018 [Report, GSK data on file]). A strong humoral immune response to the RTS,S/AS02A vaccine in terms of anti-CS and anti-HBs antibodies was demonstrated. Overall, the kinetics of the humoral immune response induced by vaccination with RTS,S/AS02A were similar in malaria-naïve and experienced populations, while the absolute GMT values appeared to be higher in malaria-naïve volunteers. The vaccine induced and boosted Th1-like cellular immunity to several T-cell epitopes in a population naturally exposed to malaria [Pinder 2004; Reece 2004].

The RTS,S/AS02A candidate vaccine progressed to clinical evaluation in children. Two age de-escalation and dose comparison trials (which compared doses with 10 µg, 25 µg and 50 µg of antigen corresponding to volume fractions of RTS,S/AS02A: 0.1 mL, 0.25 mL and 0.5 mL respectively) enrolled a total of 225 children aged 1 to 11 years from The Gambia (Malaria-015 [Report, GSK data on file] & -020 [Report, GSK data on file], [Bojang 2001]). From these trials, the 0.25 mL dose was selected due to equivalent immunogenicity and slightly less reactogenicity in the 0.25 mL group compared to the 0.5 mL group; immunogenicity was consistently lowest in the 0.1 mL group. The safety and immunogenicity of the RTS,S/AS02A 0.25 mL dose was further confirmed in another study, Malaria-025 [Report, GSK data on file], conducted in 1 to 4 year old children in Mozambique.

Subsequently, a large safety, immunogenicity and efficacy trial in children aged 1 to 4 years from an area of high transmission was conducted in Mozambique, enrolling a total of 2022 subjects (Malaria-026 [Report, GSK data on file]; [Alonso 2004]; [Alonso 2005]). In this study, 3 doses of RTS,S/AS02A (0.25 mL dose) were administered according to a 0, 1, 2-month schedule to approximately 1000 children. The primary endpoint of this trial investigated vaccine efficacy against the onset of clinical malaria disease. Up to six months post Dose 3, the VE determined as the time to the first clinical episode (after adjustment for covariates) was 29.9% (95% CI: 11.0 to 44.8; p=0.004). For the entire study period, up to 18 months post Dose 3, VE for the same endpoint was 32.8% (95% CI: 20.1 to 43.4; p=0.0001).

The trial also offered the opportunity to investigate VE against multiple attacks of malaria and severe malaria. Against multiple attacks, VE was 27.4% (95% CI: 6.2 to 43.8; p=0.014) up to six months post Dose 3. For the entire study period, VE was 32.4% (95% CI: 17.6 to 44.5; p=0.0001). Against severe malaria, VE was 57.7% (95% CI: 16.2 to 80.6; p=0.019) over six months post Dose 3: for the entire study period VE was 48.6% (95% CI 12.3 to 71.0; p=0.02).

During the single-blind phase (Months 8½ to 21) in both Cohort 1 and 2, the proportion of children experiencing a SAE was similar in the RTS,S/AS02A and control groups

(5.1% vs. 6.8%, respectively). However, in the overall surveillance period (Months 0 to 21), there was a tendency for the proportion of children experiencing a SAE to be lower in the RTS,S/AS02A group compared to the control groups in both Cohorts 1 and 2 (20.9% vs. 28.6%, respectively). The pattern of morbidity was similar to that previously observed at the study site and described in the region. At least 97% of recipients of RTS,S/AS02A were seropositive for anti-CS antibodies and at least 97% of recipients of RTS,S/AS02A were seroprotected for anti-HBs antibodies 18 months post Dose 3 [Alonso, 2005].

A 0.5 mL variant of the 0.25 mL dose of RTS,S/AS02A (RTS,S/AS02D) was developed for compatibility with standard auto-disable EPI syringe. RTS,S/AS02D, was shown in children aged 3 to 5 years in a malaria-endemic region of Mozambique to be safe and exhibit non-inferior immunogenicity to RTS,S/AS02A (0.25 mL dose) (Malaria-034 [Report, GSK data on file]); up to one year post Dose 3, SAEs had been reported in 10 subjects (six in the RTS,S/AS02D group, four in the RTS,S/AS02A group). Non-inferiority of the anti-CS and anti-HBs antibody responses induced by the RTS,S/AS02D formulation as compared to the RTS,S/AS02A (0.25 mL dose) formulation was demonstrated.

1.5. The RTS,S/AS01B candidate malaria vaccine; preliminary clinical safety, efficacy and immunogenicity data

By the time this study has started, a number of other studies of RTS,S/AS01B will have been studied in a number of other populations: malaria naive adults in the US (Malaria-027 [Report, GSK data on file]); adults in Kenya (Malaria-044 [Protocol, GSK data on file]); children aged 1 to 4 years in Gabon (Malaria-046 [GSK data on file]) and children aged 5 to 17 months in Ghana (Malaria-047 [Protocol, GSK data on file]).

To date, preliminary results of a challenge study conducted in healthy malaria-naïve adults have been encouraging (Malaria-027 [Report, GSK data on file]), indicating that RTS,S/AS01B may be more efficacious than RTS,S/AS02A. In this double-blind**,** randomized Phase I/IIa human challenge study, the safety, reactogenicity, immunogenicity and preliminary efficacy against infection after early sporozoite challenge and rechallenge, of RTS,S/AS01B and RTS,S/AS02A were assessed. Two sequential cohorts of approximately 52 subjects each were enrolled; each cohort was evenly divided into 2 groups receiving either the RTS,S/AS01B or RTS,S/AS02A vaccine (up to 48 infectivity controls for challenge and re-challenge phases were additionally enrolled). Subjects were vaccinated at 0, 1, 2-months followed by a challenge 14 to 30 days after Dose 3. Protected individuals were invited to be rechallenged approximately 6 months after Dose 3 to evaluate persistence of efficacy. To date, results for Cohort 1 are available [Report, GSK data on file].

In Cohort 1, following primary challenge, the point efficacy of VE was significantly greater in both vaccine groups compared to infectivity control. Although not statistically significant, the point estimate of vaccine efficacy was higher in the RTS,S/AS01B group compared to RTS,S/AS02A (58.8% [95% CI: 32.1, 81.6] vs. 37.5% [95% CI: 11.8, 59.4], p=0.2085). In non-protected subjects, the mean time to infection (pre-patent period; PPP)

following challenge was similar in both vaccine groups and longer than in the infectivity control.

Following re-challenge, 2/5 subjects (40.0%) in the RTS,S/AS01B group and 4/5 subjects (80.0%) in the RTS,S/AS02A group were infected; all infectivity control subjects became infected. There was a trend towards greater VE in the RTS,S/AS01B group compared to infectivity control (p=0.061) and VE was greater when compared to RTS,S/AS02A (60.0% [95% CI: 3.2, 95.1] vs. 20.0% [95% CI: -60.2, 71.6], respectively). The PPP in non-protected subjects was longer in the RTS,S/AS01B group compared RTS,S/AS02A; PPP was longer in both vaccine groups compared to control. Anti-CS antibody responses were greater in recipients of RTS,S/AS01B than of RTS,S/AS02A; anti-CS CD4+ T-cell responses were stronger in the RTS,S/AS01B group compared to RTS,S/AS02A.

The reactogenicity and safety profile of RTS,S/AS01B was comparable to RTS,S/AS02A. Full details of the reactogenicity and safety can be found in the Malaria Vaccine Investigator Brochure 2005 [GSK data on file]. The overall incidence of AEs (both solicited and unsolicited) was comparable in the RTS,S/AS01B group (following 88.2% of doses) and the RTS,S/AS02A group (following 91.9% of doses). Grade 3 AEs (solicited and unsolicited) were reported less frequently in the RTS,S/AS01B group (following 17.6% doses) compared to the RTS,S/AS02A group (following 28.4% of doses). Only 10 solicited/unsolicited AEs (following < 0.5% of all doses administered) were reported during days 5 to 7 of the follow-up period following vaccination.

Pain was the most frequently reported solicited local AE in both the RTS,S/AS01B and RTS,S/AS02A groups (following 77.9% and 81.1% of doses, respectively), occurring with a similar frequency in each vaccine group. The incidence of Grade 3 pain was lower in the RTS,S/AS01B group compared to the RTS,S/AS02A group (following 2.9% and 14.9% of doses, respectively). Fatigue, headache and malaise were the most frequently reported solicited general AEs after vaccination in both groups. All Grade 3 solicited local events resolved within the 7 day follow-up period.

Few Grade 3 solicited general AEs were reported (following ≤ 4.4% doses). The frequency of unsolicited AEs considered to be related to vaccination was similar in the RTS,S/AS01B and RTS,S/AS02A groups (following 22.1% and 17.6% of doses, respectively).

Two SAEs were reported during the study (tendon rupture, cerebral infarction), both in the RTS,S/AS01B group, neither of which was considered to be related to study vaccine.

1.6. Rationale for the study design

The RTS,S/AS01E candidate malaria vaccine is being developed for the routine immunization of infants and children living in malaria-endemic areas as part of the Expanded Program of Immunization (EPI). The RTS,S/AS01E vaccine candidate consists of sequences of the circumsporozoite (CS) protein and hepatitis B surface antigen (HBsAg) with the proprietary adjuvant AS01E (proprietary liposomes, MPL® and Stimulon ® QS21 immunostimulants).

The first RTS,S-based malaria vaccine to be evaluated in children was RTS,S/AS02A (0.25 mL dose). The AS02A adjuvant consists of a proprietary oil-in-water emulsion and two immunostimulants, MPL® and Stimulon® QS21. The RTS,S/AS02A vaccine has demonstrated 30% efficacy against clinical malaria and 58% efficacy against severe malaria disease in children aged 1 to 4 years (Malaria-026). In addition, in that study the vaccine demonstrated a favorable safety profile and induction of protective anti-HBs antibody levels.

The RTS,S/AS01 vaccines have been developed in parallel with the RTS,S/AS02 vaccines with the aim of improving the immune response and increasing vaccine efficacy. A recent challenge study with RTS,S/AS01B in malaria-naïve adults (Malaria-027) has shown encouraging results, indicating a similar safety profile to that of RTS,S/AS02A, higher humoral immunogenicity, a favorable Th1 cell mediated immune profile and a trend towards higher vaccine efficacy. In light of those promising results, a study comparing the safety of RTS,S/AS02A with RTS,S/AS01B is underway in Kenyan adults (Malaria-044; BB-IND11220). It will also examine immunogenicity and efficacy against *P. falciparum* infection versus control.

RTS,S/AS01E (0.5 mL dose) is composed of the same active constituents in the same quantities as in a 0.25 mL dose of RTS,S/AS01B. It therefore mirrors the relationship between RTS,S/AS02A used in studies in adults and RTS,S/AS02D developed for immunization of children and infants. RTS,S/AS01E was first administered to children 18 months to 4 years of age participating in the Malaria-046 trial in Gabon.

The vaccine will be further tested in younger children in a safety and immunogenicity trial in 5 to 17 month old children in Ghana (Malaria-047). The DSMB will review unblinded safety data from that trial to authorize progression to the proposed trial.

In this study, the efficacy of a three dose regimen of RTS,S/AS01E against clinical malaria disease due to infection with *P. falciparum* will be determined. Safety, reactogenicity and immunogenicity will be explored as secondary and tertiary objectives.

1.6.1. Rationale for the use of Rabies vaccine as a control

Rabies vaccine has been chosen as the comparator because: 1) volunteers will benefit from receiving Rabies vaccine as rabid animals occur in the study area and 2) Rabies vaccine can be administered on a 0, 1, 2-month schedule.

Sanofi-Pasteur's Human Diploid Cell Rabies Vaccine will be used. According to product information distributed by Sanofi Pasteur Inc, PA, USA in December 2005,

> "High titer antibody responses of the Sanofi Pasteur SA Rabies Vaccine made in human diploid cells have been demonstrated in trials conducted in England [Aoki 1975], Germany [Cox, 1976; Kuwert, 1978], France [Ajjan, 1978] and Belgium [Costy-Berger, 1978]. Seroconversion was often obtained with only one dose. With two doses one month apart, 100% of the recipients developed specific antibody and the geometric mean titer of the group was approximately 10 international units. In the US, Sanofi Pasteur SA Rabies Vaccine resulted in geometric mean titers (GMT) of 12.9 IU/mL at Day 49 and 5.1 IU/mL at Day 90 when three doses were given

intramuscularly during the course of one month. The range of antibody responses was 2.8 to 55.0 IU/mL at Day 49 and 1.8 to 12.4 IU at Day 90 [Bernard 1982]. The definition of a minimally accepted antibody titer varies among laboratories and is influenced by the type of test conducted. CDC currently specifies a 1:5 titer (complete inhibition) by the rapid fluorescent focus inhibition test (RFFIT) as acceptable. The World Health Organization (WHO) specifies a titer of 0.5 IU".

Amended 20 December 2006

High antibody titers have also been demonstrated with off-label immunization with Rabies vaccines. Among participants in England, Germany, France and Belgium who received two vaccinations one month apart, nearly 100% of the participants developed specific antibody and the geometric mean titer for the group was 10 IU/mL [Ajjan, 1978; Costy-Berger, 1978; Cox, 1976; Kuwert, 1978]. The proposed vaccination schedule of 0, 1, 2-months is therefore expected to be highly successful in conferring protective immunity against rabies among the control participants. However all parents/guardians will be advised that if their child is bitten or scratched by a potentially rabid animal, they must seek medical attention immediately.

1.6.2. Rationale for immunogenicity investigations

Neutralizing-antibody and cell-mediated immunity (CMI) responses are thought to be important immune effector mechanisms for protecting people vaccinated with adjuvanted RTS,S-based vaccines. It is hypothesized that immune responses act to limit hepatocyte invasion, destroy infected hepatocytes and/or limit intracellular parasite development.

It is hypothesized that young children will be protected from severe forms of the disease by the pre-erythrocytic immune response induced by the RTS,S vaccines by limiting the number of parasites reaching the blood stage of infection. However the continued exposure will enable them to develop blood stage immunity. Therefore the development of blood-stage immunity will be investigated in this trial.

RTS,S/AS02A and RTS,S/AS01B have been shown to be powerful inducers of antigen-specific humoral and CMI anti-CS responses in preclinical and clinical studies [Malaria Investigator's Brochure 2005, GSK data on file]. Recent preliminary results of a challenge study conducted in healthy malaria-naïve adults (Malaria-027 [Report, GSK data on file]) show that RTS,S/AS01B induces more potent CMI responses characterized predominantly by CD4+ type 1 responses and higher anti-CS antibody levels as compared to RTS,S/AS02A. This may be associated with increased vaccine efficacy on the part of RTS,S/AS01B; immunological monitoring performed during this trial revealed an association between protection against *P. falciparum* and CS-specific humoral and cellular response (Dr Kent Kester, WRAIR, personal communication, November 2005; publication in preparation).

In this study, the quality of the anti-CS immune response induced by RTS,S/AS01E will be investigated: anti-CS effector T-cell-mediated immune responses will be investigated using short-term antigen whole blood stimulation and measurement of intracellular cytokines and cell activation markers expression by FACS (fluorescent-activated cell sorter). The induction of CS-specific short-term effector cells and central memory T-cells

will also be investigated using short and long-term cell culture in the presence of CS antigen in IFN-γ Elispot assays. Finally, the induction of CS-specific and blood stage malaria antigen-specific regulatory T-cells will be assessed by the measurement of expression of regulatory markers and cytokines.

In addition to the characterization of anti-CS immune responses, the influence of RTS,S/AS01E vaccination on the acquisition of immunity to blood stage malaria antigens will be studied. Titers of antibodies against a panel of blood stage antigens will be measured.

1.6.3. Rationale for parasite genotyping

The RTS protein is derived from a sporozoite surface antigen of the *P. falciparum* strain NF54. To date, there has been no evidence of parasite-specific immunity. In both the Malaria-005 efficacy study conducted in Gambian men and the Malaria-026 study in Mozambican children, protection was not limited to the NF54 parasite genotype [Bojang 2001; Enosse 2006]. Nevertheless, specimens appropriate for genotyping will be taken on every occasion on which a malaria blood film is taken to enable potential further studies of parasite genetics, should this be deemed necessary.

1.6.4. Rationale for study extension

Vaccinations began in March 2007. A series of logistic factors, including a change of supplier for the rabies control vaccine leading to interruption in delivery of the study vaccine, and lower than expected recruitment rates resulted in a variable delay of vacination, such that surveillance post Dose 3 began in May 2007 for some children, but in October 2007 for others. 12 months of surveillance post Dose 3 are planned according per protocol, so children will finish surveillance in the period June 2008-October 2008.

The high malaria transmission season is June-October in Kilifi and March-August in Korogwe. In this amendment dated April 2008, we propose to continue active and passive case detection in all children until the end of the transmission season. This will result in a variable length of follow up, but all children will end surveillance on the same date in each site (October 2008 in Kilifi, September 2008 in Korogwe). Surveillance is therefore extended through the transmission season for all subjects and is likely to produce an increase in power.

There will be two additional visits. The first visit (Clinic Visit 8) at the start will be to request informed consent. The second visit (Clinic Visit 9) at the close of study will allow to assess safety at the final time-point, and to obtain a cross-sectional bleed in order to study parasite prevalence at the end of the malaria transmission season. The duration of protection may be linked to the duration of immunogenicity, and so serum will be used from this final time-point to measure vaccine-induced antibodies to the circumsporozoite antigen (CS).

Amended 17 April 2008

2. OBJECTIVES

2.1. Primary objective

NOTE: *In the event that malaria rates are lower in the year of the study the cross sectional survey, unblinding and analysis scheduled at 4½ months post Dose 3 will be delayed until approximately 245 children have experienced an episode.*

- For RTS,S/AS01E when administered as 3 doses intramuscularly on a 0, 1, 2-month schedule to children aged 5 to 17 months living in malaria-endemic areas:
  - to assess efficacy against clinical malaria disease meeting the primary case definition (detected on active and passive case detection) with *P. falciparum* over a period of 4½ months (starting two weeks post Dose 3).

Refer to Section 10.1 for definition of the primary endpoint.

2.2. Secondary objectives

NOTE: *In the event that malaria rates are lower in the year of the study the cross sectional survey, unblinding and analysis scheduled at 4½ months post Dose 3 will be delayed until approximately 245 children have experienced an episode.*

- For RTS,S/AS01E when administered as three doses intramuscularly on a 0, 1, 2-month schedule to children aged 5 to 17 months living in malaria-endemic areas:
  - to assess efficacy against clinical malaria disease meeting the secondary case definition (detected on active and passive case detection) with *P. falciparum* over a period of 4½ months (starting two weeks post Dose 3).
  - to assess safety and reactogenicity until 4½ months post Dose 3.
  - to assess the antibody responses to the hepatitis B surface (HBs) antigen up to 1 month post Dose 3.
  - to assess the antibody responses to the circumsporozoite (CS) antigen up to 4½ months post Dose 3.
  - to assess the T-cell immune response to CS antigen up to 1 month post Dose 3 in an Intracellular Cytokine Assay.
  - to assess the effect on hemoglobin, parasite prevalence and parasite density

Refer to Section 10.2 for definitions of secondary endpoints.

2.3. Tertiary objectives

- For RTS,S/AS01E when administered as three doses intramuscularly on a 0, 1, 2-month schedule to children aged 5 to 17 months living in malaria-endemic areas:
  - to assess efficacy against clinical malaria disease (detected on active and passive case detection) with *P. falciparum* over a period of 12 months post

Dose 3.

- to assess safety between 4½ months post Dose 3 and 12 months post Dose 3.
- ***To assess safety until October 2008 in Kilifi and September 2008 in Korogwe.***

Amended 17 April 2008

- ***To assess efficacy against clinical malaria (detected on active and passive case detection) with* P. falciparum *from Dose 3 until October 2008 in Kilifi and September 2008 in Korogwe.***

Amended 17 April 2008

- to assess antibody responses to the circumsporozoite (CS) antigen at 12 months post Dose 3.
- to assess antibody responses to the hepatitis B surface (HBs) antigen at 12 months post Dose 3.
- ***to assess antibody responses to the circumpsorozoite (CS) antigen at a cross-sectional survey occurring in October 2008 in Kilifi and September 2008 in Korogwe.***

Amended 17 April 2008

- to assess the T-cell immune response to CS antigen up to 12 months post Dose 3 in an Intracellular Cytokine Assay.

Refer to Section 10.3 for definitions of tertiary endpoints.

2.4. Exploratory objectives

- For RTS,S/AS01E when administered as three doses intramuscularly on a 0, 1, 2-month schedule to children aged 5 to 17 months living in malaria-endemic areas:
  - to evaluate the cellular immune responses to CS antigen up to 12 months post Dose 3 using cytokine elispot assay
  - to evaluate the regulatory T-cell immune response to CS and blood stage malaria antigens at 4½ months post Dose 3.
  - to evaluate the humoral immune response to malaria blood stage antigens up to 12 months post Dose 3

Refer to Section 10.4 for definition of tertiary endpoint.

3. STUDY DESIGN OVERVIEW

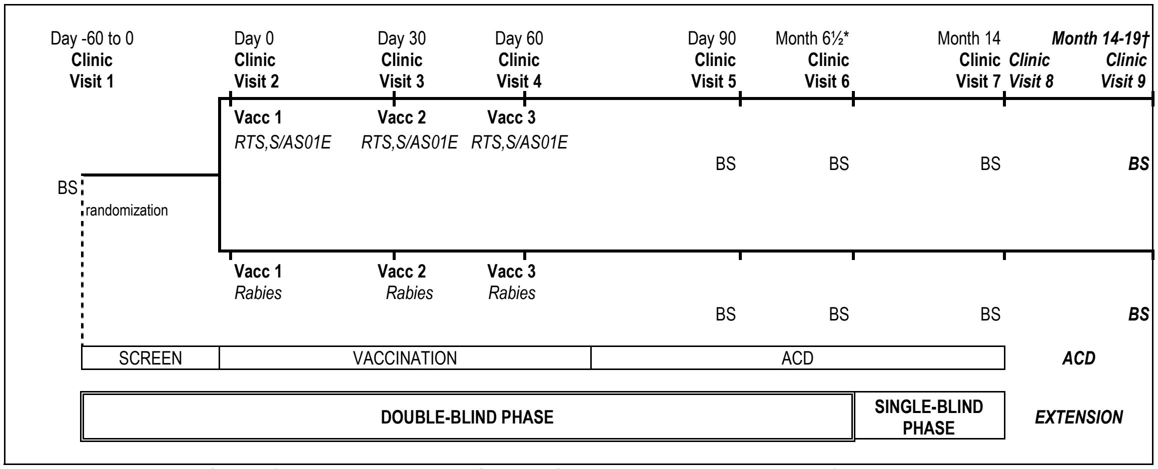

KEY: BS; Blood Sample. **Vacc;** Vaccination. **ACD;** Active Case Detection. ***Extension; extention of the single-blind phase***
Clinic Visit 7 and Clinic Visit 8 can take place on the same day. For subjects who do not present at Clinic Visit 7, an interval of 14 to 17 months post Clinic Visit 2 to Clinic Visit 8 is acceptable.
† Month 14-19 at Clinic Visit 9 corresponds to October 2008 in Kilifi and September 2008 in Korogwe
*In the event that malaria rates are lower in the year of the study, the cross sectional survey, unblinding and analysis will be delayed until sufficient cases have accumulated to have an adequately powered analysis.

Amended 17 April 2008

- Experimental design: Phase IIb, two-center, double blind (observer blind, participant blind), randomized (1:1 ratio), controlled trial with two groups.
- Healthy male and female children aged 5 to 17 months will be screened; those determined to be eligible, based on the inclusion and exclusion criteria, will be enrolled in the study.
- Hepatitis B immunization status will be recorded on the CRF at study entry.
- All EPI vaccines given throughout the trial will be recorded in the CRF.
- Route of administration: all vaccines will be administered by the intramuscular route to the left deltoid.
- Each child will be observed for at least 60 minutes after vaccination to evaluate and treat any acute adverse events (AEs).
- There will be a 7-day follow-up period for solicited AEs post-vaccination: Day 0 evaluation will be carried out by the study physician at the study center. Subsequently, trained field workers will visit the children to solicit adverse events (AEs) on days 1 to 6 after each vaccination.
- There will be a 30-day (day of vaccination and 29 subsequent days) follow-up after each vaccine dose for reporting unsolicited symptoms.
- Serious adverse events (SAEs) will be recorded throughout the study period. Prior to vaccination, any SAEs due directly to study procedures will be captured. All SAEs will be captured beginning with the administration of the first dose and ending 12 months after Dose 3 of study vaccines.

- All children will be followed by Active Case Detection (ACD) and Passive Case Detection (PCD) for a period extending to one year post Dose 3***, and then, under a separate consent, until October 2008 in Kilifi and September 2008 in Korogwe.***
Amended 17 April 2008
- Two cross sectional surveys for efficacy will be performed at 4½ and 12 months post Dose 3 to determine parasite prevalence, parasite density and hemoglobin***, and under a separate consent, a third cross-sectional survey for efficacy will be performed in October 2008 in Kilifi and September 2008 in Korogwe to determine parasite prevalence, parasite density and hemoglobin.***
Amended 17 April 2008
- In the event that malaria rates are lower in the year of the study the cross sectional survey, unblinding and analysis scheduled at 4½ months post Dose 3 will be delayed until approximately 245 children have experienced an episode.
- Anti-CS antibody titers will be determined at baseline, 1, 4½ and 12 month post Dose 3 ***and under a separate consent, at a cross-sectional survey occurring in October 2008 in Kilifi and September 2008 in Korogwe*** in all study participants.
Amended 17 April 2008
- Anti-HBs antibody titers will be determined at baseline, 1 and 12 months post Dose 3 in all study participants.
- CS-specific IFN-γ ELISPOT responses will be evaluated at baseline, 1 and 12 months post Dose 3 in study participants at KEMRI, Kenya.
- Regulatory T-cell immune response will be determined at 4½ months post Dose 3 in all study participants.
- Anti-malaria blood stage humoral immunity will be determined at baseline, 1 and 12 months post Dose 3 in all study participants.
- Timing of final analysis is projected for 4½ months post Dose 3. The study will continue in a single-blind manner and be reported as an annex at the end of the study.
- The final analysis is projected to occur after the completion of 4 months of surveillance, at which time it is assumed that 36% of children in the control group will have been affected by a malaria episode. Assuming a true vaccine efficacy of 30%, 400 evaluable subjects per group will have 90% power to detect a difference between the groups using a two sided Fischer exact test with alpha level of 5%.
- The vaccine research teams will ensure that insecticide treated bed net use is optimized in the study population.
- Data collection: conventional Case Report Form (CRF).

4. STUDY COHORT

This study will be conducted by two investigative teams: the Joint Malaria Programme in Korogwe, Tanzania (refer to Section 4.1) and the Kenya Medical Research Institute (KEMRI) in Kilifi, Kenya (refer to Section 4.2).

Figure 1 Map of Kenya and Tanzania indicating the principal towns in the study areas

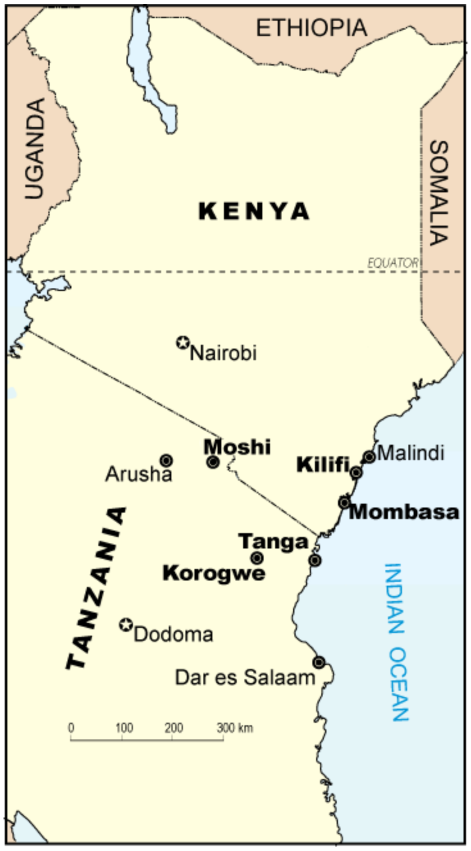

Adapted from maps created by United Nations Dept. of Public Information, Cartographic Section.

4.1. JMP Tanzania

The Joint Malaria Programme (JMP) is a collaboration between the National Institute of Medical Research (NIMR), Kilimanjaro Christian Medical Centre (KCMC), London School of Hygiene and Tropical Medicine (LSHTM) UK, and the Centre for Medical Parasitology (CMP) in Denmark.

4.1.1. JMP: Study Area Geography, Population

Subjects will be recruited from around Korogwe division in Korogwe District. Korogwe District is one of the districts of the Tanga region of Tanzania (05°09'S, 32°28'E; area 3756 km^2). The altitude within Korogwe District varies between 300 m and 1200 m above sea level. There are three distinct climatic zones in the District: 1) a cool mountainous zone 2) wet lowlands in the south and east 3) arid lowlands in the west.

The major tribes in the District include Wasambaa, Wazigua and Wabondei as well as Waha in the town of Korogwe. The population of the Korogwe District in 2002 was 261 004; the population of Korogwe Division was 78 707.

Korogwe is connected by highway to both Dar es Salaam and Arusha/Moshi.

On average there is between 500 and 1400 mm of rain per year; 1000 to 2000 mm in the mountain zone, 800 to 1000 mm in the wet lowland zone and < 800 mm per year in the arid lowlands. There are two distinct rainy seasons, with ‘short rains’ from September to November and the ‘long rains’ from March to May. Temperatures range typically from a low of 15°C from June to August and a high of 35°C from December to March.

4.1.2. JMP: Malaria Epidemiology

The main malaria vector in the Korogwe district is *Anopheles gambiae*. *An. funestus* and *An. arabiensis* are also present in the area and responsible for some transmission. It is estimated that between 16 and 28% of people sleep under a bednet. The EIR determined in 2003 was around 90 infective bites per person, per year. Transmission of malaria tends to be highest between March and July.

4.1.3. JMP: Provision of Health Care

The EPI program offers all children BCG at birth, DTPw/HepB and Oral Polio Vaccine at 1, 2 and 3 months and Measles at 9 months ***Amended 23 October 2006***.

Primary health care in the study region is provided by government run dispensaries. These typically are staffed by Clinical Officers or MCH Aid and offer outpatient treatment services.

Secondary level health care is provided by the Korogwe District Hospital. This is a district general hospital with 30 pediatric beds. The pediatric service is led by an Assistant Medical Officer. Oxygen and blood transfusion facilities are available at the District Hospital.

Referral to tertiary level facilities is rarely required, but when required will be to KCMC in Moshi. The pediatric unit has 91 beds and medical care is supervised by 7 pediatricians. Diagnostic services available include hematology, biochemistry, microbiology and radiology. In addition, there are facilities for intensive care and pediatric surgery.

4.1.4. JMP: HIV and HIV services

The prevalence of HIV in Tanzania is estimated to be approximately 9% [UNAIDS/WHO 2004a]. The prevalence among healthy children in the community is thought to be below 1%.

A Prevention of Mother to Child Transmission of HIV programme has been operational in Korogwe since 2005 and the Ministry of Health anti-viral programme is expected to reach Korogwe in the latter quarter of 2006. All patients must take part in extensive counseling before treatment. Highly Active Anti Retroviral Therapy (HAART) will be available to volunteers (if diagnosed at screening) or enrolled subjects (diagnosed during the study) with HIV ***Amended 23 October 2006***

4.2. KEMRI, Kenya

The KEMRI/Wellcome Trust collaborative programme at Kilifi was first established in 1994. The Wellcome Trust is an independent medical research funding charity based in the UK. Its mission is to foster and promote research with the aim of improving human and animal health. One of the major priorities of the Wellcome Trust's international funding is to enhance research in developing countries, by building the capacity of local partners such as the Kenyan medical Research Institute.

4.2.1. KEMRI: Study Area Geography, Population

Kilifi District stretches along the Kenyan coast from Mombasa, in the South to Malindi in the North, and for 30 to 40 km inland. The District is the second poorest in Kenya. The District of Kilifi has a population of 544 000 (KEMRI's Demographic Surveillance and the Government of Kenya Census). The largest town is Kilifi, with a population of 36 000. The town is approximately 450 km SE of Nairobi, and 75 km north of Mombasa.

The local population are predominantly from the Mijikenda ethnic group. The majority are subsistence farmers. The peak rainfall is from April to August, with the peak in the malaria transmission season from mid May to September.

4.2.2. KEMRI: Malaria Epidemiology

The primary vector of malaria is *An. gambiae*; *An funestus* also occurs in large numbers in the south of the district. The EIR is estimated to be up to 100 infective bites per person per year [Mbogo 2003]. Transmission of malaria tends to be highest between April and August.

Historically the use of bed nets has been low (<5%). In the last few years however partnerships between various NGOs and the Ministry of Health have enabled the distribution of large numbers of bed nets in the Kilifi District. Recent surveying has shown that among 1 to 5 year old children, bednet use has increased from 35% at the start of 2005 to 70% at the end of 2005.

Malaria is the commonest cause for children under the age of 18 months to be admitted to hospital.

4.2.3. KEMRI: Provision of Health Care

The EPI program offers all children BCG at birth, DTPw/Hib/HepB at 1, 2 and 3 months, Oral Polio Vaccine at 1, 2 and 3 months and Measles at 9 months ***Amended 23 October 2006***.

Primary health care in the study area is provided by a dispensary at Junju, run by the government. The dispensary operates during normal weekday working hours. A government-run health center open 7 days a week is located 14 km from Junju, in Vipingo. The center has facilities for minor surgery, obstetric care and HIV voluntary testing and counseling.

Secondary health care is provided by Kilifi District Hospital. The District Hospital has two pediatric wards; a general ward and a high dependency unit. Facilities of basic radiology, routine hematology, biochemistry and microbiology are available. Routine pediatric surgery is carried out at the District Hospital.

Referral to tertiary level facilities is rarely required, but, for example in cases requiring neurosurgery or cases of hematological malignancy, referrals are made to the government Coast General Hospital in Mombasa or the privately-run Aga Khan hospital.

Amended 23 October 2006.

4.2.4. KEMRI: HIV and HIV services

The prevalence of HIV in Kenya is estimated to be approximately 7% [UNAIDS/WHO 2004b]. In excess of 8% of children admitted to Kilifi District Hospital are HIV positive, but the prevalence among healthy children in the community is below 1%.

A campaign of Prevention of Mother to Child Transmission has been in operation for several years, and uptake of routine screening for HIV antenatally is about 60%. All patients must take part in extensive counseling before treatment. HAART is readily available to all HIV positive people in the community through a family clinic run by the Kenyan Ministry of Health, with staff from KEMRI. HAART is provided free of charge to patients at the clinic and CD4 counts are heavily subsidized (the charge may be waived for those who still cannot afford CD4 counts).

4.3. Inclusion criteria

All subjects must satisfy the following criteria at study entry:

- A male or female child of between 5 months and 17 months of age at the time of first vaccination.
- Written or oral, signed or thumb-printed and witnessed informed consent obtained from the parent(s)/guardian(s) of the child.
- Subjects who the investigator believes that their parents/guardians can and will comply with the requirements of the protocol (e.g. return for follow-up visits) should be enrolled in the study.

4.4. Exclusion criteria for enrolment

The following criteria should be checked at the time of study entry. If any apply, the subject must not be included in the study:

- Acute disease at the time of enrolment (acute disease is defined as the presence of a moderate or severe illness with or without fever). All vaccines can be administered to persons with a minor illness, such as diarrhea or mild upper respiratory infection without fever, i.e. axillary temperature < 37.5°C.

- Serious acute or chronic illness determined by clinical or physical examination and laboratory screening tests including, but not limited to:
  - Any confirmed or suspected immunosuppressive or immunodeficient condition, based on medical history and physical examination (no laboratory testing required).
  - A family history of congenital or hereditary immunodeficiency.
  - History of splenectomy.
  - Major congenital defects.
  - History of allergic reactions significant IgE-mediated events or anaphylaxis to previous immunizations.
  - History of any neurologic disorders or seizures.
  - Malnutrition at screening defined as weight for age Z-score less than -3 or other clinical signs of malnutrition.
- Laboratory screening tests out of acceptable limits:
  - refer to Table 9.
- Planned administration/administration of a vaccine not foreseen by the study protocol within 30 days of the first dose of vaccine(s) with the exception of tetanus toxoid or scheduled diphtheria, pertussis or measles vaccine.
- Use of any investigational or non-registered drug or vaccine within 30 days preceding the first dose of study vaccine, or planned use during the study period.
- Administration of immunoglobulins, blood transfusions or other blood products within the three months preceding the first dose of study vaccine or planned administration during the study period.
- Chronic administration (defined as more than 14 days) of immunosuppressants or other immune-modifying drugs within six months prior to the first vaccine dose (for corticosteroids, this will mean prednisone, or equivalent, ≥ 0.5 mg/kg/day. Inhaled and topical steroids are allowed).
- Previous participation in any other malaria vaccine trial.
- Simultaneous participation in any other clinical trial.
- Same sex twin.
- History of allergic reactions (significant IgE-mediated events) or anaphylaxis to previous immunizations.
- History of allergic disease or reactions likely to be exacerbated by any component of the vaccine.
- Any other findings that the investigator feels would increase the risk of having an adverse outcome from participation in the trial.

4.5. Elimination criteria during the study

The following criteria should be checked at each visit subsequent to the first visit. If any become applicable during the study, it will not require withdrawal of the subject from the study but may determine a subject's evaluability in the according-to-protocol (ATP) analysis.

- Use of any investigational or non-registered drug or vaccine other than the study vaccine during the study period.
- Chronic administration (defined as more than 14 days) of immunosuppressants or other immune-modifying drugs during the study period (for corticosteroids, this will mean prednisone, or equivalent, ≥ 0.5 mg/kg/day. Inhaled and topical steroids are allowed).
- Administration of a vaccine not foreseen by the study protocol during the period starting from 30 days before Dose 1 and ending 30 days after Dose 3 with the exception of vaccines for DTPw, Tetanus, *Hemophilus influenzae* type B, Measles, and Polio, for which a 14 day interval around the dose of a study vaccine will be applied.
- Administration of immunoglobulins and/or any blood products during the study period.
- Failure to thrive.

4.6. Contraindications to subsequent vaccination

4.6.1. Indications for deferral of vaccination

The following events constitute contraindications to administration of RTS,S/AS01E or Rabies vaccine at that point in time; if any one of these AEs occur at the time scheduled for vaccination, the subject may be vaccinated at a later date, within the time window specified in the protocol, or withdrawn at the discretion of the investigator. AEs should be followed-up according to the instructions in Section 8.6:

- Acute disease at the time of administration of investigational product (acute disease is defined as the presence of a moderate or severe illness with or without fever). All vaccines can be administered to persons with a minor illness such as diarrhea or mild upper respiratory infection without fever, i.e. Axillary temperature < 37.5°C.
- Axillary temperature of ≥ 37.5°C.
- Diphtheria, Tetanus, Pertussis (whole-cell), *Hemophilus influenzae* type B, Measles, or Polio vaccination within 14 days of any trial vaccination (i.e. RTS,S/AS01E or Rabies Vaccine).

4.6.2. Absolute contraindications to further vaccination

The following AEs constitute absolute contraindications to further administration of, RTS,S/AS01E or Rabies vaccine; if any of these AEs occur during the study, the subject must not receive additional doses of vaccine, but may continue other study procedures at the discretion of the investigator. AEs should be followed-up according to the instructions in Section 8.6. It is expected that the subject would continue full safety monitoring procedures, as per protocol:

- Acute allergic reaction, significant IgE-mediated event or anaphylactic shock following the administration of vaccine investigational product.
- Any confirmed or suspected immunosuppressive or immunodeficient condition, including human immunodeficiency virus (HIV) infection.

5. CONDUCT OF STUDY

5.1. Ethics and regulatory considerations

The study will be conducted according to Good Clinical Practice (GCP), the Declaration of Helsinki (Protocol Appendix A), and local rules and regulations of Tanzania and Kenya.

Submission of the protocol and any protocol amendments to regulatory agencies will occur in accordance with local regulatory requirements. The timing of the submission relative to IEC/IRB submission or approval and whether or not the authority will provide their approval of or favorable opinion on the protocol or amendment before it can be implemented will depend on local regulatory requirements.

5.1.1. Institutional Review Board/Independent Ethics Committee (IRB/IEC)

E IRB/IEC will be constituted according to the local laws/customs of each participating country. The ICH Harmonized Tripartite Guideline for Good Clinical Practice recommends that IRBs/IECs should include:

a. At least five members.

b. At least one member whose primary area of interest is in a non-scientific area.

c. At least one member who is independent of the institution/ study site.

Only those IRB/IEC members who are independent of the investigator and the sponsor of the study will vote/ provide opinion on a study-related matter.

A list of the professions of the IRBs'/IECs' members will be obtained by the Principal Investigators (PIs) or their delegates.

This protocol and any other documents that the IRBs/IECs may need to fulfill their responsibilities, including subject recruitment procedures and information about

payments and compensation available to subjects, will be submitted to each IRB/IEC by the PIs or their delegates. Written and dated unconditional approval/favorable opinion from each IRB/IEC of the protocol and amendment (if any and applicable), written informed consent form ICF), consent form updates (if any), subject recruitment procedure(s) (e.g. advertisements), and any other written information to be provided to subjects will be in the possession of the investigator and GSK before commencement of the study. This approval/favorable opinion will refer to the study by study title and number with exact protocol version and date, and will identify the documents reviewed and state the date of review. Relevant GSK Biologicals' data will be supplied by the GSK Biologicals' Central Study Coordinator to the independent IRBs/IECs for review and approval of the protocol. Verification of IRBs/IECs unconditional approval of the protocol and the written informed consent statement will be transmitted by the PIs to the GSK Biologicals' Central Study Coordinator, using the standard notification form, prior to shipment of vaccine supplies and CRFs to the site.

No deviations from, or changes to, the protocol will be initiated without prior written sponsor and IRBs'/IECs' approval/favorable opinion of an appropriate amendment, except when necessary to eliminate immediate hazards to the subjects or where permitted by all applicable regulatory requirements or when the change(s) involves only logistical or administrative aspects of the study (e.g., change of monitor[s], telephone number[s].) Administrative changes and amendments not submitted for approval will be submitted to the IRB/IEC for information only. However, written verification that such documents were submitted will be obtained. Approvals/ verifications will be transmitted in writing by the PIs.

The IRB/IEC will be informed by the Principal Investigators of:

- all subsequent protocol amendments, informed consent changes or revisions of other documents originally submitted for review,
- serious and/or unexpected adverse events occurring during the study,
- all subsequent protocol administrative changes (for information),
- new information that may affect adversely the safety of the subjects or the conduct of the study,
- regular updates and/or request for re-approval,
- when the study has been completed.

If the trial is prematurely terminated or suspended for reasons including, but not limited to, safety or ethical issues or severe non-compliance, the sponsor will promptly inform the regulatory authorities of the termination or suspension and the reason(s) for the termination or suspension. If required by applicable regulations, the PIs will inform the IEC/IRB promptly and provide the reason for the suspension or termination (see Appendix B for further details).

5.1.2. Informed consent

The details of the informed consent process are provided in Appendix C. The following principles will also apply.

In obtaining and documenting informed consent, the investigators will comply with the applicable regulatory requirement(s), and will adhere to GCP and to the ethical principles that have their origin in the appended Declaration of Helsinki. Prior to the beginning of the trial, the investigators will have the IRB/IEC's written approval/favorable opinion of the written informed consent form and any other written information to be provided to the subjects' parents/guardians.

Information should be given in both oral and written form whenever possible and as deemed appropriate by the IRB/IEC.

An investigator or designate will describe the protocol to potential subjects' parents/guardians in person. The Subject Information and Consent Form may be read to the subjects' parents/guardians, but, in any event, the investigator or designate shall give the subjects' parents/guardians ample opportunity to inquire about details of the study and ask any questions before dating and signing the Consent Form.

Subject Information and Informed Consent Forms will be in a language fully comprehensible to the prospective subjects' parents/guardians. Informed consent shall be documented by the use of a written consent form approved by the IRB/IEC and signed and dated by the subjects' parents/guardians and by the person who conducted the informed consent discussion. The signature confirms the consent is based on information that has been understood. All individuals will have the study, the Subject Information and Consent Form explained to them point by point by the interviewer in the presence of an impartial witness. The subjects' parents/guardians will thumbprint or sign the consent form. The witness will also sign and date the consent form.

Each subject's signed Informed Consent Form will be kept on file by the investigator for possible inspection by Regulatory Authorities and/or GSK Biologicals' professional and Regulatory Compliance persons. The subjects will receive a copy of the signed and dated written Informed Consent Form and any other written information provided to the subjects and should receive copies of any signed and dated consent form updates. Any amendments to the written information will be provided to subjects.

Both the informed consent discussion and the written Informed Consent Form and any other written information to be provided to the subjects' parents/guardians will include explanations of the following:

a. That the trial involves research.

b. The purpose of the trial.

c. The trial treatment(s) and the probability for random assignment to each treatment.

d. The trial procedures to be followed, including all invasive procedures.

e. The subject's and/or subject's parents'/guardians' responsibilities.

f. Those aspects of the trial that are experimental.

g. The reasonably foreseeable risks or inconveniences to the subjects and, when applicable, to an embryo, fetus or nursing infant.

h. The reasonable expected benefits. When there is no intended clinical benefit to subjects, the subjects and/or subjects' parents/guardians should be made aware of this.

i. The alternative procedure(s) or course(s) of treatment/ methods of prevention that may be available to subjects, and their important potential benefits and risks.

j. The compensation and/or treatment available to subjects in the event of trial-related injury.

k. The anticipated prorated payment, if any, to subjects / subjects' parents/guardians for participating in the trial.

l. The anticipated expenses, if any, to subjects / subjects' parents/guardians for participating in the trial.

m. That the subjects' participation in the trial is voluntary and subjects / subjects' parents/guardians may refuse to participate or withdraw from the trial, at any time, without penalty or loss of benefits to which subjects are otherwise entitled.

n. That the monitor(s), the auditor(s), the IRB/IEC and the regulatory authority(ies) will be granted direct access to the subject's original medical records for verification of clinical trial procedures and/or data, without violating the confidentiality of subjects, to the extent permitted by the applicable laws and regulations and that, by signing a written informed consent, the subject / the subject's parents/guardians is authorizing such access.

o. That records identifying subjects will be kept confidential and, to the extent permitted by the applicable laws and/or regulations, will not be made publicly available. If the results of the trial are published, subjects' identity will remain confidential.

p. That the subjects / subjects' parents/guardians will be informed in a timely manner if information becomes available that may be relevant to the subjects' / the subjects' parents/guardians willingness for continued participation in the trial.

q. The person(s) to contact for further information regarding the trial and the rights of trial subjects, and who to contact in the event of trial-related injury.

r. The foreseeable circumstances and/or reasons under which a subject's participation in the trial may be terminated.

s. The expected duration of a subject's participation in the trial.

t. The approximate number of subjects involved in the trial.

GSK Biologicals will prepare a model Informed Consent Form which will embody all the elements described above. While it is strongly recommended that this model document be followed as closely as possible, the informed consent requirements given in this document are not intended to pre-empt any local regulations which require additional information to be disclosed for informed consent to be legally effective. Clinical

judgment, local regulations and requirements should guide the final structure and content of the document.

The investigator has the final responsibility for the final presentation of Informed Consent Form, respecting the mandatory requirements of local regulations. The consent form generated by the investigator with the assistance of the sponsor's representative, must be approved (along with the protocol, and any other necessary documentation) by the IRB/IEC and be acceptable to GSK Biologicals.

5.1.3. Storage of study documentation at investigator's sites

All study documentation containing personal information relating to study subjects will be kept in a secure locked area at the investigator's sites. Such documentation will only be made available to authorized personnel. All electronic data kept at the investigator's site are kept secure. Computer access is only available to authorized personnel.

5.1.4. Safety Monitoring Plan

This trial is overseen by a Data Safety Monitoring Board (DSMB) operating under a charter assisted by a Local Safety Monitor (LSM) at each site.

5.1.4.1. Data Safety Monitoring Board (DSMB)

An independent committee consisting of experts in malaria, pediatrics, statistics and other appropriate disciplines has been appointed to oversee ethical and safety aspects of the study conduct. A quorum of 3 members is required at scheduled meetings. The composition of the DSMB is outlined in Appendix H.

The role of the DSMB includes the review of the implementation and progress of the study. It provides initial, regular, and closing advice on safety-related issues to GSK Biologicals. Its advice is based on the interpretation of study data with reference to the study protocol.

The DSMB will confer before the initiation of the study (pre-initiation review), during the study and at the close of the study. They will review the Protocol and Report and Analysis Plan (RAP). Other unscheduled meetings may be required. Meetings must be documented and minutes made available to the sponsors. The DSMB may, if deemed necessary, convene a meeting with, or request further information from the Principal Investigators, the Medical Monitor/Local Safety Monitors and GSK Biologicals' and MVI at PATH's designated project representatives at any stage of the study.

The DSMB is empowered to suspend the enrollment to the trial and/or vaccination on the trial pending review of potential safety issues arising in this trial or other relevant trials of the same product. The process will be described in study-specific SOPs.

5.1.4.1.1. Data Reviewed by the DSMB

The DSMB will be informed of:

- All SAEs.
- All withdrawals of study subjects by the Principal Investigator or the parent(s)/guardian(s) of a subject due to adverse events.
- New information that may affect adversely the safety of the subjects or the conduct of the study.
- All subsequent protocol amendments, informed consent changes or revisions of other documents originally submitted for review.
- All subsequent protocol modifications (for information).

5.1.4.2. Local Safety Monitor (LSM)

The overall role of the Local Safety Monitors (LSM), who are experienced clinicians based in-country, will be to support the clinical investigators and to act as a link between the investigators and the DSMB.

The LSM’s role will include:

- Acting as the study volunteer’s advocate.
- Promptly communicating relevant safety information to the DSMB.
- Providing advice to the investigators on whether a set of clinical circumstances in a study warrants formal notification to the DSMB.
- Unblinding a subject if deemed necessary to allow for adequate treatment.
- Liaising closely with the chair of the DSMB throughout the course of the trial.
- Suspension of vaccination for a major safety concern pending discussion with the DSMB.

5.1.4.2.1. Data Reviewed by the LSM

The relevant LSM must be informed by the investigator on an ‘as received’ basis of:

- All SAEs.
- All withdrawals of study subjects by the Principal Investigator or the parent(s)/ guardian(s) of a subject due to adverse events.

5.1.4.3. Process if the trial is suspended

Although the trial may be suspended by the DSMB, the LSMs or the Principal Investigator, it is the responsibility of the sponsor (GSK Biologicals) to make the recommendation whether or not the trial should be stopped permanently.

If the trial is suspended, the DSMB will review all available information and make a recommendation to the study sponsor (GSK Biologicals) whether to recommence the trial or to stop the trial permanently. In the event that the DSMB recommend to stop the trial permanently, the FDA will be informed by GSK Biologicals that the trial is suspended.

In the event that the trial is suspended on the recommendation of the DSMB the sponsor (GSK Biologicals) will evaluate the information. If the sponsor concurs with the DSMB's recommendation to suspend the trial, GSK Biologicals will inform the FDA that the trial has been stopped permanently. If the sponsor's recommendation is to continue, then a report will be submitted to the FDA detailing the rationale used in reaching this decision. The agreement of the FDA will be obtained prior to restarting the trial.

5.1.5. Rabies vaccination

Sanofi-Pasteur's Human Diploid Cell Rabies Vaccine will be used as the control vaccine for this trial; half of the children enrolled will be vaccinated with the control regimen.

Amended 20 December 2006

5.1.5.1. Exposure to rabies

The parents/guardians of all subjects will be reminded at Dose 1 of vaccine that should their child be bitten or scratched by an animal, they must immediately consult a physician for treatment.

5.2. Recruitment/Screening

5.2.1. Community information

The communities in which the study will take place will be informed about the nature and design of the study. Refer to Appendix C for an overview of the recruitment plan of the study.

5.2.2. Screening of volunteers

Only children with a written Informed Consent Form, signed/thumb printed and dated by parents/guardians will be screened.

Comprehension of the information contained within the Informed Consent Form will be checked prior to screening by an oral interview with the volunteer's parent(s)/guardian(s), according to SOPs at the study sites. If consent is not available from both parents/guardians, the reason for the unavailability of one of the parents/guardians will be specified on the consent form.

Subject numbers will be allocated to all volunteers who are consented for screening by their parent(s)/guardian(s). Separate lists of non-overlapping subject numbers will be provided to each site and subject numbers issued to subjects consecutively at each site.

Once consent is obtained, then per-protocol eligibility criteria will be checked, which will necessitate a physical examination and blood sampling for assessment of hematology, renal and liver function. This will be documented on clinic forms, which are prepared and filled in by the investigator. Each form will contain the subject number, information about the volunteer's date of birth, household, date of screening visit, medical history and physical and laboratory screening examination. After reviewing the medical history, physical examination and laboratory results, any reasons for non-eligibility will be documented in the CRF.

The parent(s)/guardian(s) of children who have been consented for the study and found to be ineligible will receive a full explanation by a study clinician. Any clinically relevant finding will be treated appropriately by a physician. Where necessary the child will be referred to a specialist at Coast General Hospital, Mombasa, Kenya, or KCMC Hospital in Moshi, Tanzania for evaluation and treatment as described in Section 5.7. An overview of the laboratory assays can be found in Appendix D and further details can be found in site SOPs.

A study identification card will be prepared for the parent(s)/guardian(s) of each screened subject. At screening a photograph of each screened subject being held by their parent(s)/guardian(s) will be taken and attached to the study identification card. This card will also bear the name of the study to which the child is enrolled, and the child's subject number. Parents/guardians will be instructed to come to the hospital if their child is sick and to identify their child as study participants.

5.3. Vaccination process

The RTS,S/AS01E and rabies vaccines will be packaged in identical boxes and will be identified by a treatment number. This unique treatment number will identify all doses of these vaccines administered to each subject. After randomization the treatment number assigns the subject to one vaccine group or another in a blinded way. Each subject will retain the same treatment number for their subsequent vaccine doses. The treatment number will be recorded on the subject's Clinic Form after the vaccine has been administered; information from the Clinic Form is subsequently transferred to the Case Report Form. The Clinic Form and Case Report Form link the subject number and the treatment number.

All vaccines will be administered by the intramuscular route to the left deltoid. Subjects who receive their vaccination in the incorrect arm will continue to receive subsequent blinded vaccinations as normal. The fact that the vaccine was administered in the wrong arm will be documented in clinic forms and CRF.

Vaccinations will take place at the Junju Clinic, Kenya, the Ngombezi Dispensary, Tanzania and other dispensaries in the study areas if necessary. All vaccinations will be given by a qualified person; a nurse or a doctor. A staff member experienced in the resuscitation of children will be available at all vaccination sessions. Facilities and equipment will be available to give emergency treatment in the case of an anaphylactic reaction following administration of vaccines. All children will be observed for an hour after the administration of vaccine to evaluate and treat any acute adverse events.

63f195821c4e0e87c982cb189f3de2c4

The process for each vaccination is as follows. The identity of the child will be confirmed using the study identification card. The subject number on this card will be cross-checked with that on the subject's clinic form, ensuring that the subject number on the clinic form matches that of the study identification card. On the day of the first vaccination the contraindications to vaccination, inclusion/exclusion criteria and consent form will be checked prior to vaccination. On the days of the subsequent vaccinations, elimination criteria and contraindications to vaccination will be checked prior to vaccination. A vaccine clinic form will be initiated, which will give the child's identifiers, subject number and treatment number assigned after the administration of the vaccine.

Vaccines will be administered in an observer-blinded fashion. For each vaccination during the course of the study, the Vaccine Preparer will prepare the vaccine and the Vaccinator administer the vaccine for a specific subject. Since the vaccines used in this study are of distinct appearance, the Vaccine Preparers are not blinded and together with the Vaccinators perform no other function in the study (refer to Section 6.5). The Vaccine Preparer will select the sealed box labeled with the appropriate treatment number, (containing the vials numbered with the treatment number), remove the vaccine vials and fill a syringe according to this study protocol (refer to Section 6.2). The Vaccine Preparer will then place a numbered opaque label with the subject's treatment number on the syringe. The purpose of masking the syringe is to blind the parent(s) or guardians(s) of the subject. The Vaccine Preparer will then pass the syringe to the Vaccinator in an adjacent room who will administer the vaccination. After administering the vaccine to the subject, the Vaccinator will enter the treatment number administered to the subject on the clinic form.

Subjects who cannot be vaccinated on the originally scheduled date (see Section 4.6.1) will be vaccinated within 7 days and undergo all study procedures for the visit on the same day as vaccination. In the particular case of any child found to be febrile (axillary temperature ≥ 37.5°C), a blood slide will be taken to investigate for malaria. Children will be treated as appropriate for their condition and will be followed up until resolution of any symptoms and be vaccinated if their clinical symptoms resolve within 7 days.

Those who cannot be re-vaccinated within 7 days of their scheduled date will continue all study procedures apart from receiving further study vaccinations.

5.4. Home follow-up visits for assessment of reactogenicity (7-day follow-up period)

Trained field workers under the supervision of the Principal Investigators will visit each enrolled child at daily intervals for Days 1 to 6 following each vaccination (see below in detailed study procedure; Section 5.14). In the event that the field worker finds any Grade 3 solicited general or Grade 3 unsolicited symptoms, the volunteer will be examined by a study clinician. Any further clinical data, including treatment provided, will be written on diary cards and clinic forms and transcribed onto the CRF. If the physician finds that the volunteer has experienced an SAE the appropriate measures will be taken to report this (See Section 8.7). Amended 23 October 2006.

Diary cards will be checked and verified by the Principal Investigators or designate before transcription onto CRFs after the 7-day follow-up. The Principal Investigators have a primary responsibility for the data transcribed onto the CRFs. Unresolved AEs will be followed-up by field workers until resolution under the supervision of the Principal Investigator and data will be entered onto the CRF. The procedures and frequency of visits will be outlined in an SOP at the investigators' sites.

Analgesics/antipyretics will be provided to trained field workers for the treatment of children with injection site pain and fever and their use will be documented. Parent(s)/guardian(s) will not routinely be provided with these medications.

5.5. Monitoring of hematological and biochemical laboratory parameters

Hematological and biochemical parameters will be documented on the CRF. For all values outside the acceptable limit (refer to Table 9), the reason and/or clinical condition will be documented. Results of hematological and biochemical laboratory tests will be reviewed by the PI or medically qualified designate as soon as they are generated. Any value outside the acceptable limit will be managed as appropriate by a medically qualified individual under the supervision of the Principal Investigator (refer to Section 8.6). Guidance on when to report abnormalities as SAEs is given in Section 8.3.

5.6. Surveillance for SAEs (all subjects)

5.6.1. At health facilities

Morbidity surveillance for the detection of SAEs will be in place through the health care system in place for this trial (refer to Section 5.7). Details of all outpatient attendances and admissions to health facilities will be reviewed by study clinicians to identify those meeting the criteria for SAE reporting.

In the case of a death which has occurred at home, supplementary information will be gained using the verbal autopsy technique. The verbal autopsy will be conducted according to previously published methods and detailed in the SOPs on file with the investigators [Smith, 1991].

5.7. Provision of health care

5.7.1. Health care provision to study subjects at JMP

Parents/guardians of children will be advised to seek first line health care at the dispensary at which they were enrolled. These dispensaries are staffed by clinically-qualified health workers who are on call 24 hours a day, 7 days a week. If needed participants will be referred, and if urgent, transported to Korogwe Hospital. Families will also be informed that field workers will be readily available to facilitate access to care during the course of the trial. Field workers will have access to a study clinician for

consultation by mobile phone 24 hours a day, 7 days a week and transport is available 24 hours a day, 7 days a week if necessary. Amended 23 October 2006.

Subjects may be referred to or self-present to Korogwe Hospital. Study personnel are available 24 hours a day 7 days a week to receive, investigate and document study subjects. The responsibility for nursing and medical care will be shared with the government service. Laboratory and radiological investigation will be carried out when appropriate. Treatment for medical conditions will be given according to the standard treatment regimens of Tanzania. A detailed description of the healthcare system available in the study area is provided in Section 4.1.3. Children requiring specialized care or investigation unavailable locally will be transported to KCMC Hospital in Moshi, Tanzania.

Any expenses — including transport — incurred by the parents/guardians of study participants for the purpose of obtaining a diagnosis of an adverse event as well as for clinical care related to acute conditions will be borne by the study. Long-term care for chronic conditions unrelated to study procedures will be delivered following local guidelines with no financial support from the study.

5.7.2. Health care provision to study subjects at KEMRI

Subject's families will be advised to seek first line health care at Junju Dispensary or, out of normal hours at Kilifi District Hospital. Families will also be informed that field workers will be readily available to facilitate access to care during the course of the trial. Field workers will have access to a study clinician for consultation by mobile phone 24 hours a day, 7 days a week and transport is available 24 hours a day, 7 days a week if necessary. Amended 23 October 2006.

Junju Dispensary is open during normal working hours. Subjects presented to Junju Dispensary will be seen by clinically-qualified personnel. Transfer from Junju, or from villages to Kilifi District Hospital will be facilitated if necessary. Amended 23 October 2006.

At Kilifi District Hospital KEMRI personnel are available 24 hours a day 7 days a week to receive, investigate and document study subjects. The responsibility for nursing and medical care is shared with the government service. Laboratory and radiological investigation will be carried out when appropriate. Treatment for medical conditions will be given according to the standard treatment regimens of Kenya. A detailed description of the healthcare system available in the study area is provided in Section 4.2. Tertiary care will be provided by either Coast General Hospital in Mombasa, or, for selected conditions, the Aga Khan hospital in Mombasa (refer to Section 4.2.3) Amended 23 October 2006.

Any expenses — including transport — incurred by the parents/guardians of study participants for the purpose of obtaining a diagnosis of an adverse event as well as for clinical care related to acute conditions will be borne by the study. Long-term care for chronic conditions unrelated to study procedures will be delivered following local guidelines with no financial support from the study.

5.7.3. Measles vaccination

The EPI of WHO recommends administration of Measles vaccine to children at 9 months of age in Kenya and Tanzania. Vaccination with trial vaccines will be planned so as to ensure that administration of trial vaccines to children (i.e. RTS,S/AS01E or Rabies vaccine) does not interfere with the administration of EPI vaccines. No trial vaccination will be administered within 14 days of an EPI vaccine (refer to Sections 4.4, 4.5 and 4.6).

5.8. Management and treatment of malaria in all subjects

Children with malaria that can be treated with oral medication will receive a 6-dose regimen of Coartem® (artemether/lumefantrine, Novartis AG, Zurich, Switzerland), as advocated by WHO [World Health Organization 2005] and the governments of Kenya and Tanzania.

Children who require parenteral treatment will be admitted and receive treatment with quinine, which is standard practice and effective therapy in both Kenya and Tanzania.

5.8.1. Insecticide impregnated bednets

The vaccine research teams will ensure that insecticide treated bed net use is optimized in the study population.

5.9. Active case detection (ACD) of Malaria

Active Case Detection of malaria will commence two weeks post Dose 3.

At each visit the axillary temperature of each subject will be taken and if ≥ 37.5°C a blood slide and a rapid test (OptiMal®, Flow Incorporated, Oregon, USA) to determine malaria parasitemia will be taken.

If the child is documented to be febrile and the rapid test is positive, the field worker will give the child antimalarial chemotherapy and inform a study clinician. Mobile phones will be provided to study staff. Appropriate to the clinical features of the illness, transfer of children will be arranged to a health facility, if necessary. Amended 23 October 2006.

If the child is documented to be febrile and the rapid test is negative, antimalarial chemotherapy will not be administered by the field worker. A study physician will be informed. Appropriate to the clinical features of the illness, the child's parent(s)/guardian(s) will be advised to attend their nearest health facility to be reviewed by clinically qualified personnel (medical or nursing) or transfer will be arranged to the secondary care facility.

If the parents/guardians report the child has had a fever but the axillary temperature is < 37.5°C, the field worker will revisit the child between 6 to 12 hours later to recheck the temperature and if the temperature is measured as ≥ 37.5°C a blood slide and rapid malaria test will be taken. If the test is positive, the child's case will be managed as above.

If no fever is documented at the revisit, a study physician will be informed. Appropriate to the clinical features of the illness, the child's parent(s)/guardian(s) may be advised to attend their nearest health facility to be reviewed by clinically qualified personnel (medical or nursing), or transfer will be arranged to the secondary care facility or on a study physician's advice, no further action is required. The parent(s)/guardian(s) will be informed that if they have concerns, they may always attend their nearest health facility to have their child reviewed by clinically qualified personnel.

Treatment will be indicated by rapid test result. Blood slides for parasitemia will not be read in real time to guide case management routinely. Blood slides will always be taken in duplicate.

At any time at which a child is referred to a health facility, procedures for PCD will be followed (refer to Section 5.10).

All field worker visits and revisits will be documented in the CRF. Full details for ACD visits and operational methods will be given in site-specific SOPs.

5.10. Passive case detection (PCD) of malaria

When a subject is presented to a health facility within the study areas, the child will be reviewed by clinically qualified personnel (medical or nursing) and treated as required. The temperature will be recorded and if ≥ 37.5°C a rapid test and blood slide will be performed. Antimalarial medication will be recorded and study personnel will be informed.

When a subject presents spontaneously to a fieldworker, the axillary temperature will be taken and if ≥ 37.5°C a blood slide and a rapid test (OptiMal®, Flow Incorporated, Oregon, USA) to determine malaria parasitemia will be taken.

If the child is documented to be febrile and the rapid test is positive, the field worker will give the child antimalarial chemotherapy and inform a study clinician. Mobile phones will be provided to study staff. Appropriate to the clinical features of the illness, transfer of children will be arranged to a health facility, if necessary. If the child is afebrile or the rapid test is negative, the case will be discussed with a study physician. Appropriate to the clinical features of the illness, the child's parent(s)/guardian(s) will be advised to attend their nearest health facility to be reviewed by clinically qualified personnel (medical or nursing) or transfer will be arranged to the secondary care facility. Amended 23 October 2006.

Blood slides for parasitemia will not be read in real time to guide case management routinely. Blood slides will always be taken in duplicate. Full details for PCD and operational methods will be given in site-specific SOPs.

5.11. Cross sectional surveys

Amended 23 October 2006

At the cross sectional surveys (Clinic Visit 6 and 7 ***and 9***) all children will have a blood slide taken for malaria parasitemia independent of whether they have symptomatology of malaria. To identify and treat children who have acute malaria at the time of the survey, the axillary temperature will be taken. If ≥ 37.5°C, a blood slide and a rapid test will be taken to determine malaria parasitemia. The same procedures as specified under ACD (Section 5.9) will then be followed.

Amended 17 April 2008

Blood slides will always be taken in duplicate.

5.12. Subject identification

Subjects will be issued with individual subject numbers at screening. Treatment numbers will also be assigned sequentially to eligible children at the time of Dose 1. Procedures for allocation of subject numbers and treatment numbers are detailed in Section 6.4.2.

To identify the child at subsequent contacts, each child's parent(s)/guardian(s) will be issued with an identification card bearing the child's subject number with the picture of the parent(s)/guardian(s) with the child attached to it.

Vials of RTS,S/AS01E and Rabies vaccine will be identified by a treatment number. The unique treatment number will identify all doses of the vaccine administered to each subject. The treatment number will be recorded on the clinic form and subsequently the case report form documenting the linkage between the subject number and the treatment number.

5.13. Outline of study procedures

Table 3 List of study procedures

		**DOUBLE-BLIND PHASE**										**SINGLE-BLIND**		***Extension SINGLE-BLIND***		
								**ACD**				**ACD**		***ACD***		
		**1**		**2**		**3**										
		**PCD**										**PCD**		***PCD***		
**Study Month**		**0**		**1**		**2**			**3**		**6½**	**Month 7 to 13½**	**14**			***14-19 (Oct/Sept 2008)‡***
**Study Day**	**-60 to 0**	**0**	**1 to 6**	**30**	**31 to 36**	**60**	**61 to 66**	[N]	**90**							
**Clinic Visit**	**1**	**2**		**3**		**4**			**5**		**6**[A]		**7**	***8†***		***9***
								**2 wkly visits**		**14 wkly visits**		**31 wkly visits**[B]			***1-20 wkly visits***	
**Field Worker Visit Code #**			**21 to 26**		**27 to 32**		**33 to 38**	**39, 40**		**41 to 55**		**56 to 86**[B]			***87 to 88 to 107***	
**ACD Visit**								**1 to 2**	**3**	**4 to 17**	**18**[A]	**19 to 50**[B]	**51**		***52 TO 53/73***	
**STUDY PROCEDURES**																
Informed consent	•													•*†*		
Medical history	•															
Vital Signs	○	○		○		○			○		○		○	○		○
Complete physical examination	•	○		○		○			○		○		○	○		○
Measure body weight	○	•											•	○		•
Assign Subject Number	•															
Temperature (for vaccination)		•	•	•	•	•	•									
Check inclusion/exclusion criteria	•	○														
Check elimination criteria		•		•		•										
Check contraindications to vaccination		•		•		•										
Randomization		•														
Administer RTS,S/AS01E or Rabies vaccine		•		•		•										
Recording of bednet usage& residual spraying										•[O]		•[P]				
Recording of concomitant medication		•[C]	•[C]	•[C]	•[C]	•[C]	•[C]	•[C]	•[C]	•[D]	•[D]	•[D]	•[D]	•*[D]*	•*[D]*	•*[D]*
**SAFETY DATA COLLECTION**																
Recording of solicited symptoms		•		•		•										
Recording of solicited symptoms (FW)			•		•		•									
Recording of unsolicited AEs within 1 month post-vaccination		•		•		•			•							
Recording of unsolicited Aes (FW)			•		•		•	•								

Morbidity surveillance/record SAEs	●[E]	●[F]	●[F]	●[F]	●[F]	●[F]	●[F]	●[F]	●[F]	●[F]	●[F]	●[F]	●[F]	●[F]	●[F]	●[F]
**EFFICACY DATA COLLECTION**																
Record baseline covariates	●															
Temperature (for ACD)								●	●	●	●	●	●	●[S]	●	●
Blood slide for malaria parasites (for ACD)								●[H]	●[H]	●[H]	●	●[H]	●	●[S]	●[H]	●[R]
Rapid test for malaria parasites (for ACD)								●[H]	●[H]	●[H]	●[H]	●[H]	●[H]	●[S]	●[H]	●[R]
Parasite Genotyping (for ACD)								●[H]	●[H]	●[H]	●	●[H]	●	●[S]	●[H]	●[R]
Passive Case Detection [J]		●	●	●	●	●	●	●	●	●	●	●	●	●	●	●
**SAFETY LABS**																
Complete blood count [K]	●								●		●		●			
Creatinine, ALT	●								●		●		●			
Hemoglobin																●[R]
**INVESTIGATIONAL ASSAYS**																
Antibodies to CS	●								●		●		●			●[R]
Antibodies to HBs	●								●				●			
CS-specific T-cells (ICS assay)	●								●				●			
T regulatory cells and function											●					
Anti-blood stage humoral immunity	●[Q]								●[Q]		●[Q]		●[Q]			
CS-specific T-cells (ELISPOT assay) [L]	●								●		●		●			
**FINAL ANALYSIS**											●[M]					
**STUDY CONCLUSION**													●			
**STUDY CONCLUSION**																●

for table notes, please refer overleaf…

Amended 23 October 2006
Amended 20 November 2006
Amended 17 April 2008

ACD; Active Case Detection PCD; Passive Case Detection ICS: Intracellular Cytokine Staining FW: Field Worker

[A] The survey will be postponed in the event of low case rates occurring during the first four months of surveillance

[B] These visits will occur in the double blind phase in the event that the survey at Clinic Visit 6 is postponed

[C] all antimalarial, systemic antibiotic and antipyretic medication to be recorded

[D] record administration of immunosuppressants or other immune-modifying drugs during this period (for corticosteroids this means prednisone or equivalent, ≥ 0.5 mg/kg/day. Inhaled or topical steroids are allowed and should not be recorded. ALSO all immunoglobulins and blood products should be recorded during this period. ALSO any antimalarials.

[E] SAEs related to study procedures will be collected

[F] Procedure for PCD will be followed

Amended 20 November 2006

[H] If documented fever

[J] Where the subject is febrile, blood test, rapid test, and parasite genotyping will be performed

[K] Includes analysis of hemoglobin, total white cell count and platelets.

[L] Done only in study participants enrolled by KEMRI, Kenya

[M] Final analysis will be delayed in the event of low case rates occurring during the first four months of surveillance

[N] Visit 39 to occur between 14 days post Dose 3 of vaccine and 20 days post Dose 3 of vaccine

[O] Carried out on Field Worker Visit Code # 55 ONLY

[P] Carried out on Field Worker Visit Code # 86 ONLY

Amended 23 October 2006

[Q] Samples from Tanzania will be analyzed at LSHTM and/or their collaborating laboratory, at GSK Biologicals and/or their collaborating laboratory, and in Tanzania. Samples from Kenya will be analyzed at LSHTM and/or collaborating laboratory, at GSK Biologicals and/or collaborating laboratory, and in Kenya

Amended 20 November 2006

● is used to indicate a study procedure that requires documentation in the individual CRF and ○ is used to indicate a study procedure that does not require documentation in the individual CRF. *SAEs that are related to study participation or are related to a concurrent medication will be collected and recorded from the time the subject consents to participate in the study until they are discharged.

† Clinic Visit 7 and Clinic Visit 8 can take place on the same day. For subjects who do not present at Clinic Visit 7, an interval of 14 to 17 months post Clinic Visit 2 to Clinic Visit 8 is acceptable. Subjects will be reconsented for the participation in the surveillance during the extension of the study.

‡The timing of visit 9 will be in October 2008 in Kilifi, and in September 2008 in Korogwe, in order to finish follow up at the same calender month at each site. This will mean a variable number of months after vaccination by individual children, as indicated in the table.

R Blood will not be drawn for hemoglobin or serology or parasite slide in the absence of fever from subjects who present for Clinic Visit 9 less than or equal to 14 days after Clinic Visit 7. Note that if the subject is febrile a rapid test and a parasite slide and genotyping specimens will be taken even if occurring in this period.

S If Clinic Visit 8 is more than 7 days after Clinic Visit 7 or if Clinic Visit 7 was not done.

Amended 17 April 2008

It is the investigator's responsibility to ensure that the intervals between visits/contacts are strictly followed. These intervals determine each subject's evaluability in the according to protocol analyses (see Sections 4.3 to 4.6 and 10.7 for details of criteria for evaluability and cohorts to be analyzed). The intervals are tabulated in Table 4.

Table 4 Intervals between study visits

Interval	Length of interval
Clinic Visit 1 → Clinic Visit 2	0 to 60 days
Clinic Visit 2 → Clinic Visit 3	21 to 35 days
Clinic Visit 3 → Clinic Visit 4	21 to 35 days
Clinic Visit 4 → Clinic Visit 5	21 to 42 days
Clinic Visit 5 → Clinic Visit 7	12 months ± 30 days
Clinic Visit 7→ Clinic Visit 8*	***0 to 3 months***
Clinic Visit 9	***Korogwe: September 2008 ± 14 days***<br>***Kilifi: October 2008 ± 14 days***

**** Clinic Visit 7 and Clinic Visit 8 can take place on the same day. For subjects who do not present at Clinic Visit 7, an interval of 14 to 17 months post Clinic Visit 2 to Clinic Visit 8 is acceptable.***

Amended 17 April 2008

5.14. Detailed description of study stages/visits

When materials are provided by GSK Biologicals, it is **MANDATORY** that all clinical samples (including serum samples) will be collected and stored using exclusively those materials in the appropriate manner. The use of other materials could result in the exclusion of the subject from the ATP analysis (See Section 10.7 for definition of study cohorts to be evaluated). The investigator must ensure that his/her personnel and the laboratory(ies) under his/her supervision comply with this requirement. However, when GSK Biologicals does not provide material for collecting and storing clinical samples, then appropriate materials from the investigator's site are to be used. Refer to Appendix D and Appendix E.

The vaccinees will be observed closely for at least 60 minutes, with appropriate medical treatment readily available in case of an anaphylactic reaction following the administration of vaccines.

The subjects' parents/guardians will be instructed to contact the investigator immediately should the subject manifest any signs or symptoms they perceive as serious.

On all Passive Case Detection visits the following procedures should be carried out:

- Check study identification card of vaccinee.
- Record unsolicited adverse events experienced by the vaccinee since the last visit.
- Record medication.
- Record status.
- Record maximum axillary temperature.

63f195821c4e0e87c982cb189f3de2c4

- *If subject has fever, collect blood for:*
  - Rapid test for malaria parasites.
  - Blood slide for malaria parasites.
  - Blood test for malaria parasite genotyping.

Clinic Visit 1: Screening
Day -60 to Day 0

- Obtain signed, dated, thumb printed informed consent from the parent(s)/guardians.
- Check inclusion/exclusion criteria.
- Carry out physical examination, take medical history, measure body weight, record vital signs.
- Assign subject number.
- Record any SAEs that may have occurred as a result of study procedures.
- Record baseline covariates.
- Blood sample to collect 5 mL blood for analysis of:
  - hematology (complete blood count).
  - biochemistry (creatinine and ALT).
  - serology (antibodies to CS, HBs, anti-blood-stage humoral immunity).
  - CMI:
    CS-specific T-cells (ICS);
    At KEMRI, Kenya only: CS-specific T-cells (ELISPOT).

Clinic Visit 2: Vaccination 1
Day 0

- Check study identification card of vaccinee.
- Carry out physical examination, take medical history, measure body weight, record vital signs.
- Check inclusion/exclusion criteria.
- Check contraindications to vaccination.
- Check elimination criteria.
- Randomize subjects.
- Record pre-vaccination body temperature.
- Administer the first dose of RTS,S/AS01E or rabies vaccine intramuscularly in the left deltoid.

Each child will be assessed for at least 60 minutes after vaccination to evaluate and treat any acute adverse events.

- Record any post-vaccination solicited symptoms.
- Record any post-vaccination unsolicited adverse events.
- Record any post-vaccination SAEs.
- Record concomitant medication.

Field worker post vaccination follow-up visits 21 to 26
Days 1, 2, 3, 4, 5 and 6

- Check study identification card of vaccinee.
- Record axillary temperature of subject.
- Record local (pain and swelling at the injection site) and general (fever, irritability / fussiness, drowsiness, loss of appetite) solicited adverse events.
- Record SAEs experienced by the vaccinee since the last visit.
- Record unsolicited adverse events experienced by the vaccinee since the last visit.
- Record concomitant medication.

Clinic Visit 3: Vaccination 2
Day 30

- Check study identification card of vaccinee.
- Carry out physical examination.
- Check contraindications to vaccination.
- Check elimination criteria.
- Record unsolicited adverse events experienced by the vaccinee since the last visit.
- Record pre-vaccination body temperature.
- Administer the second dose of RTS,S/AS01E or rabies vaccine intramuscularly in the left deltoid.

Each child will be assessed for at least 60 minutes after vaccination to evaluate and treat any acute adverse events.

- Record any post-vaccination solicited symptoms.
- Record any post-vaccination unsolicited adverse events.
- Record any post-vaccination SAEs.
- Record concomitant medication.

Field worker post vaccination follow-up visits 27 to 32
Days 31, 32, 33, 34, 35 and 36

- Check study identification card of vaccinee.
- Record axillary temperature of subject.
- Record local (pain and swelling at the injection site) and general (fever, irritability / fussiness, drowsiness, loss of appetite) solicited adverse events.
- Record SAEs experienced by the vaccinee since the last visit.
- Record unsolicited adverse events experienced by the vaccinee since the last visit.
- Record concomitant medication.

Clinic Visit 4: Vaccination 3
Day 60

- Check study identification card of vaccinee.
- Carry out physical examination.
- Check contraindications to vaccination.
- Check elimination criteria.
- Record unsolicited adverse events experienced by the vaccinee since the last visit.
- Record pre-vaccination body temperature.
- Administer the third dose of RTS,S/AS01E or Rabies Vaccine intramuscularly in the left deltoid.

Each child will be assessed for at least 60 minutes after vaccination to evaluate and treat any acute adverse events.

- Record any post-vaccination solicited symptoms.
- Record any post-vaccination unsolicited adverse events.
- Record any post-vaccination SAEs.
- Record concomitant medication.

Field worker post vaccination follow-up visits 33 to 38
Days 61, 62, 63, 64, 65 and 66

- Check study identification card of vaccinee.
- Record axillary temperature of subject.
- Record local (pain and swelling at the injection site) and general (fever, irritability / fussiness, drowsiness, loss of appetite) solicited adverse events.
- Record SAEs experienced by the vaccinee since the last visit.
- Record unsolicited adverse events experienced by the vaccinee since the last visit.
- Record concomitant medication.

Field Worker Visits 39 & 40: First visits for ACD

- Check study identification card of vaccinee.
- Record SAEs experienced by the vaccinee since the last visit.
- Record unsolicited adverse events experienced by the vaccinee since the last visit.
- Record concomitant medication.
- Record status.
- Record axillary temperature.
- *If subject has fever, collect blood for:*
  - Rapid test for malaria parasites.
  - Blood slide for malaria parasites.
  - Blood test for malaria parasite genotyping.

Clinic Visit 5: Cross-sectional visit for ACD
Day 90

- Check study identification card of vaccinee.
- Carry out physical examination.
- Record SAEs experienced by the vaccinee since the last visit.
- Record unsolicited adverse events experienced by the vaccinee since the last visit.
- Check elimination criteria.
- Record concomitant medication.
- Record axillary temperature of subject.
- Blood sample to collect 5 mL blood for analysis of:
  - hematology (complete blood count).
  - biochemistry (creatinine and ALT).
  - serology (antibodies to CS, HBs, anti-blood-stage humoral immunity).

- CMI:
  CS-specific T-cells (ICS);
  At KEMRI, Kenya only: CS-specific T-cells (ELISPOT).

- *In addition, blood will be drawn from all subjects for:*
  - Rapid test for malaria parasites (only if subject is febrile).
  - Blood slide for malaria parasites.
  - Blood test for malaria parasite genotyping.
  - Complete Blood Count.

Amended 20 November 2006

Field Worker Visits 41 to 55: ACD visits

- Check study identification card of vaccinee.
- Record SAEs experienced by the vaccinee since the last visit.
- Record concomitant medication.
- Record status.
- Record axillary temperature.
- Record bednet use & indoor residual spraying (Field Worker Visit 55 ONLY)

Amended 23 October 2006

- *If subject has fever, of fever collect blood for:*
  - Rapid test for malaria parasites.
  - Blood slide for malaria parasites.
  - Blood test for malaria parasite genotyping.

Clinic Visit 6: Cross-sectional visit for ACD
Month 6½

- Check study identification card of vaccinee.
- Carry out physical examination.
- Record SAEs experienced by the vaccinee since the last visit.
- Check elimination criteria.
- Record bednet usage.
- Record concomitant medication.
- Record axillary temperature of subject.
- Blood sample to collect 5.0 mL blood for analysis of:
  - hematology (complete blood count);

- biochemistry (creatinine and ALT);
- serology (antibodies to CS, anti-blood-stage humoral immunity);
- T regulatory cells and function;
- CMI:
  At KEMRI, Kenya only: CS-specific T-cells (ELISPOT).

- *In addition, blood will be drawn from all subjects for:*
  - Rapid test for malaria parasites (only if subject is febrile);
  - Blood slide for malaria parasites;
  - Blood test for malaria parasite genotyping;

Amended 23 October 2006.

Field Worker Visits 56 to 86: ACD visits

- Check study identification card of vaccinee.
- Record SAEs experienced by the vaccinee since the last visit.
- Record concomitant medication.
- Record status.
- Record axillary temperature.
- Record bednet use & indoor residual spraying (Field Worker Visit 86 ONLY)

Amended 23 October 2006

- *If subject has fever collect blood for:*
  - Rapid test for malaria parasites;
  - Blood slide for malaria parasites;
  - Blood test for malaria parasite genotyping.

Clinic Visit 7: Final study visit
Month 14

- Check study identification card of vaccinee.
- Carry out physical examination, measure body weight.
- Record SAEs experienced by the vaccinee since the last visit.
- Check elimination criteria.
- Record bednet usage.
- Record concomitant medication.
- Record axillary temperature of subject.
- Blood sample to collect 5.0 mL blood for analysis of:

- hematology (complete blood count);
- biochemistry (creatinine and ALT);
- serology (antibodies to CS, HBs, anti-blood-stage humoral immunity);
- CMI
  CS-specific T-cells (ICS);
  At KEMRI, Kenya only: CS-specific T-cells (ELISPOT).

- *In addition, blood will be drawn from all subjects for:*
  - Rapid test for malaria parasites (only if subject is febrile);
  - Blood slide for malaria parasites;
  - Blood test for malaria parasite genotyping;

Amended 23 October 2006.

Clinic Visit 8: Informed consent for study extension
Month 14 (up to Month 17)

Note: Clinic Visit 7 and Clinic Visit 8 can take place on the same day and up to 3 months post Clinic Visit 7, or for subjects who do not present at Clinic Visit 7, an interval of 14 to 17 months post Clinic Visit 2 to Clinic Visit 8 is acceptable.

- ***Check study identification card of vaccinee.***
- ***Obtain signed, dated, thumb printed informed consent from the parent(s)/guardians.***
- ***Record SAEs experienced by the vaccinee since the last visit.***
- ***Record concomitant medication.***
- ***Record status.***
- ***If Clinic Visit 8 is more than 7 days after Clinic Visit 7 or if Clinic Visit 7 was not done, record axillary temperature.***
- ***If subject has fever collect blood for:***
  - ***Rapid test for malaria parasites;***
  - ***Blood slide for malaria parasites;***
  - ***Blood test for malaria parasite genotyping.***

Amended 17 April 2008

Field Worker Visits 87 to 88 (last children to be recruited) or Visits 87 to 107 (earliest children to be recruited) : ACD visits

- ***Check study identification card of vaccinee.***
- ***Record SAEs experienced by the vaccinee since the last visit.***
- ***Record concomitant medication.***
- ***Record status.***
- ***Record axillary temperature.***
- ***If subject has fever collect blood for:***
  - ***Rapid test for malaria parasites;***
  - ***Blood slide for malaria parasites;***
  - ***Blood test for malaria parasite genotyping.***

Amended 17 April 2008

Clinic Visit 9: End of study extension visit

Month 14-19 (October 2008 for Kilifi; September 2008 for Korogwe)

- ***Check study identification card of vaccinee.***
- ***Carry out physical examination, measure body weight.***
- ***Record SAEs experienced by the vaccinee since the last visit.***
- ***Check elimination criteria.***
- ***Record concomitant medication.***
- ***Record axillary temperature of subject.***

If Clinic Visit 9 is more than 14 days after Clinic Visit 7 or if Clinic Visit 7 was not done:

- ***Blood sample to collect 1.2 mL blood for analysis of:***
  - ***Hemoglobin;***
  - ***serology (antibodies to CS);***
- ***In addition, blood will be drawn from all subjects for:***
  - ***Rapid test for malaria parasites (only if subject is febrile);***
  - ***Blood slide for malaria parasites;***
  - ***Blood test for malaria parasite genotyping.***

Amended 17 April 2008

5.15. Sample handling and analysis

5.15.1. Treatment and storage of biological samples

See Appendix D of the protocol for details of treatment and storage of biological samples.

See Appendix E for instructions for shipment of biological samples.

5.15.2. Laboratory assays

Separation of serum from the blood samples and analysis of hematology and biochemistry will be carried out at the laboratories of the respective trial sites.

Table 5 Laboratory immunological assays to be performed

Assay	Marker	Assay method	Test kit/ Manufacturer	Assay unit	Assay cut-off	Laboratory
Anti-CS antibodies	R32LR	ELISA	In-house ELISA	EU/mL	0.5	CEVAC, University of Ghent, Belgium
Anti-HBs antibodies		EIA	AUSAB EIA ABBOTT*	mIU/mL	10**	GSK Biologicals†, Rixensart, Belgium
CS-specific T-cells (ICS)		ICS	In-house ICS	Number of cytokine(s) positive cells per 10^6 cells		GSK Biologicals†, Rixensart, Belgium
CS-specific short term effectors and central memory T-cells (Elispot)		Elispot	Investigator-developed assay	Number of cytokine secreting cells per 10^6 PBMCs		KEMRI, Kenya
CS-specific T-regulatory cells		FACS and RT-PCR	Investigator-developed assay			KEMRI, Kenya and JMP, Tanzania
Anti-blood stage humoral immunity						JMP & KEMRI & LSHTM‡
						Designated collaborating laboratory

*: or equivalent
**: seroprotective level
†: or designated validated laboratory
ELISA: Enzyme-linked Immunoabsorbent Assay
EIA: Enzyme immunoassay
CEVAC: Center for Vaccinology, Ghent University
ICS: Intra-cellular cytokine staining
LSHTM: London School of Hygiene and Tropical Medicine
FACS: Fluorescent activated cell sorter
PBMC: Peripheral blood mononuclear cells

‡ Samples from Tanzania will be analyzed at LSHTM and/or their collaborating laboratory, at GSK Biologicals and/or their collaborating laboratory, and in Tanzania. Samples from Kenya will be analyzed at LSHTM and/or collaborating laboratory, at GSK Biologicals and/or collaborating laboratory, and in Kenya

Amended 23 October 2006
Amended 20 November 2006

The GSK Biologicals' laboratory at Rixensart has established Quality Control Procedures and an established Quality System. Both are audited regularly for quality assessment by an internal (sponsor-dependent) but laboratory-independent Quality Department to document the competency of the facility to perform the required tests and support the reliability of the results. Methods and equipment are validated, where required.

5.15.3. Serology and cell-mediated immunity plan

Serum for antibody determination and blood cells for CMI assessment will be collected by blood sample. Samples for safety will be analyzed at the time they are collected.

Serum samples for anti-CS and anti-HBs antibody titers determination in all study participants will be shipped to GSK Biologicals, Rixensart, Belgium.

Fixed, frozen, antigen-stimulated whole blood samples for CS-specific T-cell immune response assessment (intracellular cytokine staining assay) in all study participants will be shipped to GSK Biologicals, Rixensart, Belgium.

Plasma samples for anti-blood stage humoral immunity assessment from subjects taking part in Tanzania will be shipped to LSHTM and GSK Biologicals, Rixensart, Belgium (or designated collaborating laboratories). Samples may also be tested locally (assay details are provided in Appendix F). Plasma samples for anti-blood stage humoral immunity assessment from subjects taking part in Kenya will be shipped to LSHTM and GSK Biologicals, Rixensart, Belgium (or designated collaborating laboratories). Samples from both Kenya and Tanzania may also be tested locally (assay details are provided in Appendix F).

Amended 23 October 2006.
Amended 20 November 2006

Assessment of malaria antigen-specific T-regulatory immune responses will be done at both investigator centers (KEMRI, Kenya and JMP, Tanzania).

Assessment of CS-specific T-cells (ELISPOT) will be done at KEMRI, Kenya, on samples from children enrolled at KEMRI, Kenya only (assay details are provided in Appendix F). Amended 23 October 2006.

Any serum or plasma or blood cells not immediately used in immunological assays will be stored at -20°C or less and would only be used to assess the immune response to vaccination or to assess any potential toxicity of the vaccine Amended 23 October 2006.

Table 6 Summary of blood sampling time points/immunological assays and volumes to be collected

Blood sampling time point		Test	No. subjects	Laboratory	Priority ranking	Volume of whole blood required
**Study month**	**Clinic Visit No.**					
Day –60 to 0	1	Safety bloods	890	on-site	1	0.2 mL
		Anti-CS antibodies	890	CEVAC	2	1.0 mL
		Anti-HBs antibodies	890	GSK Biologicals*	3	
		CS-specific T-cells (ICS assay)	890	GSK Biologicals	4	2.1 mL
		Anti-blood-stage humoral immunity	890	GSK Biologicals*	8	0.3 mL
			890	LSHTM‡	5	1.4 mL
			890	JMP & KEMRI§	6	
		CS-specific T-cells (ELISPOT)	445**	KEMRI, Kenya	7	
				**Total for Clinic Visit 1**		**5.0 mL**
Month 3	5	Safety bloods	890	on-site	1	0.2 mL
		Anti-CS antibodies	890	CEVAC	2	1.0 mL
		Anti-HBs antibodies	890	GSK Biologicals*	3	
		CS-specific T-cells (ICS assay)	890	GSK Biologicals	4	2.1 mL
		Anti-blood-stage humoral immunity	890	GSK Biologicals*	8	0.3 mL
			890	LSHTM‡	5	1.4 mL
			890	JMP & KEMRI§	6	
		CS-specific T-cells (ELISPOT)	445**	KEMRI, Kenya	7	
				**Total for Clinic Visit 5**		**5.0 mL**
Month 6½	6†	Safety Bloods	890	on-site	1	0.2
		Anti-CS antibodies	890	CEVAC	2	0.5 mL
		T-regulatory cells & function	890	JMP and KEMRI	3	4.3 mL
				**Total for Clinic Visit 6**		5.0 mL
Month 14	7	Safety Bloods	890	on-site	1	0.2
		Anti-CS antibodies	890	CEVAC	2	1.0 mL
		Anti-HBs antibodies	890	GSK Biologicals*	3	
		CS-specific T-cells (ICS assay)	890	GSK Biologicals	4	2.1 mL
		Anti-blood-stage humoral immunity	890	GSK Biologicals*	8	0.3 mL
			890	LSHTM‡	5	1.6mL
			890	JMP & KEMRI§	6	
		CS-specific T-cells (ELISPOT)	445**	KEMRI, Kenya	7	
				**Total for Clinic Visit 7**		5.0 mL
⊿	***9***	***Hemoglobin***	***890***	***on-site***	***1***	***0.2***
		Anti-CS antibodies ***	***890***	***CEVAC***	***2***	***1.0 mL***
				Total for Clinic Visit 8		***1.2 mL***

*: or designated laboratory **: to be carried out on all subjects at KEMRI, Kenya WB: Whole blood ******: where Clinic Visits 7 and 9 are separated by a period of 14 days or less, the results of blood taken at Clinic Visit 7 will be used and there will be no second blood sample taken. ⊿: The timing of Clinic Visit 9 will be in October 2008 in Kilifi, and in September 2008 in Korogwe, in order to finish follow up at the same calender month at each site. This will mean a variable number of months after vaccination by individual children, as indicated in the table.***

† subjects with axillary temperature >37.5°C will also have blood drawn for a malaria rapid test for the purposes of ACD

CMI: Cell Mediated Immunity **ICS:** Intracellular Cytokine Staining

‡Note: Samples from Tanzania will be analyzed at LSHTM and/or their collaborating laboratory, at GSK Biologicals and/or their collaborating laboratory, and in Tanzania. Samples from Kenya will be analyzed at LSHTM and/or collaborating laboratory, at GSK Biologicals and/or collaborating laboratory, and in Kenya

Amended 23 October 2006.

Amended 20 November 2006

Amended 17 April 2008

5.16. Collection of covariates

Data for the calculation of covariates will be collected during this study (refer to Section 10.9.5). Study center, bednet use, geographical area, distance from health center and altitude of subject's permanent residence above sea level will be determined from the existing demographic surveillance systems in place at each site.

For bednets, a field worker will visit the home of the subject during the study (refer to Section 5.13 for timepoints) and make a direct observation of whether the child's bed has a bed net and if so, whether or not it is intact. He will ask the family whether the bednet is impregnated.

For indoor residual spraying, the field worker will ask whether the the child's bedroom has been sprayed by a residual spraying program. Refer to Section 5.13 for timepoints of the fieldworker's visit.

Amended 23 October 2006

6. INVESTIGATIONAL PRODUCTS AND ADMINISTRATION

6.1. Study vaccines

The candidate vaccines to be used have been developed and manufactured by GSK Biologicals.

The Quality Control Standards and Requirements for the candidate vaccine are described in separate release protocols and the required approvals have been obtained.

Commercial vaccines are assumed to comply with the specifications given in the manufacturer's Summary of Product Characteristics.

Refer to Appendix G for details of vaccine supplies.

6.1.1. RTS,S/AS01E vaccine

The RTS,S/AS01E vaccine consists of two fractions: the lyophilized fraction containing the RTS,S antigen in one vial and the liquid fraction, consisting of AS01E adjuvant in the other vial, to be used for reconstitution just prior to injection. A dose of 0.5 mL will be injected. The presentation of the reconstituted RTS,S/AS01E candidate malaria vaccine is an opalescent liquid.

6.1.1.1. RTS,S antigen presentation:

- The RTS,S antigen is presented as a lyophilized pellet in a 3 mL monodose vial. One dose contains approximately 25 μg of antigen with sucrose as cryoprotectant.

6.1.1.2. AS01E adjuvant:

- AS01E is presented in a 3 mL monodose vial. One dose contains 25 µg of MPL and 25 µg of Stimulon® QS21 (a triterpene glycoside purified from the bark of *Quillaja saponaria*) with liposomes.

6.1.2. Sanofi-Pasteur's Human Diploid Cell Rabies Vaccine

Sanofi-Pasteur's Human Diploid Cell Rabies Vaccine is a sterile freeze-dried suspension of rabies virus prepared from strain PM 1503-3M obtained from the Wistar Institute, Philadelphia, PA.

The potency of one dose (1.0 mL) of Sanofi-Pasteur's Human Diploid Cell Rabies Vaccine is at least 2.5 IU of rabies antigen. Sanofi-Pasteur's Human Diploid Cell Rabies Vaccine is a creamy white to orange, freeze-dried vaccine for reconstitution with the diluent prior to use; the reconstituted vaccine is a clear to slightly opaque, colorless suspension.

A dose of 1.0 mL will be injected. The presentation of the reconstituted vaccine is as a pink to red liquid. One dose of Sanofi-Pasteur's Human Diploid Cell Rabies Vaccine contains less than 100 mg albumin, less than 150 µg neomycin sulphate and 20 µg phenol red indicator. The vaccine contains no preservative or stabilizer.

Amended 20 December 2006

6.2. Dosage and administration

The vaccinees will be observed closely for at least 60 minutes following the administration of vaccines, with appropriate medical treatment readily available in case of an anaphylactic reaction.

6.2.1. RTS,S/AS01E

RTS,S/AS01E will be supplied such that the reconstituted vaccine volume will provide a 0.5 mL pediatric dose. One 0.5 mL dose will be withdrawn from each vial and used.

Disinfect top of vaccine vial (pellet) and adjuvant vial with alcohol swabs and let dry. Withdraw the contents of the adjuvant vial in a syringe and inject adjuvant into the vial of lyophilized antigen. Remove and discard the syringe and needle under appropriate safety precautions. The pellet is then dissolved by gently shaking the vial. Wait for 1 minute to ensure complete dissolution of vial contents before withdrawing a sufficient volume to provide a 0.5 mL dose (volume required for RTS,S/AS01E) of the reconstituted vaccine solution using a fresh needle and syringe for injection. The reconstituted vaccine should be administered by slow intramuscular (IM) injection, using a 25G needle with length of 1 inch (25 mm), in the left deltoid muscle within 4 hours of reconstitution (storage at +2°C to +8°C).

6.2.2. Sanofi-Pasteur's Human Diploid Cell Rabies Vaccine

Disinfect top of vaccine vial with alcohol swabs and let dry. Inject the entire contents of the diluent ***syringe*** into the vaccine vial. Keeping the syringe and needle in place, the freeze-dried vaccine is dissolved by gently shaking the vial. Wait for 1 minute to ensure complete dissolution of vial contents before withdrawing a sufficient volume to provide a 1.0 mL dose still using the original needle and original syringe. The original needle should then be replaced with a fresh needle for IM injection. The reconstituted vaccine should be used immediately.

Amended 20 December 2006

6.3. Storage

ALL VACCINE VIALS/PRE-FILLED SYRINGES AND ADJUVANTS MUST BE STORED IN THE REFRIGERATOR (+2°C to +8°C) AND MUST NOT BE FROZEN.

All vaccines will be stored in a safe and locked place with no access for unauthorized personnel. Storage temperature will be monitored daily, according to SOPs at the investigator's site. An alarm system and a back-up refrigerator will be available in case of power failure/breakdown.

The study monitor must be contacted if the cold chain is broken (e.g. vaccines become frozen or refrigeration fails).

Storage conditions for transport of vaccines from country medical department or dispatch center to study sites or between sites are described in Appendix D.

6.4. Treatment allocation and randomization

6.4.1. Randomization of supplies

There will be a separate randomization list supplied to each site with no overlap of Treatment Numbers. The randomization list will be generated at GSK Biologicals, Rixensart, using a standard SAS (Statistical Analysis System) program and will be used to number the vaccines.

In this randomization process, volunteers will be allocated randomly to one of two study groups defining which vaccine should be given (i.e. RTS,S/AS01E or Rabies vaccine). Each group will have the same number of subjects.

6.4.2. Randomization of subjects

Subjects will receive Study ID cards with Subject Numbers at the screening visit (Clinic Visit 1). Subject numbers will be issued consecutively at each site; there will be no overlap of subject numbers allocated at each site. At first vaccination visit (Clinic Visit 2 [Month 0]), after verification of eligibility criteria, subjects will be allocated a Treatment

Number in the order they present for vaccination with the lowest Treatment Number available from the randomization list for their site. The correspondence between the Subject Number and the Treatment Number will be noted down in the Clinic Form and subsequently transferred to the Case Report Form.

In order to minimize the potential for imbalance in malaria transmission exposure between recipients of RTS,S/AS01E and recipients of control vaccine, potential study subjects will be invited according to their area of residence to attend on specified days for vaccination. They will be randomized in the order that they present.

The actual treatment number used for first vaccination of the subject must be recorded by the investigator in the CRF (Rando/Treatment Allocation Section).

6.5. Method of blinding and breaking the study blind

Data pertaining to RTS,S/AS01E or rabies vaccine will be collected in a double blinded (observer blinded) manner. 'Double blinded (observer blinded)' means that the vaccine recipient and their parent(s)/guardian(s) as well as those responsible for the evaluation of safety and immunogenicity endpoints will all be unaware which treatment, RTS,S/AS01E or rabies vaccine, was administered to a particular subject. The only study staff aware of the vaccine assignment for RTS,S/AS01E or rabies vaccine will be those responsible for the storage and preparation of vaccines; these staff will play no other role in the study.

Code break envelopes, for each study enrolled subject and associating each treatment number with a specific vaccine, will be kept by the Local Safety Monitors in Kenya and Tanzania as well as by Central Safety at GSK Biologicals, Rixensart in a safe and locked place with no access for unauthorized personnel.

If deemed necessary for reasons such as safety or to guide treatment in a subject exposed to rabies, the relevant Local Safety Monitor together with the GSK Biologicals Central Safety Contact will unblind the specific enrolled subject.

A formal reporting and analysis plan (RAP) will be developed. Once the study is completed and the GSK Biologicals reference database locked, GSK Biologicals will be responsible for initiating the execution of the statistical analysis plan in collaboration with KEMRI, JMP and MVI and breaking the blind.

GSK Biologicals' policy (incorporating ICH E2A guidance, EU Clinical Trial Directive and Federal Regulations) is to unblind any serious adverse event (SAE) report associated with the use of the investigational product, which is unexpected and attributable/suspected, prior to regulatory reporting. The Clinical Safety Physician is responsible for unblinding the treatment assignment in accordance with specified time frames for expedited reporting of SAEs (Refer to Section 8.8).

6.6. Replacement of unusable vaccine doses

Additional vaccine doses will be provided to replace those that are unusable (see Appendix G for details of supplies).

In addition to the vaccine doses provided for the planned number of enrolled subjects, 3% additional doses will be supplied. In case a vaccine dose is broken or unusable, the investigator should replace it with a replacement vaccine dose. If a vaccine dose needs replacement, the envelope with the corresponding treatment number will designate the replacement without unblinding the study using a coded letter system. Although the sponsor need not be notified immediately in these cases, documentation of the use of the replacement vaccine must be recorded by the investigator on the vaccine administration page of the CRF and on the vaccine accountability form.

6.7. Packaging

See Appendix G.

6.8. Vaccine accountability

See Appendix G.

6.9. Concomitant medication/treatment

At each study visit/contact, the investigator should question the enrolled subject about any medication(s) taken.

All antipyretic, analgesic and systemic antibiotic drugs administered at ANY time during the period starting with administration of each dose and ending 30 days after each dose are to be recorded with generic name of the medication (trade names are allowed for combination drugs, i.e., multi-component drugs), medical indication, total daily dose, route of administration, start and end dates of treatment.

Amended 20 November 2006

All antimalarial drugs throughout the study period, beginning with Dose 1 are to be recorded with generic name of the medication (trade names are allowed for combination drugs, i.e., multi-component drugs), medical indication, total daily dose, route of administration, start and end dates of treatment.

Any treatments and/or medications which are listed as elimination criteria in Section 4.5, e.g., any immunoglobulins, other blood products and any immune modifying drugs administered within three months preceding the first dose or at any time during the study period are to be recorded with generic name of the medication (trade names are allowed for combination drugs only), medical indication, total daily dose, route of administration, start and end dates of treatment. Refer to Sections 4.4 and 4.5.

Any vaccine administered during the study period is to be recorded with trade name, route of administration and date(s) of administration. Refer to Sections 4.4 and 4.5.

Any Vitamin A administered from 2 months prior to Dose 1 to 30 days post Dose 3. Amended 23 October 2006.

Any concomitant medication administered prophylactically in anticipation of reaction to the vaccination must be recorded in the CRF with generic name of the medication (trade names are allowed for combination drugs only), total daily dose, route of administration, start and end dates of treatment and coded as 'Prophylactic'. The time periods between which each type of concomitant medication/treatment should be recorded is summarized in Table 7.

Table 7 Summary of time periods between which different classes of concomitant medication/treatment/vaccination must be recorded

**2 months prior to Dose 1 → Dose 1**	All treatments listed as elimination criteria in Section 4.6*, all antimalarials, Vitamin A
**Dose 1 → 30 Days post Dose 3**	All antipyretic, analgesic, systemic antibiotic, antimalarial and any treatments listed as elimination criteria in Section 4.6*, all vaccinations, Vitamin A
**31 Days post Dose 3 → *Clinic Visit 7***	All treatments listed as elimination criteria in Section 4.6*, all antimalarials, all vaccinations
Clinic Visit 8 → Final study Visit (Clinic Visit 9)	***All treatments listed as elimination criteria in Section 4.6*, all antimalarials, all vaccinations***

* e.g. any immunoglobulins, other blood products and any immune modifying drugs

Amended 23 October 2006
Amended 20 November 2006
Amended 17 April 2008

7. HEALTH ECONOMICS

Not Applicable.

8. ADVERSE EVENTS AND SERIOUS ADVERSE EVENTS

The investigators are responsible for the detection and documentation of events meeting the criteria and definition of an adverse event (AE) or serious adverse event (SAE) as provided in this protocol. During the study, when there is a safety evaluation, the investigators or site staff will be responsible for detecting AEs and SAEs, as detailed in this section of the protocol.

Each subject's parents/guardians will be instructed to contact the investigator immediately should the subject manifest any signs or symptoms they perceive as serious.

8.1. Definition of an adverse event

An AE is any untoward medical occurrence in a clinical investigation subject, temporally associated with the use of a medicinal product, whether or not considered related to the medicinal product.

An AE can therefore be any unfavorable and unintended sign (including an abnormal laboratory finding), symptom, or disease (new or exacerbated) temporally associated with the use of a medicinal product.

Examples of an AE include:

- Exacerbation of a chronic or intermittent pre-existing condition including either an increase in frequency and/or intensity of the condition.
- New conditions detected or diagnosed after investigational product administration even though it may have been present prior to the start of the study.
- Signs, symptoms, or the clinical sequelae of a suspected overdose of either investigational product or a concurrent medication (overdose per se should not be reported as an AE/SAE).
- Signs, symptoms temporally associated with vaccine administration.

AEs may include pre-or post-treatment events that occur as a result of protocol-mandated procedures (i.e., invasive procedures, modification of subject's previous therapeutic regimen).

N.B. AEs to be recorded as endpoints (solicited events) are described in Section 8.4.1. All other AEs will be recorded as **UNSOLICITED AEs**.

Example of events to be recorded in the medical history section of the CRF:

- Pre-existing conditions or signs and/or symptoms present in a subject prior to the start of the study (i.e. prior to the first study procedure) should be recorded in the medical history section of the subject's CRF.

8.2. Definition of a serious adverse event

A serious adverse event (SAE) is any untoward medical occurrence that:

a. results in death;

b. is life-threatening;

NOTE: The term 'life-threatening' in the definition of 'serious' refers to an event in which the subject was at risk of death at the time of the event. It does not refer to an event, which hypothetically might have caused death, if it were more severe.

c. requires hospitalization or prolongation of existing hospitalization;

NOTE: In general, hospitalization signifies that the subject has been detained (usually involving at least an overnight stay) at the hospital or emergency ward for observation and/or treatment that would not have been appropriate in the physician's office or out-patient setting. Complications that occur during hospitalization are AEs. If a complication prolongs hospitalization or fulfills any other serious criteria, the event is serious. When in doubt as to whether 'hospitalization' occurred or was necessary, the AE should be considered serious.

Hospitalization for elective treatment of a pre-existing condition that did not worsen from baseline is not considered an AE.

d. results in disability/incapacity;

NOTE: The term disability means a substantial disruption of a person's ability to conduct normal life functions. This definition is not intended to include experiences of relatively minor medical significance such as uncomplicated headache, nausea, vomiting, diarrhea, influenza, and accidental trauma (e.g. sprained ankle) which may interfere or prevent everyday life functions but do not constitute a substantial disruption.

e. medical or scientific judgment should be exercised in deciding whether reporting is appropriate in other situations, such as important medical events that may not be immediately life-threatening or result in death or hospitalization but may jeopardize the subject or may require medical or surgical intervention to prevent one of the other outcomes listed in the above definition. These should also be considered serious. Examples of such events are invasive or malignant cancers, intensive treatment in an emergency room or at home for allergic bronchospasm, blood dyscrasias or convulsions that do not result in hospitalization;

In this study all seizures occurring within a 30-day period of vaccination will be notified as SAEs [refer to Bonhoeffer, 2004]. Key information pertaining to seizures will be documented in the CRF.

8.3. Clinical laboratory parameters and other abnormal assessments qualifying as adverse events and serious adverse events

Abnormal laboratory findings (e.g. clinical chemistry, hematology, urinalysis) or other abnormal assessments (e.g. blood film) that are judged by the investigator to be clinically significant will be recorded as AEs or SAEs if they meet the definition of an AE, as defined in Section 8.1or SAE, as defined in Section 8.2. Clinically significant abnormal laboratory findings or other abnormal assessments that are detected during the study or are present at baseline and significantly worsen following the start of the study will be reported as AEs or SAEs.

The investigator will exercise their medical and scientific judgment in deciding whether an abnormal laboratory finding or other abnormal assessment is clinically significant.

8.4. Time period, frequency, and method of detecting adverse events and serious adverse events

All AEs occurring within 30 days following administration of each dose of vaccine must be recorded on the Adverse Event form in the subject's CRF, irrespective of severity or whether or not they are considered vaccination-related.

The standard time period for collecting and recording SAEs will begin at first receipt of vaccine and will end at the study conclusion. See Section 8.7 for instructions for reporting and recording SAEs.

Additionally, in order to fulfill international reporting obligations, SAEs that are related to study participation (e.g. procedures, invasive tests, a change from existing therapy) or are related to a concurrent medication will be collected and recorded from the time the

subject consents to participate in the study until she/he is discharged: if a child is screened and enrolled, then discharge will be the end of the study as per study protocol; if a child is screened but not enrolled, then discharge will be the point at which the decision is taken not to enroll the child.

The investigator will inquire about the occurrence of AEs/SAEs at every visit/contact during the study and throughout the follow-up phase as appropriate.

The mechanism by which SAEs will be identified in the study are detailed in Section 5.6. The investigator or study clinician will fully document any such events on the Serious Adverse Event pages appended to the individual Case Report Form including, where applicable, information from relevant hospital case records, autopsy reports and verbal autopsies.

All AEs either observed by the investigator, study clinician, field worker or reported by the subject's parent/guardian spontaneously or in response to a direct question will be evaluated by the investigator. AEs not previously documented in the study will be recorded in the Adverse Event form within the subject's CRF. The nature of each event, date and time (where appropriate) of onset, outcome, intensity and relationship to vaccination will be established. Details of any corrective treatment will be recorded on the appropriate page of the CRF. Refer to Section 6.9.

As a consistent method of determining the occurrence of unsolicited AEs, the subject or the subject's parent/guardian will be asked a non-leading question such as:

'Has your child acted differently or felt different in any way since receiving the vaccine or since the last visit'.

N.B. The investigator should record only those AEs having occurred within the time frame defined above.

AEs already documented in the CRF, i.e. at a previous assessment, and designated as 'not recovered/not resolved' or 'recovering/resolving' should be reviewed at subsequent visits, as necessary. If these have resolved, the documentation in the CRF should be completed.

When an AE/SAE occurs, it is the responsibility of the investigator to review all documentation (e.g., hospital progress notes, laboratory, verbal autopsies and diagnostics reports) relative to the event. The investigator will then record all relevant information regarding an AE/SAE on the CRF or SAE Report Form as applicable. It is not acceptable for the investigator to send photocopies of the subject's medical records to GSK Biologicals in lieu of the appropriate completed AE/SAE pages. However, there may be instances when copies of medical records and verbal autopsies for certain cases are requested by GSK Biologicals. In this instance, all subject identifiers will be blinded on the copies of the medical records prior to submission to GSK Biologicals.

The investigator will attempt to establish a diagnosis of the event based on signs, symptoms, and/or other clinical information. In such cases, the diagnosis should be documented as the AE/SAE and not the individual signs/symptoms.

8.4.1. Solicited adverse events

Local (injection site) adverse events

- Pain at injection site.
- Swelling at injection site.

General adverse events

- Fever (defined as axillary temperature ≥ 37.5°C).
- Drowsiness.
- Loss of appetite.
- Irritability/fussiness.

The visiting field worker will record these adverse events according to detailed SOPs available at the study sites during the field worker visits.

N.B. Temperature will be recorded on days 1 to 6 following each vaccination by the field worker or Principal Investigator (or their delegate). Should additional temperature measurements be performed at other times of day, the highest temperature will be recorded.

8.5. Evaluating adverse events and serious adverse events

8.5.1. Assessment of intensity

Intensity of the following AEs will be assessed as described:

Table 8 Intensity scales for solicited symptoms in infants/toddlers and children less than 6 years of age

Adverse Event	Intensity grade	Parameter
Pain at injection site	0	Absent
	1	Minor reaction to touch
	2	Cries/protests on touch
	3	Cries when limb is moved/spontaneously painful
Swelling at injection site		Record greatest surface diameter in mm
Fever*		Record temperature in °C
Irritability/Fussiness	0	Behavior as usual
	1	Crying more than usual/ no effect on normal activity
	2	Crying more than usual/ interferes with normal activity
	3	Crying that cannot be comforted/ prevents normal activity
Drowsiness	0	Behavior as usual
	1	Drowsiness easily tolerated
	2	Drowsiness that interferes with normal activity
	3	Drowsiness that prevents normal activity
Loss of appetite	0	Appetite as usual
	1	Eating less than usual/ no effect on normal activity
	2	Eating less than usual/ interferes with normal activity
	3	Not eating at all

*Fever is defined as axillary temperature ≥ 37.5°C

The maximum intensity of local injection site swelling will be scored at GSK Biologicals as follows:

0 : None
1 : < 5 mm
2 : 5 to 20 mm
3 : > 20 mm

The maximum intensity of fever will be scored at GSK Biologicals as follows:

0 : < 37.5°C
1 : 37.5 – 38.0°C
2 : > 38 – 39.0°C
3 : > 39.0°C

Table 9 Acceptable/normal ranges for blood testing

	Acceptable limit/ normal range	Toxicity grading scale			
		Grade 1	Grade 2	Grade 3	Grade 4
Hemoglobin	≥ 8.0 g/dL	< 8.0 g/dL	< 6.0 g/dL	< 5.0 g/dL	< 5.0 g/dL & clinical signs of heart failure
Total white cell count†	≥ 4.0 x 10^3/µL<br>< 17 x 10^3/µL	2.5 to 4.0 x 10^3/µL	1.5 to 2.4 x 10^3/µL	1.0 to 1.4 x 10^3/µL	< 1.0 x 10^3/µL
Platelets†	≥ 75 x 10^3/µL	50 to 74 x 10^3/µL	25 to 49 x 10^3/µL	< 25 x 10^3/µL	< 25 x 10^3/µL & clinical signs of bleeding
ALT*	≤ 60 IU/L	1.1 to 2.5 x ULN	2.6 to 5.0 x ULN	5.1 to 10.0 x ULN	> 10.0 x ULN
Creatinine*	≤ 60 µmol/L	1.1 to 1.5 x ULN	1.6 to 3.0 x ULN	3.1 to 6.0 x ULN	> 6.0 ULN or requires dialysis

† Grading scale adapted from Division of AIDS table for grading severity of adult and pediatric adverse events December 2004
* Grading scale adapted from WHO Toxicity Grading Scale for Determining Severity of Adverse Events, February 2003.
ULN: Upper Limit of Normal LLN: Lower Limit of Normal

Amended 23 October 2006

The investigator will make an assessment of the maximum intensity that occurred over the duration of the event for all other AEs, i.e. unsolicited symptoms, including SAEs reported during the study. The assessment will be based on the investigator's clinical judgment. The intensity of each AE and SAE recorded in the CRF or SAE Report Form, as applicable, should be assigned to one of the following categories:

1 (mild) = An AE which is easily tolerated by the subject, causing minimal discomfort and not interfering with everyday activities.

2 (moderate) = An AE which is sufficiently discomforting to interfere with normal everyday activities.

3 (severe) = An AE which prevents normal, everyday activities. (In a young child, such an AE would, for example, prevent attendance at school/ kindergarten/ a day-care center and would cause the parents/ guardians to seek medical advice. In adults/ adolescents, such an AE would, for example, prevent attendance at work/ school and would necessitate the administration of corrective therapy.)

An AE that is assessed as Grade 3 (severe) should not be confused with a SAE. Grade 3 is a category utilized for rating the intensity of an event; and both AEs and SAEs can be assessed as Grade 3. An event is defined as 'serious' when it meets one of the pre-defined outcomes as described in Section 8.2.

8.5.2. Assessment of causality

The investigator is obligated to assess the relationship between investigational product and the occurrence of each AE/SAE. The investigator will use clinical judgment to

determine the relationship. Alternative causes, such as natural history of the underlying diseases, concomitant therapy, other risk factors and the temporal relationship of the event to the investigational product will be considered and investigated. The investigator will also consult the Investigator Brochure and/or Product Information, for marketed products, in the determination of his/her assessment.

There may be situations when a SAE has occurred and the investigator has minimal information to include in the initial report to GSK Biologicals. However, it is very important that the investigator always makes an assessment of causality for every event prior to transmission of the SAE Report Form to GSK Biologicals. The investigator may change his/her opinion of causality in light of follow-up information, amending the SAE Report Form accordingly. The causality assessment is one of the criteria used when determining regulatory reporting requirements.

In case of concomitant administration of multiple vaccines, it may not be possible to determine the causal relationship of general AEs to the individual vaccines administered. The investigator should, therefore, assess whether the AE could be causally related to vaccination rather than to the individual vaccines.

All solicited local (injection site) reactions will be considered causally related to vaccination. Causality of all other AEs should be assessed by the investigator using the following question:

Is there a reasonable possibility that the AE may have been caused by the investigational product ?

NO	:	The AE is not causally related to administration of the study vaccine(s). There are other, more likely causes and administration of the study vaccine(s) is not suspected to have contributed to the AE.
YES	:	There is a reasonable possibility that the vaccine(s) contributed to the AE.

Non-serious and serious AEs will be evaluated as two distinct events. If an event meets the criteria to be determined ‘serious’ (see Section 8.2 for definition of serious adverse event), it will be examined by the investigator to the extent to be able to determine ALL contributing factors applicable to each serious adverse event.

Other possible contributors include:

- Medical history.
- Other medication.
- Protocol required procedure.
- Other procedure not required by the protocol.
- Lack of efficacy of the vaccine(s), if applicable.

- Erroneous administration.
- Other cause (specify).

8.6. Follow-up of adverse events and serious adverse events and assessment of outcome

After the initial AE/SAE report, the investigator is required to proactively follow each subject and provide further information to GSK Biologicals on the subject's condition.

All AEs and SAEs documented at a previous visit/contact and designated as not recovered/not resolved or recovering/resolving will be reviewed at subsequent visits/contacts.

Investigators will follow-up subjects:

- with SAEs or subjects withdrawn from the study as a result of an AE, until the event has resolved, subsided, stabilized, disappeared, the event is otherwise explained, or the subject is lost to follow-up.
- or, in the case of other non-serious AEs, until they complete the study or they are lost to follow-up.

All Grade 3, Grade 4 or clinically significant laboratory abnormalities will be followed up until they have returned to normal, or a satisfactory explanation has been provided. Additional information (including but not limited to laboratory results) relative to the subsequent course of such an abnormality noted for any subject must be made available to the Study Monitor.

GSK Biologicals may request that the investigator perform or arrange for the conduct of supplemental measurements and/or evaluations to elucidate as fully as possible the nature and/or causality of the AE or SAE. The investigator is obliged to assist. If a subject dies during participation in the study or during a recognized follow-up period, GSK Biologicals will be provided with a copy of any available post-mortem findings, including verbal autopsy and histopathology.

New or updated information will be recorded on the originally completed SAE Report Form, with all changes signed and dated by the investigator. The updated SAE report form should be resent to GSK Biologicals within 24 hours of receipt of the follow-up information as outlined in Section 8.7.1.

Outcome of any non-serious AE occurring within 30 days post-vaccination (i.e. unsolicited AE) or any SAE reported during the entire study will be assessed as:

- Recovered/resolved.
- Not recovered/not resolved.
- Recovering/resolving.
- Recovered with sequelae/resolved with sequelae.
- Fatal (SAEs only).

63f195821c4e0e87c982cb189f3de2c4

8.7. Prompt reporting of serious adverse events to GSK Biologicals

8.7.1. Time frames for submitting serious adverse event reports to GSK Biologicals

SAEs will be reported promptly to GSK once the investigator determines that the event meets the protocol definition of an SAE. The investigator or designate will fax the SAE reports to GSK Biologicals' Study Contact for Serious Adverse Event Reporting **WITHIN 24 HOURS OF HIS BECOMING AWARE OF THESE EVENTS**. Additional or follow-up information relating to the initial SAE report is also to be reported to the GSK Biologicals' Study Contact for Serious Adverse Event Reporting within 24 hours of receipt of such information.

8.7.2. Completion and transmission of serious adverse event reports to GSK Biologicals

Once an investigator becomes aware that a SAE has occurred in a study subject, she/he will report the information to GSK Biologicals Clinical Safety Physician within 24 hours as outlined in Section 8.7.1. The SAE Report Form will always be completed as thoroughly as possible with all available details of the event, signed by the investigator (or designee), and forwarded to GSK within the designated time frames. If the investigator does not have all information regarding an SAE, he/she will not wait to receive additional information before notifying GSK of the event and completing the form. The form will be updated when additional information is received and forwarded to GSK Biologicals Clinical Safety Physician **WITHIN 24 HOURS** as outlined in Section 8.7.1.

The investigator will always provide an assessment of causality at the time of the initial report as described in Section 8.5.2.

Facsimile (Fax) or electronic transmission of the SAE Report Form are the preferred methods to transmit this information to the Study Contact for Reporting SAEs. In rare circumstances and in the absence of facsimile equipment or electronic connection, notification by telephone is acceptable, with a copy of the SAE Report Form to follow. Initial notification via the telephone does not replace the need for the investigator to complete and sign the SAE Report Form within 24 hours as outlined in Section 8.7.1.

In the event of a death determined by the investigator to be related to vaccination, sending of the fax must be accompanied by telephone call to the Study Contact for Reporting SAEs.

All of the contacts listed below must be informed of each SAE occurring at JMP.

Study Contact at GSK Biologicals for Reporting Serious Adverse Events
Manager Clinical Safety Vaccines

GSK Biologicals Clinical Safety Physician,
GlaxoSmithKline Biologicals,
Rue de l'Institut 89, 1330 Rixensart,
Belgium.

Tel: +32.2.656.87.98
Fax: +32.2.656.80.09
Mobile phone for 7/7 day availability: +32.477.40.47.13 *or* +32.472.90.66.00 *or* +32.474.53.48.68
email: rix.ct-safety-vac@gskbio.com

Central Study Coordinator (JMP)

Jean François Stallaert
Central Study Coordinator,
GlaxoSmithKline Biologicals,
Rue de l'Institut 89, 1330 Rixensart,
Belgium.
Tel: +32.2.656.***94.28***
Fax: +32.2.656.80.44
email: ***jean-francois.j.stallaert@gskbio.com***

Amended 17 April 2008

Clinical Research Associate (JMP)

Ezekiel Oenga,
Clinical Research Associate,
GlaxoSmithKline, PO Box 78392-00507,
Nairobi, Kenya.

Tel: +254 20 6933 322
Fax: +254 20 558 329
email: ezekiel.r.oenga@gsk.com

Amended 23 October 2006

Local Safety Monitor (JMP)

Dr Mberesero,

Safi Medics,
Tanga,
Tanzania.

Tel: +255.264.46.46
email: +255.748.40.83.33

Malaria Vaccine Initiative

Carolyn Petersen,
Malaria Vaccine Initiative, PATH,
7500 Old Georgetown Road, 12th Floor,
Bethesda, MD 20814,
USA.

Tel: +1.240.395.27.16
or Tel: +1.240.395.27.00
Fax: +1.240.395.25.91
email: sae@malariavaccine.org

alternate email:
cpetersen@malariavaccine.org

All of the contacts listed below must be informed of each SAE occurring at KEMRI

Study Contact at GSK Biologicals for Reporting Serious Adverse Events
Manager Clinical Safety Vaccines

GSK Biologicals Clinical Safety Physician,
GlaxoSmithKline Biologicals,
Rue de l'Institut 89, 1330 Rixensart,
Belgium.

Tel: +32.2.656.87.98
Fax: +32.2.656.80.09
Mobile phone for 7/7 day availability: +32.477.40.47.13 *or* +32.472.90.66.00 *or* +32.474.53.48.68
email: rix.ct-safety-vac@gskbio.com

**Central Study Coordinator (KEMRI)**	**Clinical Research Associate (KEMRI)**
Jean François Stallaert,<br>Central Study Coordinator,<br>GlaxoSmithKline Biologicals,<br>Rue de l'Institut 89, 1330 Rixensart,<br>Belgium.<br><br>Tel: +32.2.656.94.28<br>Fax: +32.2.656.80.44<br>email: jean-francois.j.stallaert@gskbio.com	Ezekiel Oenga,<br>Clinical Research Associate,<br>GlaxoSmithKline, PO Box 78392-00507,<br>Nairobi, Kenya.<br><br>Tel: +254 20 6933 322<br>Fax: +254 20 558 329<br>email: ezekiel.r.oenga@gsk.com<br><br>Amended 23 October 2006
**Malaria Vaccine Initiative**	**Local Safety Monitor (KEMRI)**
Carolyn Petersen,<br>Malaria Vaccine Initiative, PATH,<br>7500 Old Georgetown Road, 12th Floor,<br>Bethesda, MD 20814,<br>USA.<br><br>Tel: +1.240.395.27.16<br>*or* Tel: +1.240.395.27.00<br>Fax: +1.240.395.25.91<br>email: sae@malariavaccine.org<br><br>*alternate email:*<br>cpetersen@malariavaccine.org	James Alexander Berkley,<br>KEMRI/Wellcome Trust,<br>PO Box 230,<br>Kilifi 80108,<br>Kenya.<br><br>Tel +254.41.52.25.35<br>Fax +254.4152.23.90<br>Mobile +254.720.86.70.11<br><br>email:jberkley@kilifi.kemri-wellcome.org

8.8. Regulatory reporting requirements for serious adverse events

The investigator will promptly report all SAEs to GSK in accordance with the procedures detailed in Section 8.7. GSK Biologicals has a legal responsibility to promptly notify, as appropriate, both the local regulatory authority and other regulatory agencies about the safety of a product under clinical investigation. Prompt notification of SAEs by the investigator to the Study Contact for Reporting SAEs is essential so that legal obligations and ethical responsibilities towards the safety of other subjects are met.

The investigator, or responsible person according to local requirements, will comply with the applicable local regulatory requirements related to the reporting of SAEs to the IRB/IEC and, if required, to the applicable government authority.

Investigator safety reports are prepared according to the current GSK policy and are forwarded to investigators as necessary. An investigator safety report is prepared for a SAE(s) that is both attributable to investigational product and unexpected. The purpose of the report is to fulfill specific regulatory and Good Clinical Practice (GCP) requirements, regarding the product under investigation.

An investigator who receives an investigator safety report describing a SAE(s) or other specific safety information (e.g., summary or listing of SAEs) from GSK Biologicals will file it with the Investigator Brochure or other appropriate study documentation and will notify the IRB or IEC, if appropriate according to local requirements.

8.9. Post-study adverse events and serious adverse events

A post-study AE/SAE is defined as any event that occurs outside of the AE/SAE detection period defined in Section 8.4. Investigators are not obligated to actively seek AEs or SAEs in former study participants.

However, if the investigator learns of any SAE, including a death, at any time after a subject has been discharged from the study, and he/she considers the event reasonably related to the investigational product, the investigator will promptly notify the Study Contact for Reporting SAEs.

8.10. Pregnancy

Not applicable.

8.11. Treatment of adverse events

Treatment of any adverse event is at the sole discretion of the investigator and according to current good medical practice. Any medication administered for the treatment of an AE should be recorded in the subject’s CRF. Refer to Section 6.9.

9. SUBJECT COMPLETION AND WITHDRAWAL

9.1. Subject completion

A subject who returns for the concluding visit/is available for the concluding contact foreseen in the protocol is considered to have completed the study.

A subject that comes for Clinic Visit 6 has completed the primary study phase. A subject that comes for Clinic Visit 7 has completed the extended follow-up phase of the study.

9.2. Subject withdrawal

Subjects who are withdrawn because of AEs must be clearly distinguished from subjects who are withdrawn for other reasons. Investigators will follow subjects who are withdrawn as result of a SAE/AE until resolution of the event (see Section 8.6). Withdrawals will not be replaced.

9.2.1. Subject withdrawal from the study

From an analysis perspective, a ‘withdrawal’ from the study is any subject who was not available for the concluding contact foreseen in the protocol. A subject that comes for Clinic Visit 6 has completed the primary study phase. A subject that comes for Clinic Visit 7 has completed the extended follow-up phase of the study.

A subject qualifies as a ‘withdrawal’ from the study when no study procedure has occurred, no follow-up has been performed and no further information has been collected for this subject from the date of withdrawal/last contact.

Investigators will make an attempt to contact those subjects who do not return for scheduled visits or follow-up.

Information relative to the withdrawal will be documented on the Study Conclusion page of the CRF. The investigator will document whether the decision to withdraw from the study was made by the subject’s parent or guardian or the investigator and which of the following possible reasons was responsible for withdrawal:

- serious adverse event.
- non-serious adverse event.
- protocol violation (specify).
- consent withdrawal, not due to an adverse event.
- moved from the study area.
- lost to follow-up.
- death.
- other (specify).

9.2.2. Subject withdrawal from investigational product

A 'withdrawal' from the investigational product is any subject who does not receive the complete treatment, i.e. when no further planned dose is administered from the date of withdrawal. A subject withdrawn from the investigational product may not necessarily be withdrawn from the study as further study procedures or follow-up may be performed (safety or immunogenicity) if planned in the study protocol.

Information relative to premature discontinuation of the investigational product will be documented on the Vaccine Administration page of the CRF. The investigator will document whether the decision to discontinue further vaccination/treatment was made by the subject's parent or guardian or the investigator and which of the following possible reasons was responsible for withdrawal:

- serious adverse event.
- non-serious adverse event.
- other (specify).

10. DATA EVALUATION: CRITERIA FOR EVALUATION OF OBJECTIVES

10.1. Primary endpoint

NOTE: *In the event that malaria rates are lower in the year of the study the cross sectional survey, unblinding and analysis scheduled at 4½ months post Dose 3 will be delayed until approximately 245 children have experienced an episode.*

- Time to first case of malaria meeting the primary case definition over a period starting 14 days after Dose 3 and extending for 4 months.

10.2. Secondary endpoints

Efficacy

- Time to first case of malaria meeting the secondary case definition over a period starting 14 days after Dose 3 and extending for 4 months.
- Multiple events of malaria meeting the primary case definition over a period starting 14 days after Dose 3 and extending for 4 months.
- Multiple events of malaria meeting the secondary case definition over a period starting 14 days after Dose 3 and extending for 4 months.
- Parasite prevalence and density at 4½ months post Dose 3.
- Hemoglobin at 4½ months post Dose 3.

Safety

- Occurrence of solicited symptoms and local reactions over a 7-day follow-up

period (day of vaccination and 6 subsequent days) after each vaccination.

- Occurrence of unsolicited symptoms over a 30-day follow-up period (day of vaccination and 29 subsequent days) after each vaccination.
- Occurrence of serious adverse events from the time of first vaccination (Month 0) until 4½ months post Dose 3 (Month 6½).
- Occurrence of parameters of hematological monitoring outside acceptable ranges.

Immunogenicity

- Anti-CS antibody titers prior to vaccination, 1 month post Dose 3 and 4½ months post Dose 3.
- Anti-HBs antibody titers prior to vaccination and 1 month post Dose 3.
- Frequency of CD4+ and CD8+ CS-specific T-cells expressing at least two of the following: IL-2, CD40L, TNF-α and IFN-γ prior to vaccination and 1 month post Dose 3.

10.3. Tertiary endpoints

Efficacy

- Time to first case of malaria meeting the primary case definition over a period starting 14 days after Dose 3 and extending for 12 months ***and a period starting 14 days after Dose 3 and extending until October 2008 in Kilifi and September 2008 in Korogwe***.

  Amended 17 April 2008
- Time to first case of malaria meeting the secondary case definition over a period starting 14 days after Dose 3 and extending for 12 months ***and a period starting 14 days after Dose 3 and extending until October 2008 in Kilifi and September 2008 in Korogwe***.

  Amended 17 April 2008
- Multiple events of malaria meeting the primary case definition over a period starting 14 days after Dose 3 and extending for 12 months ***and a period starting 14 days after Dose 3 and extending until October 2008 in Kilifi and September 2008 in Korogwe***.

  Amended 17 April 2008
- Multiple events of malaria meeting the secondary case definition over a period starting 14 days after Dose 3 and extending for 12 months ***and a period starting 14 days after Dose 3 and extending until October 2008 in Kilifi and September 2008 in Korogwe***.

  Amended 17 April 2008
- Parasite prevalence and density at 12 months post Dose 3 ***and in October 2008 in Kilifi and in September 2008 in Korogwe***.

  Amended 17 April 2008

- Hemoglobin at 12 months post Dose 3 ***and in October 2008 in Kilifi and in September 2008 in Korogwe***.

Amended 17 April 2008

Safety

- Occurrence of SAEs throughout the entire study period.

Immunogenicity

- Anti-CS antibody titers at 12 months post Dose 3 (Month 14) ***and in October 2008 in Kilifi and in September 2008 in Korogwe***.

Amended 17 April 2008

- Anti-HBs antibody titers at 12 months post Dose 3.
- Frequency of CD4+ and CD8+ CS-specific T-cells expressing at least two of the following: IL-2, CD40L, TNF-α and IFN-γ prior to vaccination and 12 months post Dose 3.

10.4. Exploratory endpoint

Immunogenicity

- Numbers of IFN-γ producing cells upon CS stimulation in short and long term culture Elispot assays at baseline,one month and 12 months post Dose 3.
- Frequency of malaria antigen-specific effector cells and regulatory T-cells and levels of expression of effector and regulatory cytokines at 4½ months post Dose 3.
- Anti-malaria blood stage antigens antibody levels at baseline, one month, 4½ months and 12 months post Dose 3.

10.5. Case Definitions for Malaria

**Primary malaria case definition**	*P falciparum* infection > 2 500 parasites per µL in a child with a documented fever ≥ 37.5°C (axillary) detected by ACD or PCD.
**Secondary malaria case definition**	*P falciparum* infection > 0 parasites per µL in a child with a documented fever ≥ 37.5°C (axillary) detected by ACD or PCD.

Amended 23 October 2006

10.6. Estimated sample size

10.6.1. Power Calculations

The final analysis is projected to occur after the completion of 4 months of surveillance, at which time it is assumed that 36% of children in the control group will have been affected by a malaria episode. Assuming a true vaccine efficacy of 30% and an attack rate of 36% in controls (corresponding to an attack rate of 25.2% in vaccinees), a sample size of 400 evaluable subjects per group has 90% power to detect a difference between groups using a 2-sided Fisher exact test at 5% significance level. The final analysis is projected to occur after the completion of 4 months of surveillance, at which time it is assumed that 36% of children in the control group will have been affected by a malaria episode. However, in the event that malaria rates are lower in the year of the study, the unblinding and analysis will be delayed until approximately 245 children have experienced an episode (400*36% + 400*25.2% = 245). This analysis will also have 90% power.

The assumption of 36% attack rate in controls is based on epidemiological data collected at the two sites. In Kilifi the proportion of children aged 12 to 18 months olds affected by a malaria episode using the same ACD methodology as proposed in this document over the same time frame in 2005 was 37% (personal communication P. Bejon). In Korogwe the case rate was 34% determined in children aged 5 to 17 months in Mkokola village (10 km from Korogwe) by Passive Case Detection at village level (personal communication J Lusingu, B Mmbando).

10.7. Study cohorts to be evaluated

10.7.1. Total Vaccinated cohort

The Total Vaccinated Cohort will include all vaccinated subjects for whom data are available. Thus, the total analysis of safety will include all subjects with at least one vaccine administration documented; the total analysis of efficacy will include all subjects with at least one vaccine administration documented and data concerning efficacy endpoint measures are available; the total analysis of immunogenicity will include vaccinated subjects for whom data concerning immunogenicity endpoint measures are available. The Total Vaccinated Cohort analysis will be performed per treatment actually administered.

10.7.2. According-to-Protocol (ATP) cohort for analysis of safety

The ATP cohort for analysis of safety will include all evaluable subjects;

- who have received at least one dose of study vaccine according to their random assignment.
- have sufficient data to perform an analysis of safety (at least one vaccine dose with safety follow-up).
- for whom administration site of study vaccine is per protocol.

- who have not received a vaccine not specified or forbidden in the protocol and for whom elimination criteria were not applied.
- for whom the randomization code has not been broken.
- who meet all eligibility criteria.

10.7.3. According-to-Protocol (ATP) cohort for analysis of immunogenicity

The ATP cohort for analysis of immunogenicity will include all evaluable subjects (i.e. those meeting all eligibility criteria, complying with the procedures defined in the protocol, with no elimination criteria during the study) for whom data concerning immunogenicity endpoint measures are available.

10.7.4. According-to-Protocol (ATP) cohort for analysis of efficacy

The ATP cohort for analysis of efficacy will include all evaluable subjects (i.e. those meeting all eligibility criteria, complying with the procedures defined in the protocol, with no elimination criteria during the study) for whom data concerning efficacy endpoint measures are available.

10.8. Derived and transformed data

- Seroprotection rate for anti-HBsAg is defined as the percentage of subjects with antibody titers greater than or equal to an established cut-off (anti-HBsAg $\geq$ 10 mIU/mL).
- A subject seropositive for anti-CS antibody is a subject whose antibody titer is greater than or equal to the cut-off value (anti-CS $\geq$ 0.5 EU/mL).
- The Geometric Mean Titers (GMTs) calculations are performed by taking the anti-log of the mean of the log10 titer transformations. Antibody titers below the cut-off of the assay will be given an arbitrary value of half the cut-off for the purpose of GMT calculation.

For a given subject and a given immunogenicity measurement, missing or non-evaluable measurements will not be replaced. Therefore, an analysis will exclude subjects with missing or non-evaluable measurements.

10.9. Statistical methods for Vaccine Efficacy endpoints

10.9.1. Time to event analyses

For time to endpoints (time to first or only episode of *P. falciparum* malaria), the incidence of episodes (episodes/person years at risk) for each group will be presented. Kaplan-Meier curves for both groups will be shown. The distribution of the “time to” will be compared with the Wilcoxon test (if efficacy varies with time) or the Log-rank test (if it does not). Vaccine Efficacy will be assessed using Cox regression models. Vaccine

efficacy is defined as 1 *minus* R where R is the hazard ratio of the RTS,S/AS01E group versus the Control group (with 95% CI). Crude and adjusted estimates for covariates (possible covariates include: study center, bed-net use, geographical area and distance from health center) will be presented.

Cox regression assumes proportional hazards throughout the follow-up period. This assumption will be checked by plotting per group the log of the cumulative hazard against the log of time. Under the assumption of proportionality of the hazard, both curves should be parallel. A test based on the Schoenfeld residuals will be performed.

If there is strong evidence that the hazard is not constant over the surveillance period, then alternative approaches to analyze the data may be examined according to the following:

- Cox regression models with time varying covariates, using fractional polynomials to identify the function of the time that best fits. The following functions of the time will be explored : –2, -1, -½, log, identity, ½, 2, 3. The Akaike's criterion and the Schwarz Bayesian criterion will be explored.
- Accelerated failure time (AFT) models using 'Generalized Gamma', 'Standard Gamma', 'log-normal', 'weibull' and 'exponential' distributions.

In order to assess the goodness-of-fit of these models, a graphical representation of the expected survival curve against the observed using the Kaplan-Meier methodology will be presented. Where appropriate, likelihood ratio tests will be carried out to investigate the goodness-of-fit of the different models if appropriate.

10.9.2. Total number of events analyses

For the analysis of multiple episodes (clinical malaria), a table with the frequency of children with 0, 1, 2, 3, 4 or more episodes in each group will be presented. Differences in the distribution of number of episodes will be evaluated using a Fisher exact test. The incidence of episodes (episodes/person years at risk) for each group will be presented. The significance of the group effect will be assessed using Poisson regression models with random effects including the time at risk as an off-set variable. If there is evidence of lack-of-fit to the Poisson model, alternative models will be investigated. Vaccine efficacy is defined as 1 *minus* R where R is the rate ratio of clinical episodes of the RTS,S/AS01E group versus the Control group (with 95% CI). Crude and adjusted estimates for covariates will be presented if appropriate.

10.9.3. Cross-sectional analyses

Hemoglobin mean and standard deviation will be presented (following cross-sectional visit at Clinic Visit 6 and Month 14 ***and Clinic Visit 9***) and 95% confidence intervals will be calculated. The effect of the group will be evaluated using a non-parametric Wilcoxon test.

For the assessment of the parasite density at following cross-sectional visit at Clinic Visits 6 and 7 ***and 9***, a 2x2 table presenting the frequency of positive and negative will be

presented. The effect of the group will be evaluated using the Fisher exact test. The Geometric mean of those positive in each group will be calculated. Difference in geometric mean and 95% CI will be estimated. The effect of the group will be evaluated using a non-parametric Wilcoxon test.

Amended 17 April 2008

10.9.4. Calculation of time at risk

For efficacy endpoints, the time at risk will be counted in days, and expressed as person years at risk (days/365.25).

For each subject in the Total Cohort, time at risk begins 14 days after Dose 3 for those that received Dose 3 or 2½ months after Dose 1 (Day 75) otherwise. For the According to Protocol Cohort, time at risk begins 14 days after Dose 3.

The time at risk will end whenever one of the following conditions happen first: endpoint definition met, loss of follow up, emigration from the study area, withdrawal, death, end of follow-up period.

The time at risk will take into account absences from the study area and anti-malaria drug therapy. Absences from the study area of 1 week or more will be recorded in multiples of 1 week. The date of departure will be documented. Similarly, if treatment for malaria is administered, the subject will not be considered susceptible to malaria infection for the longest duration of the combination of drugs the subject could receive for this episode as follows: 28 days if received Coartem®, 28 days if having received sulfadoxine-pyrimethamine and 7 days if having received quinine alone.

If an episode is detected during a period of time not counting for the time at risk it will not be included in the analysis, however a table will be presented showing the number of such episodes not included in the analysis.

Episodes will be included in the analysis only if the subject is identified properly showing his/her ID Card at the contact with the health facility. Only blood slides that complete the reading process according to SOP will be considered in the establishment of endpoints.

10.9.5. Covariates

The risk for malaria and development of clinical disease depends on numerous factors related to the parasite, host and vector biology. In addition to demographic data, covariates to be collected in this study will include:

- study center.
- bednet use.
- residual spraying
- geographical area.
- distance from health center.

- altitude above sea level of subject's permanent residence.

Covariates included in the adjustment for efficacy will be defined in the Report Analysis Plan. Amended 23 October 2006.

10.10. Final analyses

The final analysis will be conducted on data collected until Clinic Visit 6. The study will continue in a single-blind manner. This additional information will be appended to the study report.

In the event that malaria rates are lower in the year of the study the unblinding and analysis following Clinic Visit 6 will be delayed until approximately 245 children have experienced an episode. This analysis will have 90% power assuming true vaccine efficacy to be 30%.

10.10.1. Analysis of efficacy

Analysis of efficacy will be performed on the ATP Cohort for Efficacy and Total Cohort. Vaccine efficacy endpoints:

- time to first/only case of malaria.
- multiple events of malaria.
- parasite prevalence and density.
- hemoglobin values.

will be analyzed according to methods described in Section 10.9.

10.10.2. Analysis of demographics/baseline characteristics

Demographic characteristics (age, gender) and baseline covariates of each study cohort will be tabulated.

The mean age (plus range and standard deviation) by gender of the enrolled subjects, as a whole, and per group, will be calculated.

The distribution of subjects enrolled by study site will be tabulated as a whole and per group.

10.10.3. Analysis of immunogenicity

The primary analysis will be based on the ATP cohort for the analysis of immunogenicity. If the percent of enrolled subjects excluded from this ATP cohort is more than 5%, a second analysis based on the Total Vaccinated Cohort will be performed to complement the ATP analysis.

10.10.3.1. Anti-CS antibodies

The percentage of subjects seropositive for anti-CS (proportion of subjects with anti-CS antibody titers greater than or equal to 0.5 EU/mL) with 95% CI will be determined at each blood sampling time point. Antibody titers will be summarized by GMT with 95% CI at all time points at which serological samples are taken. Tables will be generated by vaccine group and by protection status (protected subjects are those recipients of RTS,S/AS01E who are not diagnosed with malaria meeting the primary case definition within the 4½ month ACD period). Antibody titers after the third dose will also be investigated using reverse cumulative curves. Comparisons between protected and non-protected may be performed using Wilcoxon Rank Sum tests.

10.10.3.2. Anti-HBs antibodies

The seroprotective level for anti-HBs is ≥ 10 mIU/mL. The percentage of subjects with protective levels of anti-HBs (≥ 10 mIU/mL) with 95% confidence interval (95% CI) will be determined at each blood sampling time point. Antibody titers will be summarized by GMT with 95% CI. Tables will be generated by vaccine group and by protection status (protected subjects are those recipients of RTS,S/AS01E who are not diagnosed with malaria meeting the primary case definition within the 4½ month ACD period). Antibody titers after the third dose will also be investigated using reverse cumulative curves. Comparisons between protected and non-protected may be performed using Wilcoxon Rank Sum tests.

10.10.3.3. Frequency of T-cells specific to CS

The frequency of T-cells specific to CS will be identified as CD4/CD8 + T-cells expressing at least two cytokines amongst CD40L, IL-2, TNF-α and IFN-γ upon a short term in vitro stimulation with a pool of peptides covering the vaccine CS portion. Results will be expressed as CS-specific CD4/CD8 T-cells per million respectively of CD4 or CD8+ T-cells. Descriptive statistics (N, minimum, Q1, median, Q3, maximum, mean, standard deviation) will be tabulated by group and protection status, for pre and post vaccination timepoints (pre and 1 month post Dose 3) (protected subjects are those recipients of RTS,S/AS01E who are not diagnosed with malaria meeting the primary case definition within the 4½ month ACD period). Analysis by protection status will be limited to recipients of RTS,S/AS01E. Results will also be presented graphically by box plots. Between group comparisons and between protection status comparisons will be performed by non-parametric Wilcoxon Rank Sum tests. Within group comparisons (i.e. comparing post versus pre timepoint within the RTS,S group) will be performed by Wilcoxon Signed Rank test for paired samples.

10.10.3.4. Exploratory immunogenicity endpoints

The frequency of T-cells specific to CS will be identified as cytokine secreting/expressing cells using cytokine ELISPOT assay or intracellular cytokine assays. Descriptive statistics (N, minimum, Q1, median, Q3, maximum, mean, standard deviation) will be tabulated by group and protection status, for pre and post vaccination timepoints (pre and 1 month post Dose 3), when available (protected subjects are those

recipients of RTS,S/AS01E who are not diagnosed with malaria meeting the primary case definition within the 4½ month ACD period). Results will also be presented graphically by box plots. Between group comparisons and between protection status comparisons will be performed by non-parametric Wilcoxon Rank Sum tests. Within group comparisons (i.e. comparing post versus pre timepoint within the RTS,S group) will be performed by Wilcoxon Signed Rank test for paired samples.

10.10.4. Analysis of safety

10.10.4.1. Secondary safety endpoints

Safety will be analyzed on the Total Vaccinated Cohort. If the percent of enrolled subjects excluded from the ATP cohort for analysis of safety is more than 5%, a second analysis based on this ATP cohort will be performed to complement the Total Vaccinated Cohort analysis.

Analysis of the occurrence of SAEs will be performed on the Total Vaccinated Cohort. The proportion of subjects with an SAE, classified by the MedDRA preferred term level, reported from study start until Clinic Visit 6 (end of the double-blind phase) will be tabulated with exact 95% CI.

The proportion of subjects reporting an AE (unsolicited) 30 days post each vaccination, classified by the MedDRA preferred term level, reported from study start until the end of the double-blind phase will be tabulated with exact 95% CI.

The percentage of subjects with at least one local adverse event (solicited and unsolicited), with at least one general adverse event (solicited and unsolicited) and with any adverse event during the solicited follow-up period will be tabulated with exact 95% CI after each vaccine dose and overall. The percentage of doses followed by at least one local adverse event (solicited and unsolicited), by at least one general adverse event (solicited and unsolicited) and by any adverse event will be tabulated, overall vaccination course, with exact 95% CI. Similar tables will be generated for Grade 3 events, the relationship of the event to vaccination.

The percentage of subjects reporting each individual solicited local and general adverse event during the solicited follow-up period will be tabulated with exact 95% CI. The percentage of doses followed by each individual solicited local and general adverse event will be tabulated, overall vaccination course, with exact 95% CI. Similar tables will be generated for Grade 3 events, the relationship of the event to vaccination and for fever and temperature in 0.5°C increments.

Biochemistry (ALT and creatinine) and hematology (hemoglobin, WBC, platelets) values that are outside of the reference ranges will be described for Clinic Visit 6 (end of double-blind phase). Frequency distribution of results by toxicity grades will be tabulated by group.

10.11. Annex analysis at study conclusion

10.11.1. Analysis of efficacy

Analysis of efficacy will be performed on the ATP Cohort for Efficacy and Total Cohort. Vaccine efficacy endpoints:

- time to first/only case of malaria.
- multiple events of malaria.
- parasite prevalence and density.
- hemoglobin values.

will be analyzed according to methods described in Section 10.9.

10.11.2. Analysis of safety

Analysis of the occurrence of SAEs will be performed on the Total Vaccinated Cohort. The proportion of subjects with an SAE, classified by the MedDRA preferred term level, reported from study start until Clinic Visit 7 ***and from study start until Clinic Visit 9*** will be tabulated with exact 95% CI.

Amended 17 April 2008

10.11.3. Analysis of immunogenicity

The primary analysis will be based on the ATP cohort for the analysis of immunogenicity. If the percent of enrolled subjects excluded from this ATP cohort is more than 5%, a second analysis based on the Total Vaccinated Cohort will be performed to complement the ATP analysis.

10.11.3.1. Anti-CS antibodies

The percentage of subjects seropositive for anti-CS (proportion of subjects with anti-CS antibody titers greater than or equal to 0.5 EU/mL) with 95% CI will be determined at Clinic Visit 7 ***and Clinic Visit 9***. Antibody titers will be summarized by GMT with 95% CI. Tables will be generated by vaccine group and by protection status (protected subjects are those recipients of RTS,S/AS01E who are not diagnosed with malaria meeting the primary case definition within the 4½ month ACD period). Comparisons between protected and non-protected may be performed using Wilcoxon Rank Sum tests.

Amended 17 April 2008

10.11.3.2. Anti-HBs antibodies

The seroprotective level for anti-HBs is ≥ 10 mIU/mL. The percentage of subjects with protective levels of anti-HBs (≥ 10 mIU/mL) with 95% confidence interval (95% CI) will be determined at Clinic Visit 7. Antibody titers will be summarized by GMT with 95%

CI. Tables will be generated by vaccine group and by protection status (protected subjects are those recipients of RTS,S/AS01E who are not diagnosed with malaria meeting the primary case definition within the 4½ month ACD period). Comparisons between protected and non-protected may be performed using Wilcoxon Rank Sum tests.

10.11.3.3. Frequency of CD4+/CD8+ CS-specific T-cells

The frequency of CD3+ CD4+/CD8+ T-cells specific to CS will be identified as CD4/CD8 + T-cells expressing at least two cytokines amongst CD40L, IL-2, TNF-α and IFN-γ upon short-term in vitro stimulation with a pool of peptides covering the vaccine CS portion. Results will be expressed as CS-specific CD4/CD8 T-cells per million respectively of CD4 or CD8+ T-cells. Descriptive statistics (N, minimum, Q1, median, Q3, maximum, mean, standard deviation) will be tabulated by group and protection status at Clinic Visit 7 (protected subjects are those recipients of RTS,S/AS01E who are not diagnosed with malaria meeting the primary case definition within the 4½ month ACD period). Analysis by protection status will be limited to recipients of RTS,S/AS01E. Results will also be presented graphically by box plots. Between group comparisons and between protection status comparisons will be performed by non-parametric Wilcoxon Rank Sum tests. Within group comparisons (i.e. comparing post versus pre timepoint within the RTS,S group) will be performed by Wilcoxon Signed Rank test for paired samples.

10.12. Planned interim analysis

No interim analysis is planned in this study. Final analysis will be carried out at Month 6½, or after 245 subjects have experienced an episode of malaria.

11. ADMINISTRATIVE MATTERS

To comply with Good Clinical Practice important administrative obligations relating to investigator responsibilities, monitoring, archiving data, audits, confidentiality and publications must be fulfilled. See Appendix B for details.

12. REFERENCES

Ajjan N, Soulebot JP, Stellmann C, Biron G, Charbonnier C, Triau R et al. Results of preventive rabies vaccination with a concentrated vaccine of the PM/WI38-1503-3M rabies strain cultured on human diploid cells. Preparation of mixed antirabies-antitetanus hyperimmune immunoglobulin by plasmapheresis of blood taken from vaccinated veterinary students. *Dev Biol Stand* 1978; 40: 89-199.

Alloueche A, Milligan P, Conway DJ, Pinder M, Bojang K, Doherty T et al. Protective efficacy of the RTS,S/AS02 *Plasmodium falciparum* malaria vaccine is not strain specific. *American Journal of Tropical Medicine and Hygiene.* 2003; 68: 97-101.

Alonso PL, Sacarlal J, Aponte JJ, Leach A, Macete E et al. Efficacy of the RTS,S/AS02A vaccine against *Plasmodium falciparum* infection and disease in young African children: randomised controlled trial. *The Lancet*. 2004; 364: 1411-1420.

Alonso PL, Sacarlal J, Aponte JJ, Leach A, Macete E et al. Duration of protection with RTS,S/AS02A malaria vaccine in prevention of *Plasmodium falciparum* disease in Mozambican children: single-blind extended follow-up of a randomised controlled trial. *The Lancet.* 2005; 366: 2012-2018.

Aoki FY, Tyrell DAJ, Hill LE. Immunogenicity and acceptability of a human diploid cell culture rabies vaccine in volunteers. The Lancet. 1975; 660-2.

Bernard KW, Roberts MA, Sumner J, Winkler WG, Mallonee J, Baer GM, Chaney R. Human diploid cell rabies vaccine. Journal of the American Medical Association. 1982; 247: 1138-42.

Bojang KA, Milligan PJM, Pinder M, Vigneron L, Alloueche A, Kester KE et al. Efficacy of RTS,S/AS02 Malaria Vaccine against *Plasmodium falciparum* Infection in Semi-Immune Adult Men in the Gambia: A Randomized Trial. *The Lancet*. 2001; 358: 1927-34.

Bojang K, Olodude F, Pinder M, Ofori-Anyinam O, Vigneron L et al. Phase I safety and immunogenicity of RTS,S/AS02A in children 1 to 10 years of age in The Gambia. *Vaccine*. 2005; 23(32): 4148-57.

Bonhoeffer J, Menkes J, Gold, MS, de Souza-Brito G, Fisher MC et al. Generalized convulsive seizure as an adverse event following immunization: case definition and guidelines for data collection, analysis and presentation. *Vaccine*. 2004; 22: 557-562.

Breman JG, Egan A, Keusch GT. Introduction and Summary. The Intolerable Burden of Malaria: A New Look at the Numbers. *American Journal of Tropical Medicine and Hygiene*. 2001 a; 64 (1, 2) S: iv-vii.

Breman, JG. The Ears of the Hippopotamus: Manifestations, Determinants and Estimates of the Malaria Burden. *American Journal of Tropical Medicine and Hygiene* 2001 b; 64: 1-11.

Costy-Berger F. Preventive rabies vaccination using vaccine prepared from human diploid cells. *Dev Biol Stand* 1978; 40: 101-104.

Cox JH, Schneider LG. Prophylactic immunization of humans against rabies by intradermal inoculation of human diploid cell culture vaccine. *J Clin Microbiol* 1976; 3 (2): 96-101.

Epstein JE, Charoenvit Y, Kester KE, Wang R, Newcomer R, Fitzpatrick S et al. Safety, tolerability, and antibody responses in humans after sequential immunization with a PfCSP DNA vaccine followed by the recombinant protein vaccine RTS,S/AS02A. *Vaccine.* 2004; 22: 1592–1603.

Enosse S, Donana C, Quelhas D, Aponte JJ, Lievens M et al. RTS,S/AS02A Malaria Vaccine Does Not Induce Parasite CSP T Cell Epitope Selection and Reduces Multiplicity of Infection. *PLoS Clinical Trials.* 2006; 1(1): e5.

Gallup JL, Sachs JD. The Economic Burden of Malaria. *American Journal of Tropical Medicine and Hygiene*. 2001; 64: 85-96.

GSK data on file: Investigator Brochure GlaxoSmithKline Biologicals' Candidate Vaccines Against *Plasmodium falciparum* Malaria (RTS,S/AS02A, RTS,S/AS02D and RTS,S/AS01B Malaria Vaccines). 23 May 2005. Data on File at GlaxoSmithKline Biologicals.

GSK data on file: Safety, Immunogenicity and Efficacy of Three Adjuvant Preparations of a *Plasmodium falciparum* Vaccine (RTS,S) Which Utilizes the Hepatitis B Surface Antigen (HBsAg) as a Carrier for the Malaria Circumsporozoite (CS) Antigen. Report WRMAL-003. 24 August 1996. Data on File at GlaxoSmithKline Biologicals.

GSK data on file: Safety, Immunogenicity, and Preliminary Efficacy of a Lyophilized Formulation of SmithKline Beecham Biologicals' Candidate Malaria Vaccine RTS,S. Report WRMAL-005. 19 August 1999. Data on File at GlaxoSmithKline Biologicals.

GSK data on file: Dosage and Schedule Optimization of RTS,S Malaria Vaccine. Report WRMAL-004. 30 April 1998. Data on File at GlaxoSmithKline Biologicals.

GSK data on file: Phase IIb controlled randomized double-blind study to evaluate the efficacy, safety and immunogenicity of SmithKline Beecham Biologicals' candidate RTS,S malaria vaccine in semi-immune adult males in The Gambia. Report 257049/005 (Malaria-005) 15 February 2006. Data on File at GlaxoSmithKline Biologicals.

GSK data on file: Phase I/IIa safety, immunogenicity, and preliminary efficacy of two short-course schedules of SmithKline Beecham Biologicals' candidate malaria vaccine RTS,S in non-immune adult volunteers. Report 257049/012 (Malaria-012) 01 March 2002. Data on File at GlaxoSmithKline Biologicals.

GSK data on file: A Phase I Double Blind, Randomized, Controlled, Staggered Study to Evaluate the Safety, Reactogenicity and Immunogenicity of 1/5, 1/2 and Full Doses of GlaxoSmithKline Biologicals' RTS,S/AS02 Candidate Malaria Vaccine, Administered IM According to a 0, 1, 3-month Vaccination Schedule in Semi-Immune Children aged 6 to 11 Years in The Gambia, a Malaria Endemic Region. Report 257049/015 (Malaria-015) 14 October 2003. Data on File at GlaxoSmithKline Biologicals.

GSK data on file: Phase II b controlled randomized double-blind study to evaluate the efficacy, safety and immunogenicity of GlaxoSmithKline Biologicals' candidate RTS,S malaria vaccine in semi-immune adult males in The Gambia. Report 257049/016, 257049/017 & 257049/018 (Malaria-016, -017 & -018) 12 January 2005. Data on File at GlaxoSmithKline Biologicals.

GSK data on file: A Phase I Double Blind, Randomized, Controlled, Staggered Study to Evaluate the Safety, Reactogenicity and Immunogenicity of 1/5, 1/2 and Full Doses of

GlaxoSmithKline Biologicals' RTS,S/AS02 Candidate Malaria Vaccine, Administered IM According to a 0, 1, 3-month Vaccination Schedule in Semi-Immune Children aged 1 to 5 Years in The Gambia, a Malaria Endemic Region. Report 257049/020 (Malaria-020) 27 February 2003. Data on File at GlaxoSmithKline Biologicals.

GSK data on file: A Phase I Double Blind, Randomized, Controlled, Staggered Study to Evaluate the Safety, Reactogenicity and Immunogenicity of GlaxoSmithKline Biologicals' RTS,S/AS02 Candidate Malaria Vaccine, Administered IM According to a 0, 1, 2-month Vaccination Schedule in Children aged 1 through 4 years in Mozambique, a Malaria Endemic Region. Report 257049/025 (Malaria-025) 10 October 2003. Data on File at GlaxoSmithKline Biologicals.

GSK data on file: A double-blind randomised controlled phase IIb study to evaluate the safety, immunogenicity and efficacy of GlaxoSmithKline Biologicals' candidate malaria vaccine RTS,S/AS02A, administered IM according to a 0, 1 and 2 month vaccination schedule in toddlers and children aged 1 to 4 years in a malaria-endemic region of Mozambique. Report 257049/026 (Malaria-026) 19 November 2004. Data on File at GlaxoSmithKline Biologicals.

GSK data on file: Double-blind, randomized phase I/IIa human challenge study, to evaluate the safety, reactogenicity, immunogenicity, and preliminary efficacy after primary sporozoite challenge and rechallenge, of GSK Biologicals' candidate malaria vaccines containing the antigen RTS,S adjuvanted with either AS02A or AS01B and administered intramuscularly at months 0, 1, 2 in healthy malaria-naive volunteers aged 18 - 45 years. Protocol 257049/027 (Malaria-027) 24 July 2003. Data on File at GlaxoSmithKline Biologicals.

GSK data on file: Double-blind, randomized phase I/IIa human challenge study, to evaluate the safety, reactogenicity, immunogenicity, and preliminary efficacy after primary sporozoite challenge and rechallenge, of GSK Biologicals' candidate malaria vaccines containing the antigen RTS,S adjuvanted with either AS02A or AS01B and administered intramuscularly at months 0, 1, 2 in healthy malaria-naive volunteers aged 18 - 45 years. Report 257049/027 (Malaria-027) 01 February 2006. Data on File at GlaxoSmithKline Biologicals.

GSK data on file: A Phase I/II randomized double-blind bridging study to evaluate the safety and immunogenicity of GlaxoSmithKline Biologicals' candidate vaccines RTS,S/AS02D (0.5 mL dose) and RTS,S/AS02A (0.25 mL dose) administered IM according to a 0, 1, 2 month vaccination schedule in children aged 3 to 5 years living in a malaria-endemic region of Mozambique. Report 257049/034 (Malaria-034) 06 December 2004. Data on File at GlaxoSmithKline Biologicals.

GSK data on file: A Phase I/IIb randomized, double-blind, controlled study of the safety, immunogenicity and proof-of-concept of RTS,S/AS02D, a candidate malaria vaccine in infants living in a malaria-endemic region. Protocol 103967 (Malaria-038) 05 July 2005. Data on File at GlaxoSmithKline Biologicals.

GSK data on file: A Phase IIb randomized, double-blind, controlled study of the safety, immunogenicity and proof-of-concept of RTS,S/AS02D, a candidate malaria vaccine,

when incorporated into an Expanded Program on Immunization (EPI) regimen that includes DTPw/Hib in infants living in a malaria-endemic region. Protocol 104298 (Malaria-040) 19 October 2005. Data on file at GlaxoSmithKline Biologicals.

GSK data on file: A Phase IIb randomized, double-blind, controlled study of the safety, immunogenicity and proof-of-concept of RTS,S/AS02A, and RTS,S/AS01B, two candidate malaria vaccines in malaria-experienced adults living in Western Kenya. Protocol 104743 (Malaria-044) 26 July 2005. Data on File at GlaxoSmithKline Biologicals.

GSK data on file: A Phase II randomized, double-blind bridging study of the safety and immunogenicity of GlaxoSmithKline Biologicals' candidate *Plasmodium falciparum* malaria vaccine RTS,S/AS01E (0.5 mL dose) to RTS,S/AS02D (0.5 mL dose) administered IM according to a 0, 1, 2-month vaccination schedule in children aged 18 months to 4 years living in Gabon. Protocol 105874 (Malaria-046) 16 February 2006. Data on File at GlaxoSmithKline Biologicals.

GSK data on file: A Phase II randomized, controlled, partially-blind study of the safety and immunogenicity of GlaxoSmithKline Biologicals' candidate *Plasmodium falciparum* vaccines RTS,S/AS02D and RTS,S/AS01E, when administered IM according to one of three dose schedules in children aged 5 to 17 months living in Ghana. Protocol 106357 (Malaria-047) 13 February 2006. Data on File at GlaxoSmithKline Biologicals.

Kester KE, McKinney DA, Tornieporth N, Ockenhouse CF, Heppner DG, et al. Efficacy of recombinant circumsporozoite protein vaccine regimens against experimental *Plasmodium falciparum* malaria. *Journal of Infectious Diseases*. 2001; 15:183(4):640-7.

Kuwert EK, Marcus I, Werner J, Iwand A, Thraenhart O. Some experiences with human diploid cell strain-(HDCS) rabies vaccine in pre- and post-exposure vaccinated humans. *Dev Biol Stand* 1978; 40: 79-88.

Lalvani A, Moris P, Voss G, Kester KE, Brookes R, Koutsoukos M. Potent induction of focused Th-1 type cellular and humoral immune responses by RTS,S/SBAS2 recombinant *P. falciparum* malaria vaccine. *Journal of Infectious Diseases*. 1999; 180:1656-64.

Mbogo C, Mwangangi JM, Nzovu J, Gu W, Yan G et al. Spatial and temporal heterogeneity of *Anopheles* mosquitoes and *Plasmodium falciparum* transmission along the Kenyan coast. *American Journal of Tropical Medicine and Hygiene* 2003; 68 (6): 734-742.

Mwangi TW, Ross A, Snow RW, Marsh K. Case Definitions of Clinical Malaria under Different Transmission Conditions in Kilifi District, Kenya. *Journal of Infectious Disease.* 2005; 191: 1932-9.

Pinder M, Reece WH, Plebanski M et al. Cellular immunity induced by the recombinant *Plasmodium falciparum* malaria vaccine, RTS,S/AS02, in semi-immune adults in The Gambia. *Clin Exptl Immunol* 2004; 135 (2): 286-293.

Reece WH, Pinder M, Gothard PK et al. A CD4+ T-cell immune response to a conserved epitope in the circumsporozoite protein correlates with protection from natural *Plasmodium falciparum* infection and disease. *Nature Medicine*. 2004; 10 (4): 406-410.

Smith PG, Morrow RH. *Methods for Field Trials of Interventions against Tropical Diseases: A Toolbox*. Oxford University Press 1991: 152-155; 168-175.

Snow RW, Schellenberg, JR, Peshu, D, Forster, CR, Newton, PA et al. Periodicity and space-time clustering of severe childhood malaria on the coast of Kenya. *Trans. R. Soc. Trop. Med. Hyg*. 1990: 87 (4): 386-390.

Snow RW, de Azevedo Bastos, I, Lowe, EW, Kabiru, CG, Nevill, CG et al. Severe childhood malaria in two areas of markedly different falciparum transmission in east Africa. *Acta Trop*. 1994: 57 (4): 289-300.

Stoute JA, Slaoui M, Heppner DG, Momin P, Kester KE, Desmons P et al. A Preliminary Evaluation of a Recombinant Circumsporozoite Protein Vaccine against *Plasmodium falciparum* Malaria. *N Eng J Med* 1997; 336: 86-91

Sun P, Schwenk R, White K, Stoute JA, Cohen J, Ballou WR et al. Protective immunity induced with malaria vaccine, RTS,S, is linked to *Plasmodium falciparum* circumsporozoite protein-specific CD4(+) and CD8(+) T cells producing IFN-gamma. *Journal of Immunology*. 2003; 171 (12): 6961-7.

UNAIDS/WHO. Epidemiological Fact Sheet on HIV/AIDS and Sexually Transmitted Infections. Kenya. 2004 Update. New York: UNAIDS/WHO 2004a.

UNAIDS/WHO. Epidemiological Fact Sheet on HIV/AIDS and Sexually Transmitted Infections. Tanzania. 2004 Update. New York: UNAIDS/WHO 2004b.

Vryheid RE, Kane MA, Muller N, Schatz GC, Bezabeh S. Infant and Adolescent Hepatitis B Immunization up to 1999: A Global Overview. *Vaccine* 2001;19: 1026-1037.

World Health Organization. Hepatitis B Fact Sheet WHO/204. http://www.who.int/mediacentre/factsheets/fs204/en/index.html Accessed 02 February 2006.

World Health Organization. Draft Global Immunization Strategy, 28 April 2005. http://www.who.int/vaccines/GIVS/english/full_en.pdf. Accessed 02 February 2006.

Appendix A World Medical Association Declaration of Helsinki

Recommendations guiding physicians in biomedical research involving human subjects

Adopted by the 18th World Medical Assembly
Helsinki, Finland, June 1964

and amended by the
29th World Medical Assembly
Tokyo, Japan, October 1975
35th World Medical Assembly
Venice, Italy, October 1983
41st World Medical Assembly
Hong Kong, September 1989
and the
48th General Assembly
Somerset West, Republic of South Africa, October 1996

INTRODUCTION

It is the mission of the physician to safeguard the health of the people. His or her knowledge and conscience are dedicated to the fulfillment of this mission.

The Declaration of Geneva of the World Medical Association binds the physician with the words, "The health of my patient will be my first consideration," and the International Code of Medical Ethics declares that, "A physician shall act only in the patient's interest when providing medical care which might have the effect of weakening the physical and mental condition of the patient."

The purpose of biomedical research involving human subjects must be to improve diagnostic, therapeutic and prophylactic procedures and the understanding of the etiology and pathogenesis of disease.

In current medical practice most diagnostic, therapeutic or prophylactic procedures involve hazards. This applies especially to biomedical research.

Medical progress is based on research which ultimately must rest in part on experimentation involving human subjects.

In the field of biomedical research a fundamental distinction must be recognized between medical research in which the aim is essentially diagnostic or therapeutic for a patient, and medical research, the essential object of which is purely scientific and without implying direct diagnostic or therapeutic value to the person subjected to the research.

Special caution must be exercised in the conduct of research which may affect the environment, and the welfare of animals used for research must be respected.

63f195821c4e0e87c982cb189f3de2c4

Because it is essential that the results of laboratory experiments be applied to human beings to further scientific knowledge and to help suffering humanity, the World Medical Association has prepared the following recommendations as a guide to every physician in biomedical research involving human subjects. They should be kept under review in the future. It must be stressed that the standards as drafted are only a guide to physicians all over the world. Physicians are not relieved from criminal, civil and ethical responsibilities under the laws of their own countries.

I. BASIC PRINCIPLES

1. Biomedical research involving human subjects must conform to generally accepted scientific principles and should be based on adequately performed laboratory and animal experimentation and on a thorough knowledge of the scientific literature.
2. The design and performance of each experimental procedure involving human subjects should be clearly formulated in an experimental protocol which should be transmitted for consideration, comment and guidance to a specially appointed committee independent of the investigator and the sponsor provided that this independent committee is in conformity with the laws and regulations of the country in which the research experiment is performed.
3. Biomedical research involving human subjects should be conducted only by scientifically qualified persons and under the supervision of a clinically competent medical person. The responsibility for the human subject must always rest with a medically qualified person and never rest on the subject of research, even though the subject has given his or her consent.
4. Biomedical research involving human subjects cannot legitimately be carried out unless the importance of the objective is in proportion to the inherent risk to the subject.
5. Every biomedical research project involving human subjects should be preceded by careful assessment of predictable risks in comparison with foreseeable benefits to the subject or to others. Concern for the interests of the subject must always prevail over the interests of science and society.
6. The right of the research subject to safeguard his or her integrity must always be respected. Every precaution should be taken to respect the privacy of the subject and to minimize the impact of the study on the subject's physical and mental integrity and on the personality of the subject.
7. Physicians should abstain from engaging in research projects involving human subjects unless they are satisfied that the hazards involved are believed to be predictable. Physicians should cease any investigation if the hazards are found to outweigh the potential benefits.
8. In publication of the results of his or her research, the physician is obliged to preserve the accuracy of the results. Reports of experimentation not in accordance with the principles laid down in this Declaration should not be accepted for publication.
9. In any research on human beings, each potential subject must be adequately informed of the aims, methods, anticipated benefits and potential hazards of the study and the discomfort it may entail. He or she should be informed that he or she is at liberty to

abstain from participation in the study and that he or she is free to withdraw his or her consent to participation at any time. The physician should then obtain the subject's freely-given informed consent, preferably in writing.

10. When obtaining informed consent for the research project the physician should be particularly cautious if the subject is in a dependent relationship to him or her or may consent under duress. In that case the informed consent should be obtained by a physician who is not engaged in the investigation and who is completely independent of this official relationship.

11. In case of legal incompetence, informed consent should be obtained from the legal guardian in accordance with national legislation. Where physical or mental incapacity makes it impossible to obtain informed consent, or when the subject is a minor, permission from the responsible relative replaces that of the subject in accordance with national legislation.
    Whenever the minor child is in fact able to give a consent, the minor's consent must be obtained in addition to the consent of the minor's legal guardian.

12. The research protocol should always contain a statement of the ethical considerations involved and should indicate that the principles enunciated in the present Declaration are complied with.

II. MEDICAL RESEARCH COMBINED WITH PROFESSIONAL CARE (Clinical research)

1. In the treatment of the sick person, the physician must be free to use a new diagnostic and therapeutic measure, if in his or her judgment it offers hope of saving life, re-establishing health or alleviating suffering.
2. The potential benefits, hazards and discomfort of a new method should be weighed against the advantages of the best current diagnostic and therapeutic methods.
3. In any medical study, every patient - including those of a control group, if any - should be assured of the best proven diagnostic and therapeutic method. This does not exclude the use of inert placebo in studies where no proven diagnostic or therapeutic method exists.
4. The refusal of the patient to participate in a study must never interfere with the physician–patient relationship.
5. If the physician considers it essential not to obtain informed consent, the specific reasons for this proposal should be stated in the experimental protocol for transmission to the independent committee (I, 2).
6. The Physician can combine medical research with professional care, the objective being the acquisition of new medical knowledge, only to the extent that medical research is justified by its potential diagnostic or therapeutic value for the patient.

III. NON-THERAPEUTIC BIOMEDICAL RESEARCH INVOLVING HUMAN SUBJECTS (Non-clinical biomedical research)

1. In the purely scientific application of medical research carried out on a human being, it is the duty of the physician to remain the protector of the life and health of that person on whom biomedical research is being carried out.

2. The subjects should be volunteers - either healthy persons or patients for whom the experimental design is not related to the patient's illness.
3. The investigator or the investigating team should discontinue the research if in his/her or their judgment it may, if continued, be harmful to the individual.

In research on man, the interest of science and society should never take precedence over considerations related to the well being of the subject.

63f195821c4e0e87c982cb189f3de2c4

Appendix B Administrative Matters

I. Responsibilities of the Investigator

- To ensure that he/she has sufficient time to conduct and complete the study and has adequate staff and appropriate facilities and equipment which are available for the duration of the study and to ensure that other studies do not divert essential subjects or facilities away from the study at hand.
- To submit an up-to-date curriculum vitae or Investigator Biography and other credentials (e.g., medical license number in the United States) to GSK and—where required—to relevant authorities. It is recommended that this documentation indicates any previous clinical research experience and history of training in GCP.
- To acquire the reference ranges for laboratory tests performed locally and, if required by local regulations, obtain the laboratory’s current certification or Quality Assurance procedure manual.
- To ensure that no clinical samples (including serum samples) are retained on site or elsewhere without the approval of GSK Biologicals and the express written informed consent of the subject and/or the subject’s legally authorized representative.
- To perform no other biological assays at the investigator site except those described in the protocol or its amendment(s).
- To prepare and maintain adequate subject source data or raw data designed to record observations, and other data pertinent to the study.
- To conduct the study in compliance with the protocol any amendment and “Good Clinical Practice” (GCP) and all applicable regulatory requirements.
- To co-operate with a representative of GSK Biologicals in the monitoring process of the study and in resolution of queries about the data.
- To permit drug regulatory agencies and GSK audits.

II. Protocol Amendments and Administrative changes

- No changes to the study protocol will be allowed unless discussed in detail with the GSK Biologicals' Clinical Development Manager/Medical Monitor and filed as an amendment/administrative change to this protocol.
- Any amendment/administrative change to the protocol will be adhered to by the participating centre(s) and will apply to all subjects. Written IRB/IEC approval of protocol amendments is required prior to implementation, except where permitted by all applicable regulatory requirements; administrative changes and amendments not submitted for approval are submitted to IRBs/IECs for information only.
- Submission of protocol amendments to regulatory agencies will occur in accordance with local regulatory requirements. For some countries, submission to the local regulatory authority may not be required. When submission to the local regulatory authority is required, the timing of the submission relative to IEC/IRB submission or approval and whether or not the authority will provide their approval of or

favourable opinion on the amendment before it can be implemented will depend on local regulatory requirements.

III. Sponsor's Termination of Study

GSK Biologicals reserves the right to temporarily suspend or prematurely discontinue this study either at a single site or at all sites at any time for reasons including, but not limited to, safety or ethical issues or severe non-compliance. Reasons for suspension or early termination will be documented in the study file at GSK Biologicals.

If GSK Biologicals determines that suspension or early termination is needed, GSK Biologicals will discuss this with the Investigator (including the reasons for taking such action). When feasible, GSK Biologicals will provide advance notification to the investigator of the impending action prior to it taking effect.

GSK Biologicals will promptly inform, via written communication, all investigators and/or institutions conducting the study, if the study is suspended or terminated for safety reasons, and will also inform the regulatory authorities of the suspension or termination of the study and the reason(s) for the action. If required by applicable regulations, the investigator must inform the IEC/IRB promptly and provide the reason for the suspension or termination.

If the study is prematurely discontinued, all study data must be returned to GSK. In addition, arrangements will be made for all unused investigational product(s) in accordance with the applicable GSK procedures for the study. Financial compensation to investigators and/or institutions will be in accordance with the agreement established between the investigator and/or institutions and GSK.

IV. Case Report Form Instructions

Prior to screening the first potential participant, the investigator will provide the Site Monitor with a list (Site Staff Signature Sheet) showing the name and title, signature and initials of all site staff who have a critical effect on the conduct of the study and to whom the investigator has delegated significant study related duties such as entering data on the CRFs or changing entries on CRFs. If the authorized individuals should change during the study, the investigator is to inform GSK Biologicals GSK Biologicals' representative of the specific change(s).

CRFs (and subject diary cards, if applicable), will be supplied by GSK Biologicals for recording all data. It is the responsibility of the investigator or co-investigator to ensure that study data are legible, accurate, adequately recorded and, when entered on paper copy, completely filled in with a black ink fountain or ballpoint pen.

Errors must be corrected by drawing a single line through the incorrect entry and writing in the new value/data positioned as close to the original as possible. The correction must then be initialed and dated (and justified, whenever possible), where necessary, by the authorized individual making the change. The original entry must not be obliterated, overwritten or erased when a correction is made.

When a subject completes a visit, it is anticipated that relevant sections of the CRF will be completed by the investigator (or designated staff as documented in the Site Staff Signature Sheet) as soon as possible after the last data becoming available. Similarly, when a subject completes a study, it is anticipated that all relevant CRF pages will be completed promptly after the last data becoming available. This also applies to forms for potential study participants who were screened but not randomized to a study group.

As soon as the subject has completed/withdrawn from the study and the CRF is completed, the investigator or medically qualified sub-investigator to whom this task has been delegated will sign the study conclusion pages of the CRF to confirm that they have reviewed the data and that the data are complete and accurate. In all cases the investigator remains accountable for the study data collected.

An original (top copy) CRF or log sheets must be submitted for all subjects who have undergone protocol specific procedures, whether or not the subject completed the study.

While completed CRFs are reviewed by a GSK Biologicals' professional monitor at the study site, errors detected by subsequent in-house CRF review may necessitate clarification or correction of errors with documentation and approval by the investigator or appropriately qualified designee as documented on the Site Staff Signature Sheet. In all cases, the investigator remains accountable for the study data. Wherever possible the investigator should assist in the clarification or correction of errors detected after study finalization promptly after being brought to the attention of the investigator (preferably within 5 working days).

Any questions or comments related to the CRF should be directed to the assigned Site Monitor.

V. Monitoring by GSK Biologicals

To ensure compliance with the protocol, monitoring visits by a professional representative of the sponsor will be scheduled to take place early in the study, during the study at appropriate intervals and after the last subject has completed the study. It is anticipated that monitoring visits will occur at a frequency defined and communicated to the investigator before study start.

These visits are for the purpose of confirming that GSK Biologicals' sponsored studies are being conducted in accordance with the ethical principles that have their origins in the Declaration of Helsinki and that are consistent with Good Clinical practice (GCP) and the applicable regulatory requirement(s) (verifying continuing compliance with the protocol, amendment(s), reviewing the investigational product accountability records, verifying that the site staff and facilities continue to be adequate to conduct the study. Direct access to all study-related site and source data/ documents is mandatory for the purpose of monitoring review. The monitor will perform a CRF review and a Source Document verification (verifying CRF/ RDE entries by comparing them with the source data/documents that will be made available by the investigator for this purpose: any data item for which the CRF will serve as the source must be identified, agreed and documented. Data to be recorded directly into the CRF pages/RDE screens will be specified in writing preferably in the source documentation agreement form that is

contained in both the monitor's and investigator's study file. For RDE, the monitor will mark completed and approved screens at each visit. The investigator must ensure provision of reasonable time, space and adequate qualified personnel for monitoring visits. Source data verification (SDV) must be conducted using a GSK standard SDV sampling strategy (as defined at the study start in the study specific monitoring guidelines) in which monitors will perform partial SDV for all subjects and full SDV for selected subjects.

VI. Archiving of Data

Following closure of the study, the investigator must maintain all site study records in a safe and secure location. The records must be maintained to allow easy and timely retrieval, when needed (e.g., audit or inspection), and, whenever feasible, to allow any subsequent review of data in conjunction with assessment of the facility, supporting systems, and staff. Where permitted by applicable laws/regulations or institutional policy, some or all of these records can be maintained in a validated format other than hard copy (e.g., microfiche, scanned, electronic for studies with an eCRF, for example); however, caution needs to be exercised before such action is taken. The investigator must assure that all reproductions are legible and are a true and accurate copy of the original, and meet accessibility and retrieval standards, including re-generating a hard copy, if required. Furthermore, the investigator must ensure there is an acceptable back-up of these reproductions and that an acceptable quality control process exists for making these reproductions.

GSK will inform the investigator/ institution of the time period for retaining these records to comply with all applicable regulatory requirements. However, the investigator/ institution should seek the written approval of the sponsor before proceeding with the disposal of these records. The minimum retention time will meet the strictest standard applicable to that site for the study, as dictated by ICH GCP E6 Section 4.9, any institutional requirements or applicable laws or regulations, or GSK standards/procedures; otherwise, the minimum retention period will default to 15 years.

The investigator/ institution must notify GSK of any changes in the archival arrangements, including, but not limited to, the following: archival at an off-site facility, transfer of ownership of the records in the event the investigator leaves the site.

VII. Audits

For the purpose of compliance with Good Clinical Practice and Regulatory Agency Guidelines it may be necessary for GSK or a Drug Regulatory Agency to conduct a site audit. This may occur at any time from start to after conclusion of the study.

When an investigator signs the protocol, he agrees to permit drug regulatory agencies and GSK audits, providing direct access to source data/ documents. Furthermore, if an investigator refuses an inspection, his data will not be accepted in support of a New Drug Registration and/or Application, Biologics Licensing Application.

Having the highest quality data and studies are essential aspects of vaccine development. GSK has a Regulatory Compliance staff who audit investigational sites. Regulatory

Compliance assesses the quality of data with regard to accuracy, adequacy and consistency. In addition, Regulatory Compliance assures that GSK Biologicals' sponsored studies are in accordance with GCP and that relevant regulations/guidelines are being followed.

To accomplish these functions, Regulatory Compliance selects investigational sites to audit. These audits usually take 1 to 2 days. GSK's audits entail review of source documents supporting the adequacy and accuracy of CRFs, review of documentation required to be maintained, and checks on vaccine accountability. GSK's audit therefore helps prepare an investigator for a possible regulatory agency inspection as well as assuring GSK Biologicals of the validity of the database across investigational sites.

The Inspector will be especially interested in the following items:

- Log of visits from the sponsor's representatives
- Study personnel
- Study file
- Safety reporting
- IRB/IEC and regulatory authority approvals
- Facilities
- monitoring
- Vaccine accountability
- Approved study protocol and amendments and investigator brochure
- Informed consent of the subjects (written consent [or witnessed oral if applicable])
- Medical records and other source documents supportive of CRF data
- Reports to the IRB/IEC and the sponsor
- Record retention.

GSK Biologicals will gladly help investigators prepare for an inspection.

VIII. Ownership, Confidentiality and Publication

Ownership:

All information provided by GSK and all data and information generated by the site as part of the study (other than a subject's medical records) are the sole property of GSK.

All rights, title, and interests in any inventions, know-how or other intellectual or industrial property rights which are conceived or reduced to practice by site staff during the course of or as a result of the study are the sole property of GSK, and are hereby assigned to GSK.

If a written contract for the conduct of the study which includes ownership provisions inconsistent with this statement is executed between GSK and the study site, that contract's ownership provisions shall apply rather than this statement.

Confidentiality:

Documented evidence that a potential investigator is aware and agrees to the confidential nature of the information related to the study must be obtained by means of a confidentiality agreement.

All information provided by GSK and all data and information generated by the site as part of the study (other than a subject's medical records) will be kept confidential by the investigator and other site staff. This information and data will not be used by the investigator or other site personnel for any purpose other than conducting the study. These restrictions do not apply to: (1) information which becomes publicly available through no fault of the investigator or site staff; (2) information which it is necessary to disclose in confidence to an IEC or IRB solely for the evaluation of the study; (3) information which it is necessary to disclose in order to provide appropriate medical care to a study subject; or (4) study results which may be published as described in the next paragraph. If a written contract for the conduct of the study which includes confidentiality provisions inconsistent with this statement is executed, that contract's confidentiality provisions shall apply rather than this statement.

Publication:

For multicenter studies, the first publication or disclosure of study results shall be a complete, joint multicenter publication or disclosure coordinated by GSK. Thereafter, any secondary publications will reference the original publication(s).

Prior to submitting for publication, presentation, use for instructional purposes, or otherwise disclosing the study results generated by the site (collectively, a "Publication"), the investigator shall provide GSK with a copy of the proposed Publication and allow GSK a period to review the proposed Publication (at least 21 (twenty-one) days [or at least 15 (fifteen) working days for abstracts/posters/presentations]. Proposed Publications shall not include either GSK confidential information other than the study results or personal data on any subject, such as name or initials.

At GSK's request, the submission or other disclosure of a proposed Publication will be delayed a sufficient time to allow GSK to seek patent or similar protection of any inventions, know-how or other intellectual or industrial property rights disclosed in the proposed Publication.

If a written contract for the conduct of the study, which includes publication provisions inconsistent with this statement is executed, that contract's publication provisions shall apply rather than this statement.

Appendix C Overview of the Recruitment Plan

JMP, Korogwe

Prior to enrollment, the community will be informed about the trial. First the community leaders will be approached. Based on the advice of the community leaders the community will be informed. The need and the difficulties of developing a vaccine against malaria will be discussed in a culturally appropriate fashion, as well as an outline of the proposed trial, including the rationale, the background data available, the study objectives and the screening and consent procedures. Particular attention will be paid to study procedures, immunization and blood collection. To avoid misunderstandings the purpose of blood collection and the associated risks will be explained in a culturally sensitive fashion.

Following the village information campaign outlined in the paragraph above a list of children in the eligible age group will be compiled based on recent census data. Following community meetings and the recommendations of community leaders parents/guardians of children in the eligible age group will be invited to have their children participate in the trial. A list of children whose parents/guardians express an interest will be compiled. Study personnel will seek individual informed consent for each child from the parents/guardians in privacy. Parents/guardians will again be informed about the study objectives and procedures and they will be encouraged to ask and clarify questions about the trial. The parents/guardians comprehension of the trial will be verified. The parents/guardians and the witness (where parents/guardians are illiterate) will be invited to sign the Informed Consent Form. Children whose parents/guardians consent for them to enter the study will be invited to be screened according to the procedures outlined in the Study Protocol ***Amended 23 October 2006***.

KEMRI, Kilifi

A series of public meetings will be held to explain the study to the community. This will be held in public locations within the study area, and parents/guardians will be invited to attend by field workers. The local area chief, and dispensary committee will be invited to be present for these meetings. The need and the difficulties of developing a vaccine against malaria will be discussed in a culturally appropriate fashion, as well as an outline of the proposed trial, including the rationale, the background data available, the study objectives and the screening and consent procedures. Particular attention will be paid to study procedures, immunization and blood collection. To avoid misunderstandings the purpose of blood collection and the associated risks will be explained in a culturally sensitive fashion.

Potentially interested parents/guardians will be asked to register their names at the dispensary, or with field workers who live locally to them. Those who have registered their names will be invited to attend the dispensary for screening. Transport will be provided to facilitate attendance if necessary. At the dispensary, the full content of the information sheet will be discussed with the group, and an opportunity for public and then private questioning offered. Parents/guardians who consent for their children to take part in the trial will be asked to sign or thumbprint (witnessed where parents/guardians are illiterate) the Informed Consent Form.

63f195821c4e0e87c982cb189f3de2c4

Appendix D Handling of Biological Samples Collected by the Investigator

Instructions for Handling of Serum Samples

When materials are provided by GSK Biologicals, it is MANDATORY that all clinical samples (including serum samples) will be collected and stored using exclusively those materials in the appropriate manner. The use of other materials could result in the exclusion of the subject from the ATP analysis. The investigator must ensure that his/her personnel and the laboratory(ies) under his/her supervision comply with this requirement. However, when GSK Biologicals does not provide material for collecting and storing clinical samples, then appropriate materials from the investigator's site are to be used.

1. Collection

The whole blood should be collected observing appropriate aseptic conditions. It is recommended that Vacutainer® tubes WITH integrated serum separator (e.g. Becton-Dickinson Vacutainer SST or Corvac® Sherwood Medical) be used to minimize the risk of hemolysis and to avoid blood cell contamination of the serum when transferring to standard serum tubes.

2. Serum separation

These guidelines aim to ensure high quality serum by minimizing the risk of hemolysis, blood cell contamination of the serum or serum adverse cell toxicity at testing.

- For separation of serum using Vacutainer tubes, the instructions provided by the manufacturer should be followed. Siliconized tubes should never be used (cell toxicity). Often the manufacturer's instruction states that the relative centrifugal acceleration known also as "G" must be "between 1000 and 1300 G" with tubes spinning for ten minutes. Error in calculation of centrifuge speed can occur when laboratory personnel confuse "G" acceleration with "RPM" (revolutions per minute). The speed of centrifugation must be calculated using the "G" rate provided in the manufacturer's instructions and the radius of the centrifuge head. After measuring the radius of the centrifuge machine, a speed/acceleration nomograph must be employed to determine the centrifuge speed in "RPM".
- Following separation, the serum should be aseptically transferred to the appropriate standard tubes using a sterile disposable pipette. The serum should be transferred as gently as possible to avoid blood cell contamination.
- The tube should not be overfilled (max. 3/4 of the total volume) to allow room for expansion upon freezing.
- The tube should be identified by the appropriate label provided by GSK Biologicals (see point 3).

3. Labeling

- The standard labels provided by GSK Biologicals should be used to label each serum sample.

- If necessary, any hand-written additions to the labels should be made using indelible ink.
- The label should be attached to the tube as follows (see diagram):
  - first attach the paper part of the label to the tube
  - then wrap the label around the tube so that the transparent, plastic part of the label overlaps with the label text and bar code and shields them.

  This will ensure optimal label attachment.

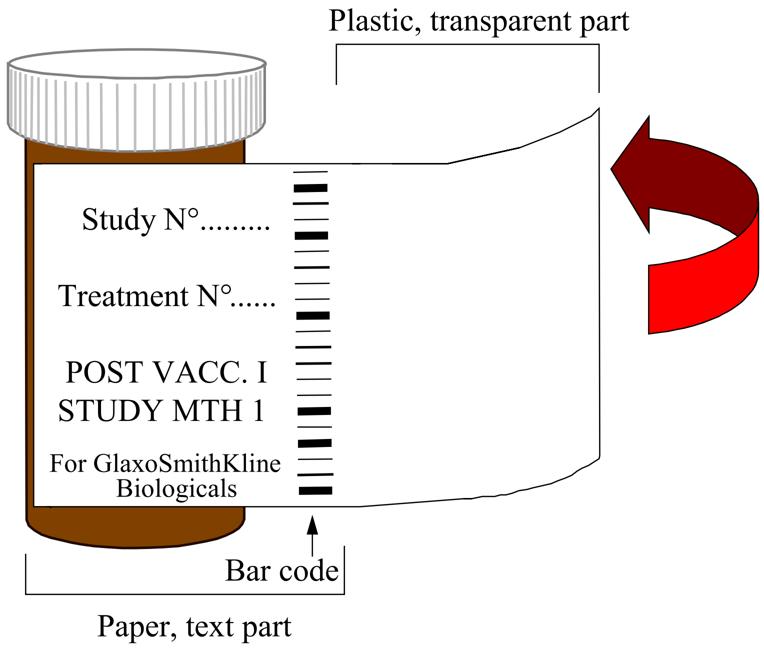

Labels should not be attached to caps.

4. Sorting and storage

- Tubes should be placed in the GSK Biologicals' cardboard boxes in numerical order from left to right, starting from the lower left hand corner, beginning with the pre-vaccination samples series, then with the post-vaccination sample series.
- The tubes of serum should be stored in a vertical position at approximately -20°C (alternatively at approximately -70°/80°C is also acceptable) until shipment to GSK Biologicals. The storage temperature should be checked regularly and documented. Wherever possible, a backup facility for storage of serum samples should be available.
- A standard Serum Listing Form, specifying the samples being shipped for individual subjects at each time point, should be prepared for each shipment. A copy of this list should be retained at the study site, while the original should be sealed in a plastic envelope and shipped with the serum samples.

- Once flight details are known, a standard Specimen Transfer Form must be completed and faxed to GSK Biologicals to the number provided below. A copy of the Specimen Transfer Form must be in the parcel[1]

GLAXOSMITHKLINE BIOLIGICALS
Attention Biospecimen Reception
Clinical Immunology
R & D Department/Building 44
Rue de l'Institut, 89
B-1330 Rixensart – Belgium

Telephone	+32-2-656 8949 or +32-2-656 6130
	or +32-2-656 8549 or +32-2-656 6108
Fax	+32-2-656 6052
E-mail	rix.ugbiospecimen-reception@gskbio.com

Instructions for Handling Cells for Whole Blood Cell-Mediated Immunity Assay

When materials are provided by GSK Biologicals, it is mandatory that all clinical samples be collected and stored using exclusively those materials in the appropriate manner. The use of other materials could result in the exclusion of the subject from analysis. The investigator must ensure that his/her personnel and the laboratory(ies) under his/her supervision comply with this requirement.

1. Collection of whole blood

Collect blood by venipuncture in Terumo tubes with heparin (mandatory) and record time of collection. The tubes should be kept at constant room temperature and shipped as quickly as possible to a designated clinical site. Use well closed Styrofoam boxes of 5 cm thickness for blood samples transport (see current version of GSK Biologicals SOP RD_HCI_001 for guidance).

2. Labeling of tubes

- If labels are provided by GSK, it is mandatory to use them.
- If necessary, any hand-written additions to the labels should be made using indelible ink.

3. Sorting and storage of blood cell samples

Samples should be stored frozen (<-80°C) until shipment to GSK Biologicals. Wherever possible, a backup facility for storage of samples should be available.

---

[1] The Serum Listing Form and the Specimen Transfer Form are standard documents used in GSK Biologicals' clinical trials. These documents are provided by GSK Biologicals' Clinical Trials' monitor at study initiation.

A standard Cryotube Listing Form (see current version of GSK Biologicals SOP RD_HCI_009 for guidance), specifying the samples being shipped for individual subjects at each timepoint, should be prepared for each shipment. A copy of this list should be retained at the study site, while the original should be sealed in a plastic envelope and shipped with the samples.

Appendix E Shipment of Biological Samples

Instructions for shipment of serum and cell samples

Serum and cell samples should be sent to GSK Biologicals at regular intervals. The frequency of shipment of samples should be decided upon by the Site Monitor, Central Study Coordinator and the investigator prior to the study start.

Serum and cell samples should always be sent by air, preferably on a Monday, Tuesday or Wednesday, unless otherwise requested by the sponsor.

Serum and cell samples must be placed with dry ice (maximum -20°C) in a container complying with International Air Transport Association (IATA) requirements. The completed standard serum listing form should always accompany the shipment.

The container must be clearly identified with the labels provided by GSK Biologicals specifying the shipment address and the storage temperature (-20°C).

The airway bill should contain the instruction for storage of samples at maximum -20°C.

A ‘proforma’ invoice, stating a value for customs purposes only, should be prepared and attached to the container. This document should contain the instruction for storage of samples at maximum -20°C.

Details of the shipment, including:

* number of samples
* airway bill
* flight number
* flight departure and arrival times

should be sent by fax or email two days before shipment, to:

GLAXOSMITHKLINE BIOLOGICALS,
Attention Biospecimen Reception
Clinical Immunology
R & D Department/Building 44
Rue de l’Institut, 89
B-1330 Rixensart – Belgium

Telephone +32-2-656 8949 or +32-2-656 6130
or +32-2-656 8549 or +32-2-656 6108
Fax +32-2-656 6052
E-mail rix.ugbiospecimen-receiption@gskbio.com

The central study coordinator Jean François Stallaert and Clinical Research Associate (Ezekiel Oenga) should be informed 2 days before any shipment.

Jean François Stallaert, Rixensart, Belgium<br>Telephone: +32.2.656.***94.28***<br>Fax: +32.2.656.80.44<br>email: ***jean-francois.j.stallaert@gskbio.com***<br>Ezekiel Oenga, Nairobi, Kenya<br>Tel: +254 20 6933 322<br>Fax: +254 20 558 329<br>email: ezekiel.r.oenga@gsk.com	Jean François Stallaert, Rixensart, Belgium<br><br>Telephone: +32.2.656.94.28<br>Fax: +32.2.656.80.44<br>email: jean-francois.j.stallaert@gskbio.com

Amended 23 October 2006.
Amended 17 April 2008

Appendix F Laboratory Assays

Serology testing

Serological responses will be measured principally by evaluating antibody responses to HBs and to CSP repeats (anti R32LR). Serum for antibody determination will be collected at the time points defined in the flowcharts in protocol Section 5.14.

Antibody levels against CS will be measured at GSK Biologicals (or a designated laboratory) by standard ELISA methodology using plate adsorbed R32LR antigen with a standard reference antibody as a control according to SOPs from the laboratory. Results will be reported in EU/mL.

Antibody to hepatitis B surface antigen will be measured at GSK Biologicals using a commercially available ELISA immunoassay (AUSAB EIA test kit from Abbott) or equivalent according to the assay instructions. Results will be reported in mIU/mL.

Determination of parasitemia

Slides for the determination of parasitemia will be made and read in duplicate. Estimates of asexual *P. falciparum* parasite density will be made at the investigator's sites according to laboratory SOPs.

Biochemical and hematological analyses

Hematological and biochemical testing will be done at the investigator's sites, following laboratory SOPs.

Cell-mediated immunity

IntraCellular Cytokine Staining Assay

CMI will be investigated at four time points in this study, corresponding to screening, one month and one year post last dose of vaccination.

After collection, whole blood samples will be stimulated in vitro with CS-derived peptide for 2 hours, then cytokine secretion inhibitor (brefeldin A) will be added for an additional overnight incubation. The day after, the cells will be washed and fixed before being frozen and and kept in storage for subsequent cytometry analysis.

Intracellular fluorescent staining for cytokines (ICS) is a method for detecting defined populations of cells (for example CD4+ or CD8+ T-cells expressing cytokines and activation markers upon in vitro stimulation). These analyses will allow the determination of the frequencies of Ag-specific cells and determine the functionalities of these cells.

The main assay (secondary and tertiary endpoints) will look at expression of IL-2, IFN-γ, TNF-α and CD40-L among cell populations defined by CD4 and CD8 staining.

Other markers may be used for exploratory analysis and further definition of the quality of the induced immune response (not secondary endpoint).

63f195821c4e0e87c982cb189f3de2c4

IFN-gamma producing CS-specific short term effectors and central memory T-cells (Elispot assays)

This assay will be conducted in Kilifi, Kenya, in samples from children enrolled there, collected at screening, one month and one year post Dose 3, when cell numbers allow.

Briefly, the cellular pellet of heparinized blood will be resuspended in culture medium. PBMC will be separated by density gradient centrifugation. For the detection of short term effectors, an overnight incubation in the presence of a pool of overlapping peptides of the CS vaccine antigen in Elispot plates will allow the detection of IFN-γ producing cells. The presence of central memory T-cells is assessed in a cultured assay where PBMCs are stimulated with the antigen for 10 days in the presence of IL-2 after Day 3. After 10 days the cells are washed and left overnight in media alone. They are then plated out in various conditions in the morning: media alone (negative control), PHA (positive control), and antigenic peptides. IFN-γ producing cells are detected by a standard IFN-γ Elispot.

Regulatory T-cells

These investigations will be done on samples collected 4½ months post Dose 3.

Separated PBMC will be cultured in the presence of malaria antigens, media only (negative control) or mitogen (positive control). The expression of Regulatory T-cells markers (such as CD4, CD8, CD56, IFN-γ, CD25, Foxp3, IL-10) will be measured by FACS and RT-PCR.

Humoral response to blood-stage antigens

These investigations will be done on samples collected at screening, one month and one year post Dose 3. Heparinised blood will be centrifuged, plasma will be collected and stored at -80°C for analysis of the presence of IgG, IgG1 and IgG3 antibodies to whole parasite lysate (PfSE) and to defined, recombinant *P. falciparum* blood stage antigens by ELISA and/or protein microarray and/or bead-based multiplex assay. The antigens to be tested may include (but not necessarily be limited to) MSP-1, MSP-2, AMA-1, EBA-175, RAP-1, GPI, GLURP, MSP-3, MSP-6, MSP-7 and VSA.

Appendix G Vaccine supplies, packaging and accountability

1. Vaccine and/or other supplies

GSK Biologicals will supply the following study vaccines, sufficient number of doses to administer to all subjects as described in the present protocol.

- Sufficient doses for 445 recipients of the candidate vaccine RTS,S/AS01E (doses of RTS,S vaccine in monodose vials and doses of AS01E adjuvant in vials).
- Sufficient doses for 445 recipients of Sanofi-Pasteur's Human Diploid Cell Rabies Vaccine

Amended 20 December 2006

At least an additional 3% of their respective amounts of RTS,S/AS01E and Sanofi-Pasteur's Human Diploid Cell Rabies Vaccine will be supplied for replacement in case of breakage, bad storage conditions or any other reason that would make the vaccine unusable (i.e. given by mistake to another subject).

All monodose vials/pre-filled syringes must be accounted for on the form provided.

Labels for sample identification:
The investigator will receive labels from GSK Biologicals to identify samples taken from each subject at each timepoint. Each label will contain the following information: study number, identification number for the subject (e.g. **Subject number)**, sampling timepoint (e.g., post vacc 3), timing (e.g., study Month 7).

Other supplies provided by GSK Biologicals:
In addition to the vaccines, the study documentation and the sample labels, the investigator will receive the following supplies:

- tubes with screw caps for serum samples,
- racks and cardboard boxes for the tubes of serum.

The investigator or pharmacist must sign a statement that he/she has received the clinical supplies for the study.

It is NOT permitted to use any of the supplies provided by GSK Biologicals for purposes other than those specified in the protocol.

2. Vaccine packaging

The vaccines will be packed in labelled boxes. The box label will contain, as a minimum, the following information: study number, treatment number, lot number (or numbers, when double-blind), instructions for vaccine administration and any other relevant regulatory requirements.

3. Vaccine shipment from GSK Biologicals Rixensart to local country medical department, dispatching centre or investigational site from local country medical department to investigational site

On arrival of vaccine shipment, the cold-chain monitoring device should be removed from the vaccine boxes and checked. The temperature recording chart (chart from Cox recorder or print-out data of the electronic device) should be obtained from the temperature recording device.

The following documents should be completed and returned to GSK Biologicals on reception of vaccine shipment:

Notification of vaccine delivery/temperature control
Temperature recording (chart).

These documents should then be returned to:

Attention of Clinical Trials Supply Unit

Clinical Operations Logistics

GSK Biologicals Rixensart

Fax : +32 (0)2 656 75 17

E-mail: rix.ugCTSU@gskbio.com.

In case of any temperature deviation, the official written approval for the use of vaccine must be obtained from GSK.

4. Vaccine accountability

At all times the figures on supplied, used and remaining vaccine doses should match. At the end of the study, it must be possible to reconcile delivery records with those of used and unused stocks. An explanation must be given of any discrepancies.

After approval from GSK Biologicals and in accordance with GSK SOP WWD-1102, used and unused vaccine vials/syringes should be destroyed at the study site using locally approved biosafety procedures and documentation unless otherwise described in the protocol. If no adequate biosafety procedures are available at the study site, the used and unused vaccine vials/syringes are to be returned to an appropriate GSK Biologicals site for destruction, also in accordance with current GSK SOP WWD-1102.

5. Transfers of clinical vaccines or products from country medical department or dispatch centre to study sites or between sites

Storage temperatures must be maintained during transport and deviations must be reported to GSK for guidance. All transfers of clinical vaccines or products must be documented using the Clinical Supply Transfer Form.

All packaging and shipment procedures for transfer of clinical vaccines or products must follow procedures approved by the sponsor.

Clinical vaccines or products should always be sent by contract courier designated by the sponsor, unless otherwise requested by the sponsor.

Appendix H Composition of Data Safety Monitoring Board

For this trial, the Data Safety Monitoring Board will be composed of six members:

Prof. Malcolm Molyneux (Chairperson),

Malaria Project and Wellcome Trust Centre,
Box 30096, Blantyre 3,
Malawi.

Dr. Juanita Hatcher (Statistician),

Community Health Sciences Department,
Aga Khan University,
Stadium Road, PO Box 3500,
Karachi 74800,
Pakistan.

Dr. Umberto D'Allesandro (Co-chairperson),

Prince Leopold Institute of Tropical Medicine,
Department of Epidemiology,
Nationalestraat 155,
2000 Antwerp,
Belgium.

Dr. Tom Doherty (Secretary),

London School of Hygiene and Tropical Medicine,
Keppel Street,
London WC1E 7HT,
United Kingdom.

Dr. Kojo Koram,

Research Fellow and Ag. Head of Unit,
Noguchi Memorial Institute for Medical Research,
University of Ghana,
PO Box LG 581, Legon,
Ghana.

Prof. Zulfiqarali G Premji,

Professor of Infectious Diseases,
Department of Parasitology/Medical Entomology,
School of Public Health and Social Sciences,
Muhimbili University College of Health Sciences,
Box 65011, Dar es Salaam,
Tanzania.

Appendix I Amendments and Administrative Changes to the Protocol

GlaxoSmithKline Biologicals

Clinical Research & Development

Protocol Amendment Approval

**eTrack study number**	106367
**eTrack abbreviated title**	Malaria-049
**IND number**	BB-IND 12937
**Protocol title:**	A Phase IIb randomized, double-blind, controlled study of the efficacy against episodes of clinical malaria due to *Plasmodium falciparum* infection of GlaxoSmithKline Biologicals' candidate vaccine RTS,S/AS01E, administered IM according to a 0, 1, 2-month vaccination schedule in children aged 5 months to 17 months living in Tanzania and Kenya
**Amendment number:**	Amendment 1
**Amendment date:**	***23 October 2006***
**Co-ordinating author:**	Conor Cahill, Scientific Writer

Rationale/background for changes:

A number of changes have been made to the study at the request of the investigators and the study team at GSK Biologicals. These include:

The investigators at JMP have been informed that HAART may not be officially rolled out at Korogwe by the time of study start. However, if this is the case it will be ensured that any subjects who test positive for HIV during the study will have access to HAART at another facility.

The health center at Vipingo (Kenya) will not be used for out of hours consultation during this study.

To ensure a high level of access to care for all participants, they will be advised that they can seek care facilitated by a field worker in their village 24 hours a day, seven days a week.

Details regarding the provision of healthcare at both sites have been added and the role of the study physicians and clinicians have been clarified.

Additional blood sampling timepoints have been added at the end of the double-blind phase and the end of the trial to facilitate long-term safety surveillance.

Some errors/missions in the original version have been corrected:

The GSK Biologicals Clinical Safety Physcian contact has been added to the study contacts for an SAE occurring at JMP.

The name of the Clinical Research Associate for this study, based in Kenya, has been added

The instructions for Passive Case Detection of malaria have been clarified.

Due to the potential impact of Vitamin A on vaccine response and malaria infection, the administration of Vitamin A supplementation will be recorded as a concomitant medication.

Any incidence of residual insecticide spraying will be recorded for the purpose of covariate analysis.

Normal/acceptable ranges for the community in Kenya are now available. The acceptable/normal ranges for the study have been adapted to reflect these. Specifically the acceptable limit for ALT is now 75 and the acceptable limit for platelet count is now 75.

Blood samples taken from children will be analysed for blood stage immunity at JMP and KEMRI in addition to the laboratory at LSHTM as specified in the original protocol.

Case definitions for the trial have been added to the body of the study protocol; previously they were only provided in the study synopsis.

Amended text has been included in *bold italics* in the following sections, deletions indicated by strikethrough:

Synopsis:

7. Study Contacts for Reporting of a Serious Adverse Event occurring at JMP

All of the contacts listed below must be informed of each SAE occurring at JMP.

Study Contact at GSK Biologicals for Reporting Serious Adverse Events
Manager Clinical Safety Vaccines

GSK Biologicals Clinical Safety Physician,
GlaxoSmithKline Biologicals,
Rue de l'Institut 89, 1330 Rixensart,
Belgium.

Tel: +32.2.656.87.98
Fax: +32.2.656.80.09
Mobile phone for 7/7 day availability: +32.477.40.47.13 or +32.472.90.66.00 or +32.474.53.48.68
email: rix.ct-safety-vac@gskbio.com

Clinical Research Associate (JMP)

Ezekiel Oenga,
Clinical Research Associate,
GlaxoSmithKline, PO Box 78392-00507, Nairobi, Kenya.

Tel: +254 20 6933 322
Fax: +254 20 558 329
email: ezekiel.r.oenga@gsk.com

Clinical Research Associate (KEMRI)

Ezekiel Oenga,
Clinical Research Associate,
GlaxoSmithKline, PO Box 78392-00507, Nairobi, Kenya.

Tel: +254 20 6933 322
Fax: +254 20 558 329
email: ezekiel.r.oenga@gsk.com

Section 4.1.3.

The EPI program offers all children BCG at birth, DTPw/~~Hib/~~HepB and Oral Polio Vaccine at 1, 2 and 3 months and Measles at 9 months.

Section 4.1.4.

A Prevention of Mother to Child Transmission of HIV programme has been operational in Korogwe since 2005 and the Ministry of Health anti-viral programme is expected to reach Korogwe in the latter quarter of 2006. All patients must take part in extensive counseling before treatment. Highly Active Anti Retroviral Therapy (HAART) will be available to ***volunteers (if diagnosed at screening) or enrolled subjects (diagnosed during the study) with HIV*** ~~at Korogwe by the time of study start~~.

Section 4.2.3

The EPI program offers all children BCG at birth, DTPw/Hib/HepB at 1, 2 and 3 months, Oral Polio Vaccine at 1, 2 and 3 ~~weeks~~ ***months*** and Measles at 9 months.

…

Referral to tertiary level facilities is rarely required, but, for example in cases requiring neurosurgery or cases of hematological malignancy, referrals are made to ***the***

government Coast General Hospital in Mombasa ***or the privately-run Aga Khan hospital.***

Section 5.4.

Trained field workers under the supervision of the Principal Investigators will visit each enrolled child at daily intervals for Days 1 to 6 following each vaccination (see below in detailed study procedure; Section 5.14). In the event that the field worker finds any Grade 3 solicited general or ***Grade 3*** unsolicited symptoms, the volunteer will be examined by a study clinician. Any further clinical data, including treatment provided, will be written on diary cards and clinic forms and transcribed onto the CRF. If the physician finds that the volunteer has experienced an SAE the appropriate measures will be taken to report this (See Section 8.7).

Section 5.7.1.

Parents/guardians of children will be advised to seek first line health care at the dispensary at which they were enrolled. These dispensaries are staffed by clinically-qualified health workers who are on call 24 hours a day, 7 days a week. If needed participants will be referred, and if urgent, transported to Korogwe Hospital. ~~Field workers will also be readily available to subject's families during the course of the trial and will facilitate referral and access to care.~~ ***Families will also be informed that field workers will be readily available to facilitate access to care during the course of the trial. Field workers will have access to a study clinician for consultation by mobile phone 24 hours a day, 7 days a week and transport is available 24 hours a day, 7 days a week if necessary***

Section 5.7.2.

Subject's families will be advised to seek first line health care at Junju Dispensary or, out of normal hours at ~~Vipingo Health Center~~ ~~and~~ Kilifi District Hospital. ***Families will also be informed that field workers will be readily available to facilitate access to care*** ~~Field workers will also be readily available to subject's families~~ during the course of the trial. Field workers will have access to a study clinician for consultation by mobile phone 24 hours a day, 7 days a week and transport is available 24 hours a day, 7 days a week if necessary.

Junju Dispensary is open during normal working hours. Subjects presented to Junju Dispensary will be seen by clinically-qualified personnel. ~~Vipingo Health Center provides a service 24 hours a day, 7 days a week. All subjects will be seen by clinically-qualified personnel.~~ Transfer from ***Junju, or from villages*** ~~both facilities~~ to Kilifi District Hospital will be facilitated if necessary.

At Kilifi District Hospital KEMRI personnel are available 24 hours a day 7 days a week to receive, investigate and document study subjects. The responsibility for nursing and medical care is shared with the government service. Laboratory and radiological investigation will be carried out when appropriate. Treatment for medical conditions will be given according to the standard treatment regimens of Kenya. A detailed description of the healthcare system available in the study area is provided in Section 4.2. Tertiary

care will be provided by ***either*** Coast General Hospital in Mombasa ***, or, for selected conditions, the Aga Khan hospital in Mombasa*** (refer to Section 4.2.3).

Section 5.9.

If the child is documented to be febrile and the rapid test is positive, the field worker will give the child antimalarial chemotherapy and-inform a study ~~physician~~ ***clinician***. Mobile phones will be provided to study staff. Appropriate to the clinical features of the illness, transfer of children will be arranged to a health facility, if necessary..

Section 5.10.

When a subject presents spontaneously to a fieldworker, the axillary temperature will be taken and if ≥ 37.5 °C a blood slide and a rapid test (OptiMal®, Flow Incorporated, Oregon, USA) to determine malaria parasitemia will be taken.

If the child is documented to be febrile and the rapid test is positive, the field worker will give the child antimalarial chemotherapy and inform a study clinician. Mobile phones will be provided to study staff. Appropriate to the clinical features of the illness, transfer of children will be arranged to a health facility, if necessary. If the child is afebrile or the rapid test is negative, the case will be discussed with a study physician. Appropriate to the clinical features of the illness, the child's parent(s)/guardian(s) will be advised to attend their nearest health facility to be reviewed by clinically qualified personnel (medical or nursing) or transfer will be arranged to the secondary care facility.

Section 5.11.

Cross sectional surveys ~~for monitoring of cases of Malaria~~ [Section Title amended]

Section 5.13.

[Note that 'recording of bednet usage & residual spraying' has moved from Clinic Visits 6 and 7 to Field Worker Visit Code #s 55 and 86 respectively. In addition 'complete blood count' will not be measured on Month 6½ and 14; instead Hemoglobin will be measured at these visits].

	Recruit/ Screen	DOUBLE-BLIND PHASE										SINGLE-BLIND	
		VACCINATION						ACD				ACD	
		1		2		3							
		PCD										PCD	
**Study Month**		**0**		**1**		**2**			**3**		**6½**	**Month 7 to 13½**	**14**
**Study Day**	**-60 to 0**	**0**	**1 to 6**	**30**	**31 to 36**	**60**	**61 to 66**	***N***	**90**				
**Clinic Visit**	**1**	**2**		**3**		**4**			**5**		**6A**		**7**
								**2 wkly visits**		**14 wkly visits**		**31 wkly visitsB**	
**Field Worker Visit Code #**			**21 to 26**		**27 to 32**		**33 to 38**	**39, 40**		**41 to 55**		**56 to 86B**	
**ACD Visit**								**1 to 2**	**3**	**4 to 17**	**18A**	**19 to 50B**	**51**
Vital Signs	~~●~~ ○	○		○		○			○		○		○
Recording of bednet usage***& residual spraying***										● *O*		● *P*	
Parasite Genotyping (for ACD)								● H	● H	● H	● ~~H~~	● H	● ~~H~~
Complete blood count K	●								●		~~●~~		~~●~~
T regulatory cells and function											●		
Anti-blood stage humoral immunity	●								●		●		●

N Visit 39 to occur between 14 days post Dose 3 of vaccine and 20 days post Dose 3 of vaccine
O Carried out on Field Worker Visit Code # 55 ONLY
P Carried out on Field Worker Visit Code # 86 ONLY

Clinic Visit 5: Cross-sectional visit for ACD
Day 90

- *In addition, blood will be drawn from all subjects for:*
  - Rapid test for malaria parasites ***(only if subject is febrile)***.
  - Blood slide for malaria parasites.
  - Blood test for malaria parasite genotyping.
  - ~~Complete Blood Count.~~

Amended 20 November 2006

Field Worker Visits 41 to 55: ACD visits

- ***Record bednet use & indoor residual spraying (Field Worker Visit 55 ONLY)***

Clinic Visit 6: Cross-sectional visit for ACD
Month 6½

- Blood sample to collect ~~4.5~~ ***5.0*** mL blood for analysis of:
  - ***hematology (complete blood count)***;
  - ***biochemistry (creatinine and ALT)***;
  - serology (antibodies to CS***, anti-blood-stage humoral immunity***);
  - CMI:
    At KEMRI, Kenya only: CS-specific T-cells (ELISPOT).
- *In addition, blood will be drawn from all subjects for:*
  - Rapid test for malaria parasites ***(only if subject is febrile)***;
  - ~~Complete Blood Count.~~

Field Worker Visits 56 to 86: ACD visits

- ***Record bednet use & indoor residual spraying (Field Worker Visit 86 ONLY)***

Clinic Visit 7: Final study visit
Month 14

- Blood sample to collect ~~4.8~~ ***5.0*** mL blood for analysis of:
- *In addition, blood will be drawn from all subjects for:*
  - Rapid test for malaria parasites ***(only if subject is febrile)***;
  - ~~Complete Blood Count.~~

5.15.2. Laboratory assays

Separation of serum from the blood samples and analysis of hematology and biochemistry will be carried out at the laboratories of the respective trial sites.

Table 5 Laboratory immunological assays to be performed

Assay	Marker	Assay method	Test kit/ Manufacturer	Assay unit	Assay cut-off	Laboratory
CS-specific T-regulatory cells		FACS and RT-PCR	Investigator-developed assay			KEMRI, Kenya and JMP, Tanzania
Anti-blood stage humoral immunity						~~LSHTM~~ ***JMP & KEMRI & LSHTM‡***
						Designated collaborating laboratory

‡Note: samples taken at JMP may be sent to LSHTM for anti-blood stage humoral immunity testing. Samples taken at KEMRI will be tested in Kenya.

Section 5.15.3.

Plasma samples for anti-blood stage humoral immunity assessment ***from subjects taking part in Tanzania*** will be shipped to LSHTM and GSK Biologicals, Rixensart, Belgium (or designated collaborating laboratory). ***Samples may also be tested locally (assay details are provided in Appendix F) Plasma samples for anti-blood stage humoral immunity assessment from subjects taking part in Kenya will be shipped to GSK Biologicals, Rixensart, Belgium (or designated collaborating laboratory). Samples may also be tested locally (assay details are provided in Appendix F)***

Assessment of CS-specific T-cells (ELISPOT) will be done at KEMRI, Kenya, on samples from children enrolled at KEMRI, Kenya only ***(assay details are provided in Appendix F)***.

Any serum or plasma or blood cells not immediately used in immunological assays will be stored at -20°C or less and would only be used to assess the immune response to vaccination or to assess any potential toxicity of the vaccine ~~(assay details are provided in Appendix F).~~

Table 6 Summary of blood sampling time points/immunological assays and volumes to be collected

Blood sampling time point		Test	No. subjects	Laboratory	Priority ranking	Volume of whole blood required
Study month	Clinic Visit No.					
Day –60 to 0	1	Safety bloods	890	on-site	1	0.2 mL
		Anti-CS antibodies	890	CEVAC	2	1.0 mL
		Anti-HBs antibodies	890	GSK Biologicals*	3	
		CS-specific T-cells (ICS assay)	890	GSK Biologicals	4	2.1 mL
		Anti-blood-stage humoral immunity	890	GSK Biologicals*	7	0.3 mL
			890	~~LSHTM~~ ***JMP & KEMRI & LSHTM***	5	1.4 mL
		CS-specific T-cells (ELISPOT)	445**	KEMRI, Kenya	6	
				**Total for Clinic Visit 1**		**5.0 mL**
Month 3	5	Safety bloods	890	on-site	1	0.2 mL
		Anti-CS antibodies	890	CEVAC	2	1.0 mL
		Anti-HBs antibodies	890	GSK Biologicals*	3	
		CS-specific T-cells (ICS assay)	890	GSK Biologicals	4	2.1 mL
		Anti-blood-stage humoral immunity	890	GSK Biologicals*	7	0.3 mL
			890	~~LSHTM~~ ***JMP & KEMRI & LSHTM***	5	1.4 mL
		CS-specific T-cells (ELISPOT)	445**	KEMRI, Kenya	6	
				**Total for Clinic Visit 5**		**5.0 mL**
Month 6½	6†	***Safety Bloods***	***890***	***on-site***	***1***	***0.2***
		Anti-CS antibodies	890	CEVAC	~~1~~ ***2***	0.5 mL
		T-regulatory cells & function	890	JMP and KEMRI	~~2~~ ***3***	~~4.0~~***4.3*** mL
				**Total for Clinic Visit 6**		**~~4.5~~ *5.0* mL**
Month 14	7	***Safety Bloods***	***890***	***on-site***	***1***	***0.2***
		Anti-CS antibodies	890	CEVAC	~~1~~ ***2***	1.0 mL
		Anti-HBs antibodies	890	GSK Biologicals*	~~2~~ ***3***	
		CS-specific T-cells (ICS assay)	890	GSK Biologicals	~~3~~ ***4***	2.1 mL
		Anti-blood-stage humoral immunity	890	GSK Biologicals*	~~6~~ ***7***	0.3 mL
			890	~~LSHTM~~ ***JMP & KEMRI & LSHTM***	~~4~~ ***5***	~~1.4~~ ***1.6***mL
		CS-specific T-cells (ELISPOT)	445**	KEMRI, Kenya	~~5~~ ***6***	
				**Total for Clinic Visit 7**		**5.0 mL**

*: or designated laboratory **: to be carried out on all subjects at KEMRI, Kenya WB ; Whole blood
† subjects with axillary temperature >37.5°C will also have blood drawn for a malaria rapid test for the purposes of ACD
CMI: Cell Mediated Immunity **ICS:** Intracellular Cytokine Staining

Section 5.16

Data for the calculation of covariates will be collected during this study (refer to Section 10.9.5). Study center, bednet use, geographical area, distance from health center and altitude of subject's permanent residence above sea level will be determined from the existing demographic surveillance systems in place at each site.

For bednets, a field worker will visit the home of the subject during the study (refer to Section 5.13 for timepoints) and make a direct observation of whether the child's bed has a bed net and if so, whether or not it is intact. He will ask the family whether the bednet is impregnated.

For indoor residual spraying, the field worker will ask whether the the child's bedroom has been sprayed by a residual spraying program. Refer to Section 5.13 for timepoints of the fieldworker's visit.

Section 6.9

Any Vitamin A administered from 2 months prior to Dose 1 to 30 days post Dose 3.

Table 7 Summary of time periods between which different classes of concomitant medication/treatment/vaccination must be recorded

**2 months prior to Dose 1 → Dose 1**	All treatments listed as elimination criteria in Section 4.6*, all antimalarials***, Vitamin A***
**Dose 1 → 30 Days post Dose 3**	All antipyretic, analgesic, antibiotic, antimalarial and any treatments listed as elimination criteria in Section 4.6*, all vaccinations***, Vitamin A***
**31 Days post Dose 3 → Final Study Visit**	All treatments listed as elimination criteria in Section 4.6*, all antimalarials, all ~~vaccines~~ ***vaccinations***

* e.g. any immunoglobulins, other blood products and any immune modifying drugs

Section 8.5.1

Table 9 Acceptable/normal ranges for blood testing

	Acceptable limit/ normal range	Toxicity grading scale			
		Grade 1	Grade 2	Grade 3	Grade 4
Platelets[†]	≥ ~~100~~ ***75*** x 10^3 /µL	50 to ~~99~~ ***74*** x 10^3 /µL	25 to 49 x 10^3 /µL	< 25 x 10^3 /µL	< 25 x 10^3 /µL & clinical signs of bleeding

Section 8.7.2

Clinical Research Associate (JMP)

Ezekiel Oenga,
Clinical Research Associate,
GlaxoSmithKline, PO Box 78392-00507, Nairobi, Kenya.

Tel: +254 20 6933 322
Fax: +254 20 558 329
email: ezekiel.r.oenga@gsk.com

Clinical Research Associate (KEMRI)

Ezekiel Oenga,
Clinical Research Associate,
GlaxoSmithKline, PO Box 78392-00507, Nairobi, Kenya.

Tel: +254 20 6933 322
Fax: +254 20 558 329
email: ezekiel.r.oenga@gsk.com

Section 10.5

10.5 Case Definitions for Malaria

Primary malaria case definition	***P falciparum infection > 2 500 parasites per μL in a child with a documented fever ≥37.5°C (axillary) detected by ACD or PCD.***
Secondary malaria case definition	***P falciparum infection > 0 parasites per μL in a child with a documented fever ≥37.5°C (axillary) detected by ACD or PCD.***

Section 10.9.5.

The risk for malaria and development of clinical disease depends on numerous factors related to the parasite, host and vector biology. In addition to demographic data, covariates to be collected in this study will include:

- study center.
- bednet use.
- ***residual spraying***
- geographical area.
- distance from health center.
- altitude above sea level of subject's permanent residence.

Covariates included in the adjustment for efficacy will be defined in the Report Analysis Plan.

Appendix C

Children whose parents/guardians consent for them to enter the study will be invited to ~~come to the maternal child health centre and~~ be screened according to the procedures outlined in the Study Protocol.

GlaxoSmithKline Biologicals

Clinical Research & Development

Protocol Amendment Approval

**eTrack study number**	106367
**eTrack abbreviated title**	Malaria-049
**IND number**	BB-IND 12937
**Protocol title:**	A Phase IIb randomized, double-blind, controlled study of the efficacy against episodes of clinical malaria due to *Plasmodium falciparum* infection of GlaxoSmithKline Biologicals' candidate vaccine RTS,S/AS01E, administered IM according to a 0, 1, 2-month vaccination schedule in children aged 5 months to 17 months living in Tanzania and Kenya
**Amendment number:**	**Amendment 2**
**Amendment date:**	**20 November 2006**
**Co-ordinating author:**	Conor Cahill, Scientific Writer

Rationale/background for changes:

Some errors relating to the Cell Mediated Immunity testing planned for the study have been corrected. 'Status' will not be recorded on the CRF as it is captured as part of routine monitoring onto source documents. The text relating to the collection of concomitant medications has been amended to ensure that only systemic antibiotic drugs (i.e. not topical antibiotics) are recorded in the CRF. In addition a new staff member at MVI has been added to the authors list

Amended text has been included in *bold italics* in the following sections, deletions indicated by strikethrough:

Cover Material:

- ***Terrell Carter, Technical Coordinator***

Section 5.13

Table 3 List of study procedures

	Recruit/ Screen	DOUBLE-BLIND PHASE										SINGLE-BLIND	
		VACCINATION						ACD				ACD	
		1		2		3							
		PCD										PCD	
**Study Month**		0		1		2			3		6½	Month 7 to 13½	14
**Study Day**	-60 to 0	0	1 to 6	30	31 to 36	60	61 to 66	N	90				
**Clinic Visit**	1	2		3		4			5		6[A]		7
								2 wkly visits		14 wkly visits		31 wkly visits[B]	
**Field Worker Visit Code #**			21 to 26		27 to 32		33 to 38	39, 40		41 to 55		56 to 86[B]	
**ACD Visit**								1 to 2	3	4 to 17	18[A]	19 to 50[B]	51
**STUDY PROCEDURES**													
~~Record status [G]~~								~~●~~	~~●~~	~~●~~	~~●~~	~~●~~	~~●~~
Anti-blood stage humoral immunity	● [Q]								● [Q]		● [Q]		● [Q]

[C] all antimalarial, ***systemic*** antibiotic and antipyretic medication to be recorded

~~[G] 'Status' should be recorded as 'present', 'unavailable on day of visit', 'traveled', 'deceased', 'other'~~

[Q] ***Samples from Tanzania will be analyzed at LSHTM and/or their collaborating laboratory, at GSK Biologicals and/or their collaborating laboratory, and in Tanzania. Samples from Kenya will be analyzed at LSHTM and/or collaborating laboratory, at GSK Biologicals and/or collaborating laboratory, and in Kenya***

Section 5.14

Clinic Visit 5: Cross-sectional visit for ACD
Day 90

- *In addition, blood will be drawn from all subjects for:*
  - Rapid test for malaria parasites ***(only if subject is febrile)***.

Section 5.15.2

Table 5 Laboratory immunological assays to be performed

Assay	Marker	Assay method	Test kit/ Manufacturer	Assay unit	Assay cut-off	Laboratory
Anti-CS antibodies	R32LR	ELISA	In-house ELISA	EU/mL	0.5	CEVAC, University of Ghent, Belgium
Anti-HBs antibodies		EIA	AUSAB EIA ABBOTT*	mIU/mL	10**	GSK Biologicals†, Rixensart, Belgium
CS-specific T-cells (ICS)		ICS	In-house ICS	Number of cytokine(s) positive cells per 10^6 cells		GSK Biologicals†, Rixensart, Belgium
CS-specific short term effectors and central memory T-cells (Elispot)		Elispot	Investigator-developed assay	Number of cytokine secreting cells per 10^6 PBMCs		KEMRI, Kenya
CS-specific T-regulatory cells		FACS and RT-PCR	Investigator-developed assay			KEMRI, Kenya and JMP, Tanzania
Anti-blood stage humoral immunity						JMP & KEMRI & LSHTM‡
						Designated collaborating laboratory

*: or equivalent
**: seroprotective level
†: or designated validated laboratory
ELISA: Enzyme-linked Immunoabsorbent Assay
EIA: Enzyme immunoassay
CEVAC: Center for Vaccinology, Ghent University
ICS: Intra-cellular cytokine staining
LSHTM: London School of Hygiene and Tropical Medicine
FACS: Fluorescent activated cell sorter
PBMC: Peripheral blood mononuclear cells

~~‡Note: samples taken at JMP may be sent to LSHTM for anti-blood stage humoral immunity testing. Samples taken at KEMRI will be tested in Kenya.~~

‡ Samples from Tanzania will be analyzed at LSHTM and/or their collaborating laboratory, at GSK Biologicals and/or their collaborating laboratory, and in Tanzania. Samples from Kenya will be analyzed at LSHTM and/or collaborating laboratory, at GSK Biologicals and/or collaborating laboratory, and in Kenya

Section 5.15.3

Plasma samples for anti-blood stage humoral immunity assessment from subjects taking part in Tanzania will be shipped to LSHTM and GSK Biologicals, Rixensart, Belgium (or designated collaborating ~~laboratory~~ ***laboratories***). Samples may also be tested locally (assay details are provided in Appendix F). Plasma samples for anti-blood stage humoral immunity assessment from subjects taking part in Kenya will be shipped to ***LSHTM and*** GSK Biologicals, Rixensart, Belgium (or designated collaborating ~~laboratory~~ ***laboratories***). Samples ***from both Kenya and Tanzania*** may also be tested locally (assay details are provided in Appendix F).

Table 6 Summary of blood sampling time points/immunological assays and volumes to be collected

Blood sampling time point: Study month	Blood sampling time point: Clinic Visit No.	Test	No. subjects	Laboratory	Priority ranking	Volume of whole blood required
Day –60 to 0	1	Safety bloods	890	on-site	1	0.2 mL
		Anti-CS antibodies	890	CEVAC	2	1.0 mL
		Anti-HBs antibodies	890	GSK Biologicals*	3	
		CS-specific T-cells (ICS assay)	890	GSK Biologicals	4	2.1 mL
		Anti-blood-stage humoral immunity	890	GSK Biologicals*	~~7~~ ***8***	0.3 mL
			890	~~JMP & KEMRI &~~ LSHTM‡	5	1.4 mL
				JMP & KEMRI§	***6***	
		CS-specific T-cells (ELISPOT)	445**	KEMRI, Kenya	~~6~~ ***7***	
				**Total for Clinic Visit 1**		**5.0 mL**
Month 3	5	Safety bloods	890	on-site	1	0.2 mL
		Anti-CS antibodies	890	CEVAC	2	1.0 mL
		Anti-HBs antibodies	890	GSK Biologicals*	3	
		CS-specific T-cells (ICS assay)	890	GSK Biologicals	4	2.1 mL
		Anti-blood-stage humoral immunity	890	GSK Biologicals*	~~7~~ ***8***	0.3 mL
			890	~~JMP & KEMRI &~~ LSHTM‡	5	1.4 mL
				JMP & KEMRI§	***6***	
		CS-specific T-cells (ELISPOT)	445**	KEMRI, Kenya	~~6~~ ***7***	
				**Total for Clinic Visit 5**		**5.0 mL**
Month 6½	6†	Safety Bloods	890	on-site	1	0.2
		Anti-CS antibodies	890	CEVAC	2	0.5 mL
		T-regulatory cells & function	890	JMP and KEMRI	3	4.3 mL
				**Total for Clinic Visit 6**		5.0 mL
Month 14	7	Safety Bloods	890	on-site	1	0.2
		Anti-CS antibodies	890	CEVAC	2	1.0 mL
		Anti-HBs antibodies	890	GSK Biologicals*	3	
		CS-specific T-cells (ICS assay)	890	GSK Biologicals	4	2.1 mL
		Anti-blood-stage humoral immunity	890	GSK Biologicals*	~~7~~ ***8***	0.3 mL
			890	~~JMP & KEMRI &~~ LSHTM‡	5	1.6mL
				JMP & KEMRI§	***6***	
		CS-specific T-cells (ELISPOT)	445**	KEMRI, Kenya	~~6~~ ***7***	
				**Total for Clinic Visit 7**		5.0 mL

*: or designated laboratory **: to be carried out on all subjects at KEMRI, Kenya WB ; Whole blood

† subjects with axillary temperature >37.5°C will also have blood drawn for a malaria rapid test for the purposes of ACD

CMI: Cell Mediated Immunity **ICS:** Intracellular Cytokine Staining

‡Note: samples taken at JMP may be sent to LSHTM for anti-blood stage humoral immunity testing. ~~Samples taken at KEMRI will be tested in Kenya.~~

§Note: Samples from Tanzania will be analyzed at LSHTM and/or their collaborating laboratory, at GSK Biologicals and/or their collaborating laboratory, and in Tanzania. Samples from Kenya will be analyzed at LSHTM and/or collaborating laboratory, at GSK Biologicals and/or collaborating laboratory, and in Kenya

Section 6.9

All antipyretic, analgesic and ***systemic*** antibiotic drugs administered at ANY time during the period starting with administration of each dose and ending 30 days after each dose are to be recorded with generic name of the medication (trade names are allowed for

combination drugs, i.e., multi-component drugs), medical indication, total daily dose, route of administration, start and end dates of treatment.

Table 7 Summary of time periods between which different classes of concomitant medication/treatment/vaccination must be recorded

**2 months prior to Dose 1 → Dose 1**	All treatments listed as elimination criteria in Section 4.6*, all antimalarials, Vitamin A
**Dose 1 → 30 Days post Dose 3**	All antipyretic, analgesic, ***systemic*** antibiotic, antimalarial and any treatments listed as elimination criteria in Section 4.6*, all vaccinations, Vitamin A
**31 Days post Dose 3 → Final Study Visit**	All treatments listed as elimination criteria in Section 4.6*, all antimalarials, all vaccinations

* e.g. any immunoglobulins, other blood products and any immune modifying drugs

63f195821c4e0e87c982cb189f3de2c4

GlaxoSmithKline Biologicals

Clinical Research & Development

Protocol Amendment Approval

**eTrack study number**	106367
**eTrack abbreviated title**	Malaria-049
**IND number**	BB-IND 12937
**Protocol title:**	A Phase IIb randomized, double-blind, controlled study of the efficacy against episodes of clinical malaria due to *Plasmodium falciparum* infection of GlaxoSmithKline Biologicals' candidate vaccine RTS,S/AS01E, administered IM according to a 0, 1, 2-month vaccination schedule in children aged 5 months to 17 months living in Tanzania and Kenya
**Amendment number:**	**Amendment 3**
**Amendment date:**	**20 December 2006**
**Co-ordinating author:**	Conor Cahill, Scientific Writer

Rationale/background for changes:

Novartis Vaccines, the manufacturer of the rabies control vaccine for this study (Rabipur), have recalled the batches of vaccine purchased by GlaxoSmithKline Biologicals for use in this study. According to the issuer of the recall—the Defective Medicines Report Centre of the Medicines and Healthcare Products Regulatory Agency, UK—there is an issue regarding the integrity of the sterile packaging of the syringe and needle, and all doses must be returned to Novartis.

GlaxoSmithKline Biologicals has been unable to source sufficient doses of Rabipur to replace the recalled doses. They have procured a similar vaccine manufactured from Human Diploid Cells, produced by Sanofi-Pasteur as a replacement.

The informed consent has been amended to be in line the side effect profile provided in the package insert by Sanofi Pasteur: the package insert states "***In extremely rare cases, people who have received the rabies vaccine have developed abnormalities of the nerves.*** It should be noted that the rate of neurological side effects associated with modern cell culture vaccines such as those of Sanofi Pasteur and Novartis are substantially lower than historical nerve-tissue vaccines.

In this amendment, 'Rabipur' has been changed to 'Sanofi-Pasteur's Human Diploid Cell Rabies vaccine', and other relevant information amended wherever necessary.

63f195821c4e0e87c982cb189f3de2c4

Amended text has been included in *bold italics* in the following sections, deletions indicated by strikethrough:

Cover Material

**Study vaccines**	GlaxoSmithKline Biologicals' candidate *Plasmodium falciparum* malaria vaccine RTS,S/AS01E (0.5 mL dose) and ***Sanofi-Pasteur's*** ~~Rabipur®~~ Rabies Vaccine ***produced from Human Diploid Cells***.

Glossary of Terms

Investigational product:	A pharmaceutical form of an active ingredient or placebo being tested or used as a reference in a clinical trial, including a product with a marketing authorization when used in a way different from the approved form, or when used for an unapproved indication, or when used to gain further information about an approved use. The investigational products for this study are RTS,S/AS01E and ***Sanofi-Pasteur's Human Diploid Cell Rabies Vaccine*** ~~Rabipur~~.

Section 1.6.1.

Rabies vaccine has been chosen as the comparator because: 1) volunteers will benefit from receiving Rabies vaccine as rabid animals occur in the study area and 2) Rabies vaccine can be administered on a 0, 1, 2-month schedule.

Sanofi-Pasteur's Human Diploid Cell Rabies Vaccine will be used. According to product information distributed by Sanofi Pasteur Inc, PA, USA in December 2005,

> ***"High titer antibody responses of the Sanofi Pasteur SA Rabies Vaccine made in human diploid cells have been demonstrated in trials conducted in England [Aoki 1975], Germany [Cox, 1976; Kuwert, 1978], France [Ajjan, 1978] and Belgium [Costy-Berger, 1978]. Seroconversion was often obtained with only one dose. With two doses one month apart, 100% of the recipients developed specific antibody and the geometric mean titer of the group was approximately 10 international units. In the US, Sanofi Pasteur SA Rabies Vaccine resulted in geometric mean titers (GMT) of 12.9 IU/mL at Day 49 and 5.1 IU/mL at Day 90 when three doses were given intramuscularly during the course of one month. The range of antibody responses was 2.8 to 55.0 IU/mL at Day 49 and 1.8 to 12.4 IU at Day 90 [Bernard 1982]. The definition of a minimally accepted antibody titer varies among laboratories and is influenced by the type of test conducted. CDC currently specifies a 1:5 titer (complete inhibition) by the rapid fluorescent focus inhibition test (RFFIT) as acceptable. The World Health Organization (WHO) specifies a titer of 0.5 IU".***

~~Chiron's Rabipur® vaccine will be used. When the Rabipur Rabies vaccine is administered according to the recommended vaccination schedule (days 0, 7, 21), nearly 100% of subjects attain a protective titer. In two studies carried out in the US in 101 subjects, protective antibody titers > 0.5 IU/mL were obtained by day 28 in all subjects. In studies carried out in Thailand in 22 subjects, and in Croatia in 25 subjects, antibody~~

~~titers of > 0.5 IU/mL were obtained by day 14 (injections on days 0, 7, 21) in all subjects [Dreesen, 1989; Nicholson, 1987; Vodopija, 1986; Wasi, 1986].~~

Section 5.1.5.

~~Chiron's Rabipur~~® ***Sanofi-Pasteur's Human Diploid Cell Rabies Vaccine*** will be used as the control vaccine for this trial; half of the children enrolled will be vaccinated with the control regimen.

Section 6.1.2.

Sanofi-Pasteur's Human Diploid Cell Rabies Vaccine is a sterile freeze-dried suspension of rabies virus prepared from strain PM 1503-3M obtained from the Wistar Institute, Philadelphia, PA.

~~Rabipur®, is a sterile freeze-dried vaccine obtained by growing the fixed-virus strain Flury LEP in primary cultures of chicken fibroblasts.~~

The potency of one dose (1.0 mL) ~~Rabipur~~ of ***Sanofi-Pasteur's Human Diploid Cell Rabies Vaccine*** is at least 2.5 IU of rabies antigen. ***Sanofi-Pasteur's Human Diploid Cell Rabies Vaccine*** ~~Rabipur~~ is a ***creamy white to orange*** ~~white~~, freeze-dried vaccine for reconstitution with the diluent prior to use; the reconstituted vaccine is a clear to slightly opaque, colorless suspension.

A dose of 1.0 mL will be injected. The presentation of the reconstituted vaccine is as a ***pink to red liquid*** ~~clear or slightly opaque suspension~~. ***One dose of Sanofi-Pasteur's Human Diploid Cell Rabies Vaccine contains less than 100 mg albumin, less than 150 µg neomycin sulphate and 20 µg phenol red indicator. The vaccine contains no preservative or stabilizer.***

Section 6.2.2.

Disinfect top of vaccine vial with alcohol swabs and let dry. Inject the entire contents of the diluent ~~ampoule~~ ***syringe*** into the vaccine vial. Keeping the syringe and needle in place, the freeze-dried vaccine is dissolved by gently shaking the vial. Wait for 1 minute to ensure complete dissolution of vial contents before withdrawing a sufficient volume to provide a 1.0 mL dose still using the original needle and original syringe. The original needle should then be replaced with a fresh needle for IM injection. The reconstituted vaccine should be used immediately.

GlaxoSmithKline Biologicals

Clinical Research & Development

Protocol Amendment Approval

**eTrack study number**	106367
**eTrack abbreviated title**	Malaria-049
**IND number**	BB-IND 12937
**Protocol title:**	A Phase IIb randomized, double-blind, controlled study of the efficacy against episodes of clinical malaria due to *Plasmodium falciparum* infection of GlaxoSmithKline Biologicals' candidate vaccine RTS,S/AS01E, administered IM according to a 0, 1, 2-month vaccination schedule in children aged 5 months to 17 months living in Tanzania and Kenya
**Amendment number:**	**Amendment 4**
**Amendment date:**	**17 April 2008**
**Co-ordinating authors:**	Philip Bejon and Marie-Sylvie Remacle

Rationale/background for changes:

- The Central Study Coordinator contact for JMP (Korogwe) was modified.
- A list of Tables and a List of Figures were added to the protocol.
- The surveillance period post Dose 3 is extended for all subjects through the transmission season (October 2008 in Kilifi, September 2008 in Korogwe):

  Vaccinations began in March 2007. A series of logistic factors, including a change of supplier for the rabies control vaccine leading to interruption in delivery of the study vaccine, and lower than expected recruitment rates resulted in a variable delay of vacination, such that surveillance post Dose 3 began in May 2007 for some children, but in October 2007 for others. 12 months of surveillance post Dose 3 are planned according per protocol, so children will finish surveillance in the period June 2008-October 2008.

  The high malaria transmission season is June-October in Kilifi and March-August in Korogwe. In this amendment dated April 2008, we propose to continue active and passive case detection in all children until the end of the transmission season. This will result in a variable length of follow up, but all children will end surveillance on the same date in each site (October in Kilifi, September in Korogwe). Surveillance is therefore extended through the transmission season for all subjects and is likely to produce an increase in power.

There will be two additional visits. The first visit (Clinic Visit 8) will be to request informed consent. The second visit (Clinic Visit 9) at the close of study will allow to assess safety at the final time-point, and to obtain a cross-sectional bleed in order to study parasite prevalence at the end of the malaria transmission season. The duration of protection may be linked to the duration of immunogenicity, and so serum will be used from this final time-point to measure vaccine-induced antibodies to the circumsporozoite antigen (CS).

Amended text has been included in *bold italics* in the following sections, deletions indicated by strikethrough:

Sponsor Information:

4. Central Study Coordinator (JMP)

~~Isabelle Ramboer,~~ ***Jean François Stallaert***
Central Study Coordinator,
GlaxoSmithKline Biologicals,
Rue de l'Institut 89, 1330 Rixensart,
Belgium.
Tel: +32.2.656.~~68.20~~ ***94.28***
Fax: +32.2.656.80.44
email: ~~isabelle.ramboer@gskbio.com~~
jean-francois.j.stallaert@gskbio.com

Amended 17 April 2008

7. Study Contacts for Reporting of a Serious Adverse Event occurring at JMP

…

Central Study Coordinator (JMP)

~~Isabelle Ramboer,~~ ***Jean François Stallaert***
Central Study Coordinator,
GlaxoSmithKline Biologicals,
Rue de l'Institut 89, 1330 Rixensart,
Belgium.
Tel: +32.2.656.~~68.20~~ ***94.28***
Fax: +32.2.656.80.44
email: ~~isabelle.ramboer@gskbio.com~~
jean-francois.j.stallaert@gskbio.com

Amended 17 April 2008

Synopsis:

Tertiary objectives

- For RTS,S/AS01E when administered as three doses intramuscularly on a 0, 1, 2-month schedule to children aged 5 to 17 months living in malaria-endemic areas:
  - ***To assess safety until October 2008 in Kilifi and September 2008 in Korogwe.***

    Amended 17 April 2008
  - ***To assess efficacy against clinical malaria (detected on active and passive case detection) with*** **P. falciparum** ***from Dose 3 until October 2008 in Kilifi and September 2008 in Korogwe.***

    Amended 17 April 2008
  - ***to assess antibody responses to the circumpsorozoite (CS) antigen at a cross-sectional survey occurring in October 2008 in Kilifi and September 2008 in Korogwe.***

    Amended 17 April 2008

Study design

- All children will be followed by Active Case Detection (ACD) and Passive Case Detection (PCD) for a period extending to one year post Dose 3***, and then, under a separate consent, until October 2008 in Kilifi and September 2008 in Korogwe.***

  Amended 17 April 2008
- Two cross sectional surveys for efficacy will be performed at 4½ and 12 months post Dose 3 to determine parasite prevalence, parasite density and hemoglobin***, and under a separate consent, a third cross-sectional survey for efficacy will be performed in October 2008 in Kilifi and in September 2008 in Korogwe to determine parasite prevalence, parasite density and hemoglobin.***

  Amended 17 April 2008
- Anti-CS antibody titers will be determined at baseline, 1, 4½ and 12 months post Dose 3***, and under a separate consent, at a cross-sectional survey occurring in October 2008 in Kilifi and in September 2008 in Korogwe*** in all study participants.

  Amended 17 April 2008

Tertiary endpoints Efficacy

- Time to first case of malaria meeting the primary case definition over a period starting 14 days after Dose 3 and extending for 12 months ***and a period starting 14 days after Dose 3 and extending until October 2008 in Kilifi and September 2008 in Korogwe***.

  Amended 17 April 2008

- Time to first case of malaria meeting the secondary case definition over a period starting 14 days after Dose 3 and extending for 12 months ***and a period starting 14 days after Dose 3 and extending until October 2008 in Kilifi and September 2008 in Korogwe***.

Amended 17 April 2008

- Multiple events of malaria meeting the primary case definition over a period starting 14 days after Dose 3 and extending for 12 months ***and a period starting 14 days after Dose 3 and extending until October 2008 in Kilifi and September 2008 in Korogwe***.

Amended 17 April 2008

- Multiple events of malaria meeting the secondary case definition over a period starting 14 days after Dose 3 and extending for 12 months ***and a period starting 14 days after Dose 3 and extending until October 2008 in Kilifi and September 2008 in Korogwe***.

Amended 17 April 2008

- Parasite prevalence and density at 12 months post Dose 3 ***and in October 2008 in Kilifi and in September 2008 in Korogwe***.

Amended 17 April 2008

- Hemoglobin at 12 months post Dose 3 ***and in October 2008 in Kilifi and in September 2008 in Korogwe***.

Amended 17 April 2008

Tertiary endpoints Immunogenicity

- Anti-CS antibody titers at 12 months post Dose 3 (Month 14) ***and in October 2008 in Kilifi and in September 2008 in Korogwe***.

Amended 17 April 2008

Section 1: Introduction:

1.6.4 Rationale for study extension

Vaccinations began in March 2007. A series of logistic factors, including a change of supplier for the rabies control vaccine leading to interruption in delivery of the study vaccine, and lower than expected recruitment rates resulted in a variable delay of vacination, such that surveillance post Dose 3 began in May 2007 for some children, but in October 2007 for others. 12 months of surveillance post Dose 3 are planned according per protocol, so children will finish surveillance in the period June 2008-October 2008.

The high malaria transmission season is June-October in Kilifi and March-August in Korogwe. In this amendment dated April 2008, we propose to continue active and passive case detection in all children until the end of the transmission season. This will result in a variable length of follow up, but all children will end surveillance on the

same date in each site (October in Kilifi, September in Korogwe). Surveillance is therefore extended through the transmission season for all subjects and is likely to produce an increase in power.

There will be two additional visits. The first visit (Clinic Visit 8) will be to request informed consent. The second visit (Clinic Visit 9) at the close of study will allow to assess safety at the final time-point, and to obtain a cross-sectional bleed in order to study parasite prevalence at the end of the malaria transmission season. The duration of protection may be linked to the duration of immunogenicity, and so serum will be used from this final time-point to measure vaccine-induced antibodies to the circumsporozoite antigen (CS).

Amended 17 April 2008

Section 2: Objectives:

2.3 Tertiary Objectives

- For RTS,S/AS01E when administered as three doses intramuscularly on a 0, 1, 2-month schedule to children aged 5 to 17 months living in malaria-endemic areas:
  - ***To assess safety until October 2008 in Kilifi and September 2008 in Korogwe.***

    Amended 17 April 2008
  - ***To assess efficacy against clinical malaria (detected on active and passive case detection) with* P. falciparum *from Dose 3 until October 2008 in Kilifi and September 2008 in Korogwe.***

    Amended 17 April 2008
  - ***to assess antibody responses to the circumpsorozoite (CS) antigen at a cross-sectional survey occurring in October 2008 in Kilifi and September 2008 in Korogwe.***

    Amended 17 April 2008

Section 3: Study design:

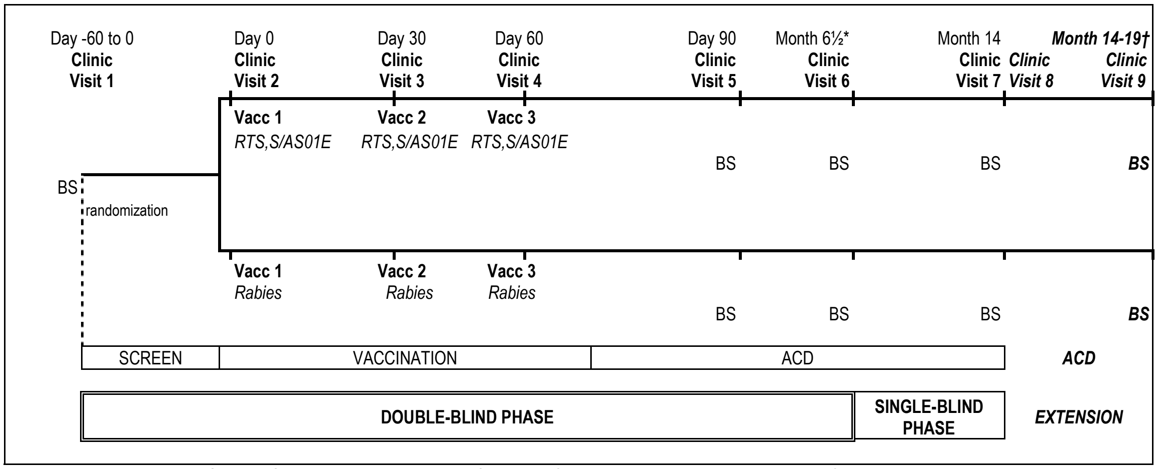

KEY: BS; Blood Sample. **Vacc;** Vaccination. **ACD;** Active Case Detection. ***Extension; extention of the single-blind phase***
Clinic Visit 7 and Clinic Visit 8 can take place on the same day. For subjects who do not present at Clinic Visit 7, an interval of 14 to 17 months post Clinic Visit 2 to Clinic Visit 8 is acceptable.
† Month 14-19 at Clinic Visit 9 corresponds to October 2008 in Kilifi and September 2008 in Korogwe
*In the event that malaria rates are lower in the year of the study, the cross sectional survey, unblinding and analysis will be delayed until sufficient cases have accumulated to have an adequately powered analysis.

Amended 17 April 2008

- All children will be followed by Active Case Detection (ACD) and Passive Case Detection (PCD) for a period extending to one year post Dose 3***, and then, under a separate consent, until October 2008 in Kilifi and September 2008 in Korogwe.***
  Amended 17 April 2008
- Two cross sectional surveys for efficacy will be performed at 4½ and 12 months post Dose 3 to determine parasite prevalence, parasite density and hemoglobin***, and under a separate consent, a third cross-sectional survey for efficacy will be performed in October 2008 in Kilifi and in September 2008 in Korogwe to determine parasite prevalence, parasite density and hemoglobin.***
  Amended 17 April 2008
- Anti-CS antibody titers will be determined at baseline, 1, 4½ and 12 months post Dose 3***, and under a separate consent, at a cross-sectional survey in October 2008 in Kilifi and in September 2008 in Korogwe*** in all study participants.
  Amended 17 April 2008

Section 5: Conduct of Study:

5.11 Cross sectional surveys

At the cross sectional surveys (Clinic Visit 6 and 7 ***and 9***) all children will have a blood slide taken for malaria parasitemia independent of whether they have symptomatology of malaria. To identify and treat children who have acute malaria at the time of the survey, the axillary temperature will be taken. If ≥ 37.5°C, a blood slide and a rapid test will be taken to determine malaria parasitemia. The same procedures as specified under ACD (Section 5.9) will then be followed.

Amended 17 April 2008

5.13 *Outline of study procedures*

Table 3 List of study procedures

		**DOUBLE-BLIND PHASE**										**SINGLE-BLIND**		***Extension SINGLE-BLIND***		
								**ACD**				**ACD**		***ACD***		
		**1**		**2**		**3**										
		**PCD**										**PCD**		***PCD***		
**Study Month**		**0**		**1**		**2**			**3**		**6½**	**Month 7 to 13½**	**14**			***14-19 (Oct/Sept 2008)‡***
**Study Day**	**-60 to 0**	**0**	**1 to 6**	**30**	**31 to 36**	**60**	**61 to 66**	N	**90**							
**Clinic Visit**	**1**	**2**		**3**		**4**			**5**		**6[A]**		**7**	***8†***		***9***
								**2 wkly visits**		**14 wkly visits**		**31 wkly visits[B]**			***1-20 wkly visits***	
**Field Worker Visit Code #**			**21 to 26**		**27 to 32**		**33 to 38**	**39, 40**		**41 to 55**		**56 to 86[B]**			***87 to 88 to 107***	
**ACD Visit**								**1 to 2**	**3**	**4 to 17**	**18[A]**	**19 to 50[B]**	**51**		***52 TO 53/73***	
**STUDY PROCEDURES**																
Informed consent	●													●†		
Medical history	●															
Vital Signs	○	○		○		○			○		○		○	○		○
Complete physical examination	●	○		○		○			○		○		○	○		○
Measure body weight	○	●											●	○		●
Assign Subject Number	●															
Temperature (for vaccination)		●	●	●	●	●	●									
Check inclusion/exclusion criteria	●	○														
Check elimination criteria		●		●		●										
Check contraindications to vaccination		●		●		●										
Randomization		●														
Administer RTS,S/AS01E or Rabies vaccine		●		●		●										
Recording of bednet usage& residual spraying										● [O]		● [P]				
Recording of concomitant medication		● [C]	● [C]	● [C]	● [C]	● [C]	● [C]	● [C]	● [C]	● [D]	● [D]	● [D]	● [D]	● *[D]*	● *[D]*	● *[D]*
**SAFETY DATA COLLECTION**																
Recording of solicited symptoms		●		●		●										
Recording of solicited symptoms (FW)			●		●		●									
Recording of unsolicited AEs within 1 month post-vaccination		●		●		●			●							
Recording of unsolicited Aes (FW)			●		●		●	●								

Morbidity surveillance/record SAEs	●[E]	●[F]	●[F]	●[F]	●[F]	●[F]	●[F]	●[F]	●[F]	●[F]	●[F]	●[F]	●[F]	●*[F]*	●*[F]*	●*[F]*
**EFFICACY DATA COLLECTION**																
Record baseline covariates	●															
Temperature (for ACD)								●	●	●	●	●	●	●*[S]*	●	●
Blood slide for malaria parasites (for ACD)								●[H]	●[H]	●[H]	●	●[H]	●	●*[S]*	●*[H]*	●*[R]*
Rapid test for malaria parasites (for ACD)								●[H]	●[H]	●[H]	●[H]	●[H]	●[H]	●*[S]*	●*[H]*	●*[R]*
Parasite Genotyping (for ACD)								●[H]	●[H]	●[H]	●	●[H]	●	●*[S]*	●*[H]*	●*[R]*
Passive Case Detection [J]		●	●	●	●	●	●	●	●	●	●	●	●	●	●	●
**SAFETY LABS**																
Complete blood count [K]	●								●		●		●			
Creatinine, ALT	●								●		●		●			
Hemoglobin																●*[R]*
**INVESTIGATIONAL ASSAYS**																
Antibodies to CS	●								●		●		●			●*[R]*
Antibodies to HBs	●								●				●			
CS-specific T-cells (ICS assay)	●								●				●			
T regulatory cells and function											●					
Anti-blood stage humoral immunity	●[Q]								●[Q]		●[Q]		●[Q]			
CS-specific T-cells (ELISPOT assay) [L]	●								●		●		●			
**FINAL ANALYSIS**											●[M]					
**STUDY CONCLUSION**													●			
**STUDY CONCLUSION**																●

for table notes, please refer overleaf…

Amended 23 October 2006
Amended 20 November 2006
Amended 17 April 2008

63f195821c4e0e87c982cb189f3de2c4

ACD; Active Case Detection PCD; Passive Case Detection ICS: Intracellular Cytokine Staining FW: Field Worker

[A] The survey will be postponed in the event of low case rates occurring during the first four months of surveillance

[B] These visits will occur in the double blind phase in the event that the survey at Clinic Visit 6 is postponed

[C] all antimalarial, systemic antibiotic and antipyretic medication to be recorded

[D] record administration of immunosuppressants or other immune-modifying drugs during this period (for corticosteroids this means prednisone or equivalent, ≥ 0.5 mg/kg/day. Inhaled or topical steroids are allowed and should not be recorded. ALSO all immunoglobulins and blood products should be recorded during this period. ALSO any antimalarials.

[E] SAEs related to study procedures will be collected

[F] Procedure for PCD will be followed

Amended 20 November 2006

[H] If documented fever

[J] Where the subject is febrile, blood test, rapid test, and parasite genotyping will be performed

[K] Includes analysis of hemoglobin, total white cell count and platelets.

[L] Done only in study participants enrolled by KEMRI, Kenya

[M] Final analysis will be delayed in the event of low case rates occurring during the first four months of surveillance

[N] Visit 39 to occur between 14 days post Dose 3 of vaccine and 20 days post Dose 3 of vaccine

[O] Carried out on Field Worker Visit Code # 55 ONLY

[P] Carried out on Field Worker Visit Code # 86 ONLY

Amended 23 October 2006

[Q] Samples from Tanzania will be analyzed at LSHTM and/or their collaborating laboratory, at GSK Biologicals and/or their collaborating laboratory, and in Tanzania. Samples from Kenya will be analyzed at LSHTM and/or collaborating laboratory, at GSK Biologicals and/or collaborating laboratory, and in Kenya

Amended 20 November 2006

● is used to indicate a study procedure that requires documentation in the individual CRF and ○ is used to indicate a study procedure that does not require documentation in the individual CRF. *SAEs that are related to study participation or are related to a concurrent medication will be collected and recorded from the time the subject consents to participate in the study until they are discharged.

† Clinic Visit 7 and Clinic Visit 8 can take place on the same day. For subjects who do not present at Clinic Visit 7, an interval of 14 to 17 months post Clinic Visit 2 to Clinic Visit 8 is acceptable. Subjects will be reconsented for the participation in the surveillance during the extension of the study.

‡The timing of visit 9 will be in October 2008 in Kilifi, and in September 2008 in Korogwe, in order to finish follow up at the same calender month at each site. This will mean a variable number of months after vaccination by individual children, as indicated in the table.

R Blood will not be drawn for hemoglobin or serology or parasite slide in the absence of fever from subjects who present for Clinic Visit 9 less than or equal to 14 days after Clinic Visit 7. Note that if the subject is febrile a rapid test and a parasite slide and genotyping specimens will be taken even if occurring in this period.

S If Clinic Visit 8 is more than 7 days after Clinic Visit 7 or if Clinic Visit 7 was not done.

Amended 17 April 2008

63f195821c4e0e87c982cb189f3de2c4

Table 4 Intervals between study visits

Interval	Length of interval
Clinic Visit 1 → Clinic Visit 2	0 to 60 days
Clinic Visit 2 → Clinic Visit 3	21 to 35 days
Clinic Visit 3 → Clinic Visit 4	21 to 35 days
Clinic Visit 4 → Clinic Visit 5	21 to 42 days
Clinic Visit 5 → Clinic Visit 7	12 months ± 30 days
Clinic Visit 7→ Clinic Visit 8*	***0 to 3 months***
Clinic Visit 9	***Korogwe: September 2008 ± 14 days***<br>***Kilifi: October 2008 ± 14 days***

**** Clinic Visit 7 and Clinic Visit 8 can take place on the same day. For subjects who do not present at Clinic Visit 7, an interval of 14 to 17 months post Clinic Visit 2 to Clinic Visit 8 is acceptable.***

Amended 17 April 2008

5.14 Detailed description of study stages/visits

Clinic Visit 8: Informed consent for study extension
Month 14 (up to Month 17)

Note: Clinic Visit 7 and Clinic Visit 8 can take place on the same day and up to 3 months post Clinic Visit 7, or for subjects who do not present at Clinic Visit 7, an interval of 14 to 17 months post Clinic Visit 2 to Clinic Visit 8 is acceptable.

- ***Check study identification card of vaccinee.***
- ***Obtain signed, dated, thumb printed informed consent from the parent(s)/guardians.***
- ***Record SAEs experienced by the vaccinee since the last visit.***
- ***Record concomitant medication.***
- ***Record status.***
- ***If Clinic Visit 8 is more than 7 days after Clinic Visit 7 or if Clinic Visit 7 was not done, record axillary temperature.***
- ***If subject has fever collect blood for:***
  - ***Rapid test for malaria parasites;***
  - ***Blood slide for malaria parasites;***
  - ***Blood test for malaria parasite genotyping.***

Amended 17 April 2008

Field Worker Visits 87 to 88 (last children to be recruited) or Visits 87 to 107 (earliest children to be recruited) : ACD visits

- ***Check study identification card of vaccinee.***
- ***Record SAEs experienced by the vaccinee since the last visit.***
- ***Record concomitant medication.***
- ***Record status.***
- ***Record axillary temperature.***
- ***If subject has fever collect blood for:***
  - ***Rapid test for malaria parasites;***
  - ***Blood slide for malaria parasites;***
  - ***Blood test for malaria parasite genotyping.***

Amended 17 April 2008

Clinic Visit 9: End of study extension visit
Month 14-19 (October 2008 for Kilifi; September 2008 for Korogwe)

- ***Check study identification card of vaccinee.***
- ***Carry out physical examination, measure body weight.***
- ***Record SAEs experienced by the vaccinee since the last visit.***
- ***Check elimination criteria.***
- ***Record concomitant medication.***
- ***Record axillary temperature of subject.***

If Clinic Visit 9 is more than 14 days after Clinic Visit 7 or if Clinic Visit 7 was not done:

- ***Blood sample to collect 1.2 mL blood for analysis of:***
  - ***Hemoglobin;***
  - ***serology (antibodies to CS);***
- ***In addition, blood will be drawn from all subjects for:***
  - ***Rapid test for malaria parasites (only if subject is febrile);***
  - ***Blood slide for malaria parasites;***
  - ***Blood test for malaria parasite genotyping;***

Amended 17 April 2008

5.15.3 Serology and cell-mediated immunity plan

Table 6 Summary of blood sampling time points/immunological assays and volumes to be collected

Blood sampling time point		Test	No. subjects	Laboratory	Priority ranking	Volume of whole blood required
Study month	Clinic Visit No.					
Day –60 to 0	1	Safety bloods	890	on-site	1	0.2 mL
		Anti-CS antibodies	890	CEVAC	2	1.0 mL
		Anti-HBs antibodies	890	GSK Biologicals*	3	
		CS-specific T-cells (ICS assay)	890	GSK Biologicals	4	2.1 mL
		Anti-blood-stage humoral immunity	890	GSK Biologicals*	8	0.3 mL
			890	LSHTM‡	5	1.4 mL
			890	JMP & KEMRI§	6	
		CS-specific T-cells (ELISPOT)	445**	KEMRI, Kenya	7	
				**Total for Clinic Visit 1**		**5.0 mL**
Month 3	5	Safety bloods	890	on-site	1	0.2 mL
		Anti-CS antibodies	890	CEVAC	2	1.0 mL
		Anti-HBs antibodies	890	GSK Biologicals*	3	
		CS-specific T-cells (ICS assay)	890	GSK Biologicals	4	2.1 mL
		Anti-blood-stage humoral immunity	890	GSK Biologicals*	8	0.3 mL
			890	LSHTM‡	5	1.4 mL
			890	JMP & KEMRI§	6	
		CS-specific T-cells (ELISPOT)	445**	KEMRI, Kenya	7	
				**Total for Clinic Visit 5**		**5.0 mL**
Month 6½	6†	Safety Bloods	890	on-site	1	0.2
		Anti-CS antibodies	890	CEVAC	2	0.5 mL
		T-regulatory cells & function	890	JMP and KEMRI	3	4.3 mL
				**Total for Clinic Visit 6**		5.0 mL
Month 14	7	Safety Bloods	890	on-site	1	0.2
		Anti-CS antibodies	890	CEVAC	2	1.0 mL
		Anti-HBs antibodies	890	GSK Biologicals*	3	
		CS-specific T-cells (ICS assay)	890	GSK Biologicals	4	2.1 mL
		Anti-blood-stage humoral immunity	890	GSK Biologicals*	8	0.3 mL
			890	LSHTM‡	5	1.6mL
			890	JMP & KEMRI§	6	
		CS-specific T-cells (ELISPOT)	445**	KEMRI, Kenya	7	
				**Total for Clinic Visit 7**		5.0 mL
Δ	***9***	***Hemoglobin***	***890***	***on-site***	***1***	***0.2***
		Anti-CS antibodies ***	***890***	***CEVAC***	***2***	***1.0 mL***
				Total for Clinic Visit 8		***1.2 mL***

*: or designated laboratory **: to be carried out on all subjects at KEMRI, Kenya WB: Whole blood ******: where Clinic Visits 7 and 9 are separated by a period of 14 days or less, the results of blood taken at Clinic Visit 7 will be used and there will be no second blood sample taken. Δ: The timing of Clinic Visit 9 will be in October 2008 in Kilifi, and in September 2008 in Korogwe, in order to finish follow up at the same calender month at each site. This will mean a variable number of months after vaccination by individual children, as indicated in the table.***

† subjects with axillary temperature >37.5°C will also have blood drawn for a malaria rapid test for the purposes of ACD

CMI: Cell Mediated Immunity **ICS:** Intracellular Cytokine Staining

‡Note: Samples from Tanzania will be analyzed at LSHTM and/or their collaborating laboratory, at GSK Biologicals and/or their collaborating laboratory, and in Tanzania. Samples from Kenya will be analyzed at LSHTM and/or collaborating laboratory, at GSK Biologicals and/or collaborating laboratory, and in Kenya

Amended 23 October 2006.

Amended 20 November 2006

Amended 17 April 2008

Section 6: Investigational products and administration:

6.9 Concomitant medication

Table 7 Summary of time periods between which different classes of concomitant medication/treatment/vaccination must be recorded

**2 months prior to Dose 1 → Dose 1**	All treatments listed as elimination criteria in Section 4.6*, all antimalarials, Vitamin A
**Dose 1 → 30 Days post Dose 3**	All antipyretic, analgesic, systemic antibiotic, antimalarial and any treatments listed as elimination criteria in Section 4.6*, all vaccinations, Vitamin A
**31 Days post Dose 3 → ~~*Final Study Visit*~~ *Clinic Visit 7***	All treatments listed as elimination criteria in Section 4.6*, all antimalarials, all vaccinations
Clinic Visit 8→Final study Visit (Clinic Visit 9)	***All treatments listed as elimination criteria in Section 4.6*, all antimalarials, all vaccinations***

* e.g. any immunoglobulins, other blood products and any immune modifying drugs

Amended 23 October 2006
Amended 20 November 2006
Amended 17 April 2008

Section 8: Adverse Events and Serious Adverse Events:

8.7.2 Completion and transmission of serious adverse event reports to GSK Biologicals

Study Contact at GSK Biologicals for Reporting Serious Adverse Events
Manager Clinical Safety Vaccines

Central Study Coordinator
(JMP)

~~Isabelle Ramboer,~~ ***Jean François Stallaert***
Central Study Coordinator,
GlaxoSmithKline Biologicals,
Rue de l'Institut 89, 1330 Rixensart,
Belgium.
Tel: +32.2.656.~~68.20~~ ***94.28***
Fax: +32.2.656.80.44
email: ~~isabelle.ramboer@gskbio.com~~ ***jean-francois.j.stallaert@gskbio.com***

Amended 17 April 2008

63f195821c4e0e87c982cb189f3de2c4

Section 10: Data Evaluation: Criteria for Evaluation of Objectives:

10.3 Tertiary endpoints

Tertiary endpoints Efficacy

- Time to first case of malaria meeting the primary case definition over a period starting 14 days after Dose 3 and extending for 12 months ***and a period starting 14 days after Dose 3 and extending until October 2008 in Kilifi and September 2008 in Korogwe***.

  Amended 17 April 2008
- Time to first case of malaria meeting the secondary case definition over a period starting 14 days after Dose 3 and extending for 12 months ***and a period starting 14 days after Dose 3 and extending until October 2008 in Kilifi and September 2008 in Korogwe***.

  Amended 17 April 2008
- Multiple events of malaria meeting the primary case definition over a period starting 14 days after Dose 3 and extending for 12 months ***and a period starting 14 days after Dose 3 and extending until October 2008 in Kilifi and September 2008 in Korogwe***.

  Amended 17 April 2008
- Multiple events of malaria meeting the secondary case definition over a period starting 14 days after Dose 3 and extending for 12 months ***and a period starting 14 days after Dose 3 and extending until October 2008 in Kilifi and September 2008 in Korogwe***.

  Amended 17 April 2008
- Parasite prevalence and density at 12 months post Dose 3 ***and in October 2008 in Kilifi and in September 2008 in Korogwe***.

  Amended 17 April 2008
- Hemoglobin at 12 months post Dose 3 ***and in October 2008 in Kilifi and in September 2008 in Korogwe***.

  Amended 17 April 2008

Tertiary endpoints Immunogenicity

- Anti-CS antibody titers at 12 months post Dose 3 (Month 14) ***and in October 2008 in Kilifi and in September 2008 in Korogwe***.

  Amended 17 April 2008

10.9.3 Cross-sectional analyses

Hemoglobin mean and standard deviation will be presented (following cross-sectional visit at Clinic Visit 6 and Month 14 ***and Clinic Visit 9***) and 95% confidence intervals will be calculated. The effect of the group will be evaluated using a non-parametric Wilcoxon test.

For the assessment of the parasite density at following cross-sectional visit at Clinic Visits 6 and 7 ***and 9***, a 2x2 table presenting the frequency of positive and negative will be presented. The effect of the group will be evaluated using the Fisher exact test. The

Geometric mean of those positive in each group will be calculated. Difference in geometric mean and 95% CI will be estimated. The effect of the group will be evaluated using a non-parametric Wilcoxon test.

Amended 17 April 2008

10.11.2 Analysis of safety

Analysis of the occurrence of SAEs will be performed on the Total Vaccinated Cohort. The proportion of subjects with an SAE, classified by the MedDRA preferred term level, reported from study start until ~~study conclusion (~~Clinic Visit 7 ***and from study start until Clinic Visit 9***~~)~~ will be tabulated with exact 95% CI.

Amended 17 April 2008

10.11.3.1. Anti-CS antibodies

The percentage of subjects seropositive for anti-CS (proportion of subjects with anti-CS antibody titers greater than or equal to 0.5 EU/mL) with 95% CI will be determined at Clinic Visit 7 ***and Clinic Visit 9***. Antibody titers will be summarized by GMT with 95% CI. Tables will be generated by vaccine group and by protection status (protected subjects are those recipients of RTS,S/AS01E who are not diagnosed with malaria meeting the primary case definition within the 4½ month ACD period). Comparisons between protected and non-protected may be performed using Wilcoxon Rank Sum tests.

Amended 17 April 2008

Appendix E: Shipment of Biological Samples:

The central study coordinator~~s, Isabelle Ramboer and~~ Jean François Stallaert and Clinical Research Associate (Ezekiel Oenga) should be informed 2 days before any shipment.

~~Isabelle Ramboer~~ ***Jean François Stallaert***, Rixensart, Belgium
Telephone: +32.2.656.~~68.20~~***94.28***
Fax: +32.2.656.80.44
email: ~~isabelle.ramboer@gskbio.com~~
jean-francois.j.stallaert@gskbio.com
Ezekiel Oenga, Nairobi, Kenya
Tel: +254 20 6933 322
Fax: +254 20 558 329
email: ezekiel.r.oenga@gsk.com

Jean François Stallaert, Rixensart, Belgium

Telephone: +32.2.656.94.28
Fax: +32.2.656.80.44
email: jean-francois.j.stallaert@gskbio.com

Amended 23 October 2006.
Amended 17 April 2008

63f195821c4e0e87c982cb189f3de2c4
